# A new twist for Stork-Danheiser products enabled by visible light mediated *trans*-cyclohexene formation; access to acyclic distal enones†

**DOI:** 10.1039/d1sc03774a

**Published:** 2022-07-22

**Authors:** Erik Lantz, Roukaya El Mokadem, Tim Schoch, Tyler Fleske, Jimmie D. Weaver

**Affiliations:** 107 Physical Science, Department of Chemistry, Oklahoma State University Stillwater Oklahoma 74078 USA Jimmie.Weaver@OKSTATE.EDU; Campus Box 3290 Chapel Hill NC 27599-3290 USA

## Abstract

Herein, we investigate the use of visible light to indirectly drive ring opening in unstrained 6- and 7-membered ring systems *via* reaction with a transiently generated *trans*-cycloalkene. Identification of conditions that capture visible light energy in the form of ring strain was key to success. Under mildly acidic conditions, cycloalkenols were shown to undergo formally endothermic ring-opening isomerization to give acyclic exo-methylene and distal ketones or aldehydes in high yields. Ultimately, this work demonstrates the ability of cycloalkenes to capture visible light energy and its use to drive both kinetically and thermally unfavorable rearrangements.

## Abstract 2

In this work, visible light drives a contrathermodynamic isomerization of the classic Stork-Danheiser products through an energy transfer process to yield acyclic distal enone(al) isomers. This is possible because the photochemical energy is transiently captured as ring strain as a trans-, or twisted, cyclohexene.

## Graphical abstract

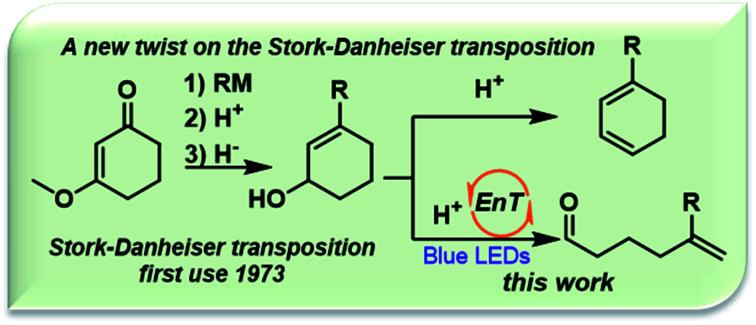

## Introduction

**Scheme 1:**
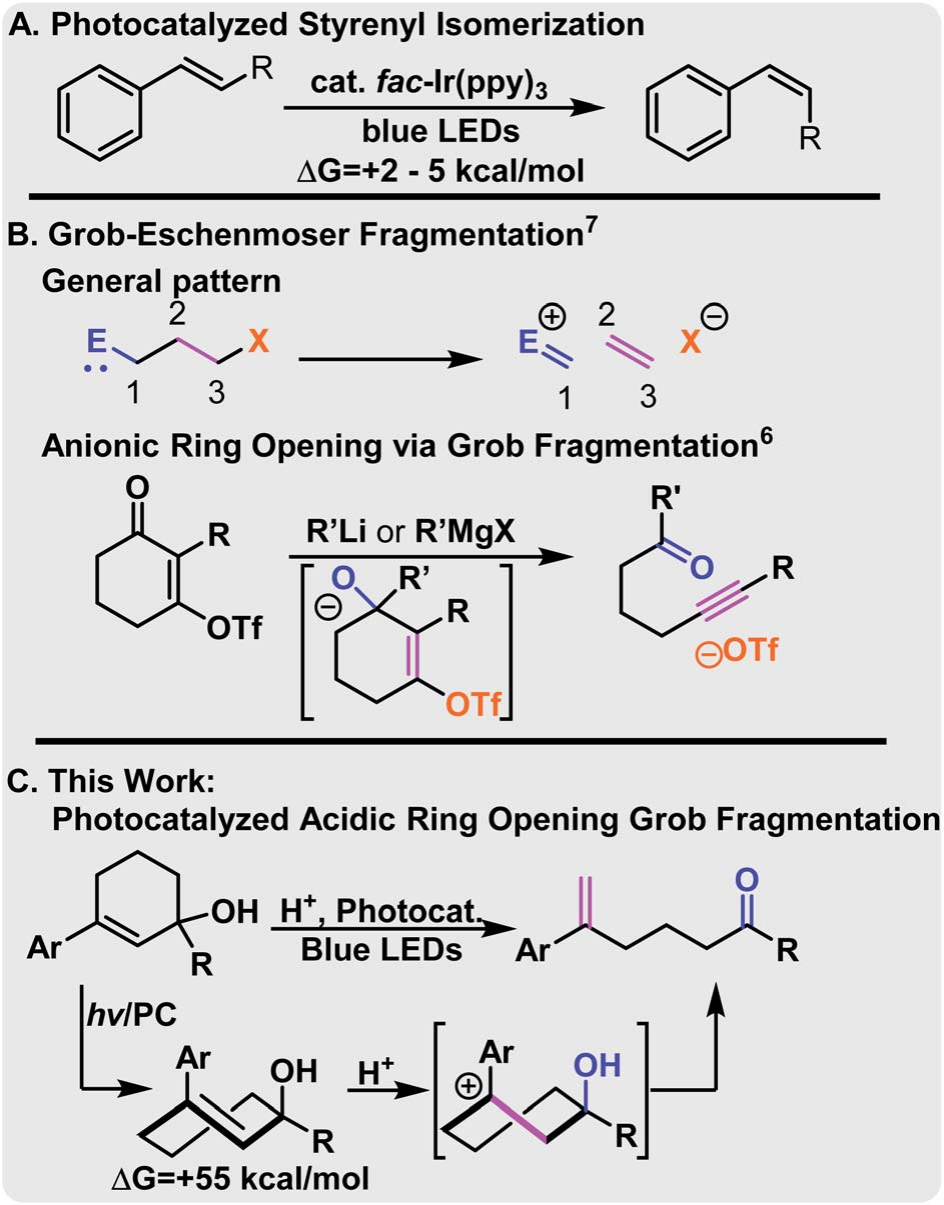
Conceptually Related work.^15^

Photocatalysis is a powerful and versatile tool in the chemist's toolbox. It offers attractive alternatives to harsh reaction conditions, provides alternative reaction pathways to achieve carbon–carbon bond formation, and often affords excellent functional group tolerance.^1–3^ While the majority of these processes involve electron transfer, visible light photocatalysis can also facilitate energy transfer to access electronically excited molecules. Typically, these molecules do not return to the ground state *via* the same path, opening the possibility of energetic pumping. We posited that identifying new well-behaved reactions that capture photochemical energy could allow the more general development of contrathermodynamic synthesis-that is synthesis that without energetic intervention would generally be considered thermally unfavorable.^4–8^ Our initial investigation in this area focused on the visible light photocatalytic contrathermodynamic isomerization of *E*-to *Z*-styrenoids (Scheme 1A).^9–11^ While well-behaved and synthetically useful, styrenoid isomerization captures little of the photochemical energy that is available in the excited state. The relaxed T1 state is ∼60 kcal mol^−1^ above the ground state *E*-alkene,^12–14^ however, the *Z*-isomer is only ∼2–5 kcal mol^−1^ above the *E*-isomer. Because lifetimes of excited state molecules are relatively short, we wanted to capture the energy in ground-state molecules which would lead to longer-lived, but energetically enriched molecules that could be used to drive mechanistically diverse synthetic processes. In this vein, we imbedded the styrenoid motif within small cycles that upon isomerization become strained. Indeed, each family of molecules that we and others have explored has displayed diverse reactivity. For instance, 1-aryl cyclohept-1-enes undergo thermal [2 + 2] reactions,^16^ while benzofused cycloheptenes undergo facile [3 + 2] reactions with dipoles such as azides.^12,17^ More recently, we and others have seen that highly strained *trans*-cyclohexenes^18^ also offer attractive synthetic opportunities.^13,19,20^ While conveniently generated using visible light and a triplet sensitizer, their ring strain leads to short lifetimes (9 μs at room temperature),^21,22^ and a remarkable increase in Brønsted basicity of the alkene that can be used to facilitate intramolecular cyclizations of appropriately substituted nucleophiles. We posited that this strain could also be utilized to drive C–C activation of cycloalkenols by preferential protonation of the alkene over the alcohol group.

The convenience and utility of the Grob–Eschenmoser fragmentation has been demonstrated by Dudley and others.^15^ Typically, the reaction takes place under anionic or base catalyzed conditions (Scheme 1B, top), and results in the extrusion of an anion. This typically requires installation of a leaving group and limits the reaction only to substrates that are insensitive to bases and strong nucleophiles. In contrast, in this case the strain energy of the *trans*-cyclohexene would drive the selective protonation of the alkene over the alcohol. With an allylic alcohol, we anticipated ring-opening would occur. Importantly, the use of stronger acids on the relaxed cycloalkenol leads to elimination, highlighting the enabling nature of photo-strained approach. The requisite cyclohexenols are readily available *via* the Stork-Danheiser transposition, which allows modular synthesis of the substrates, and expedited routes to natural products.^23–25^

## Results

We initiated our studies by subjecting cyclohexenol 1a to blue light, formic acid, and Ir(Fppy)_3_ (PC1), conditions which had previously been used to generate *trans*-cyclohexenes *in situ* (Table 1, entry 1).^13^ Indeed, we found evidence of ring-opening isomerization by GCMS which indicated an isomer was forming, while 1D NMR and 2D NMR analyses indicated that the C_2_–C_3_ bond was broken, and showed the disappearance of the carbinol, and the formation of both a new exo-methylene and carbonyl signals. While the photocatalytic isomerization takes place in a number of solvents, the fragmentation step was sensitive to the solvent choice, likely due to its polar nature, and worked best in dichloromethane (See ESI† for details).^10,19,26^ Control experiments (entries 2–4) showed that both light and the photocatalyst were critical to reaction, while a modest amount of reaction took place even in the absence of the acid. It is possible that this reactivity is result of adventitious water, or alternatively the alcohol itself.^27^ Formic acid was found to perform slightly worse than benzoic acid (entry 5), making benzoic acid the clear choice, given that it is a bench stable solid that can be washed away upon workup and is generally considered safe. A small screen of photocatalysts (entries 6–8) previously implicated in energy transfer processes revealed that higher emissive energy along with compactness seem to be important factors in effectively promoting the reaction which is consistent with previous observations.^9,28,29^ It has been suggested that the 3D-nature of the iridium dyes and heightened orbital overlap requirements in Dexter energy transfer processes give rise to this phenomenon.^9^ Because conformation populations are thermally sensitive and because achieving a conjugated conformer is critical for excitation, reaction temperature can have significant effects on the reaction rate.^13^ Indeed, we found that the rate slowed at temperatures both higher (entry 9) and lower (entry 10) than 0 °C. Presumably, this is because of competing effects. Higher temperatures decrease the lifetime of the reactive *trans*-cyclohexene, and consequently the probability of bimolecular reactions involving it.^14^ However, at too low of a temperature the non-conjugated and non-excitable conformer of the *cis*-cyclohexene becomes dominant and resists excitation.^13^ Finally, we looked at light intensity as a factor and found that indeed the rate could be dramatically increased by moving to a higher intensity light bath, suggesting that the reaction was photo-starved under these conditions. We began our investigation using a prototype design photoreactor from TechVen Systems. Eventually the prototype model was substituted for the Lumiére PR-W8 model (see ESI† for details), which provided increased number of LEDs, and overall power of the LEDs, while still allowing tight temperature control which we had seen was important. Indeed, we found that higher intensity LEDs led to approximately a 3-fold rate increase (entry 11), and led to an isolated yield of 95% of 2a (Table 2). We continued to use this photoreactor which provided both consistent high intensity light flux and tighter temperature control to explore the scope of this transformation. Later in our studies we tested another photoreactor model, Bellatrix PR-N2, (entry 12) which utilized 40 W LEDs and led to even greater reaction rates. At 5 minutes, the reaction had reached 63% conversion and was complete in less than 30 minutes. Again, these results suggest that the reaction was photon starved. While the photoreactors proved useful for studying the reaction, the reaction is expected to work with simpler light sources that can produce blue light.^10,12,13^ Having established reaction conditions, we utilized the Stork-Danheiser transposition^23^ to build substrates which allowed us to explore the scope of the reaction (Table 2). Starting with the substituent at the carbinol position, we found that the reaction tolerates electron-poor and electron-neutral arenes (2b and 2c), alkanes (2e), alkenes (2f), alkynes (2h and 2i), and even propargylamines (2g). However, more electron-rich arenes failed to give the acyclic product (2s) which is likely because of competing ionization of the labile alcohol.

**Table 1:** Optimization of reaction conditions^9,13^

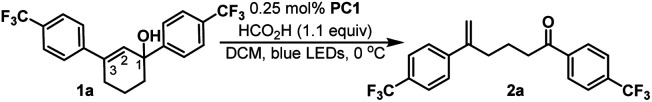			
Entry	Modifications of conditions	Conversion to 2a (%)	Reaction time (2h)
1	None	71, 100	3, 15
2	No acid	12	24
3	No light	0	24
4	No photocatalyst	0	24
5	PhCo_2_H instead HCo_2_H	77, 100	3, 15
6	PC2 instead of PC1	11, 24	3, 15
7	PC3 instead of PC1	41, 48	3, 15
8	PC4 instead of PC1	58, 100	3, 15
9	Increased from room temperature	74, 77	10, 32
10	Decreased temperature to −20 °C	58, 71	10, 32
11	Lumiere PR-W8 (5 W)	65, 100	1, 5
12	Bellatrix PR-N2 (40 W)	63, 100	0.08, 0.5
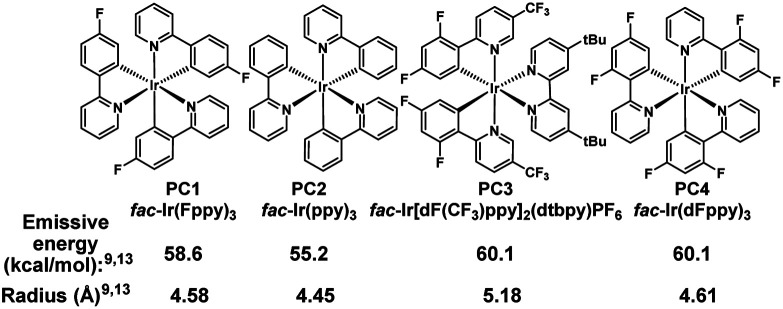			

**Table 2:** Scope of the photocatalytic ring opening osomerisation

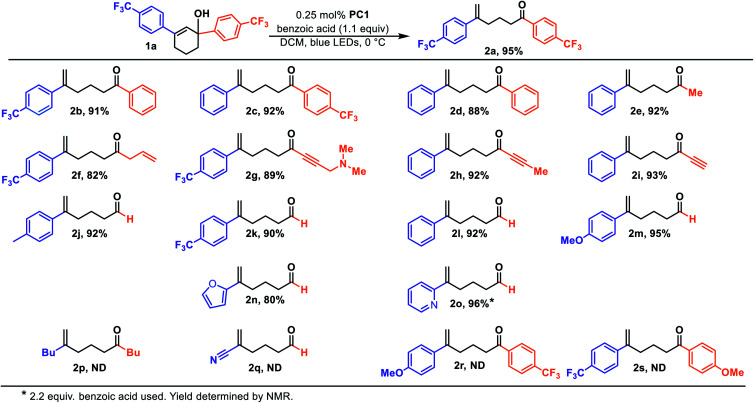

We next surveyed the vinyl substituent. As expected, conjugation with an aryl group was essential and alkyl substituted cyclohexenes failed (2p) most likely because its triplet state energy is too high to be excited using visible light and an Ir-based photosensitizer. However, through an alcohol migration process ring-opening is possible in some instances, *infra vide*. Nitriles (2q), while conjugated, also did not undergo ring opening. The reaction tolerates electron-poor and electron-neutral arenes (2b and 2c) but failed to give the acyclic product in the case when the arene was too electron-rich (2r). However, similar electron rich systems work if the carbinol is secondary, rather than tertiary, (2m). Indeed, the secondary carbinol with an electron rich furanyl group, (2n), was also well-behaved. One potential explanation for this observation is that, in the case of electron rich arenes, the carbenium ion is sufficiently stable such that other bimolecular processes outcompete the unimolecular fragmentation. In the case of secondary carbinols unimolecular fragmentation is sufficiently accelerated relative to the bimolecular processes. Interestingly, the arene scope also includes basic pyridines (2o), but successful reaction required an additional equivalent of acid. This result suggests that the active acid responsible for protonating the alkene was the benzoic acid and not the pyridinium.

**Scheme 2:**
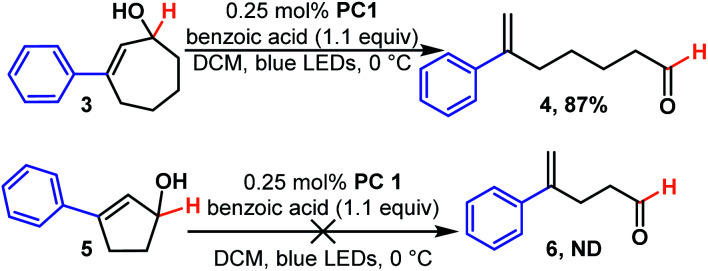
The effect of ring size on reactivity.

**Scheme 3:**
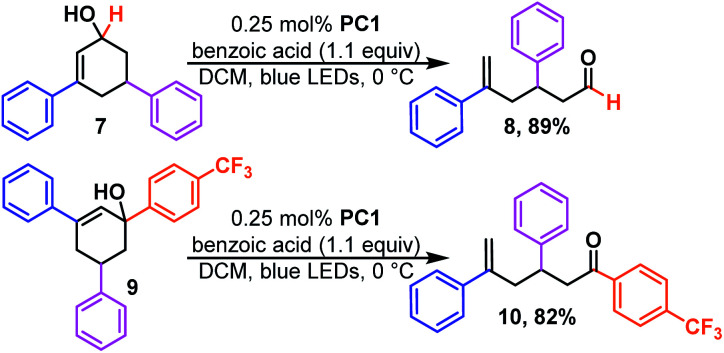
Densely substituted cyclohexenols.

We next examined the effect of ring size. We found cycloheptenols (3, Scheme 2) also readily undergo ring opening isomerization under the same conditions giving the even more remote enal (4) in excellent yield. However, smaller rings, such as cyclopentenols (5) do not yield any of the acyclic product (6). This is likely explained by the fact that the ring size is too small to form the ground state *trans*-cyclopentene.^30^ Given numerous methods to build six-membered rings, we were curious to learn how the reaction would tolerate more substituted cyclohexenols that might be more prone to form unproductive *trans*-cyclohexene diastereomers (Scheme 3). We found that di- and tri-substituted cyclohexenols readily undergo transformation (8 and 10) in good yields. The mild conditions of the reaction suggest that one could exploit the inherent diastereoselectivity of six-membered rings to build molecules asymmetrically, and then photocatalytically isomerize them to their corresponding asymmetric acyclic isomer in which asymmetric induction tends to be more challenging, if even possible.

**Scheme 4:**
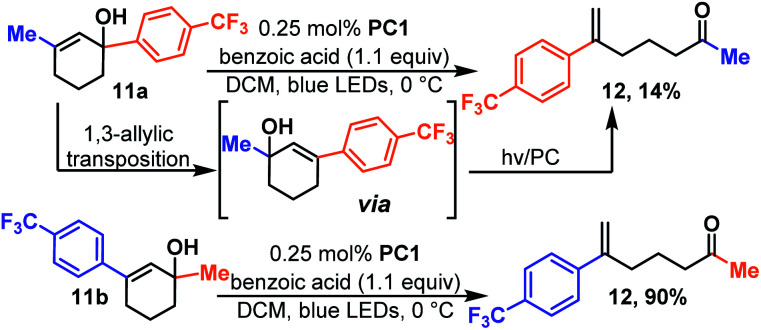
Tandem hydroxy transposition-ring opening isomerization.

As mentioned earlier, the alkene must be conjugated so that its triplet state is energetically accessible. Thus, cyclohexenols substituted with aliphatic groups do not typically undergo reaction. However, in the event that the alkene and alcohol group can migrate to the reactive positions, ring-opening is possible. For instance, 11a (Scheme 4) which is a methyl substituted cyclohexenol yields 12, albeit slowly and in low yield.

**Scheme 5:**
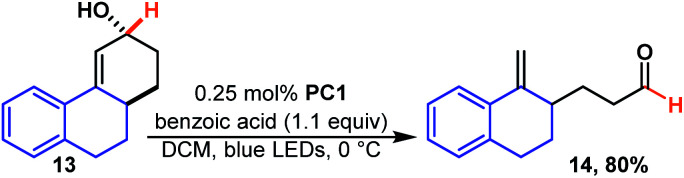
Ring opening of a polycyclic system.

This is the same product (12) that is formed when regio-isomeric 11b is isomerized which takes place quickly and in high yield. Presumably, in the case of 11a the rate determining step becomes hydroxy migration. We were pleased to see that the reaction takes place rapidly for polycyclic 13 (Scheme 5) a motif that contains three, of four, rings found in estradiol and related steroid derivatives. The reaction is quick, and unlike previous reactions, it was essential to carefully monitor the reaction and halt the reaction upon completion as the product (14) appeared to undergo further photochemistry under the reaction conditions. The additional photo-reactivity of 14 is likely because it is fused and this forces the product molecule to remain in an easily excited conformer.

The reaction is expected to take place *via* excitation of the ground state photocatalyst by a blue photon which undergoes inter-system crossing to generate a long-lived triplet excited state. This species undergoes Dexter energy transfer^31^ with the *cis*-cyclohexene to generate a triplet biradical, T1. On the excited state surface, the biradical undergoes rotation about the former double bond, and then through a conical intersection, re-enters the ground surface at or near the transition state for rotation about the double bond. Rapidly, it relaxes to either the *cis*- or *trans*-cyclohexene. The *trans*-isomer is expected to be ∼55 kcal mol^−1^ higher in energy than the *cis*-isomer.^14^ It is the release of this captured strain energy that drives the protonation of the alkene and ultimately the overall transformation. We calculated the transformation to be +2.6 kcal mol^−1^ endothermicc (ignoring the photonic energy). Once the carbenium ion is formed, unimolecular decomposition (*i.e.* the Grob–Eschenmoser fragmentation) takes place. Finally, proton transfer from the carbonyl to the carboxylate regenerates the acid. Unlike many recent examples of contrathermodynamic isomerization which are driven to their photostationary state by a loss in conjugation, this reaction is driven forward by coupling it to an irreversible secondary process.

**Scheme 6:**
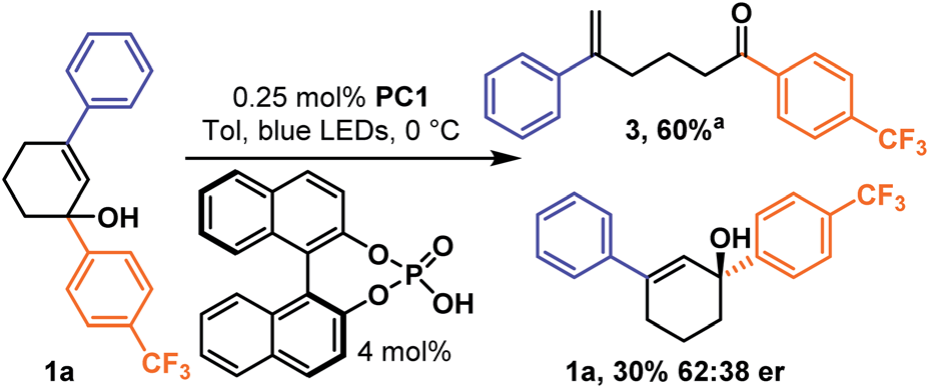
Ring opening kinetic resolution.

While a catalytic amount of acid proved competent, it slowed the reaction, so we continued to utilize a stoichiometric amount, given the low cost of benzoic acid. However, we became curious if a catalytic amount of chiral binol derived phosphoric acid could be utilized to couple the photocatalytic ring opening isomerization to an asymmetric discrimination step (Scheme 6). Simply replacing the stoichiometric benzoic acid with 4.0 mol% binol-derived phosphoric acid allowed a classic kinetic resolution to occur about the racemic carbinol stereocenter, giving a 62 : 38 er at 60% conversion. While the unoptimized result is modest, it does demonstrate that the transiently generated highly strained *trans*-cyclohexenes are susceptible to chiral induction. This result is particularly significant, as it accomplishes stereoselectivity while circumventing both thermodynamic and kinetic limitations-not typical of most reactions.^32^

## Conclusions

Herein, we have described how visible light can be utilized to reverse the normal polarity of the Grob–Eschenmoser fragmentation which provides a convenient method to synthesize acyclic distal hexenones and hexenals from readily accessible cyclic compounds. The method utilizes visible light to facilitate a triplet energy transfer process which in the case of cyclo-hexene and -heptene leads to highly strained *trans*-cycloalkenes. The captured energy can be utilized on the ground state surface to drive chemical processes. In this case, chemoselective protonation of an alkene rather than typically more basic alcohols. Critically, the mild conditions allow the alcohol to undergo unimolecular C–C fragmentation rather than elimination that would be expected under thermal conditions-even allowing enantiodescrimination by a chiral catalyst. These results serve as a demonstration of how photochemical energy can be harvested to drive chemical processes that would otherwise not be possible.

## Data availability

The datasets supporting this article have been uploaded as part of the supplementary material.

## Author contributions

Lantz (data curation, formal analysis, investigation, methodology, resources, writing – original draft), El Mokadem (investigation, resources), Schoch (investigation, resources), Fleske (investigation, resources), Weaver (conceptualization, formal analysis, funding acquisition, project administration, resources, supervision, writing – review & editing).

## Conflicts of interest

There are no conflicts to declare.

## Supplementary Material

SC-013-D1SC03774A-s001
