# Supplementary material for: A new twist for Stork-Danheiser products enabled by visible light mediated trans-cyclohexene formation; access to acyclic distal enones
Source: Chem Sci. 2022 Jul 22;13(32):9271–6. doi: 10.1039/d1sc03774a (PMC9384155; doi:10.1039/d1sc03774a)
Supplement: SC-013-D1SC03774A-s001 [file SC-013-D1SC03774A-s001.pdf]

Electronic Supplementary Material (ESI) for Chemical Science.
This journal is © The Royal Society of Chemistry 2022

A New Twist in the Stork-Danheiser Reaction Enabled by Visible Light Mediated *Trans*-Cyclohexene Formation; Access to Acyclic Distal Enones

Erik Lantz, Roukaya El Mokadem, Tim Schoch, and Tyler Fleske, and Jimmie D. Weaver III*

Department of Chemistry, Oklahoma State University, Stillwater, OK 74078 USA

jimmie.weaver@okstate.edu

Electronic Supplementary Information

Table of Contents

I.	General Information	E2
II.	Synthesis of Substrates	E3
III.	List of Substrates	E13
IV.	General Procedure for Photocatalytic Reactions	E23
V.	List of Reaction Products	E24
VI.	Calculations	E31
VII.	NMR and GCMS Spectra	E39
VIII.	References	E183

I. General Information

Reagents were purchased from commercial suppliers including Sigma, TCI, and Oakwood chemical; they were used without further purification. ACS grade solvents were purchased from Fisher. Tetrahydrofuran (THF) used in synthesis of substrates was dried refluxing over sodium metal in a still and distillation from benzophenone ketyl indicator. Where necessary, methanol and ethanol were dried over 3Å molecular sieves.

Photocatalysts were prepared by our previously reported method.[1] Light-promoted reactions were performed with a TechVen Systems Lumière PR-W8 photoreactor. It is a stand-alone unit with eight individually controlled reaction stations. The unit provides up to 5 watts (at 1 W per LED) centered at 447 nm. The LEDs are stacked vertically on each module. Each reaction station is composed of borosilicate glass, which puts reaction vessels (NMR tubes) an approximate 1.5 cm distance from the light source. The reactor is cooled to 0 °C with an external chiller unit (using propylene glycol/water as the coolant). A Bellatrix PR-N2 photoreactor, the latest model from TechVen Systems, was also used in a trial run to test reaction conversion and effect of higher optical power (40 W). For more information on the photoreactors used in these experiments, visit www.techvensystems.com.

Reaction progressions were monitored by NMR, utilizing C_6D_6 capillaries or deuterated solvent (where necessary). Reactions were also monitored by thin-layer chromatography (TLC), on silica XHL TLC plates (UV254, glass-backed, 250 µm) from Sorbent Technologies, Inc. Synthesized compounds were purified by flash chromatography on a Teledyne ISCO Combiflash Rf, using refillable Redisep columns. The silica used was 60Å technical grade (40-63 µm) supplied by Sorbtech. Detectors were set to 254 and 280 nm; for compounds without a readily detectable chromophore, the evaporative light scattering detector (ELSD) was utilized.

NMR spectra were obtained using a 400 MHz Bruker Avance III. GCMS traces were obtained by a Shimadzu GCMS-QP2010 SE. High resolution masses were obtained by a ThermoScientific Orbitrap Fusion, operated in Orbitrap-FTMS mode (with a nominal resolution of 120,000). Melting points were obtained on a Stuart SMP10 and reported uncorrected.

II. Synthesis of Substrates

General Procedure A1 for Intermediate A Synthesis

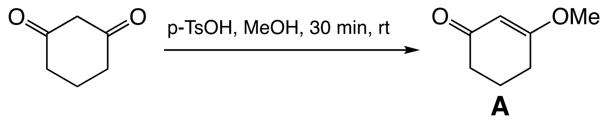

Cyclohexanedione (5.00 g, 44.6 mmol), methanol (100 mL), and a catalytic amount of p-toluenesulfonic acid (0.42 g, 2.2 mmol) were added to a round-bottom flask, equipped with a magnetic stir bar. The reaction was allowed to stir for 30 min at room temperature. The methanol was removed in vacuo. The resulting mixture was then dissolved into ethyl acetate (100 mL) and quenched by addition of saturated sodium bicarbonate solution (50 mL). Extraction was performed with ethyl acetate (3 x 50 mL). The combined organic layers were washed with deionized (DI) water (50 mL), followed by saturated sodium chloride solution (50 mL). After separating the organic layer, it was dried over magnesium sulfate ($MgSO_4$), filtered and then concentrated. The crude material was purified by flash chromatography, using hexane : ethyl acetate as the eluent, the product eluted at 25% EtOAc. The reaction yielded 81% (4.56 g, 36.1 mmol) of 3-methoxycyclohex-2-en-1-one. The material was subsequently recrystallized in cyclohexane and was retained for seeding future batches.

Afterwards, the above procedure was used except that chromatographic purification could be replaced with a crystallization, albeit at reduced yield. After workup the compound could be directly isolated via crystallization from a minimal amount of hot cyclohexane using previously purified compound to seed the crystallization to afford an average yield of 48% (2.70 g, 21.4 mmol) over 8 runs.

General Procedure B1 for Synthesis of Cyclohexenone B

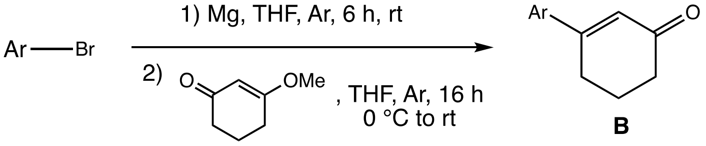

A magnetic stir bar and magnesium turnings (0.18 g, 7.5 mmol) were added to a dried two-neck round-bottom flask; following with a pinch of iodine and dried THF (0.5 M). An argon atmosphere was maintained in the flask due to the oxygen and moisture sensitivity of Grignard reactions. The mixture was allowed to stir for 20 min; then 4-bromobenzotrifluoride (1.1 mL, 7.5 mmol) was added drop-wise. After consumption of the bromide, the reaction mixture was titrated using dry THF and iodine to determine the amount of Grignard reagent formed. The reaction mixture was then cooled in an ice bath for 20 min. Methoxycyclohexenone **A** (0.77 g, 6.1 mmol, 1 equivalent with respect to the titrated amount of Grignard formed), dissolved in dried THF (1 M), was slowly added drop-wise. The ice bath was removed and the reaction mixture was allowed to warm to room temperature. The reaction was stirred until reaction completion was reached (16-24 h) as indicated by the consumption of the methoxycyclohexenone by TLC. The reaction mixture was quenched with saturated ammonium chloride solution (50 mL), then extracted with ethyl acetate (3 x 50 mL). Combined organic layers were washed with saturated sodium bicarbonate solution (50 mL), DI water (50 mL), then saturated sodium chloride solution (50 mL). The separated organic layer was then dried over $MgSO_4$, filtered, and then concentrated in vacuo. The product mixture was purified via flash chromatography (hexane : ethyl acetate). The reaction yielded 48% (0.70 g, 2.9 mmol) of 4'-(trifluoromethyl)-5,6-dihydro-[1,1'-biphenyl]-3(4H)-one.

Alternative methods to preparation of enone **B** can be accomplished by purchasing pre-prepared Grignard reagents, or organolithium reagents. In this case, the reagents can be directly added to a cooled solution of methoxycyclohexenone in dry solvent (see procedure **D3**).

General Procedure C1 for Synthesis of Secondary Allylic Alcohol C

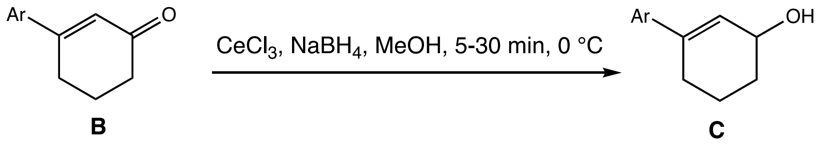

Enone **B** was reduced to the secondary allylic alcohol **C** via Luche reduction. A dried round-bottom flask, equipped with a magnetic stir bar, was added to an ice bath. 3-[4-(trifluoromethyl)phenyl]cyclohex-2-en-1-one (Enone **B)** (0.50 g, 2.1 mmol, 1 equiv), methanol (0.2 M), and a catalytic amount of cerium trichloride heptahydrate (0.08 g, 0.2 mmol) were added to the flask, and allowed to stir for 15 min. Sodium borohydride (0.12 g, 3.2 mmol, 1.5 equiv) was

then added portion-wise to the reaction mixture. Reaction progress was monitored by TLC, until enone **B** was completely consumed. The reaction was quenched by the slow addition of a minimal amount of water. The methanol was removed in vacuo, and the crude mixture was extracted with ethyl acetate (20 mL). The organic layer was dried over $MgSO_4$, filtered, and concentrated. The crude alcohol was purified via flash chromatography (hexane : ethyl acetate) with the product eluting at 10% EtOAc. The yield of the reaction was 63% (0.32 g, 1.3 mmol) of 4'-(trifluoromethyl)-3,4,5,6-tetrahydro-[1,1'-biphenyl]-3-ol.

For substrate **5**, the same procedures were used as above; however, the intermediate was formed with 1,3-cyclopentanedione.

Table 1. Summary of various secondary alcohol substrates and their yields

Substrate ID	Identity of Ar—Br	Yield (%) [over three steps]
**1j**	1-bromo-4-methylbenzene	44
**1k**	1-bromo-4-(trifluoromethyl)benzene	37
**1l**	bromobenzene	45
**1m**	1-bromo-4-methoxybenzene	30
**5**	bromobenzene	33

General Procedure D1 for Synthesis of Tertiary Allylic Alcohol D

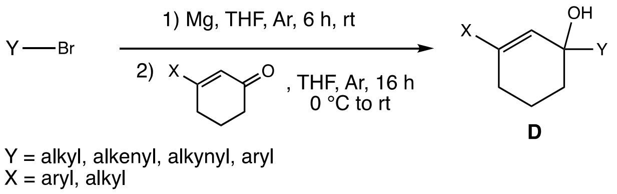

Tertiary allylic alcohols were then synthesized via a Grignard addition to enone **B**. To a dried round-bottom flask with a magnetic stir bar, magnesium turnings (0.19 g, 7.9 mmol), a pinch of iodine, and dried THF (0.5 M) were added. The mix was allowed to stir for 20 min before adding 4-bromobenzotrifluoride (1.11 mL, 7.9 mmol). After visible consumption of the magnesium turnings, an aliquot of the reaction mixture was removed and titrated with a solution of iodine in

dry THF. With the Grignard concentration known, the reaction mixture was cooled in an ice bath. Meanwhile, 3-[4-(trifluoromethyl)phenyl]cyclohex-2-en-1-one (1.39 g, 5.8 mmol, 1 equivalent with respect to amount of Grignard reagent titrated) was dissolved in dried THF to 1 M (5.8 mL). Once the reaction mixture had cooled, the enone solution was added dropwise. After addition, the ice bath was removed and the reaction allowed to warm to room temperature. Reaction progress was monitored via TLC. Upon reaction completion, the reaction was quenched with saturated ammonium chloride solution (50 mL), then extracted with ethyl acetate (3 x 50 mL). The combined organic layers were washed with saturated sodium bicarbonate solution (50 mL), DI water (50 mL), then saturated sodium chloride solution (50 mL). The organic layer was then dried over $MgSO_4$, filtered, and concentrated in vacuo. The crude material was purified via flash chromatography using 1% triethylamine buffered hexane : ethyl acetate. The product eluted at 5% EtOAc. The yield was 48% (1.08 g, 2.8 mmol) of 1,3-di[4-(trifluoromethyl)phenyl]cyclohex-2-en-1-ol. Note, base buffering was essential to prevent decomposition of the product on the column.

Table 2. Summary of various tertiary alcohol substrates with yields (by procedures **B1 and D1**)

Substrate ID	Identity of X	Identity of Y—Br	Yield (%) [over two steps]
**1a**	(4'-trifluoromethyl)phenyl	1-bromo-4-(trifluoromethyl)benzene	48
**1b**	(4'-trifluoromethyl)phenyl	bromobenzene	52
**1c**	phenyl	1-bromo-4-(trifluoromethyl)benzene	54
**1d**	phenyl	bromobenzene	54
**1f**	(4'-trifluoromethyl)phenyl	allyl bromide	39
**1r**	(4'-methoxy)phenyl	1-bromo-4-(trifluoromethyl)benzene	59
**1s**	(4'-trifluoromethyl)phenyl	1-bromo-4-methoxybenzene	32
**11a**	methyl	1-bromo-4-(trifluoromethyl)benzene	38

General Procedure D2 for Synthesis of Tertiary Allylic Alcohol D

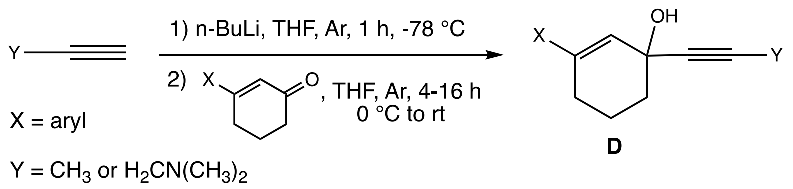

A magnetic stir bar was added to a dried round-bottom flask, followed by propyne (3.8 mL of a 1 M solution in THF, 3.8 mmol) and 7.6 mL dried THF (to 0.5 M). The resulting solution was cooled in a dry ice/acetone bath; then n-butyllithium (1.4 mL of a 2.5 M solution in hexanes, 3.5 mmol) was slowly added drop-wise. The reaction was allowed to stir for 1 h; afterwards, 3-phenylcyclohex-2-en-1-one (0.6 g, 3.5 mmol) was dissolved in 3.5 mL of dry THF (to 1 M), then was slowly added dropwise. Upon completion of addition, the ice bath was removed and the reaction mixture was allowed to warm to room temperature. The reaction progress was monitored by TLC by the consumption of the enone. Once the enone was consumed, the reaction mixture was quenched with saturated ammonium chloride solution (50 mL), then extracted with ethyl acetate (3 x 50 mL). Combined organic layers were washed with saturated sodium bicarbonate solution (50 mL), DI water (50 mL), then saturated sodium chloride solution (50 mL). The separated organic layer was then dried over $MgSO_4$, filtered, and concentrated in vacuo. The product mixture was purified via flash chromatography using 1% triethylamine buffered DCM : MeOH. The product eluted at 1% MeOH. The reaction yielded 72% (0.53 g, 2.5 mmol) of 3-(prop-1-yn-1-yl)-3,4,5,6-tetrahydro-[1,1'-biphenyl]-3-ol . Note, base buffering was essential to prevent decomposition on the column.

Table 3. Summary of various tertiary alcohol substrates and their yields (by procedure **D2**)

Substrate ID	Identity of X	Identity of Y	Yield (%) [over two steps]
**1g**	(4’-trifluoromethyl)phenyl	$CH_2N(Me)_2$	76
**1h**	Phenyl	Me	72

General Procedure D3 for Synthesis of Tertiary Allylic Alcohol D

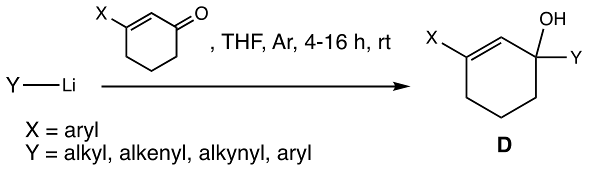

As with synthesis of the enone **B**, prepared organolithium reagents can be used as an alternative method to synthesize **D**. With use of purchased reagents, substrates were synthesized

in one step. A magnetic stir bar was added to a dried round-bottom flask, followed by 3-phenylcyclohex-2-en-1-one (1.0 g, 5.8 mmol) and 11.6 mL dried THF (to 0.5 M). The resulting solution was cooled in an ice bath; then methyllithium (3.4 mL of a 1.9 M solution in hexane, 6.5 mmol) was added drop-wise. Upon completion of addition, the ice bath was removed, and the reaction mixture was allowed to warm to room temperature. The reaction was monitored by TLC for consumption of the enone. The substrate was isolated as above in procedure **D2**. The reaction yielded 47% (0.51 g, 2.7 mmol) of 1-methyl-3-phenylcyclohex-2-en-1-ol.

Table 4. Summary of various tertiary alcohol substrates and their yields (by procedure **D3**)

Substrate ID	Identity of X	Identity of Y	Yield (%)
**1e**	phenyl	Me	47
**1i**	(4'-trifluoromethyl)phenyl	acetylide (ethylenediamine complex)	55
**1p**	butyl	n-butyl	49
**11b**	(4'-trifluoromethyl)phenyl	Me	38

Procedure P1 for Synthesis of Substrate 1o: 3-hydroxycyclohex-1-ene-1-carbonitrile

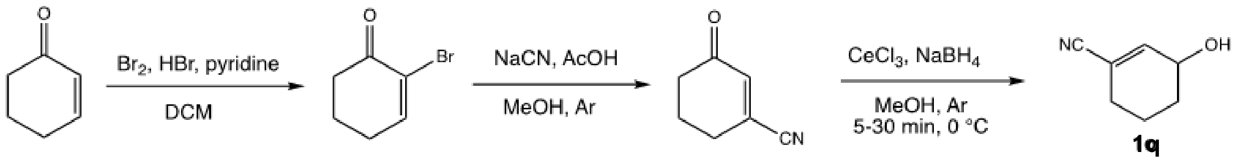

The synthesis of 3-oxocyclohex-1-ene-1-carbonitrile (**1q**) was utilized unmodified from a previously reported method.[2] The third step of the synthesis is Luche reduction of 3-oxocyclohex-1-ene-1-carbonitrile. This step is not part of the previously cited protocol, this was performed as in procedure C1. The yield of **1q** from this step was 46% (2.1 g, 17.4 mmol) of 3-hydroxycyclohex-1-ene-1-carbonitrile.

Note: hydrogen cyanide is generated during this procedure – it is critical that this reaction is performed in a fully functioning fume hood and that the reaction mixtures be neutralized before removal.

Procedure P2 for Synthesis of Substrate 3: 3-phenylcyclohept-2-en-1-ol

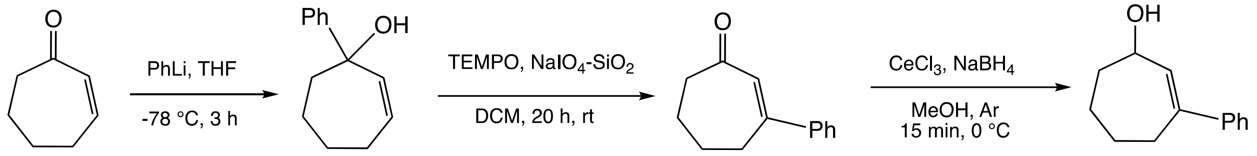

3

The synthesis of 3-phenylcyclohept-2-en-1-ol consists of three steps. The first two steps are to prepare 3-phenylcyclohept-2-en-1-one.[3] To a dried round-bottom flask, equipped with a magnetic stir bar, cycloheptenone (1.0 g, 9.1 mmol) was added to 30 mL of dry THF (0.3 M). The flask was placed in a dry ice/acetone bath. Phenyllithium (7.6 mL of a 1.8 M solution in butyl ether) was then added drop-wise, and allowed to stir for 3 h. The reaction was quenched with saturated ammonium chloride solution (30 mL) and extracted with ethyl acetate (3 x 10 mL). The separated organic layer was dried over $MgSO_4$, filtered, and concentrated. The crude product was purified by column chromatography (hexane : ethyl acetate) which eluted at 5% EtOAc to afford the desired product in 60% yield (1.0 g, 5.5 mmol).

The resulting tertiary allylic alcohol (0.75 g, 4 mmol) was dissolved in 30.8 mL of DCM (0.13 M) and added to a round-bottom flask with magnetic stir bar. TEMPO (1 mol%) was then added, followed by $NaIO_4$-SiO_2 (7.0 g). The silica supported periodate was made according to a reported procedure without modification.[6] The reaction mixture was stirred at room temperature for 20 h. The reaction mixture was then filtered, and the solid was washed with DCM. The resulting filtrate was concentrated and purified via column chromatography (hexane : ethyl acetate) with the product eluting at 4% EtOAc to afford a 41% yield (0.31 g, 1.6 mmol) of 3-phenylcyclohept-2-en-1-one.

The third step consists of a Luche reduction, performed as previously stated in procedure **C1**. The reaction was performed using 1.1 mmol of 3-phenylcyclohept-2-en-1-one (0.21 g), which gave a yield of 89% (0.19 g, 1.0 mmol) of 3-phenylcyclohept-2-en-1-ol.

Procedure P3 for Synthesis of Substrate 7: 1',2',5',6'-tetrahydro-[1,1':3',1''-terphenyl]-5'-ol

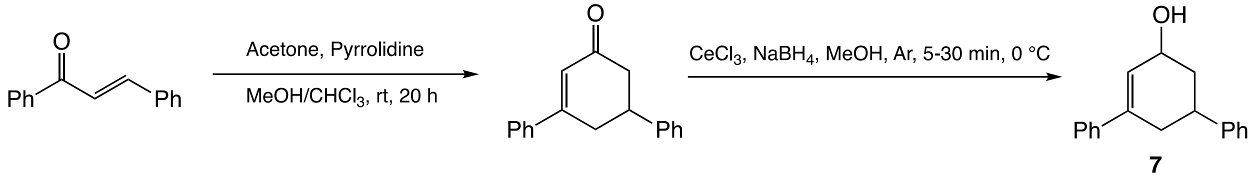

The two-step process to synthesize substrate **7** was followed as previously reported.[4] A 1:1 mixture of methanol and chloroform (50 mL) was added to a round-bottom flask, followed by a magnetic stir bar. Chalcone (4.2 g, 20 mmol) was added and stirred at room temperature until dissolved. Acetone (18 mL) and pyrrolidine (0.33 mL, 4 mmol) were then added. The reaction mixture was stirred at room temperature for 20 h. The solvent was removed from the reaction mixture, and the crude cyclohexenone was purified by column chromatography (hexane : ethyl acetate, product eluted at 5% ethyl acetate) to yield 44% (2.2 g, 8.9 mmol) of 3,5-diphenylcyclohex-2-en-1-one.

The second step is a Luche reduction, performed as previously stated in procedure **C1**. The reaction was performed using 1.0 g of 3,5-diphenylcyclohex-2-en-1-one (4.0 mmol). The yield from this step was 77% (0.78 g, 3.1 mmol) of 3,5-diphenylcyclohex-2-en-1-ol (**7**).

Procedure P4 for Synthesis of Substrate 9: 5'-phenyl-4-(trifluoromethyl)-3',4'-dihydro-[1,1':3',1''-terphenyl]-1'(2'H)-ol

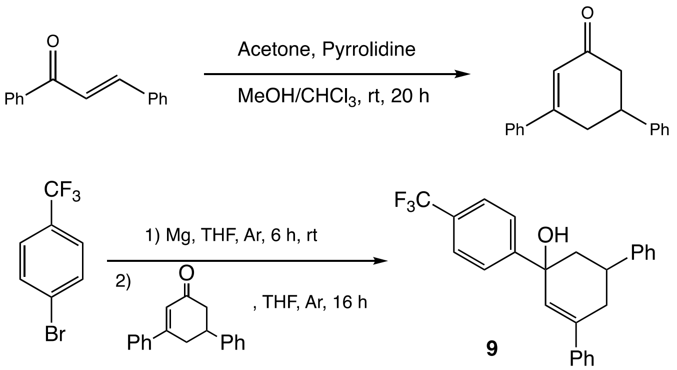

The three-step synthesis for Substrate **9** begins as above, in procedure **P3**, with the synthesis of 3,5-diphenylcyclohex-2-ene-1-one. A Grignard reagent is prepared and the enone is added, following procedure **D1**. The reaction was performed using 1.0 g of 3,5-diphenylcyclohex-

2-en-1-one (4.0 mmol). The final step yielded 38% (0.59 g, 1.5 mmol) of 3,5-diphenyl-1-[4-(trifluoromethyl)phenyl]cyclohex-2-en-1-ol (**9**).

Procedure P5 for Synthesis of Substrate 13: (3R, 10a*R*)-1,2,3,9,10,10a-hexahydrophenanthren-3-ol

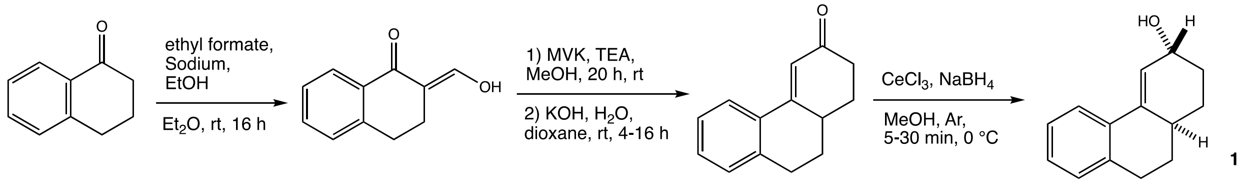

13

The preparation of **13** is a three-step process which begins with the synthesis of 1,9,10,10a-tetrahydrophenanthren-3(2H)-one, this synthesis is modified from a reported method.[5] The formation of the enone begins with alpha-formylation of alpha-tetralone. Anhydrous diethyl ether (12.6 mL, to 1 M) was added to a round-bottom flask with a magnetic stir bar, followed by addition of tetralone (2.9 mL, 12.6 mmol) under an atmosphere of argon gas. The mixture was then cooled in an ice bath. Six thin pieces of sodium metal at approximately 0.5 cm^2 (approximately 0.5 g) were added to the flask. Ethyl formate (1.5 mL, 19 mmol) and dry ethanol (0.15 mL) were also added, then stirred for 30 min before removing the ice bath. The reaction was allowed to stir for 16 h at room temperature. The flask was then cooled in an ice bath. The reaction was quenched by slow addition of cold water and then stirred for an additional 30 min at 0 °C. The aqueous phase was removed and acidified with concentrated hydrochloric acid solution. The resulting acidic aqueous solution was extracted with ether, then ether layer was washed with saturated sodium bicarbonate solution, dried with $MgSO_4$, filtered, and concentrated in vacuo. The crude compound was then purified via flash chromatography (hexane) to achieve a yield of 88% (1.93 g, 11.1 mmol) of 2-(hydroxymethylene)-3,4-dihydronapthalen-1(2*H*)-one.

The target enol (**13**) was then formed by Robinson annulation of the previously formed vinyl alcohol and methyl vinyl ketone. 2-(hydroxymethylene)-3,4-dihydronapthalen-1(2*H*)-one (1.7 g, 10 mmol) was added to a round-bottom flask, along with a magnetic stir bar and 40 mL of dried methanol (0.25 M). The solution was then cooled in an ice bath before slowly adding triethylamine (2.8 mL, 20 mmol) drop-wise, and subsequently adding methyl vinyl ketone (1.0 mL, 12 mmol). The ice bath was then removed and the reaction mixture was allowed to warm to

room temperature and stirred for 20 h. After 20 h, the reaction mixture was then neutralized with glacial acetic acid. The methanol was removed in vacuo, then replaced with dioxane (0.1 M). A solution of 8.7% (mass/vol) KOH in water was prepared by dissolving 1.80 g of KOH into 20.7 mL of water; this solution was then added to the flask. The reaction mixture was stirred vigorously for 3.5 h at room temperature. The reaction mixture was then washed with water, followed by saturated sodium chloride solution. The aqueous layer was then extracted with DCM. The combined organic layers were dried over $MgSO_4$, filtered, concentrated, and purified by column chromatography (hexane : DCM to 30% DCM, then hexane : ethyl acetate with a slow increase of EtOAc to 3% and holding until product is eluted). A yield of 79% (1.6 mg, 7.9 mmol) of 1,9,10,10a-tetrahydrophenanthren-3(2*H*)-one was obtained.

The isolated enone was then reduced as in procedure **C1**. Substrate **13** was subsequently isolated via flash chromatography using 1% triethylamine buffered hexane : ethyl acetate. The product eluted at 13.5% EtOAc with a yield of 67% (0.20 g, 1.0 mmol) of (3*R*,10a*R*)-1,2,3,9,10,10a-hexahydrophenanthren-3-ol. The diastereomer was confirmed via 2D NOE experiments.

III. List of Substrates

1a 4,4''-bis(trifluoromethyl)-5',6'-dihydro-[1,1':3',1''-terphenyl]-1'(4'H)-ol

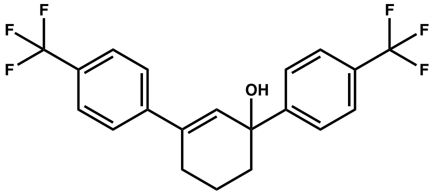

General procedures **B1** and **C1** were followed using 1-bromo-4-(trifluoromethyl)benzene. The substrate was isolated as a white solid with a yield of 60% (2.905 g, 7.5 mmol). ^{1}H NMR (400 MHz, $CDCl_3$) δ 7.65 – 7.53 (m, 8H), 6.17 (s, 1H), 2.66 – 2.47 (m, 2H), 2.13 – 1.80 (m, 4H). ^{19}F NMR (376 MHz, $CDCl_3$) δ -62.4, -62.5. ^{13}C NMR (101 MHz, Methylene Chloride-d2) δ 152.6 (q, *J* = 1.4 Hz), 145.4 (q, *J* = 1.4 Hz), 140.6, 130.6, 130.0 (q, *J* = 32.4 Hz), 129.5 (q, *J* = 32.1 Hz), 126.6, 126.5, 125.9 (q, *J* = 3.8 Hz), 125.6 (q, *J* = 3.9 Hz), 125.0 (q, *J* = 271.8 Hz), 124.9 (q, *J* = 271.8 Hz), 73.1, 39.7, 28.0, 20.0. GC/MS (m/z, relative intensity) M+ (386.2, 95) and M+ minus H_2O (368.2, 100). Melting point 141-143 °C.

1b 4''-(trifluoromethyl)-5',6'-dihydro-[1,1':3',1''-terphenyl]-1'(4'H)-ol

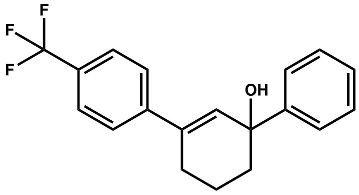

General procedure **B1** was followed using 1-bromo-4-(trifluoromethyl)benzene. Subsequently, general procedure **D1** was followed using bromobenzene. The substrate was isolated as a colorless, viscous oil with a yield of 42% (0.53 g, 1.7 mmol). ^{1}H NMR (400 MHz, CD_2Cl_2) δ 7.66 – 7.57 (m, 4H), 7.52 – 7.48 (m, 2H), 7.38 – 7.32 (m, 2H), 7.29 – 7.24 (m, 1H), 6.21 (t, *J* = 1.9 Hz, 1H), 2.62 – 2.45 (m, 2H), 2.12 (d, *J* = 1.3 Hz, 1H), 2.06 – 1.72 (m, 4H). ^{19}F NMR (376 MHz, CD_2Cl_2) δ -62.8. ^{13}C NMR (101 MHz, CD_2Cl_2) δ 148.5, 145.7, 139.6, 131.6, 129.7 (q, *J* = 32.4 Hz), 128.7, 127.5, 126.5, 126.0, 125.8 (q, *J* = 3.9 Hz), 124.9 (q, *J* = 271.6 Hz), 73.1, 39.7, 28.0, 20.2. GC/MS M+ (318.2, 100) and M+ -H2O (300.1, 80). HRMS could not be obtained on this compound. Elemental analysis was not performed. The product of this substrate is an isomer (M+ of the isomer was detected, see **2b**).

1c 4-(trifluoromethyl)-5',6'-dihydro-[1,1':3',1''-terphenyl]-1'(4'H)-ol

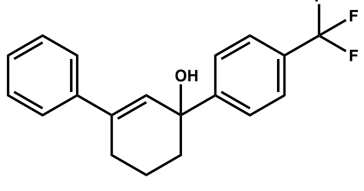

General procedure **B1** was followed using bromobenzene. Subsequently, general procedure **D1** was followed using 1-bromo-4-(trifluoromethyl)benzene. The substrate was isolated as a white solid with a yield of 54% (1.645 g, 5.2 mmol). ^{1}H NMR (400 MHz,

CD_2Cl_2) δ 7.73 – 7.56 (m, 4H), 7.48 (d, *J* = 7.2 Hz, 2H), 7.33 (m, 3H), 6.10 (s, 1H), 2.56 (m, 2H), 2.19 (s, 1H), 2.08 – 1.77 (m, 4H). ^{19}F NMR (376 MHz, CD_2Cl_2) δ -62.6 ^{13}C NMR (101 MHz, CD_2Cl_2) δ 153.1, 141.7, 129.3 (q, *J* = 32.1 Hz), 129.0, 128.6, 128.4, 126.7, 126.1, 125.5 (q, *J* = 3.7 Hz), 125.0 (q, *J* = 271.9 Hz), 73.1, 39.6, 28.1, 20.1. GC/MS (m/z, relative intensity) M+ (318.1, 100) and M+ -H_2O (300.2, 100). Melting point 145-147 °C.

1d 5',6'-dihydro-[1,1':3',1''-terphenyl]-1'(4'H)-ol

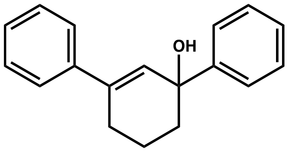

General procedures **B1** and **C1** were followed using bromobenzene. The substrate was isolated as a colorless oil with a yield of 46% (0.40 g, 1.6 mmol). ^{1}H NMR (400 MHz, $CDCl_3$) δ 7.59 – 7.48 (m, 4H), 7.43 – 7.34 (m, 4H), 7.35 – 7.26 (m, 2H), 6.19 (s, 1H), 2.67 – 2.48 (m, 2H), 2.14 – 1.91 (m, 4H), 1.91 – 1.78 (m, 1H). ^{13}C NMR (101 MHz, $CDCl_3$) δ 148.2, 141.4, 140.4, 129.0, 128.5, 128.3, 127.8, 127.1, 125.7, 73.0, 39.4, 27.7, 19.9. Note: vinyl carbon signal is not apparent. GC/MS (m/z, relative intensity) M+ (318.1, 100) and M+ -H_2O (300.2, 100). HRMS could not be obtained on this compound. Elemental analysis was not performed. The product of this substrate is an isomer (M+ of the isomer was detected, see **2d**).

1e 3-methyl-3,4,5,6-tetrahydro-[1,1'-biphenyl]-3-ol

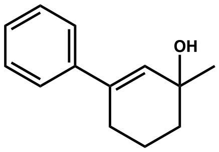

General procedure **B1** was followed using bromobenzene, subsequently procedure **D3** was followed with methyllithium. The substrate was isolated as a white solid with a yield of 47% (0.26 g, 1.4 mmol). ^{1}H NMR (400 MHz, CD_2Cl_2) δ 7.45 – 7.37 (m, 2H), 7.37 – 7.28 (m, 2H), 7.28 – 7.22 (m, 1H), 5.97 (t, *J* = 1.7 Hz, 1H), 2.53 – 2.27 (m, 2H), 1.94 – 1.58 (m, 5H), 1.35 (s, 3H). ^{13}C NMR (101 MHz, CD_2Cl_2) δ 142.2, 134.0, 131.2, 128.8, 127.8, 125.9, 68.9, 38.1, 30.0, 28.1, 20.6. GC/MS M+ -H_2O (170.2, 100). HRMS could not be obtained on this compound. Elemental analysis was not performed, since **1e** is isomeric with the ring-opened product and the M+ of the ring-opened isomer was detected (see **2e**). Melting point 41-44 °C.

1f 3-allyl-4'-(trifluoromethyl)-3,4,5,6-tetrahydro-[1,1'-biphenyl]-3-ol

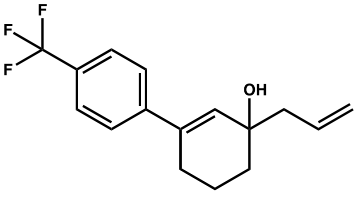

General procedure **B1** was followed using 1-bromo-4-(trifluoromethyl)benzene. Subsequently, procedure **D1** was followed using allyl bromide. The product was isolated as a colorless oil with a yield of 47% (0.28 g, 1.0 mmol). ^{1}H NMR (400 MHz, $CDCl_3$) δ 7.57 (d, J = 8.3 Hz, 2H), 7.48 (d, J = 8.2 Hz, 2H), 6.04 (s, 1H), 5.92 (ddt, J = 16.8, 10.4, 7.3 Hz, 1H), 5.23 – 5.12 (m, 2H), 2.51 – 2.30 (m, 4H), 1.97 – 1.67 (m, 5H). ^{19}F NMR (376 MHz, $CDCl_3$) δ -62.5. ^{13}C NMR (101 MHz, $CDCl_3$) δ 145.1 (q, J = 1.4 Hz), 138.8, 133.3, 130.9, 129.4 (q, J = 32.4 Hz), 125.8, 125.3 (q, J = 3.8 Hz), 124.2 (q, J = 271.9 Hz), 119.1, 69.8, 46.9, 35.1, 27.7, 19.4. GC/MS M+ -H_2O (264.2, 100). HRMS could not be obtained on this compound. Elemental analysis was not performed. The product of this substrate is an isomer (M+ of the isomer was detected, see **2f**).

1g 3-(3-(dimethylamino)prop-1-yn-1-yl)-4'-(trifluoromethyl)-3,4,5,6-tetrahydro-[1,1'-biphenyl]-3-ol

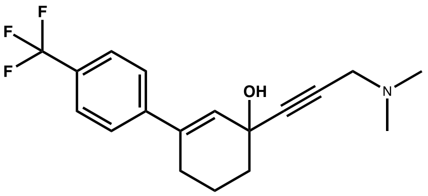

General procedure **B1** was followed using 1-bromo-4-(trifluoromethyl)benzene. Subsequently, procedure **D2** was followed using *N,N*-dimethylprop-2-yn-1-amine. The product was isolated as an off-white solid with a yield of 61% (0.41 g, 1.3 mmol). ^{1}H NMR (400 MHz, CD_2Cl_2) δ 7.62 – 7.49 (m, 4H), 6.16 (t, J = 1.8 Hz, 1H), 3.56 (b, 1H), 3.25 (s, 2H), 2.50 – 2.33 (m, 2H), 2.25 (s, 6H), 2.16 – 1.84 (m, 4H). ^{19}F NMR (376 MHz, CD_2Cl_2) δ -62.8. ^{13}C NMR (101 MHz, CD_2Cl_2) δ 144.8 (d, J = 1.4 Hz), 137.3, 129.7, 129.0 (q, J = 32.3 Hz), 125.8, 125.2 (q, J = 3.8 Hz), 124.3 (q, J = 271.7 Hz), 88.8, 79.1, 65.4, 47.9, 43.9, 37.8, 27.1, 19.7. GC/MS (m/z, relative intensity) reported M+ (323.2, 60). Melting point 128-130 °C.

1h 3-(prop-1-yn-1-yl)-3,4,5,6-tetrahydro-[1,1'-biphenyl]-3-ol

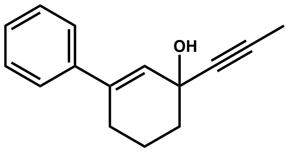

General procedure **B1** was followed using bromobenzene. Subsequently, procedure **D2** was followed using propyne. The product was isolated as a white solid with a yield of 72% (0.42 g, 2.0 mmol). ^{1}H NMR (400 MHz, CD_2Cl_2) δ 7.44 – 7.39 (m, 2H), 7.37 – 7.30 (m, 2H), 7.30 – 7.24 (m, 1H), 6.04 (t, J = 1.6 Hz, 1H), 2.51 – 2.33 (m, 2H), 2.09 (s, 1H), 2.06 – 1.87 (m, 4H), 1.85 (s, 3H). ^{13}C NMR (101 MHz, CD_2Cl_2)

δ 141.6, 139.2, 128.9, 128.2, 128.1, 126.0, 83.6, 80.4, 66.4, 38.4, 27.8, 20.3, 3.9. GC/MS M+ - H_2O (194.2, 100). HRMS could not be obtained on this compound. Elemental analysis was not performed. The product of this substrate is an isomer (M+ of the isomer was detected, see **2h**). Melting point 117-120 °C.

1i 3-ethynyl-4'-(trifluoromethyl)-3,4,5,6-tetrahydro-[1,1'-biphenyl]-3-ol

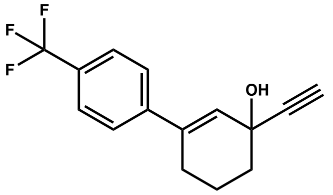

General procedure **B1** was followed using 1-bromo-4-(trifluoromethyl)benzene. Subsequently, procedure **D3** was followed using lithium acetylide (ethylenediamine complex). The product was isolated as a white solid with a yield of 38% (0.44 g, 1.7 mmol). ^{1}H NMR (400 MHz, CD_2Cl_2) δ 7.63 – 7.52 (m, 4H), 6.14 (t, *J* = 1.9 Hz, 1H), 2.61 (s, 1H), 2.54 – 2.36 (m, 2H), 2.23 (s, 1H), 2.16 – 2.04 (m, 1H), 2.02 – 1.89 (m, 3H). ^{19}F NMR (376 MHz, CD_2Cl_2) δ -62.8. ^{13}C NMR (101 MHz, CD_2Cl_2) δ 145.1, 139.2, 129.9 (q, *J* = 32.4 Hz), 129.1, 126.5, 125.8 (q, *J* = 3.8 Hz), 124.9 (q, *J* = 271.7 Hz), 87.8, 72.4, 66.2, 37.9, 27.7, 20.0. GC/MS (m/z, relative intensity) M+ (266.1, 15). Melting point 88-90 °C.

1j 4'-methyl-3,4,5,6-tetrahydro-[1,1'-biphenyl]-3-ol

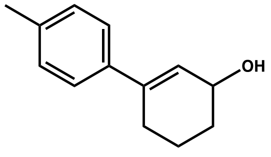

General procedure **B1** was followed using 1-bromo-4-methylbenzene. The resulting enone was reduced as in procedure **C1**. The product was isolated as a white solid with a yield of 84% (0.86 g, 4.6 mmol). ^{1}H NMR (400 MHz, $CDCl_3$) δ 7.34 – 7.30 (m, 2H), 7.14 (d, *J* = 8.0 Hz, 2H), 6.11 (dt, *J* = 3.6, 1.8 Hz, 1H), 4.43 – 4.34 (m, 1H), 2.54 – 2.28 (m, 5H), 2.01 – 1.85 (m, 3H), 1.81 – 1.61 (m, 2H). ^{13}C NMR (101 MHz, $CDCl_3$) δ 139.9, 138.5, 137.2, 129.0, 125.8, 125.3, 66.4, 31.7, 27.5, 21.1, 19.5. GC/MS M+ -H_2O (170.1, 100). HRMS could not be obtained on this compound. Elemental analysis was not performed. This substrate is isomeric with the ring-opened product and the M+ of the ring-opened isomer was detected (see **2j**). Melting point 71-73 °C.

1k 4'-(trifluoromethyl)-3,4,5,6-tetrahydro-[1,1'-biphenyl]-3-ol

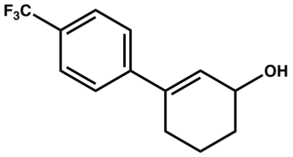

General procedure **B1** was followed using 1-bromo-4-(trifluoromethyl)benzene. The resulting enone was reduced as in procedure **C1**. The product was isolated as a white solid with a yield of 63% (1.1 g, 4.5 mmol). ^{1}H NMR (400 MHz, Acetonitrile-d3) δ 7.64 (d, *J* = 8.6 Hz, 2H), 7.60 (d, *J* = 8.6 Hz, 2H), 6.20 (dt, *J* = 3.5, 1.8 Hz, 1H), 4.28 (s, 1H), 2.93 (d, *J* = 5.5 Hz, 1H), 2.53 – 2.28 (m, 2H), 1.93 – 1.85 (m, 2H), 1.75 – 1.63 (m, 1H), 1.61 – 1.48 (m, 1H). ^{19}F NMR (376 MHz, CD3CN) δ -62.9. ^{13}C NMR (101 MHz, Acetonitrile-d3) δ 146.6, 138.4, 131.1, 129.3 (q, J = 32.2 Hz), 126.8, 126.2 (q, J = 3.9 Hz), 125.6 (q, J = 271.0 Hz), 66.5, 32.3, 27.9, 20.5. GC/MS (m/z, relative intensity) M+ (242.1, 40). Melting point 65-66 °C.

1l 3,4,5,6-tetrahydro-[1,1'-biphenyl]-3-ol

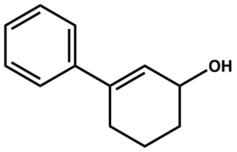

General procedure **B1** was followed using bromobenzene. The resulting enone was reduced as in procedure **C1**. The product was isolated as a white solid with a yield of 52% (0.52 g, 3.0 mmol). ^{1}H NMR (400 MHz, CD_2Cl_2) δ 7.46 – 7.41 (m, 2H), 7.37 – 7.31 (m, 2H), 7.30 – 7.24 (m, 1H), 6.13 (dt, *J* = 3.5, 1.8 Hz, 1H), 4.36 (s, 1H), 2.53 – 2.30 (m, 2H), 2.04 (d, *J* = 3.9 Hz, 1H), 1.99 – 1.86 (m, 2H), 1.80 – 1.58 (m, 2H). ^{13}C NMR (101 MHz, CD_2Cl_2) δ 142.1, 140.2, 128.8, 127.8, 127.5, 125.9, 66.8, 32.3, 28.0, 20.1. GC/MS M+ (174.1, 75). Melting point 63-65 °C.

1m 4'-methoxy-3,4,5,6-tetrahydro-[1,1'-biphenyl]-3-ol

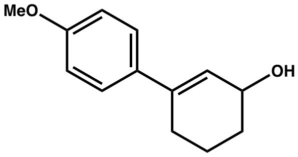

General procedure **B1** was followed using 1-bromo-4-methoxybenzene. The resulting enone was reduced as in procedure **C1**. The product was isolated as a white solid with a yield of 80% (0.80 g, 3.9 mmol). ^{1}H NMR (400 MHz, CD_2Cl_2) δ 7.39 – 7.33 (m, 2H), 6.89 – 6.82 (m, 2H), 6.04 (dt, *J* = 3.6, 1.7 Hz, 1H), 4.37 – 4.29 (m, 1H), 3.79 (s, 3H), 2.49 – 2.27 (m, 2H), 1.97 – 1.82 (m, 2H), 1.78 – 1.57 (m, 2H), 1.54 (d, *J* = 3.0 Hz, 1H). ^{13}C NMR (101 MHz, CD_2Cl_2) δ 159.7, 139.6, 134.4, 126.9, 125.8, 114.1, 66.8, 55.8, 32.4, 28.0, 20.1. GC/MS M+ -H_2O (186.2, 100). HRMS could not be obtained on this compound. Elemental analysis was not performed. The product of this substrate is an isomer (HRMS of the isomer was obtained, see **2m**). Melting point 74-78 °C.

1p 1,3-dibutylcyclohex-2-en-1-ol

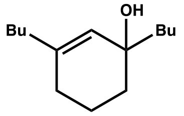

Procedure **D3** was followed using intermediate **A** as the enone and n-butyllithium as the organolithium reagent. Subsequently, procedure **D3** was followed using n-butyllithium. The substrate was isolated as a yellow oil with a yield of 49% (1.2 g, 5.7 mmol). ^{1}H NMR (400 MHz, $CDCl_3$) δ 5.31 (s, 1H), 2.01 – 1.77 (m, 4H), 1.74 – 1.58 (m, 3H), 1.57 – 1.19 (m, 12H), 0.92 – 0.83 (m, 6H). ^{13}C NMR (101 MHz, $CDCl_3$) δ 141.7, 127.0, 70.4, 42.6, 37.5, 35.4, 29.8, 28.8, 26.0, 23.4, 22.5, 19.6, 14.2, 14.1 GC/MS M+ -H_2O (192.2, 40). HRMS could not be obtained on this compound. Elemental analysis was not performed.

1q 3-hydroxycyclohex-1-ene-1-carbonitrile

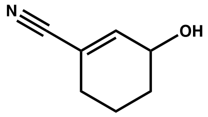

Procedure **P1** was followed to synthesize the substrate as a clear oil with a yield of 29% (1.0 g, 8.3 mmol). ^{1}H NMR (400 MHz, CD_3CN) δ 6.57 – 6.50 (m, 1H), 4.22 – 4.12 (m, 1H), 3.24 (d, *J* = 5.3 Hz, 1H), 2.28 – 2.07 (m, 2H), 1.92 – 1.71 (m, 2H), 1.66 – 1.54 (m, 1H), 1.53 – 1.43 (m, 1H). ^{13}C NMR (101 MHz, CD_3CN) δ 147.8, 120.0, 114.6, 65.2, 31.0, 27.3, 19.5. GC/MS (m/z, relative intensity) M+ (123.1, 100).

1r 4''-methoxy-4-(trifluoromethyl)-5',6'-dihydro-[1,1':3',1''-terphenyl]-1'(4'H)-ol

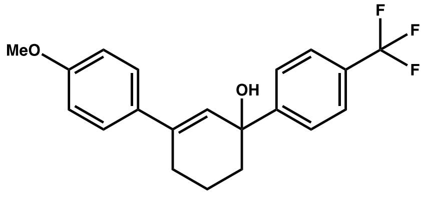

General procedure **B1** was followed using 1-bromo-4-methoxybenzene. Subsequently, general procedure **D1** was followed using 1-bromo-4-(trifluoromethyl)benzene. The substrate was isolated as a white solid with a yield of 59% (2.0 g, 5.7 mmol). ^{1}H NMR (400 MHz, CDCl3) δ 7.64 (d, *J* = 8.4 Hz, 1H), 7.59 (d, *J* = 8.5 Hz, 1H), 7.44 – 7.39 (m, 2H), 6.92 – 6.86 (m, 2H), 6.04 (s, 1H), 3.83 (s, 3H), 2.65 – 2.41 (m, 2H), 2.09 – 1.78 (m, 5H). ^{19}F NMR (376 MHz, $CDCl_3$) δ -62.4. ^{13}C NMR (101 MHz, $CDCl_3$) δ 159.6, 152.3, 140.6, 133.4, 129.2 (q, *J* = 32.2 Hz), 126.7, 126.5, 126.1, 125.8 (q, *J* = 3.8 Hz), 124.4 (q, *J* = 271.9 Hz), 113.9, 72.9, 55.4, 39.4, 27.6, 19.7. GC/MS M+ -H_2O (330.2, 100). HRMS could not be obtained on this compound. Elemental analysis was not performed. Melting point 107-110 °C.

1s 4-methoxy-4''-(trifluoromethyl)-5',6'-dihydro-[1,1':3',1''-terphenyl]-1'(4'H)-ol

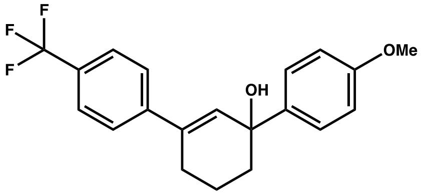

General procedure **B1** was followed using 1-bromo-4-(trifluoromethyl)benzene. Subsequently, general procedure **D1** was followed using 1-bromo-4-methoxybenzene. The substrate was isolated as a colorless oil with a yield of 54% (1.6 g, 5.0 mmol). ^{1}H NMR (400 MHz, CD_3CN) δ 7.66 (m, 4H), 7.42 – 7.37 (m, 2H), 6.92 – 6.85 (m, 2H), 6.17 (t, J = 1.8 Hz, 1H), 3.77 (s, 3H), 3.33 (s, 1H), 2.59 – 2.40 (m, 2H), 2.00 – 1.79 (m, 3H), 1.77 – 1.64 (m, 1H). ^{19}F NMR (376 MHz, CD_3CN) δ -62.9. ^{13}C NMR (101 MHz, CD_3CN) δ 159.5, 146.7, 141.6, 138.7, 132.8, 129.5 (q, J = 32.1 Hz), 127.8, 127.1, 126.2 (q, J = 3.9 Hz), 125.6 (q, J = 271.1 Hz), 114.2, 72.7, 55.8, 40.0, 28.0, 20.3. GC/MS M+ (318.1, 100), and M+ -H_2O (300.2, 100).

1n 3-(furan-2-yl)cyclohex-2-en-1-ol

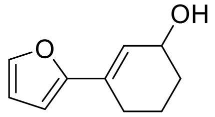

3-(furan-2-yl)cyclohex-2-en-1-one (390 mg, 2.4 mmol) from general procedure **B1** with 2-bromofuran, was set stirring at room temperature in ethanol solution with 1.5 equivalents of $NaBH_4$ (137 mg, 3.6 mmol). When deemed complete by ^{1}HNMR, the reaction mixture was quenched with saturated NH_4Cl solution, concentrated in vacuo, and extracted into EtOAc. The organic layer was washed with brine (50 mL) and subsequently dried over $MgSO_4$ before being concentrated down to afford the desired product as a viscous yellow oil (355 mg, 90% yield). 1H NMR (599 MHz, Acetonitrile-d3) δ 7.43 (d, J = 1.8 Hz, 1H), 6.42 (dd, J = 3.4, 1.8 Hz, 1H), 6.34 (d, J = 3.3 Hz, 1H), 6.19 – 6.16 (m, 1H), 4.25 (s, 1H), 2.83 (s, 1H), 2.34 – 2.28 (m, 1H), 2.27 – 2.21 (m, 1H), 1.90 – 1.80 (m, 2H), 1.70 – 1.60 (m, 1H), 1.57 – 1.49 (m, 1H). 13C NMR (151 MHz, Acetonitrile-d3) δ 155.4, 142.9, 129.8, 125.7, 112.2, 106.7, 65.9, 32.6, 25.6, 19.9. GC/MS (m/z, relative intensity) M+ (164.1, 80).

1o 3-(pyridin-2-yl)cyclohex-2-en-1-ol

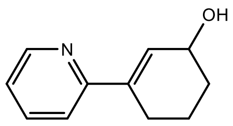

3-(pyridine-2-yl)cyclohex-2-en-1-one (780 mg, 4.5 mmol) from general procedure **B1** with 2-bromopyridine, was stirred at room temperature in ethanol. To the solution was added 1.5 equivalents of $NaBH_4$ (255 mg, 6.8 mmol). The reaction was monitored via NP-TLC. The reaction was quenched with water, concentrated in vacuo, dissolved in CH_2Cl_2. The solution was then washed with 1 M NaOH, brine and then subsequently dried over $MgSO_4$ before being concentrated down to afford the

crude product as a light-brown solid (1076 mg). An aliquot (250 mg) was drawn from the crude product and was subjected to silica gel column chromatography (DCM/MeOH, buffered with 1% triethylamine). The pyridinyl alcohol was isolated as a light-brown oil (40 mg, 16% yield). ^{1}H NMR (800 MHz, chloroform-d) δ 8.55 (ddd, J = 4.8 Hz, 1.8 Hz, 0.9 Hz, ^{1}H), δ 7.63 (td, J = 8.1 Hz, 1.9 Hz, ^{1}H), δ 7.41 (d, J = 8.0 Hz, ^{1}H), δ 7.14 (ddd, 7.4 Hz, 4.8 Hz, 1.1 Hz, ^{1}H), δ 6.61 (dt, 3.6 Hz, 1.8 Hz, ^{1}H), δ 2.57 (m, ^{1}H), δ 2.55 (m, ^{1}H), δ 2.48 (m, ^{1}H), δ 2.46 (m, ^{1}H), δ 1.97 (m, ^{1}H), δ 1.92 (m, ^{1}H), δ 1.73 (m, ^{1}H), δ 1.67 (m, ^{1}H). ^{13}C NMR (200 MHz, chloroform-d) δ 158.1, 148.9, 139.6, 136.3, 129.8, 122.2, 119.7, 66.2, 31.7, 26.0, 19.5. CI-GC/MS (m/z, relative intensity) M+1 (176).

3 3-phenylcyclohept-2-en-1-ol

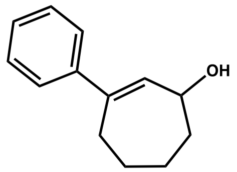

Procedure **P2** was followed to synthesize the substrate as an oil with a yield of 89% (0.14 g, 0.74 mmol). ^{1}H NMR (400 MHz, CD_2Cl_2) δ 7.37 – 7.27 (m, 4H), 7.25 – 7.19 (m, 1H), 5.96 (dd, *J* = 3.5, 1.7 Hz, 1H), 4.57 (d, *J* = 7.3 Hz, 1H), 2.64 (m, 1H), 2.52 – 2.42 (m, 1H), 2.06 – 1.96 (m, 1H), 1.89 – 1.61 (m, 5H), 1.49 – 1.36 (m, 1H). ^{13}C NMR (101 MHz, CD_2Cl_2) δ 144.6, 142.2, 136.7, 128.7, 127.2, 126.2, 72.6, 37.2, 33.2, 28.7, 26.7. GC/MS (m/z, relative intensity) M+ (188.1, 95) and M+ -H_2O (170.1, 80).

5 3-phenylcyclopent-2-en-1-ol

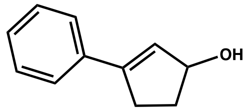

Procedure **A1** was followed using 1,3-cyclopentanedione to form the intermediate for use with procedure **B1**. Procedure **B1** was followed using bromobenzene. The isolated enone was then reduced as in procedure **C1** to produce the substrate as a white solid with a yield of 63% (0.32 g, 2.0 mmol). ^{1}H NMR (400 MHz, CD_3CN) δ 7.55 – 7.48 (m, 2H), 7.38 – 7.32 (m, 2H), 7.31 – 7.25 (m, 1H), 6.22 (q, *J* = 2.1 Hz, 1H), 4.87 (q, *J* = 5.9, 5.5 Hz, 1H), 2.91 – 2.79 (m, 2H), 2.66 – 2.56 (m, 1H), 2.36 (m, 1H), 1.81 – 1.71 (m, 1H). ^{13}C NMR (101 MHz, CD_3CN) δ 145.5, 137.1, 129.5, 129.4, 128.8, 126.9, 77.8, 34.4, 32.0. GC/MS M+ -H_2O (142.2, 100). HRMS could not be obtained on this compound. Elemental analysis was not performed. Melting point 90-93 °C.

7 1',2',5',6'-tetrahydro-[1,1':3',1''-terphenyl]-5'-ol

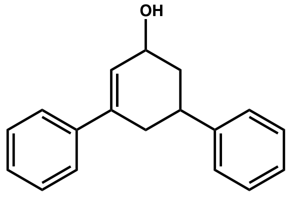

Procedure **P3** was followed to produce the substrate as a clear oil with a yield of 45% (2.2 g, 8.9 mmol). ^{1}H NMR (400 MHz, CD_3CN) δ 7.48 – 7.44 (m, 2H), 7.36 – 7.30 (m, 6H), 7.29 – 7.20 (m, 2H), 6.12 (dt, *J* = 2.5, 1.2 Hz, 1H), 4.58 – 4.47 (m, 1H), 3.08 (d, *J* = 5.8 Hz, 1H), 3.02 (dddd, *J* = 13.4, 11.0, 5.2, 2.5 Hz, 1H), 2.68 – 2.57 (m, 1H), 2.53 (dddd, *J* = 17.1, 11.1, 3.7, 2.5 Hz, 1H), 2.26 – 2.15 (m, 1H), 1.74 (ddd, *J* = 13.2, 11.8, 10.0 Hz, 1H). ^{13}C NMR (101 MHz, CD_3CN) δ 147.1, 142.0, 138.3, 129.8, 129.5, 129.4, 128.3, 127.9, 127.3, 126.3, 69.2, 40.4, 39.9, 36.8. GC/MS M+ -H_2O (232.0, 100). HRMS could not be obtained on this compound. Elemental analysis was not performed. The product of this substrate is an isomer (M+ of the isomer was detected, see **8**). Specific diastereomer not confirmed.

9 5'-phenyl-4-(trifluoromethyl)-3',4'-dihydro-[1,1':3',1''-terphenyl]-1'(2'H)-ol

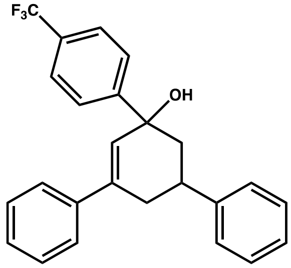

Procedure **P4** was followed to synthesize the substrate as a white solid with a yield of 32% (0.38 g, 1.0 mmol). ^{1}H NMR (400 MHz, CD_2Cl_2) δ 7.80 (d, *J* = 8.2 Hz, 2H), 7.69 (d, *J* = 8.2 Hz, 2H), 7.62 – 7.57 (m, 2H), 7.48 – 7.30 (m, 5H), 7.29 – 7.21 (m, 3H), 6.23 (s, 1H), 2.98 – 2.84 (m, 2H), 2.71 (m, 1H), 2.45 – 2.33 (m, 2H), 2.30 (s, 1H). ^{19}F NMR (376 MHz, CD_2Cl_2) δ -62.7. ^{13}C NMR (101 MHz, CD_2Cl_2) δ 151.5, 145.4, 141.1, 140.3, 129.8 (q, *J* = 32.2 Hz), 129.1, 129.1, 128.6, 128.5, 127.6, 127.4, 127.1, 126.1, 125.6 (q, *J* = 3.8 Hz), 125.0 (q, *J* = 271.9 Hz), 75.8, 46.3, 38.2, 36.8. One quartet of quaternary carbon signal not fully resolved. GC/MS M+ -H_2O (376.3, 80). HRMS could not be obtained on this compound. Elemental analysis was not performed. The product of this substrate is an isomer (M+ of the isomer was detected, see **10**). Melting point 123-126 °C. Specific diasteromer not confirmed.

11a 5-methyl-4'-(trifluoromethyl)-3,4-dihydro-[1,1'-biphenyl]-1(2H)-ol

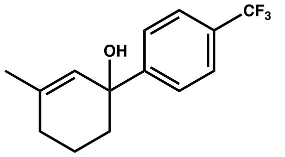

General procedure **D3** was performed with enone **A** using methyllithium; subsequently, general procedure **D1** was followed using 1-bromo-4-(trifluoromethyl)benzene. The substrate was isolated as a colorless oil with a yield of 42% (0.36 g, 1.4 mmol). ^{1}H NMR (400 MHz, C_6D_6) δ 7.46 – 7.38 (m, 4H), 5.19 (p, *J* = 1.5 Hz, 1H), 1.78 – 1.70 (m, 1H), 1.64 (t, *J* = 6.0 Hz, 2H), 1.61 – 1.41 (m, 5H), 1.39 – 1.27 (m, 2H). ^{19}F NMR (376 MHz, C_6D_6) δ -61.9. ^{13}C NMR (101 MHz, C_6D_6) δ 153.3, 138.7, 129.0 (q, *J* = 32.1 Hz), 127.0, 126.4, 125.3 (q, *J* = 271.7 Hz), 125.1 (q, *J* = 3.8 Hz), 72.23, 39.5, 30.0, 23.7, 19.5. GC/MS (m/z, relative intensity) M+ (256.1, 30).

11b 3-methyl-4'-(trifluoromethyl)-3,4,5,6-tetrahydro-[1,1'-biphenyl]-3-ol

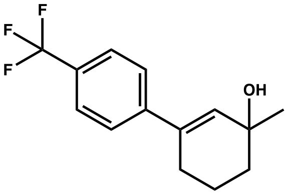

General procedure **B1** was followed using 1-bromo-4-(trifluoromethyl)benzene. Subsequently, procedure **D3** was followed using methyllithium to produce the substrate as a white solid with a yield of 29% (0.30 g, 1.2 mmol). ^{1}H NMR (400 MHz, $CDCl_3$) δ 7.57 (d, *J* = 8.3 Hz, 2H), 7.48 (d, *J* = 8.2 Hz, 2H), 6.05 (s, 1H), 2.49 – 2.29 (m, 2H), 1.96 – 1.77 (m, 3H), 1.72 (d, *J* = 10.7 Hz, 2H), 1.39 (s, 3H). ^{19}F NMR (376 MHz, $CDCl_3$) δ -62.5. ^{13}C NMR (101 MHz, $CDCl_3$) δ 145.1 (d, *J* = 1.5 Hz), 137.7, 132.5, 129.4 (q, *J* = 32.3 Hz), 125.8, 125.4 (q, *J* = 3.8 Hz), 124.4 (q, *J* = 271.8 Hz), 68.7, 37.5, 29.7, 27.6, 20.0. GC/MS (m/z, relative intensity) M+ (256.1, 30). Melting point 54-56 °C.

13 (3*R*,10a*R*)-1,2,3,9,10,10a-hexahydrophenanthren-3-ol

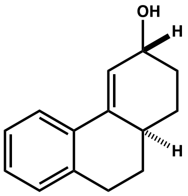

Procedure **P5** was followed to produce the substrate as a colorless oil with a yield of 64% (0.20 g, 1.0 mmol). ^{1}H NMR (400 MHz, $CDCl_3$) δ 7.65 (dd, *J* = 7.3, 2.0 Hz, 1H), 7.21 – 7.07 (m, 3H), 6.27 (s, 1H), 4.46 (ddt, *J* = 9.4, 6.0, 2.8 Hz, 1H), 2.99 – 2.77 (m, 2H), 2.39 – 2.14 (m, 3H), 2.04 – 1.88 (m, 2H), 1.55 – 1.27 (m, 3H). ^{13}C NMR (101 MHz, $CDCl_3$) δ 138.6, 137.1, 133.7, 129.4, 127.5, 126.0, 124.0, 123.5, 68.3, 36.2, 32.1, 31.4, 30.0, 29.2. GC/MS M+ (200.1, 70), and M+ -H_2O (182.1, 100). HRMS could not be obtained on this compound. Elemental analysis was not performed. The product of this substrate is an isomer (M+ of the isomer was detected, see **14**).

IV. **General Procedure E for Photocatalytic Reactions**

Light-promoted reactions were setup in NMR tubes charged with substrate (1 equiv), benzoic acid (1.1 equiv), and catalyst **PC1** (0.25 mol%) dissolved in dichloromethane (1.0 mg/mL, 1.41 mM) or toluene (0.3 mg/mL, 0.42 mM) stock solutions. Deuterated benzene (C_6D_6), sealed in a glass capillary tube, was added to aid in the NMR locking process. A rubber septum was used to seal the NMR tube, which was then degassed by sparging with argon for 10 min. The degassed NMR tubes were then placed in the photoreactor, and monitored periodically by proton and (if applicable) fluorine NMR. Upon reaction completion, the mixtures were neutralized with saturated sodium bicarbonate solution. The organic layer was washed with water and saturated sodium chloride solution. It was then dried over $MgSO_4$, filtered, and concentrated in vacuo. The crude products were then purified by flash chromatography.

V. List of Reaction Products

2a 1,5-bis(4-(trifluoromethyl)phenyl)hex-5-en-1-one

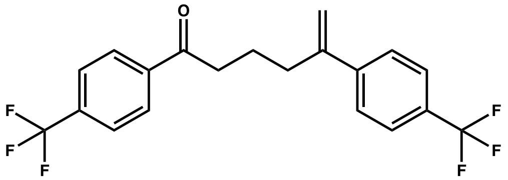

General procedure E was followed using **1a** to give the product as a colorless oil with a yield of 95% (47.5 mg, 0.12 mmol). ^{1}H NMR (400 MHz, CD_3CN) δ 8.05 (d, J = 8.1 Hz, 2H), 7.79 (d, J = 8.2 Hz, 2H), 7.69 – 7.60 (m, 4H), 5.44 (s, 1H), 5.24 (q, J = 1.3 Hz, 1H), 3.06 (t, J = 7.1 Hz, 2H), 2.64 (t, J = 7.6 Hz, 2H), 1.82 (p, J = 7.2 Hz, 2H). ^{19}F NMR (376 MHz, CD_3CN) δ -63.0, -63.6. ^{13}C NMR (101 MHz, CD_3CN) δ 200.24, 148.05, 145.78 (d, J = 1.5 Hz), 141.11 (d, J = 1.3 Hz), 134.27 (q, J = 32.4 Hz), 129.70 (q, J = 32.2 Hz), 129.48, 127.78, 126.58 (q, J = 3.8 Hz), 126.25 (q, J = 3.9 Hz), 125.00 (q, J = 271.0 Hz), 124.98 (q, J = 271.8 Hz), 115.65, 38.70, 34.84, 23.31. GC/MS (m/z, relative intensity) M+ (385.9, 5).

2b 1-phenyl-5-(4-(trifluoromethyl)phenyl)hex-5-en-1-one

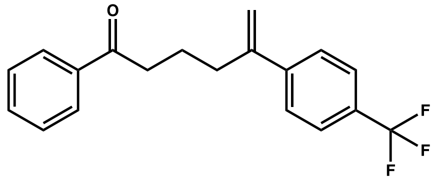

General procedure **E** was followed using **1b** to give the product as a colorless oil with a yield of 91% (45.5 mg, 0.14 mmol). ^{1}H NMR (400 MHz, CD_3CN) δ 7.95 – 7.89 (m, 2H), 7.70 – 7.60 (m, 4H), 7.62 – 7.56 (m, 1H), 7.52 – 7.43 (m, 2H), 5.43 (s, 1H), 5.24 (d, J = 1.2 Hz, 1H), 3.02 (t, J = 7.1 Hz, 2H), 2.64 (t, J = 7.6 Hz, 2H), 1.80 (p, J = 7.3 Hz, 2H). ^{19}F NMR (376 MHz, CD_3CN) δ -63.0. ^{13}C NMR (101 MHz, CD_3CN) δ 200.9, 148.2, 145.8, 138.1, 133.9, 129.7 (q, J = 32.2 Hz), 129.6, 128.8, 127.8, 126.2 (q, J = 3.9 Hz), 125.5 (q, J = 271.0 Hz) 115.6, 38.4, 35.0, 23.6. GC/MS (m/z, relative intensity) M+ (318.1, <5).

2c 5-phenyl-1-(4-(trifluoromethyl)phenyl)hex-5-en-1-one

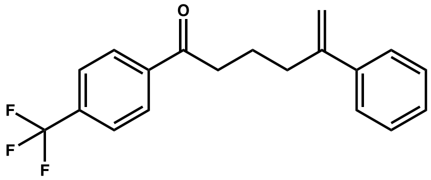

General procedure **E** was followed using **1c** to give the product as a colorless oil with a yield of 92% (46 mg, 0.14 mmol). ^{1}H NMR (400 MHz, CD_3CN) δ 8.05 (d, J = 8.1 Hz, 2H), 7.79 (d, J = 8.2 Hz, 2H), 7.49 – 7.45 (m, 2H), 7.38 – 7.25 (m, 3H), 5.32 (d, J = 1.5 Hz, 1H), 5.11 (q, J = 1.4 Hz, 1H), 3.05 (t, J = 7.2 Hz, 2H), 2.62 (td, J = 7.5, 1.3 Hz, 2H), 1.81 (p, J = 7.3 Hz, 2H). ^{19}F NMR (376 MHz, CD_3CN) δ -63.6. ^{13}C NMR (101 MHz, CD_3CN)

δ 200.4, 149.2, 141.8, 141.2, 134.2 (q, *J* = 32.3 Hz), 129.5, 129.4, 128.5, 127.1, 126.6 (q, *J* = 3.9 Hz), 125.0 (q, *J* = 271.8 Hz), 113.4, 38.8, 35.1, 23.5. GC/MS (m/z, relative intensity) M+ (318.1, 5).

2d 1,5-diphenylhex-5-en-1-one

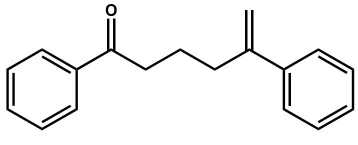

General procedure **E** was followed using **1d** to give the product as a colorless oil with a yield of 88% (44 mg, 0.18 mmol). ^{1}H NMR (400 MHz, Chloroform-d) δ 7.86 – 7.80 (m, 2H), 7.49 – 7.43 (m, 1H), 7.38 – 7.32 (m, 4H), 7.28 – 7.22 (m, 2H), 7.22 – 7.14 (m, 1H), 5.24 (d, *J* = 1.4 Hz, 1H), 5.02 (q, *J* = 1.4 Hz, 1H), 2.90 (t, *J* = 7.3 Hz, 2H), 2.55 (td, *J* = 7.4, 1.3 Hz, 2H), 1.84 (p, *J* = 7.4 Hz, 2H). ^{13}C NMR (101 MHz, CDCl3) δ 200.3, 147.9, 141.0, 137.1, 133.0, 128.7, 128.5, 128.1, 127.6, 126.3, 113.1, 37.9, 34.8, 22.8. GC/MS (m/z, relative intensity) M+ (250.2, 5).

2e 6-phenylhept-6-en-2-one

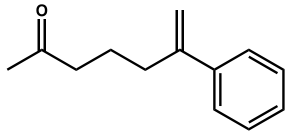

General procedure **E** was followed using **1e** to give the product as a colorless oil with a yield of 92% (46 mg, 0.24 mmol). ^{1}H NMR (400 MHz, CD_2Cl_2) δ 7.45 – 7.40 (m, 2H), 7.37 – 7.30 (m, 2H), 7.29 – 7.24 (m, 1H), 5.30 (d, *J* = 1.5 Hz, 1H), 5.07 (q, *J* = 1.4 Hz, 1H), 2.51 (td, *J* = 7.5, 1.3 Hz, 2H), 2.44 (t, *J* = 7.3 Hz, 2H), 2.07 (s, 3H), 1.70 (p, *J* = 7.4 Hz, 2H). ^{13}C NMR (101 MHz, CD_2Cl_2) δ 208.9, 148.6, 141.5, 128.8, 128.0, 126.6, 113.0, 43.2, 35.0, 30.2, 22.8. GC/MS (m/z, relative intensity) M+ (188.0, 15).

2f 8-(4-(trifluoromethyl)phenyl)nona-1,8-dien-4-one

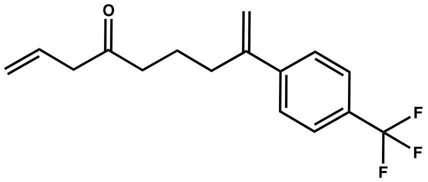

General procedure **E** was followed using **1f** to give the product as a colorless oil with a yield of 82% (41 mg, 0.15 mmol). ^{1}H NMR (400 MHz, $CDCl_3$) δ 7.58 (d, *J* = 8.3 Hz, 2H), 7.50 (d, *J* = 8.2 Hz, 2H), 5.90 (ddt, *J* = 17.2, 10.2, 7.0 Hz, 1H), 5.36 (s, 1H), 5.20 – 5.09 (m, 3H), 3.14 (dt, *J* = 7.0, 1.4 Hz, 2H), 2.52 (td, *J* = 7.5, 1.2 Hz, 2H), 2.47 (t, *J* = 7.2 Hz, 2H), 1.73 (p, *J* = 7.3 Hz, 2H). ^{19}F NMR (376 MHz, $CDCl_3$) δ -62.5. ^{13}C NMR (101 MHz, $CDCl_3$) δ 208.4, 146.9, 144.6, 130.7, 129.6 (q, *J* = 32.4 Hz), 126.6, 125.46 (q, *J* = 3.8 Hz), 124.3

(q, J = 271.9 Hz), 119.0, 114.9, 48.0, 41.4, 34.5, 22.0. GC/MS (m/z, relative intensity) M+ (282.2, 20).

2g 1-(dimethylamino)-8-(4-(trifluoromethyl)phenyl)non-8-en-2-yn-4-one

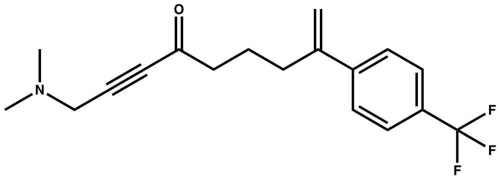

General procedure **E** was followed using **1g** to give the product as a yellow oil with a yield of 89% (45 mg, 0.14 mmol). ^{1}H NMR (400 MHz, CD_3CN) δ 7.66 (d, J = 8.3 Hz, 2H), δ 7.62 (d, J = 8.4 Hz, 2H), 5.43 (s, 1H), 5.22 (q, J = 1.3 Hz, 1H), 3.39 (s, 2H), 2.62 – 2.54 (m, 4H), 2.20 (s, 6H), 1.76 (p, J = 7.4 Hz, 2H). ^{19}F NMR (376 MHz, CD_3CN) δ -63.0. ^{13}C NMR (101 MHz, CD_3CN) δ 187.8, 147.4, 145.2, 129.3 (q, J = 32.2 Hz), 127.4, 125.9 (q, J = 3.8 Hz), 125.1 (q, J = 271.1 Hz), 115.4, 88.8, 84.9, 47.7, 45.0, 43.8, 34.3, 23.0. GC/MS did not show M+; however, HRMS was obtained on this compound. HRMS (m/z) calculated for $C_{18}H_{20}F_3NO$ (Orbitrap-FTMS, $(M + H)^+$) 324.1575, found 324.1565.

2h 8-phenylnon-8-en-2-yn-4-one

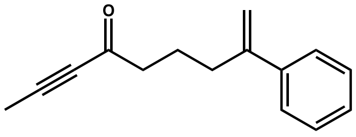

General procedure **E** was followed using **1h** to give the product as a colorless oil with a yield of 92% (46 mg, 0.22 mmol). ^{1}H NMR (400 MHz, CD_2Cl_2) δ 7.44 – 7.40 (m, 2H), 7.36 – 7.31 (m, 2H), 7.30 – 7.25 (m, 1H), 5.31 (d, J = 1.5 Hz, 1H), 5.08 (q, J = 1.3 Hz, 1H), 2.54 (t, J = 7.4 Hz, 2H), 2.53 (t, J = 7.4 Hz, 2H), 1.99 (s, 3H), 1.78 (p, J = 7.4 Hz, 2H). ^{13}C NMR (101 MHz, CD_2Cl_2) δ 188.2, 148.3, 141.4, 128.9, 128.0, 126.7, 113.2, 90.4, 80.5, 45.2, 34.9, 23.1, 4.3. GC/MS (m/z, relative intensity) M+ (211.0, <5).

2i 7-(4-(trifluoromethyl)phenyl)oct-7-en-1-yn-3-one

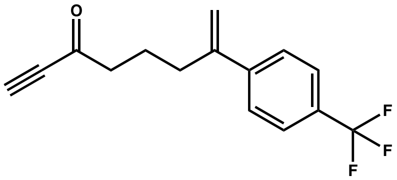

General procedure **E** was followed using **1i** to give the product as a colorless oil with a yield of 93% (47 mg, 0.17 mmol). ^{1}H NMR (400 MHz, CD_2Cl_2) δ 7.60 (d, J = 8.3 Hz, 2H), 7.54 (d, J = 8.2 Hz, 2H), 5.40 (d, J = 1.1 Hz, 1H), 5.20 (q, J = 1.2 Hz, 1H), 3.26 (s, 1H), 2.62 (t, J = 7.2 Hz, 2H), 2.56 (td, J = 7.5, 1.3 Hz, 2H), 1.80 (p, J = 7.4 Hz, 2H). ^{19}F NMR (376 MHz, CD_2Cl_2) δ -62.8. ^{13}C NMR (101 MHz, CD_2Cl_2) δ 187.3, 147.1, 145.1 (d, J = 1.5 Hz),

129.8 (q, J = 32.3 Hz), 127.1, 125.8 (q, J = 3.8 Hz), 124.9 (q, J = 272.27 Hz), 115.5, 81.8, 78.7, 45.1, 34.6, 22.6. GC/MS (m/z, relative intensity) M+ (265.9, 30).

2j 5-(p-tolyl)hex-5-enal

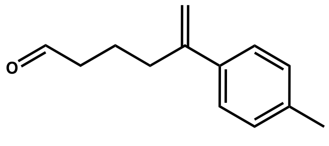

General procedure **E** was followed using **1j** to give the product as a colorless oil with a yield of 92% (46 mg, 0.24 mmol). ^{1}H NMR (400 MHz, $CDCl_3$) δ 9.74 (t, J = 1.7 Hz, 1H), 7.32 – 7.28 (m, 2H), 7.15 (d, J = 8.0 Hz, 2H), 5.29 (d, J = 1.5 Hz, 1H), 5.03 (q, J = 1.4 Hz, 1H), 2.55 (td, J = 7.4, 1.2 Hz, 2H), 2.45 (td, J = 7.3, 1.7 Hz, 2H), 2.35 (s, 3H), 1.80 (p, J = 7.3 Hz, 2H). ^{13}C NMR (101 MHz, $CDCl_3$) δ 202.5, 147.2, 137.7, 137.3, 129.1, 126.0, 112.4, 43.2, 34.5, 21.1, 20.6. GC/MS (m/z, relative intensity) M+ (188.0, 10).

2k 5-(4-(trifluoromethyl)phenyl)hex-5-enal

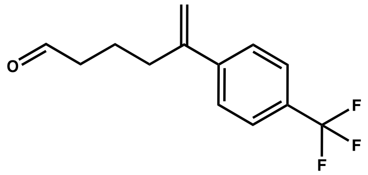

General procedure **E** was followed using **1k** to give the product as a colorless oil with a yield of 90% (45 mg, 0.19 mmol). ^{1}H NMR (400 MHz, CD_2Cl_2) δ 9.73 (t, J = 1.5 Hz, 1H), 7.61 (d, J = 8.2 Hz, 2H), 7.54 (d, J = 8.2 Hz, 2H), 5.40 (apparent singlet, 1H), 5.20 (q, J = 1.3 Hz, 1H), 2.57 (td, J = 7.5, 1.3 Hz, 2H), 2.46 (td, J = 7.2, 1.5 Hz, 2H), 1.76 (p, J = 7.4 Hz, 2H). ^{19}F NMR (376 MHz, CD_2Cl_2) δ -62.8. ^{13}C NMR (101 MHz, CD_2Cl_2) δ 202.5, 147.2, 145.1 (d, J = 1.5 Hz), 129.8 (q, J = 32.3 Hz), 127.1, 125.8 (q, J = 3.9 Hz), 124.9 (q, J = 271.7 Hz), 115.3, 43.6, 34.8, 21.0. GC/MS (m/z, relative intensity) M+ (242.0, 10).

2l 5-phenylhex-5-enal

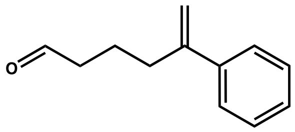

General procedure **E** was followed using **1l** to give the product as a colorless oil with a yield of 92% (46 mg, 0.26 mmol). ^{1}H NMR (400 MHz, CD_2Cl_2) δ 9.62 (t, J = 1.6 Hz, 1H), 7.34 – 7.30 (m, 2H), 7.27 – 7.21 (m, 2H), 7.20 – 7.15 (m, 1H), 5.22 (d, J = 1.4 Hz, 1H), 4.99 (q, J = 1.4 Hz, 1H), 2.46 (td, J = 7.5, 1.3 Hz, 2H), 2.34 (td, J = 7.3, 1.6 Hz, 2H), 1.66 (p, J = 7.4 Hz, 2H). ^{13}C NMR (101 MHz, CD_2Cl_2) δ 202.8, 148.3, 141.3, 128.9, 128.0, 126.6, 113.3, 43.7, 35.0, 21.2. GC/MS (m/z, relative intensity) M+ (174.0, 5).

2m 5-(4-methoxyphenyl)hex-5-enal

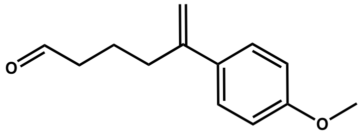

General procedure **E** was followed using **1m** to give the product as a colorless oil with a yield of 95% (48 mg, 0.23 mmol). ^{1}H NMR (400 MHz, CD_2Cl_2) δ 9.72 (t, *J* = 1.6 Hz, 1H), 7.39 – 7.34 (m, 2H), 6.90 – 6.84 (m, 2H), 5.24 (d, *J* = 1.5 Hz, 1H), 4.99 (q, *J* = 1.3 Hz, 1H), 3.80 (s, 3H), 2.53 (td, *J* = 7.5, 1.2 Hz, 2H), 2.43 (td, *J* = 7.3, 1.6 Hz, 2H), 1.76 (p, *J* = 7.4 Hz, 2H). ^{13}C NMR (101 MHz, CD_2Cl_2) δ 202.9, 159.8, 147.5, 133.6, 127.7, 114.19, 111.7, 55.8, 43.7, 35.0, 21.2. GC/MS did not show M+; however, HRMS was obtained on this compound. HRMS (m/z) calculated for $C_{13}H_{16}O_2$ (Orbitrap-FTMS, $(M + H)^{+}$) 205.1229, found 205.1226.

2n 5-(furan-2-yl)hex-5-enal

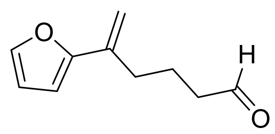

General procedure **E** was followed using **1n** (50 mg, 305 μmol) and employing buffered silica gel chromatography (gradient 0-20% CH_2Cl_2 in hexane with 1% triethylamine) to give the product as a yellow oil with a yield of 80% (40.2 mg). ^{1}H NMR (599 MHz, Acetonitrile-*d*$_3$) δ 9.70 (t, *J* = 1.5 Hz, 1H), 7.44 (d, *J* = 1.4 Hz, 1H), 6.44 (d, *J* = 1.4 Hz, 2H), 5.50 (d, *J* = 1.4 Hz, 1H), 5.00 (t, *J* = 1.3 Hz, 1H), 2.47 (td, *J* = 7.2, 1.5 Hz, 2H), 2.42 – 2.36 (m, 2H), 1.82 (p, *J* = 7.3 Hz, 2H). 13C NMR (151 MHz, Acetonitrile-d3) δ 203.7, 155.2, 143.3, 138.2, 112.3, 110.2, 107.6, 43.7, 33.1, 22.1. GC/MS (m/z, relative intensity) M+ (164, 5).

2o 5-(pyridin-2-yl)hex-5-enal

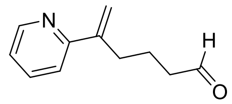

General procedure E was followed using **1o** (28 mg, 160 μmol) with a small modification to the workup. Neutralization was performed with concentrated $NaOH_{(aq)}$ (1 mL), rather than $NaHCO_3$ solution. An internal standard (1,2,4,5 tetrafluorobenzene, 78 micromoles) was used to determine a 96% NMR yield in lieu of column chromatography, which led to product degradation. ^{1}H NMR (800.3 MHz, $CDCl_3$) δ 9.76 (t, J = 1.7 Hz, 1H), δ 8.56 (ddd, J = 4.8, 1.9, 0.9 Hz, 1H), δ 7.64 (td, J = 7.7, 7.7, 1.9 Hz, 1H), δ 7.47 (dt, J = 8.0, 1.0, 1.0 Hz, 1H), δ 7.15 (ddd, J = 7.5, 4.8, 1.2 Hz, 1H), δ 5.74 (d, J = 1.2 Hz, 1H) δ 5.28 (q, J = 1.3 Hz, 1H), δ 1.68 (td, J = 7.6, 7.5, 1.3 Hz, 2H), δ 2.48 (td, J = 7.4, 7.4, 1.7 Hz, 2H), δ 1.85 (p, J = 7.4 Hz, 2H). ^{13}C NMR (201.3 MHz, CDCl3) δ 202.9, 158.3, 149.2, 147.6, 136.6, 122.5, 120.7, 115.9, 43.6, 33.2, 21.1. CI GCMS (m/z, relative intensity) [M+1] = 176.

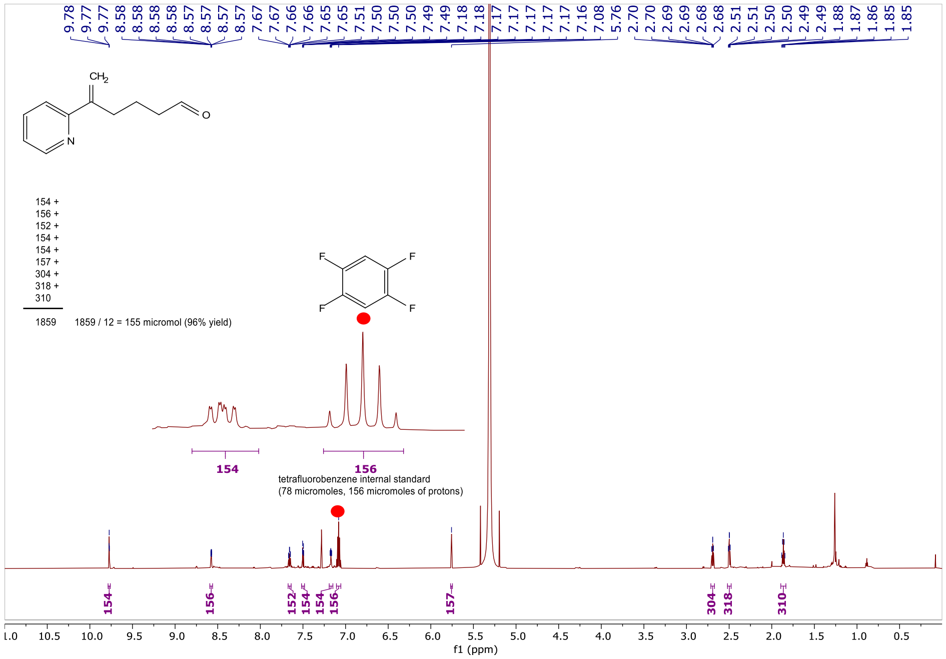

6-phenylhept-6-enal

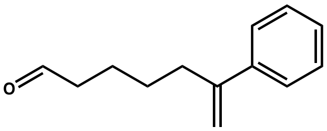

General procedure **E** was followed using **3** to give the product as a colorless oil with a yield of 87% (44 mg, 0.23 mmol). ^{1}H NMR (400 MHz, $CDCl_3$) δ 9.66 (t, *J* = 1.8 Hz, 1H), 7.34 – 7.29 (m, 2H), 7.29 – 7.23 (m, 2H), 7.22 – 7.17 (m, 1H), 5.20 (d, *J* = 1.5 Hz, 1H), 4.99 (q, *J* = 1.4 Hz, 1H), 2.46 (td, *J* = 7.4, 1.3 Hz, 2H), 2.35 (td, *J* = 7.3, 1.8 Hz, 2H), 1.64 – 1.55 (m, 2H), 1.47 – 1.37 (m, 2H). ^{13}C NMR (101 MHz, $CDCl_3$) δ 202.7, 148.1, 141.2, 128.5, 127.5, 126.2, 112.8, 43.8, 35.2, 27.8, 21.8. GC/MS (m/z, relative intensity) M+ (188.1, <5).

8 3,5-diphenylhex-5-enal

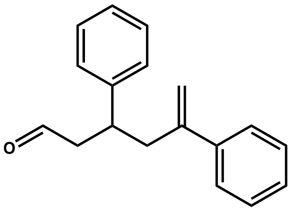

General procedure **E** was followed using **7** to give the product as a colorless oil with a yield of 89% (45 mg, 0.18 mmol). ^{1}H NMR (400 MHz, CD_2Cl_2) δ 9.56 (t, *J* = 2.0 Hz, 1H), 7.43 – 7.24 (m, 7H), 7.23 – 7.18 (m, 1H), 7.16 – 7.11 (m, 2H), 5.24 (d, *J* = 1.5 Hz, 1H), 4.96 (q, *J* = 1.3 Hz, 1H), 3.29 (dddd, *J* = 7.8, 7.8, 7.8, 6.0 Hz, 1H), 2.86 (dd, *J* = 7.5, 1.1 Hz, 2H), 2.82 – 2.67 (m, 2H). ^{13}C NMR (101 MHz, CD_2Cl_2) δ 202.1, 146.7, 144.2, 141.0, 129.0, 129.0, 128.2, 128.0, 127.1, 126.9, 115.5, 49.9, 43.4, 38.8. GC/MS (m/z, relative intensity) M+ (250.0, 5).

10 3,5-diphenyl-1-(4-(trifluoromethyl)phenyl)hex-5-en-1-one

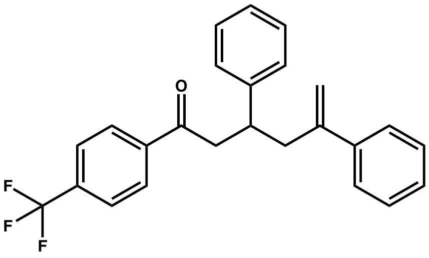

General procedure **E** was followed using **9** to give the product as a colorless oil with a yield of 82% (41 mg, 0.10 mmol). ^{1}H NMR (400 MHz, CD_2Cl_2) δ 7.88 (d, *J* = 8.1 Hz, 2H), 7.65 (d, *J* = 8.2 Hz, 2H), 7.44 – 7.09 (m, 10H), 5.25 (d, *J* = 1.5 Hz, 1H), 4.99 (d, *J* = 1.4 Hz, 1H), 3.46 – 3.22 (m, 3H), 2.92 (dddd, *J* = 14.2, 14.2, 14.0, 7.3 Hz, 2H). ^{19}F NMR (376 MHz, CD_2Cl_2) δ -63.4. ^{13}C NMR (101 MHz, CD_2Cl_2) δ 221.6, 170.1, 167.7, 164.2, 163.5, 157.5 (q, *J* = 32.7 Hz), 152.2, 152.1, 152.0, 151.4, 151.2, 150.2, 150.1, 149.2 (q, *J* = 3.8 Hz), 138.6, 68.5, 66.3, 63.3. Quaternary carbon (CF_3) signals not fully resolved. GC/MS (m/z, relative intensity) M+ (394.2, <5).

12 6-(4-(trifluoromethyl)phenyl)hept-6-en-2-one

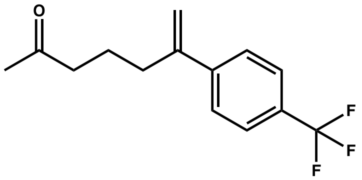

General procedure E was followed using **11a** or **11b** to give the product as a colorless oil with a yield of 90% (45 mg, 0.18 mmol). ^{1}H NMR (400 MHz, $CDCl_3$) δ 7.58 (d, *J* = 8.2 Hz, 2H), 7.50 (d, *J* = 8.2 Hz, 2H), 5.36 (s, 1H), 5.16 (d, *J* = 1.3 Hz, 1H), 2.52 (td, *J* = 7.5, 1.3 Hz, 2H), 2.45 (t, *J* = 7.2 Hz, 2H), 2.11 (s, 3H), 1.72 (p, *J* = 7.3 Hz, 2H). ^{19}F NMR (376 MHz, $CDCl_3$*d*) δ -62.5. ^{13}C NMR (101 MHz, $CDCl_3$) δ 208.6, 146.9, 144.6, 129.6 (q, *J* = 32.4 Hz), 126.5, 125.4 (q, *J* = 3.8 Hz), 124.3 (q, *J* = 271.9 Hz), 114.9, 42.8, 34.5, 30.1, 22.1. GC/MS (m/z, relative intensity) M+ (256.2, 5).

14 3-(1-methylene-1,2,3,4-tetrahydronaphthalen-2-yl)propanal

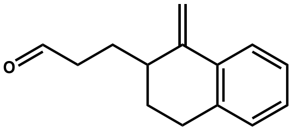

General procedure E was followed using **13** to give the product as a colorless oil with a yield of 80% (40 mg, 0.20 mmol). ^{1}H NMR (400 MHz, Chloroform-*d*) δ 9.76 (t, *J* = 1.7 Hz, 1H), 7.58 (dd, *J* = 7.5, 1.7 Hz, 1H), 7.27 – 7.08 (m, 5H), 5.48 (s, 1H), 4.95 (s, 1H), 3.03 – 2.87 (m, 1H), 2.80 (m, 1H), 2.61 – 2.47 (m, 3H), 2.10 – 1.98 (m, 1H), 1.89 – 1.71 (m, 4H). ^{13}C NMR (101 MHz, $CDCl_3$) δ 202.7, 146.2, 136.2, 134.1, 129.2, 127.9, 126.2, 125.3, 109.3, 42.1, 40.8, 28.4, 26.1, 24.3. GC/MS (m/z, relative intensity) M+ (200.1, 20).

VI. Calculations

Computational studies have been performed utilizing the Gaussian 09 package[7], on the Pete supercomputer at the High Performance Computing Center at Oklahoma State University. Several computations were performed using B3LYP theory and the 6-311++G(d,p) basis set.[8-10] Each compound was subjected to geometry optimization and frequency calculations to confirm structures are converged and stationary points are at minima. Energy calculations for the ground state *cis*-cyclohexenol **1a** and its subsequent product **2a** give evidence to the ring opening isomerization being an endothermic process, as the calculated ΔH (change in enthalpy) was found to be +2.6 kcal/mol. The sums of electronic and thermal enthalpies are listed below each molecule.

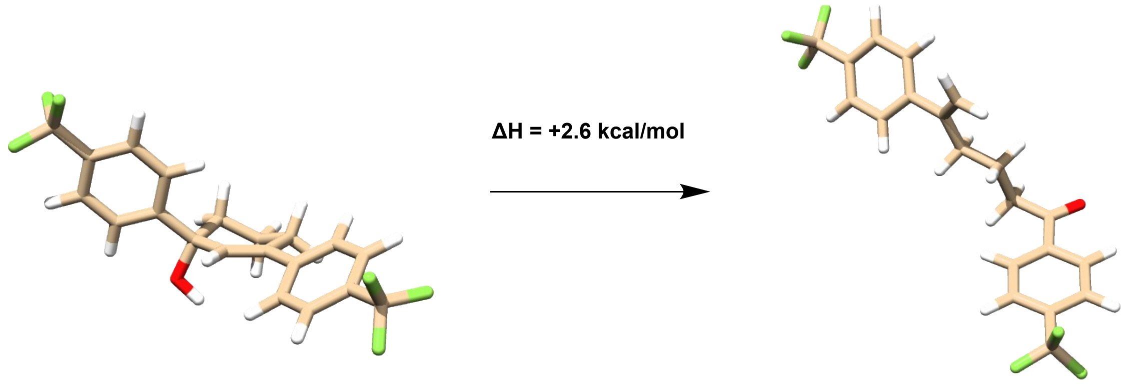

1a

-1446.129383 hartrees
(sum of electronic and thermal enthalpies)

2a

-1446.125317 hartrees
(sum of electronic and thermal enthalpies)

Upon intersystem crossing, during the formation of the *trans*-cyclohexene, four potential diastereomers are formed. Because acid pre-coordination has been found to be key,[11] of the four diastereomers, only two lead to ring opening (those with axial hydroxy groups). The diastereomers and their energies are listed below. The ΔE values represent the energy difference between ground state *cis*-cyclohexene and the ground state *trans*-cyclohexene diastereomer. The total energies for the ground state *cis*-cyclohexene (**1a**) and for the individual *trans* conformers are listed along with their cartesian coordinates on the following pages (see pages E31-E36).

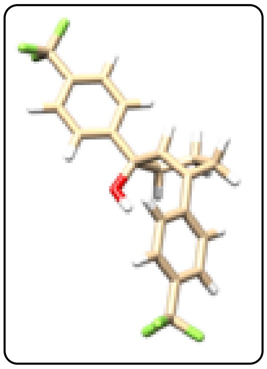

ΔE = +57.5 kcal/mol

1a-D1

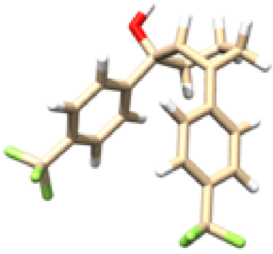

ΔE = +60.8 kcal/mol

1a-D2

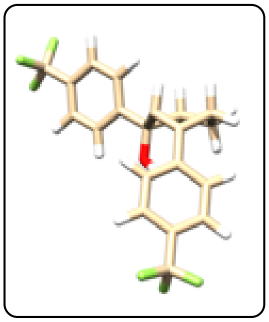

ΔE = +52.8 kcal/mol

1a-D3

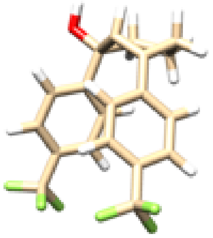

ΔE = +68.2 kcal/mol

1a-D4

1a

E = -1446.47335775 hartrees

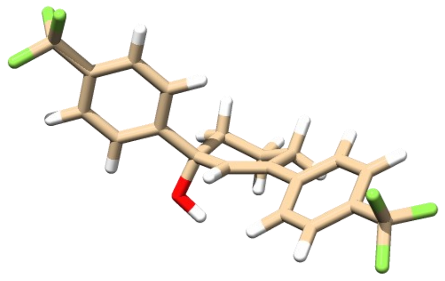

Cartesian Coordinates:
C1 -1.1260000000 2.7650000000 -1.0790000000
C2 0.0340000000 3.7080000000 -0.7450000000
C3 1.3470000000 2.9330000000 -0.6380000000
C4 1.2850000000 1.8630000000 0.4760000000
C5 -0.0420000000 1.1310000000 0.4670000000
C6 -1.1400000000 1.5290000000 -0.1990000000
C7 2.4470000000 0.8810000000 0.3260000000
O8 1.4450000000 2.4880000000 1.7700000000
C9 -2.4070000000 0.7560000000 -0.1130000000
C10 -3.2750000000 0.6750000000 -1.2120000000
C11 -4.4560000000 -0.0570000000 -1.1460000000
C12 -4.8040000000 -0.7130000000 0.0330000000
C13 -3.9610000000 -0.6330000000 1.1440000000
C14 -2.7820000000 0.0950000000 1.0690000000
C15 3.5260000000 0.8910000000 1.2110000000
C16 4.5880000000 0.0040000000 1.0430000000
C17 4.5780000000 -0.9070000000 -0.0110000000
C18 3.5020000000 -0.9280000000 -0.9000000000
C19 2.4480000000 -0.0400000000 -0.7280000000
F20 -5.8090000000 -2.8530000000 -0.1480000000
C21 -6.0560000000 -1.5410000000 0.1050000000
F22 -6.9880000000 -1.1450000000 -0.7910000000
F23 -6.6320000000 -1.4900000000 1.3290000000
F24 5.3820000000 -2.9930000000 -0.8080000000
C25 5.7460000000 -1.8290000000 -0.2220000000
F26 6.3690000000 -2.1440000000 0.9360000000
F27 6.6900000000 -1.2730000000 -1.0270000000
H28 -1.0520000000 2.4580000000 -2.1320000000
H29 -2.0820000000 3.2880000000 -0.9860000000
H30 0.1160000000 4.4860000000 -1.5100000000
H31 -0.1750000000 4.2290000000 0.1970000000
H32 1.5560000000 2.4440000000 -1.5950000000
H33 2.1890000000 3.5940000000 -0.4210000000
H34 -0.0720000000 0.2340000000 1.0770000000
H35 0.6260000000 2.9480000000 1.9800000000
H36 -3.0260000000 1.1770000000 -2.1380000000
H37 -5.1080000000 -0.1120000000 -2.0090000000
H38 -4.2370000000 -1.1230000000 2.0690000000
H39 -2.1540000000 0.1740000000 1.9480000000
H40 3.5280000000 1.5880000000 2.0370000000
H41 5.4170000000 0.0180000000 1.7400000000
H42 3.4850000000 -1.6420000000 -1.7140000000
H43 1.6100000000 -0.0760000000 -1.4160000000

2a

E = -1446.46821518

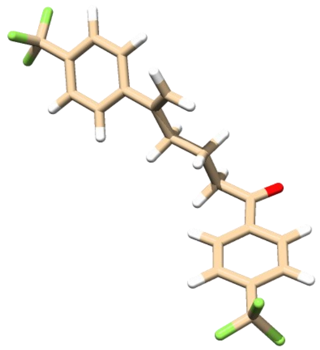

Cartesian Coordinates:
C1 -3.3730000000 0.2160000000 0.6010000000
C2 -1.9640000000 0.5470000000 0.9580000000
C3 -1.0510000000 0.9350000000 -0.1910000000
C4 0.3750000000 1.3470000000 0.1830000000
C5 1.1950000000 1.7510000000 -1.0600000000
C6 2.5930000000 2.2150000000 -0.6800000000
C7 3.6930000000 1.2050000000 -0.4840000000
C8 -4.4370000000 0.6770000000 1.3920000000
C9 -5.7550000000 0.3670000000 1.0800000000
C10 -6.0400000000 -0.4100000000 -0.0430000000
C11 -4.9990000000 -0.8720000000 -0.8470000000
C12 -3.6830000000 -0.5540000000 -0.5300000000
C13 4.9510000000 1.6720000000 -0.0740000000
C14 6.0040000000 0.7910000000 0.1220000000
C15 5.8120000000 -0.5770000000 -0.0900000000
C16 4.5690000000 -1.0580000000 -0.4950000000
C17 3.5170000000 -0.1670000000 -0.6930000000
C18 -1.5630000000 0.5030000000 2.2340000000
O19 2.8150000000 3.3980000000 -0.5020000000
F20 7.1030000000 -1.7720000000 1.4950000000
C21 6.9490000000 -1.5300000000 0.1690000000
F22 8.1290000000 -1.0410000000 -0.2730000000
F23 6.7640000000 -2.7270000000 -0.4270000000
F24 -8.3400000000 0.1680000000 0.0260000000
C25 -7.4610000000 -0.7860000000 -0.3570000000
F26 -7.8340000000 -1.9290000000 0.2780000000
F27 -7.6560000000 -1.0010000000 -1.6780000000
H28 -1.0020000000 0.1010000000 -0.9010000000
H29 -1.5310000000 1.7530000000 -0.7440000000
H30 0.3500000000 2.1950000000 0.8740000000
H31 0.8740000000 0.5260000000 0.7080000000
H32 1.2330000000 0.9210000000 -1.7720000000
H33 0.7140000000 2.5950000000 -1.5580000000
H34 -4.2270000000 1.3070000000 2.2480000000
H35 -6.5610000000 0.7440000000 1.6970000000
H36 -5.2150000000 -1.4730000000 -1.7210000000
H37 -2.8910000000 -0.9300000000 -1.1660000000
H38 5.0800000000 2.7360000000 0.0830000000
H39 6.9740000000 1.1610000000 0.4310000000
H40 4.4250000000 -2.1170000000 -0.6630000000
H41 2.5600000000 -0.5580000000 -1.0140000000
H42 -0.5530000000 0.7560000000 2.5320000000
H43 -2.2350000000 0.1950000000 3.0260000000

1a-D1

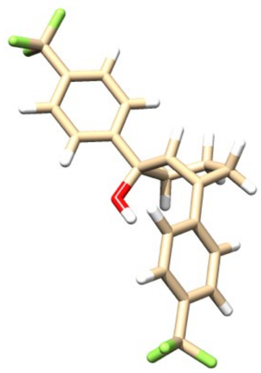

E = -1446.38178480 hartrees

Cartesian Coordinates:
C1 -0.9307850000 3.1489990000 -0.6397200000
H2 -0.3985090000 3.6637020000 -1.4437090000
C3 -1.1389730000 1.6905820000 -0.9748540000
C4 -2.3695530000 0.9748130000 -0.6599570000
C5 -3.4297420000 1.5347800000 0.0860350000
C6 -4.5887980000 0.8160160000 0.3361440000
C7 -4.7320500000 -0.4838180000 -0.1577210000
C8 -3.7059630000 -1.0559430000 -0.9097200000
C9 -2.5495670000 -0.3310340000 -1.1700390000
H10 -1.7814120000 -0.7502540000 -1.8085270000
H11 -3.8215220000 -2.0534580000 -1.3149130000
C12 -5.9712050000 -1.2730550000 0.1597430000
F13 -7.0679870000 -0.4866910000 0.2533620000
F14 -5.8627910000 -1.9233250000 1.3479900000
F15 -6.2327690000 -2.2156730000 -0.7722710000
H16 -5.3869900000 1.2638960000 0.9152290000
H17 -3.3396750000 2.5340420000 0.4922360000
C18 0.0964350000 1.0956710000 -1.0056370000
H19 0.8755590000 1.7589880000 -1.3847360000
C20 0.6154930000 0.4681360000 0.2802950000
C21 0.3169900000 1.7365340000 1.2706820000
C22 0.0086290000 3.1381560000 0.6570540000
H23 0.9449050000 3.6410430000 0.3992270000
H24 -0.4517780000 3.7415120000 1.4457080000
H25 -0.5599630000 1.4022050000 1.8299860000
H26 1.1358810000 1.8305810000 1.9879580000
O27 -0.0570350000 -0.6667640000 0.7871490000
H28 -1.0035770000 -0.5720200000 0.6295840000
C29 2.0953300000 0.1108400000 0.1986080000
C30 3.0712210000 1.1082670000 0.0934620000
C31 4.4194100000 0.7851960000 -0.0123400000
H32 5.1607850000 1.5696500000 -0.0982900000
C33 4.8131880000 -0.5524790000 -0.0108070000
C34 3.8529840000 -1.5567640000 0.1012840000
C35 2.5051060000 -1.2253250000 0.2024160000
H36 1.7609620000 -2.0043290000 0.2909280000
H37 4.1544790000 -2.5970460000 0.1031160000
C38 6.2729300000 -0.9053610000 -0.0667990000
F39 6.4921260000 -2.0945050000 -0.6721990000
F40 6.8199320000 -0.9996790000 1.1739870000
F41 6.9996130000 0.0208800000 -0.7345270000
H42 2.7879630000 2.1541230000 0.0966800000
H43 -1.8604260000 3.6971320000 -0.4735240000

1a-D2

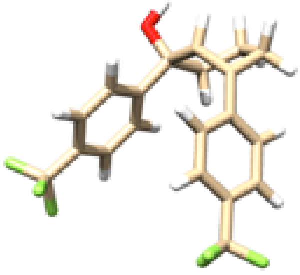

E = -1446.37654325 hartrees

Cartesian Coordinates:
C1 -4.0206370000 -1.9454190000 0.1620700000
H2 -4.5580780000 -2.6036560000 0.8490970000
C3 -2.7856010000 -1.3706680000 0.8214930000
C4 -2.3266680000 -0.0080010000 0.5662870000
C5 -2.9248560000 0.8357290000 -0.3923650000
C6 -2.4687140000 2.1340990000 -0.5875060000
C7 -1.4116130000 2.6273090000 0.1836420000
C8 -0.8160180000 1.8152310000 1.1507380000
C9 -1.2786120000 0.5220620000 1.3465010000
H10 -0.8663090000 -0.0902690000 2.1370030000
H11 -0.0056220000 2.1991180000 1.7559000000
C12 -0.8913250000 4.0139590000 -0.0718350000
F13 -1.8699460000 4.8702020000 -0.4457930000
F14 0.0330700000 4.0329340000 -1.0691730000
F15 -0.2922910000 4.5485930000 1.0131560000
H16 -2.9408890000 2.7722520000 -1.3245750000
H17 -3.7419230000 0.4764460000 -1.0017420000
C18 -1.9466210000 -2.4197740000 1.1063850000
H19 -2.4929540000 -3.3210240000 1.4086290000
C20 -0.9490990000 -2.8323120000 0.0201020000
C21 -1.8904720000 -2.6921460000 -1.2630590000
C22 -3.4266210000 -2.8423670000 -1.0246040000
H23 -3.6680500000 -3.8903650000 -0.8223050000
H24 -3.9335510000 -2.5922140000 -1.9610120000
H25 -1.7108410000 -1.6957370000 -1.6714290000
H26 -1.5680520000 -3.4195930000 -2.0120710000
C27 0.3791370000 -2.0731230000 -0.0708460000
C28 0.8703990000 -1.5006330000 -1.2409700000
H29 0.2947210000 -1.5391460000 -2.1555980000
C30 2.1081680000 -0.8572890000 -1.2621490000
C31 2.8802440000 -0.7979410000 -0.1035920000
C32 2.4148160000 -1.3977970000 1.0707630000
C33 1.1807980000 -2.0294420000 1.0798580000
H34 0.8213190000 -2.4942550000 1.9897350000
H35 3.0171720000 -1.3702210000 1.9705540000
C36 4.2320480000 -0.1375460000 -0.1011880000
F37 4.4431980000 0.6331140000 -1.1927100000
F38 5.2376050000 -1.0465060000 -0.0779620000
F39 4.4188900000 0.6533920000 0.9835930000
H40 2.4680700000 -0.4058860000 -2.1776210000
O41 -0.5449270000 -4.1977270000 0.2010820000
H42 -1.3320700000 -4.7364890000 0.3620350000
H43 -4.7307690000 -1.1963190000 -0.1936070000

1a-D3

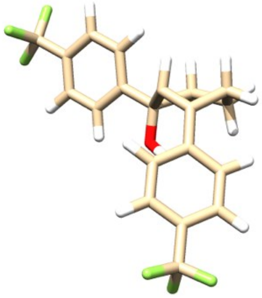

E = -1446.38914414 hartrees

Cartesian Coordinates:
C1 -1.2099260000 3.3574890000 -0.5323490000
C2 -1.2326450000 1.9023610000 -0.9342480000
C3 -2.3695710000 1.0387280000 -0.6355890000
C4 -3.5376560000 1.4883890000 0.0214740000
C5 -4.6128260000 0.6399150000 0.2358420000
C6 -4.5599690000 -0.6872030000 -0.2000650000
C7 -3.4235980000 -1.1541900000 -0.8612590000
C8 -2.3547290000 -0.2990770000 -1.0927560000
H9 -1.5004850000 -0.6438640000 -1.6623860000
H10 -3.3862190000 -2.1749820000 -1.2205580000
C11 -5.7067650000 -1.6159740000 0.0825970000
F12 -6.8955860000 -0.9704950000 0.1029930000
F13 -5.5786880000 -2.2218590000 1.2926230000
F14 -5.8043390000 -2.6050500000 -0.8331920000
H15 -5.4974730000 1.0069730000 0.7414000000
H16 -3.6000730000 2.5075980000 0.3806160000
C17 0.0720790000 1.4648390000 -0.9805480000
C18 0.6872320000 0.9726010000 0.3172130000
C19 0.6929950000 2.4247670000 1.0531060000
C20 -0.6096970000 3.2560630000 0.9329370000
H21 -1.3739460000 2.8258510000 1.5873050000
H22 -0.4121220000 4.2643330000 1.3120420000
H23 0.9031450000 2.2162410000 2.1062910000
H24 1.5339220000 3.0003530000 0.6603510000
C25 2.1060140000 0.4186820000 0.2163860000
C26 2.4337690000 -0.7991720000 0.8177430000
C27 3.7249620000 -1.3146300000 0.7309680000
C28 4.7111260000 -0.6141570000 0.0417580000
C29 4.3992160000 0.6037650000 -0.5643490000
C30 3.1099210000 1.1122340000 -0.4726910000
H31 2.8971980000 2.0605020000 -0.9512130000
H32 5.1621450000 1.1556040000 -1.0994780000
C33 6.0940450000 -1.1843240000 -0.1024860000
F34 7.0465170000 -0.2215910000 -0.0967150000
F35 6.2434300000 -1.8608330000 -1.2717280000
F36 6.4010510000 -2.0532960000 0.8860000000
H37 3.9620670000 -2.2579230000 1.2068150000
H38 1.6705410000 -1.3445290000 1.3540750000
O39 -0.0738710000 0.0656630000 1.0790850000
H40 -1.0124280000 0.1940780000 0.9017340000
H41 0.7519360000 2.1949060000 -1.4189130000
H42 -0.5259260000 3.9325290000 -1.1620960000
H43 -2.1766230000 3.8685160000 -0.5305880000

1a-D4

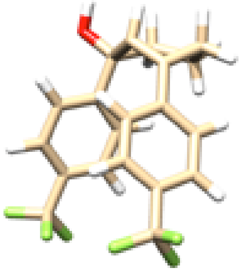

E = -1446.36461776 hartrees

Cartesian Coordinates:
C1 -3.4899460000 -2.4163000000 0.5318650000
C2 -2.5541060000 -1.7931610000 -0.4719700000
C3 -1.1059180000 -1.8851540000 -0.3268080000
C4 -0.4796010000 -2.5048080000 0.7749190000
C5 0.9003750000 -2.6196310000 0.8441760000
C6 1.6981720000 -2.1208480000 -0.1887810000
C7 1.1011500000 -1.5234950000 -1.2991280000
C8 -0.2808290000 -1.4221940000 -1.3710680000
H9 -0.7468450000 -1.0042610000 -2.2543940000
H10 1.7156950000 -1.1487350000 -2.1078000000
C11 3.1887350000 -2.2985720000 -0.1234440000
F12 3.5652390000 -3.5520720000 -0.4948640000
F13 3.6664950000 -2.1162740000 1.1307640000
F14 3.8486480000 -1.4428810000 -0.9312710000
H15 1.3615740000 -3.0875200000 1.7055280000
H16 -1.0726720000 -2.8828410000 1.5979370000
C17 -3.2219560000 -0.7631740000 -1.0953320000
C18 -3.1949210000 0.5956430000 -0.4172160000
C19 -3.9978130000 0.1562890000 0.9148710000
C20 -3.6751840000 -1.2205940000 1.5584600000
H21 -2.7651910000 -1.1481120000 2.1602260000
H22 -4.4827790000 -1.4724310000 2.2539850000
H23 -3.8862220000 0.9533150000 1.6559220000
H24 -5.0496380000 0.1635260000 0.6139770000
O25 -3.9662410000 1.5887410000 -1.0914850000
H26 -4.7379670000 1.1657050000 -1.4847350000
C27 -1.8418150000 1.2504890000 -0.1686190000
C28 -1.1825140000 1.2148670000 1.0589350000
C29 0.0543280000 1.8358580000 1.2281180000
C30 0.6397480000 2.5141870000 0.1645580000
C31 1.9907250000 3.1569940000 0.3134400000
F32 2.3330120000 3.3510490000 1.6061200000
F33 2.0486900000 4.3639300000 -0.2988560000
F34 2.9731300000 2.4009100000 -0.2405930000
C35 -0.0164200000 2.5729860000 -1.0675030000
C36 -1.2436600000 1.9466770000 -1.2276040000
H37 -1.7595110000 2.0060580000 -2.1769810000
H38 0.4283770000 3.1145820000 -1.8936510000
H39 0.5524570000 1.7976490000 2.1883200000
H40 -1.6198320000 0.7032740000 1.9052870000
H41 -4.2762210000 -1.0040470000 -1.2795580000
H42 -4.4567260000 -2.6507540000 0.0784130000
H43 -3.1227900000 -3.3136550000 1.0364940000

VII. **NMR Spectra**

1a 4,4''-bis(trifluoromethyl)-5',6'-dihydro-[1,1':3',1''-terphenyl]-1'(4'H)-ol

1H NMR at 400.15 MHz in CDCl3

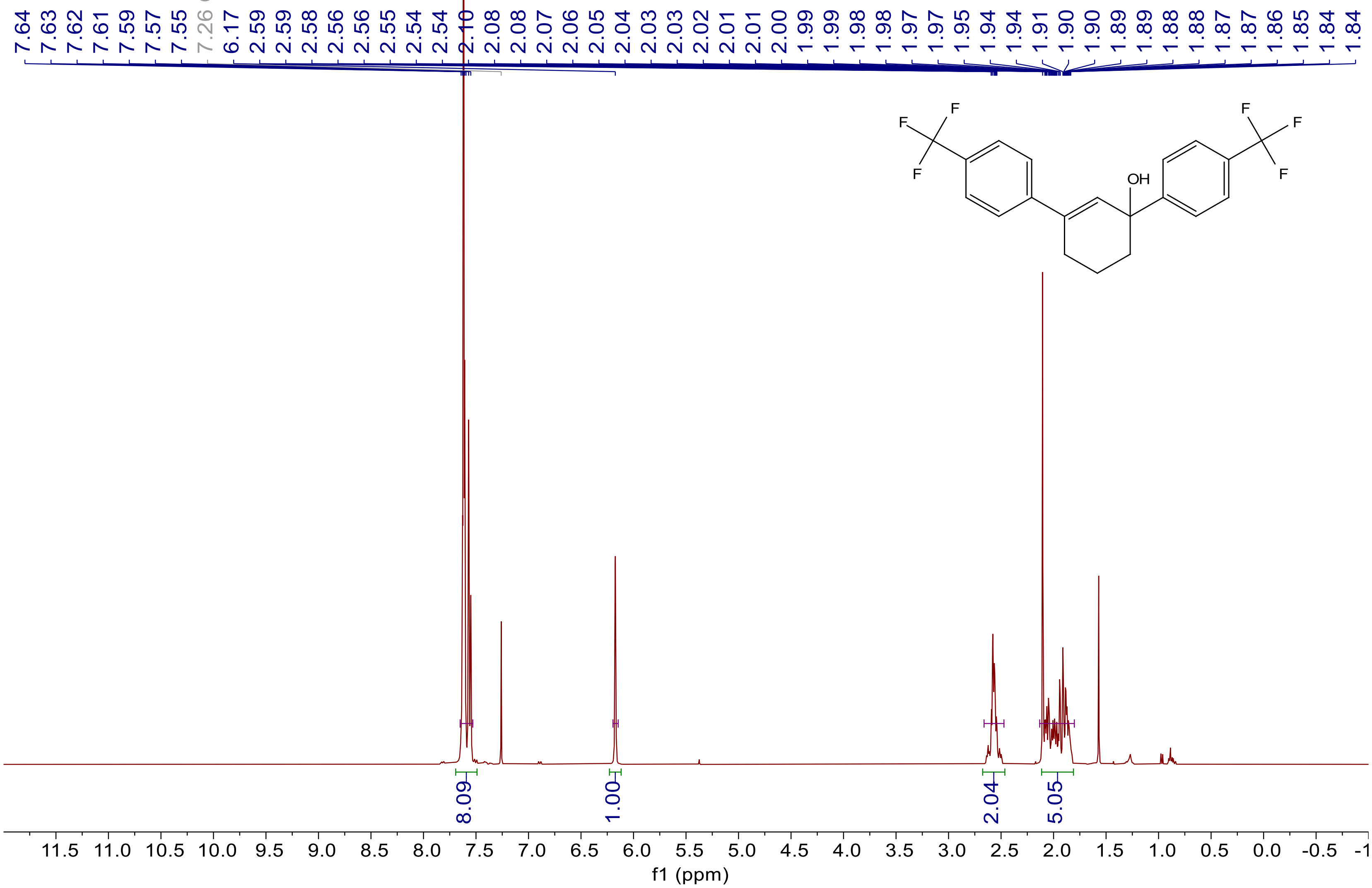

1a 4,4''-bis(trifluoromethyl)-5',6'-dihydro-[1,1':3',1''-terphenyl]-1'(4'H)-ol
19F NMR at 376.48 MHz in CDCl3

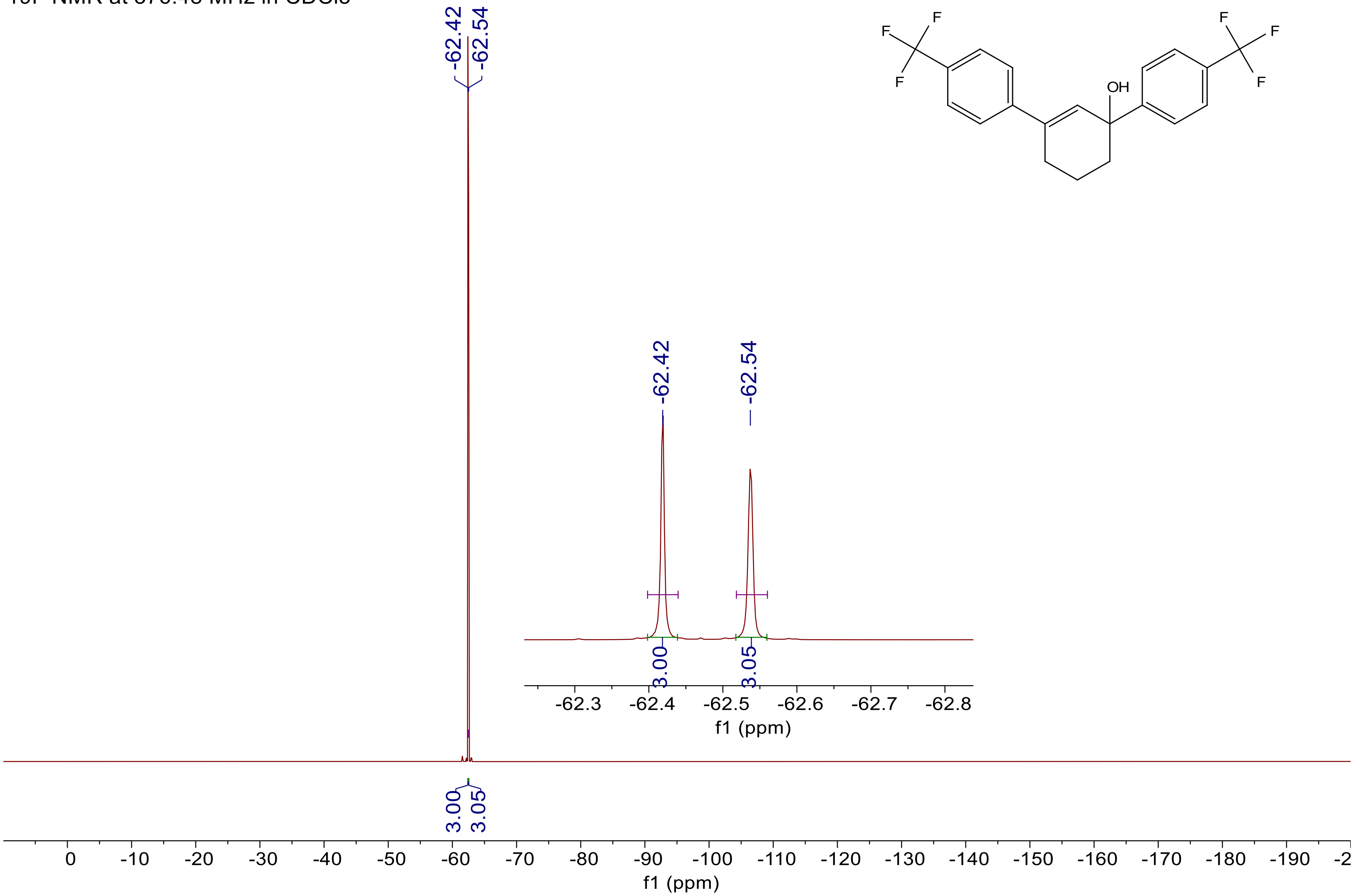

1a 4,4''-bis(trifluoromethyl)-5',6'-dihydro-[1,1':3',1''-terphenyl]-1'(4'H)-ol
13C NMR at 100.63 MHz in CDCl3

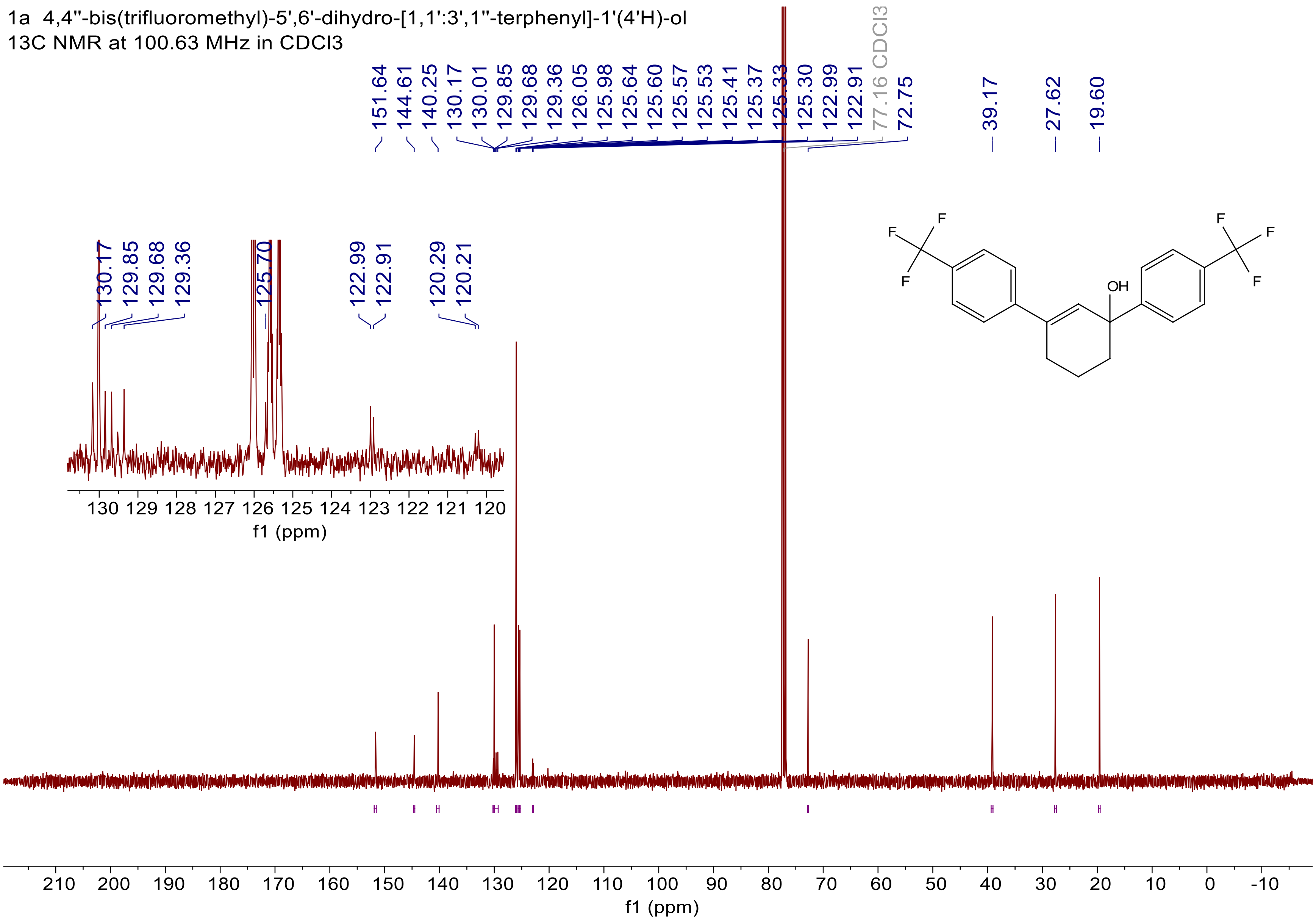

1a 4,4''-bis(trifluoromethyl)-5',6'-dihydro-[1,1':3',1''-terphenyl]-1'(4'H)-ol

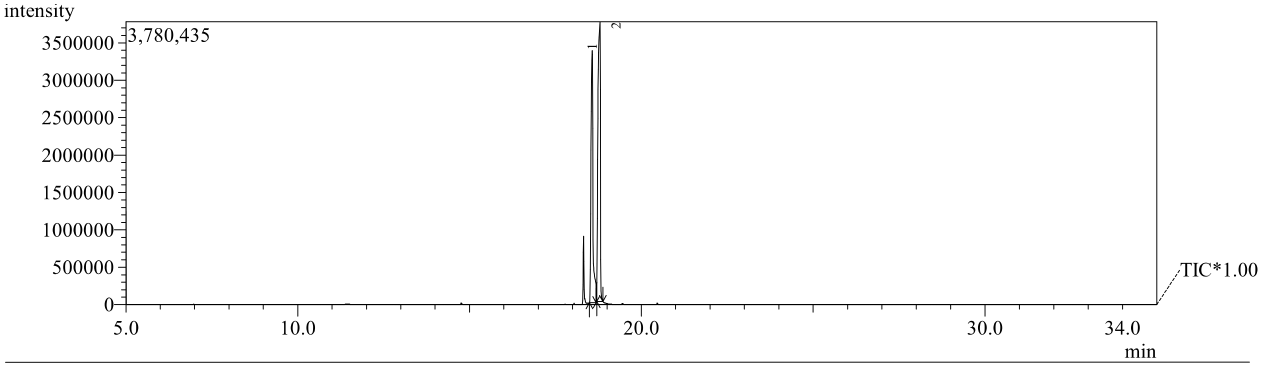

Spectrum

Line#:1 R.Time:18.6(Scan#:1629)
MassPeaks:235
RawMode:Averaged 18.6-18.6(1628-1630) BasePeak:368(612437)
BG Mode:Calc. from Peak

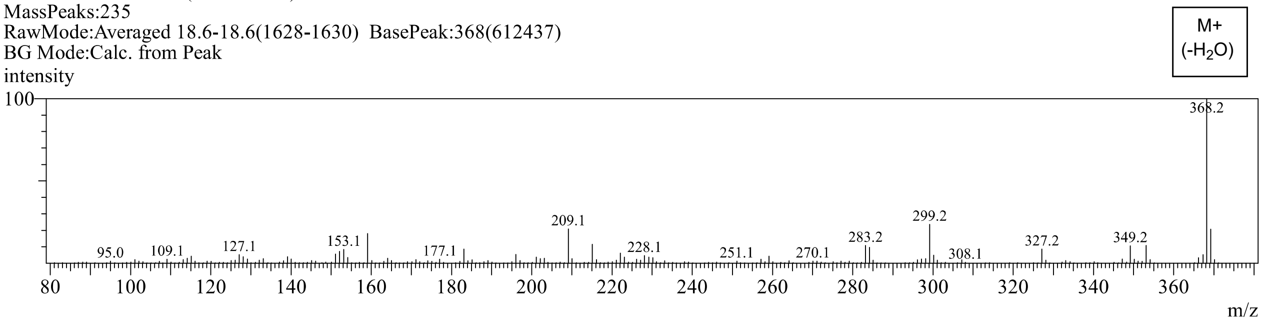

Line#:2 R.Time:18.8(Scan#:1657)
MassPeaks:257
RawMode:Averaged 18.8-18.8(1656-1658) BasePeak:289(199376)
BG Mode:Calc. from Peak

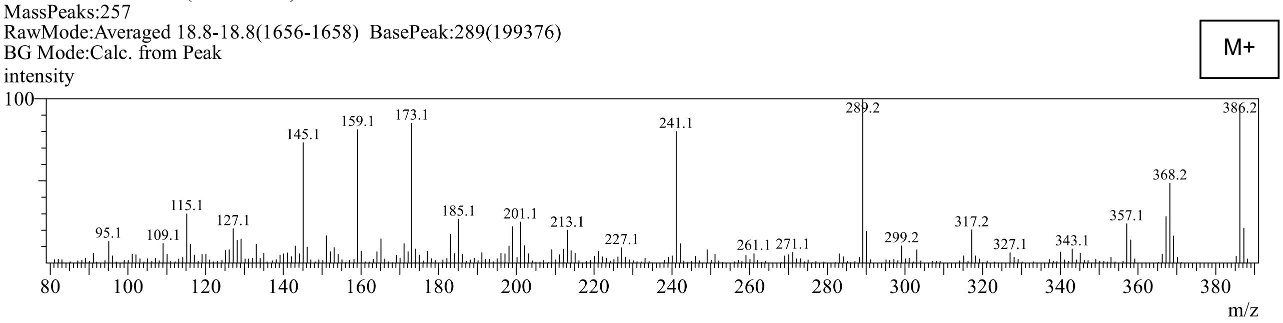

1b 4''-(trifluoromethyl)-5',6'-dihydro-[1,1':3',1''-terphenyl]-1'(4'H)-ol
1H NMR at 400.15 MHz in CD2Cl2

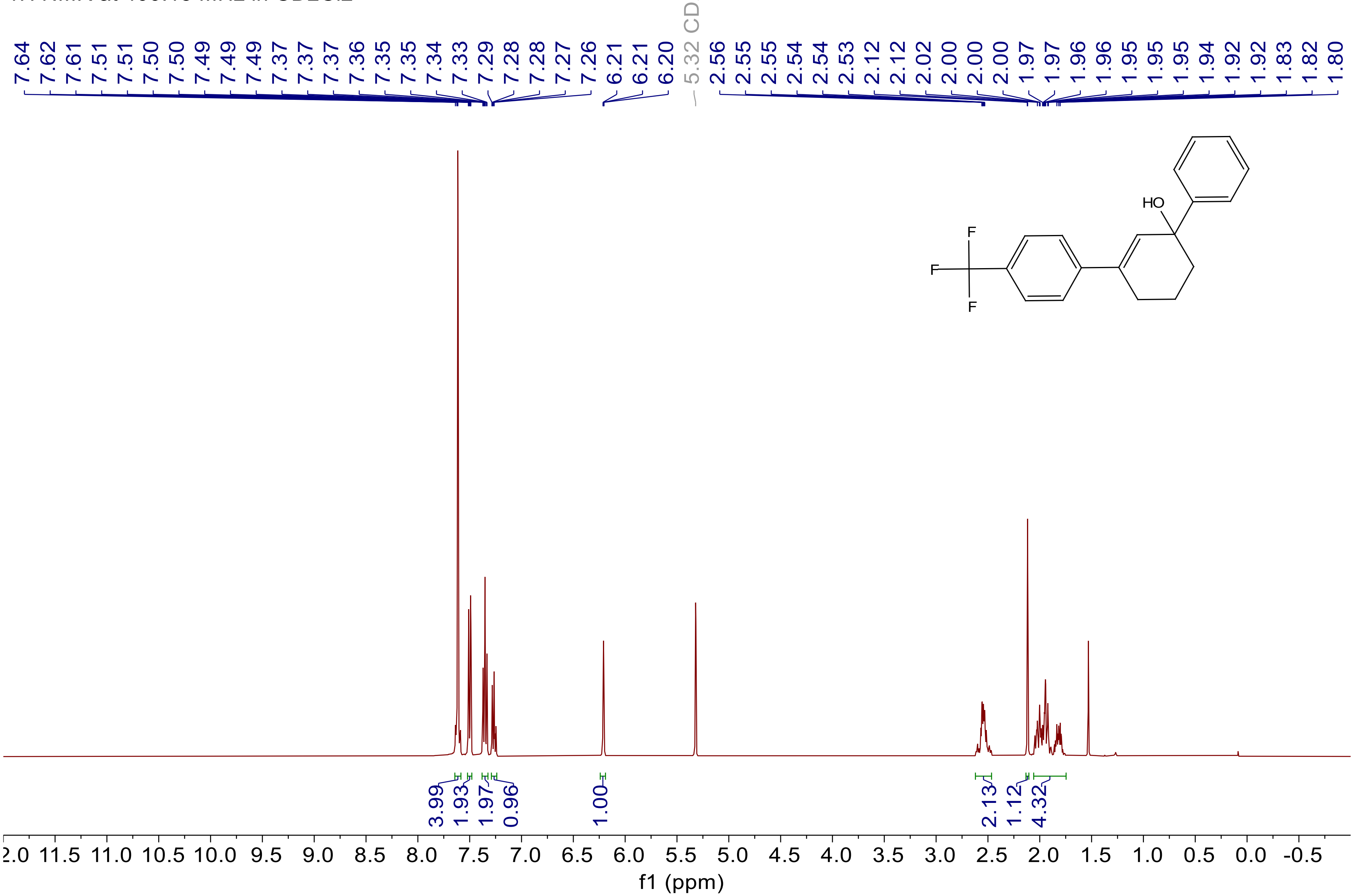

1b 4''-(trifluoromethyl)-5',6'-dihydro-[1,1':3',1''-terphenyl]-1'(4'H)-ol
19F NMR at 376.48 MHz in CD2Cl2

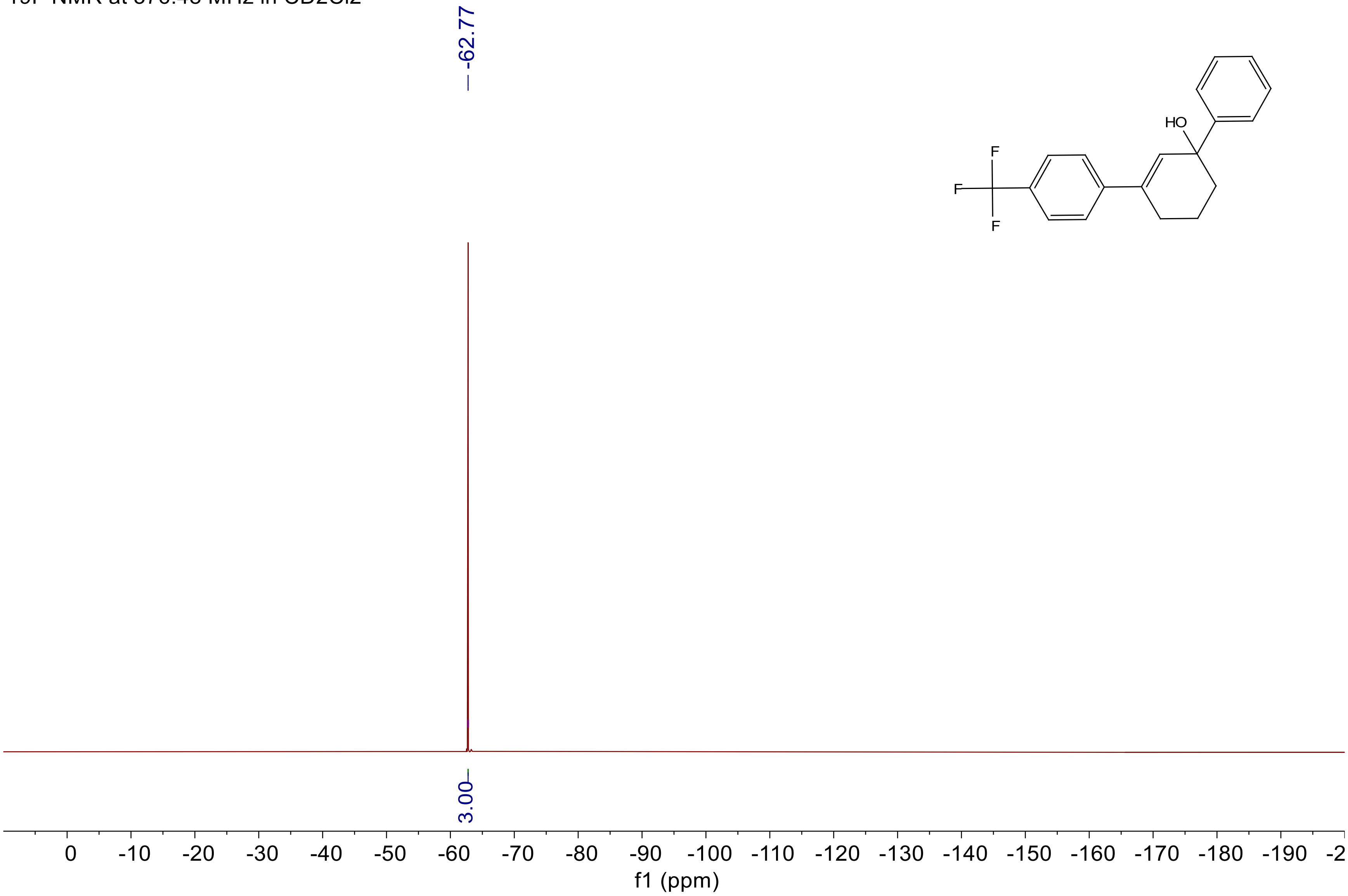

1b 4''-(trifluoromethyl)-5',6'-dihydro-[1,1':3',1''-terphenyl]-1'(4'H)-ol
13C NMR at 100.63 MHz in CD2Cl2

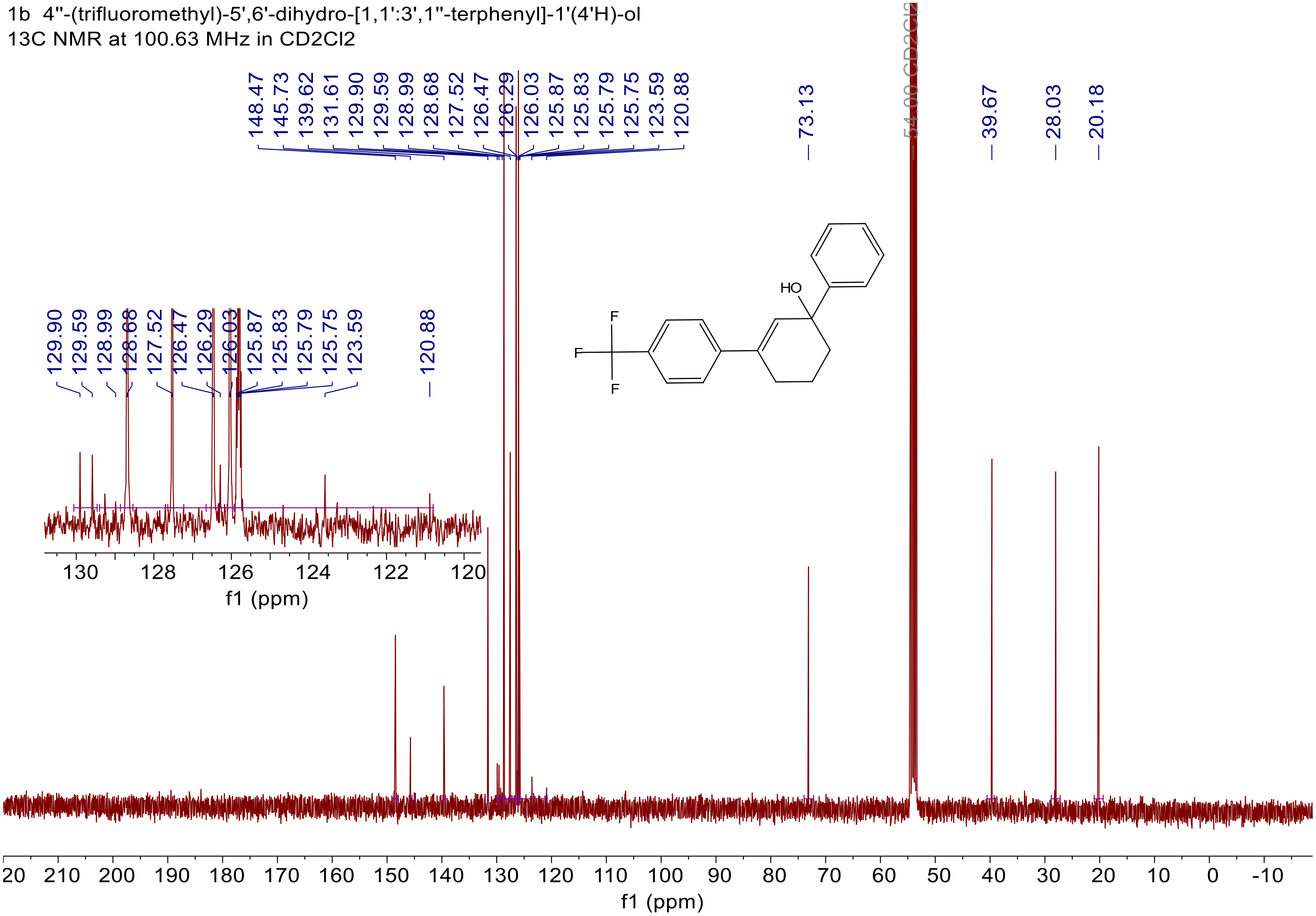

1c 4-(trifluoromethyl)-5',6'-dihydro-[1,1':3',1''-terphenyl]-1'(4'H)-ol
1H NMR at 400.15 MHz in CD2Cl2

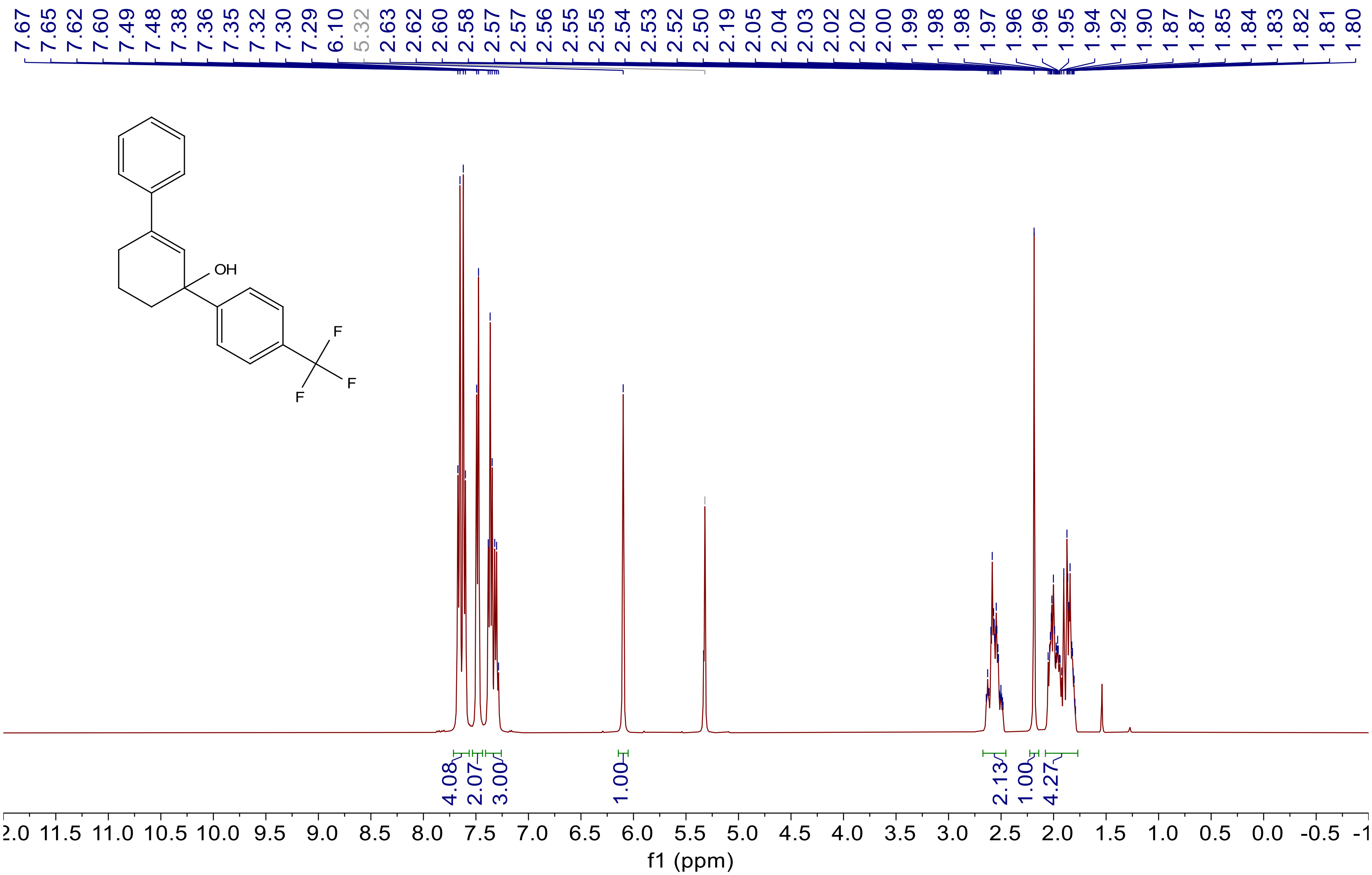

1c 4-(trifluoromethyl)-5',6'-dihydro-[1,1':3',1''-terphenyl]-1'(4'H)-ol
19F NMR at 376.48 MHz in CD2Cl2

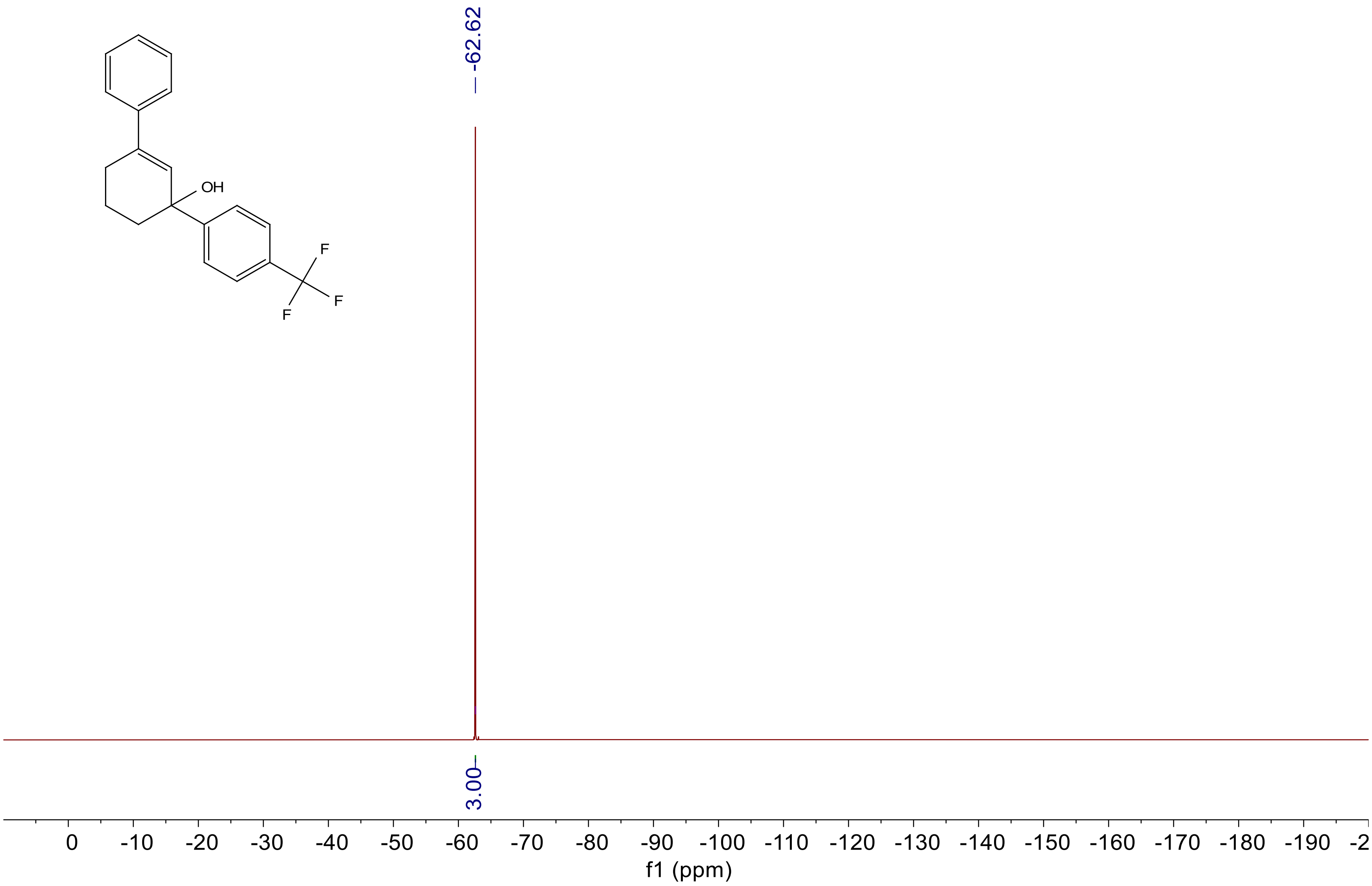

1c 4-(trifluoromethyl)-5',6'-dihydro-[1,1':3',1''-terphenyl]-1'(4'H)-ol
13C NMR at 100.63 MHz in CD2Cl2

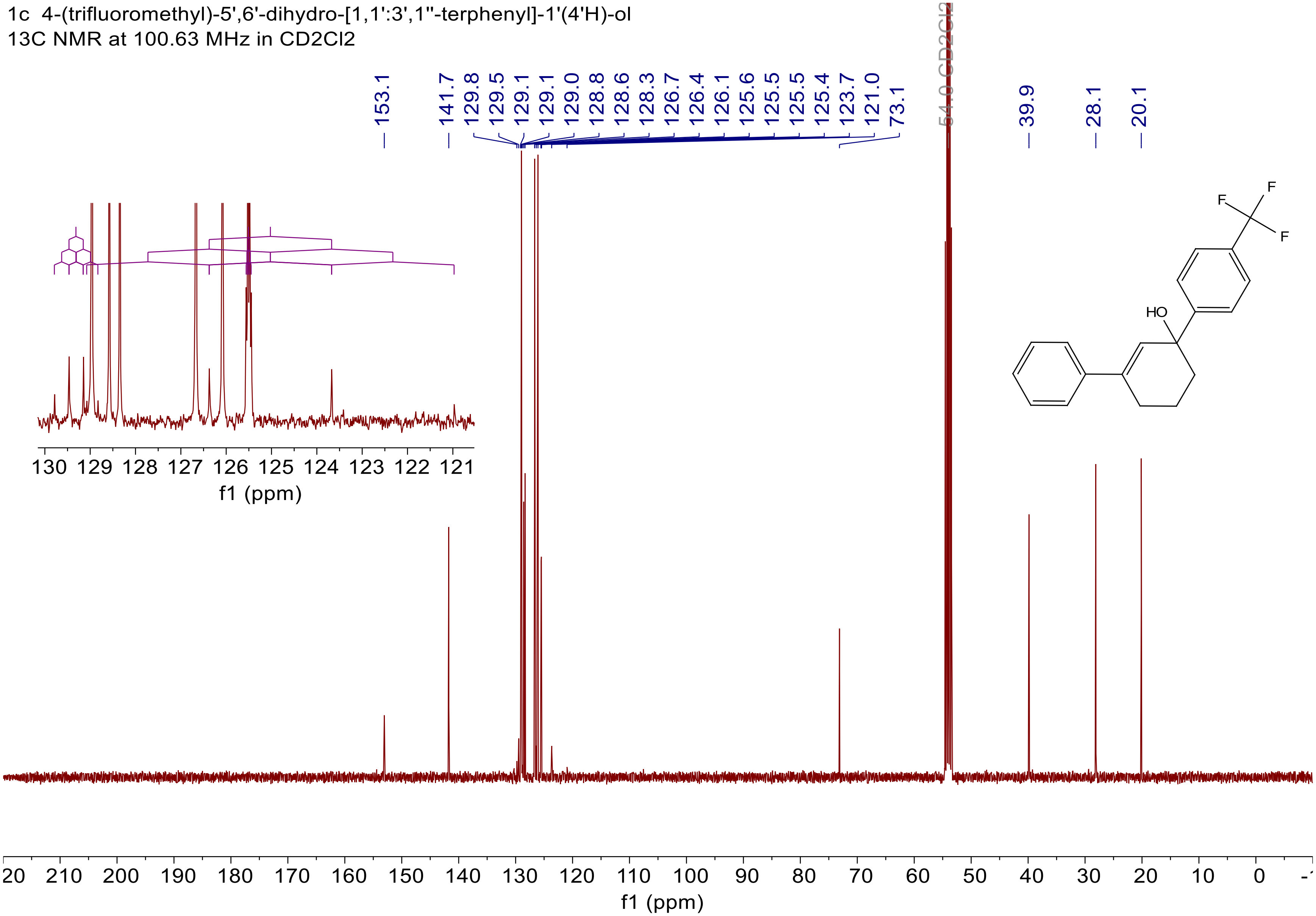

1c 4-(trifluoromethyl)-5',6'-dihydro-[1,1':3',1''-terphenyl]-1'(4'H)-ol

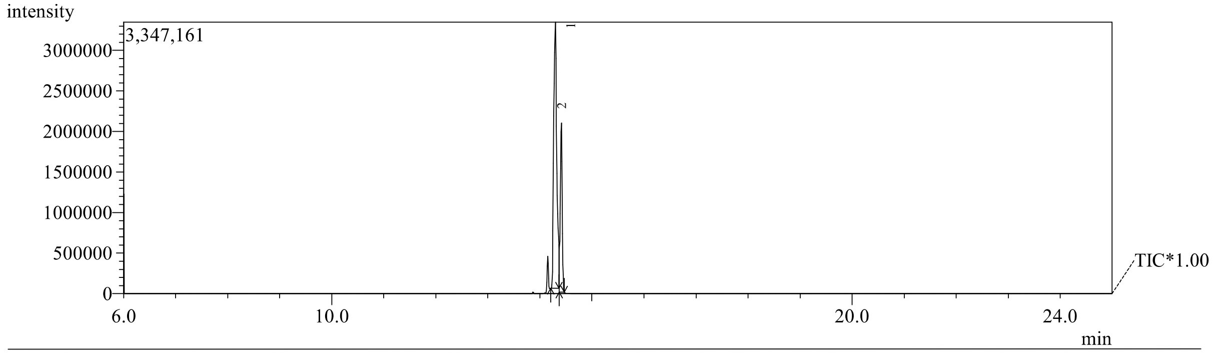

Spectrum

Line#:1 R.Time:14.3(Scan#:997)
MassPeaks:171
RawMode:Averaged 14.3-14.3(996-998) BasePeak:300(542882)
BG Mode:Calc. from Peak

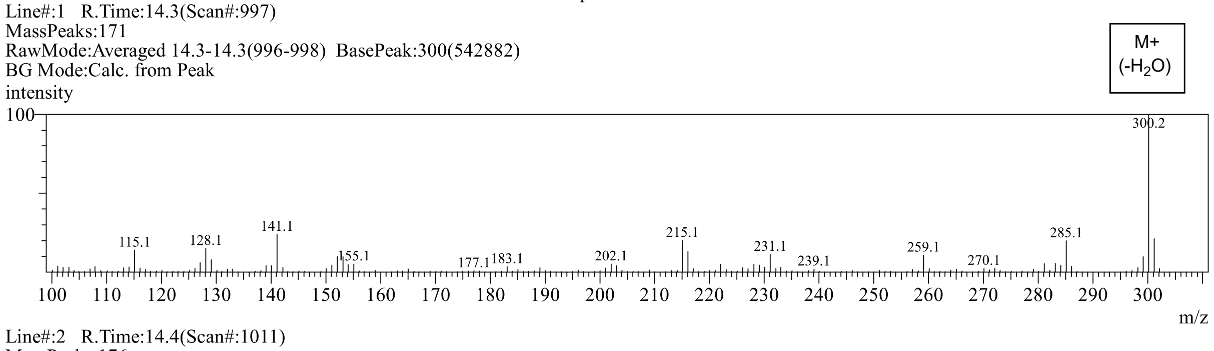

Line#:2 R.Time:14.4(Scan#:1011)
MassPeaks:176
RawMode:Averaged 14.4-14.4(1010-1012) BasePeak:318(99716)
BG Mode:Calc. from Peak

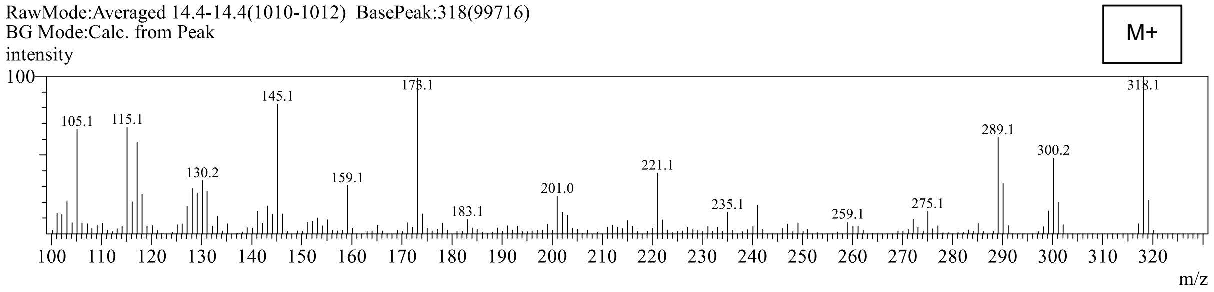

1d 5',6'-dihydro-[1,1':3',1''-terphenyl]-1'(4'H)-ol
1H NMR at 400.15 MHz in CDCl3

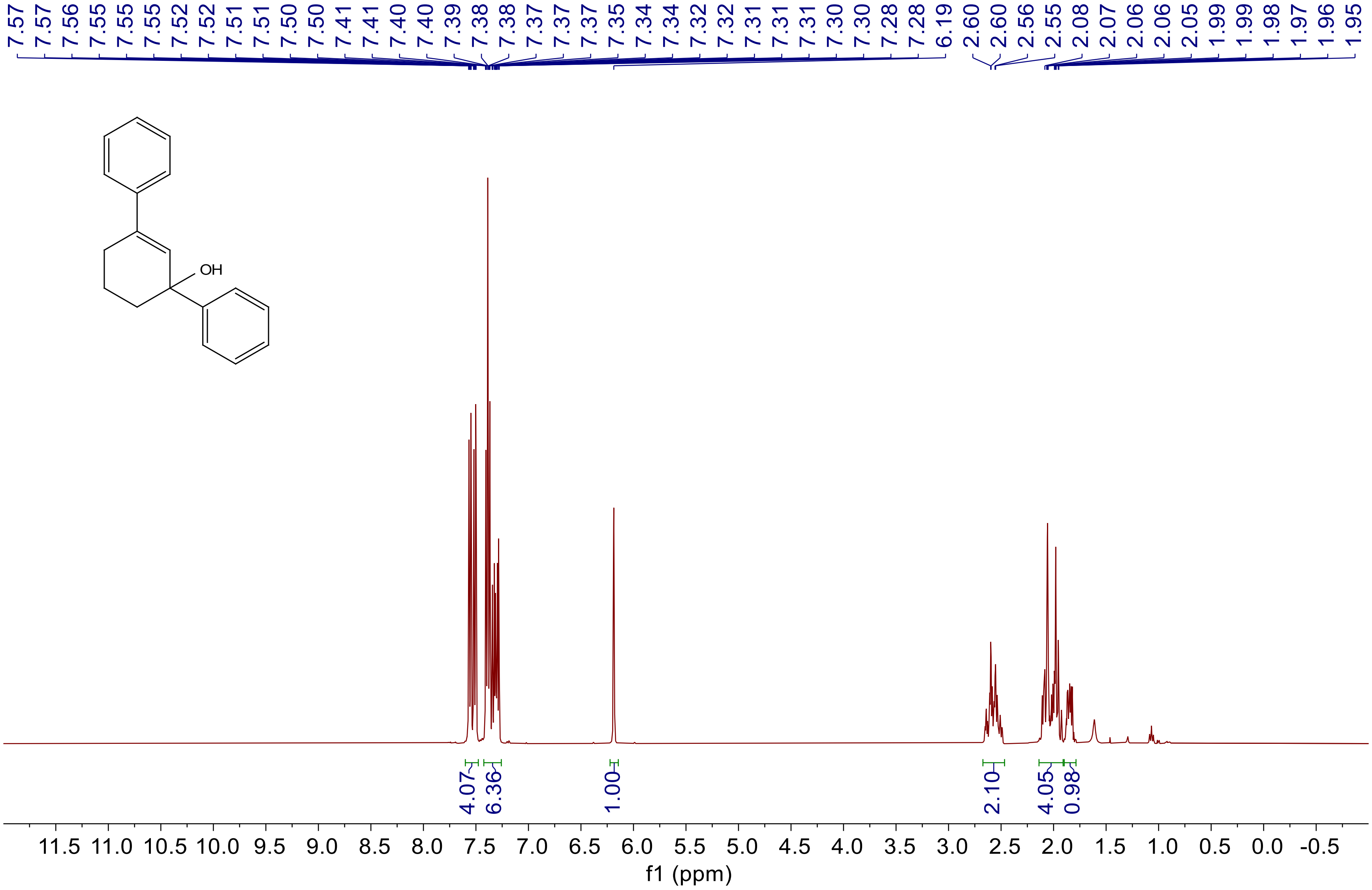

1d 5',6'-dihydro-[1,1':3',1''-terphenyl]-1'(4'H)-ol
13C NMR at 100.63 MHz in CDCl3

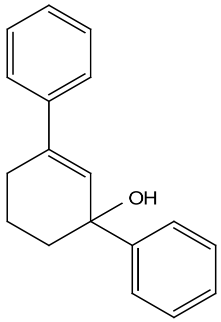

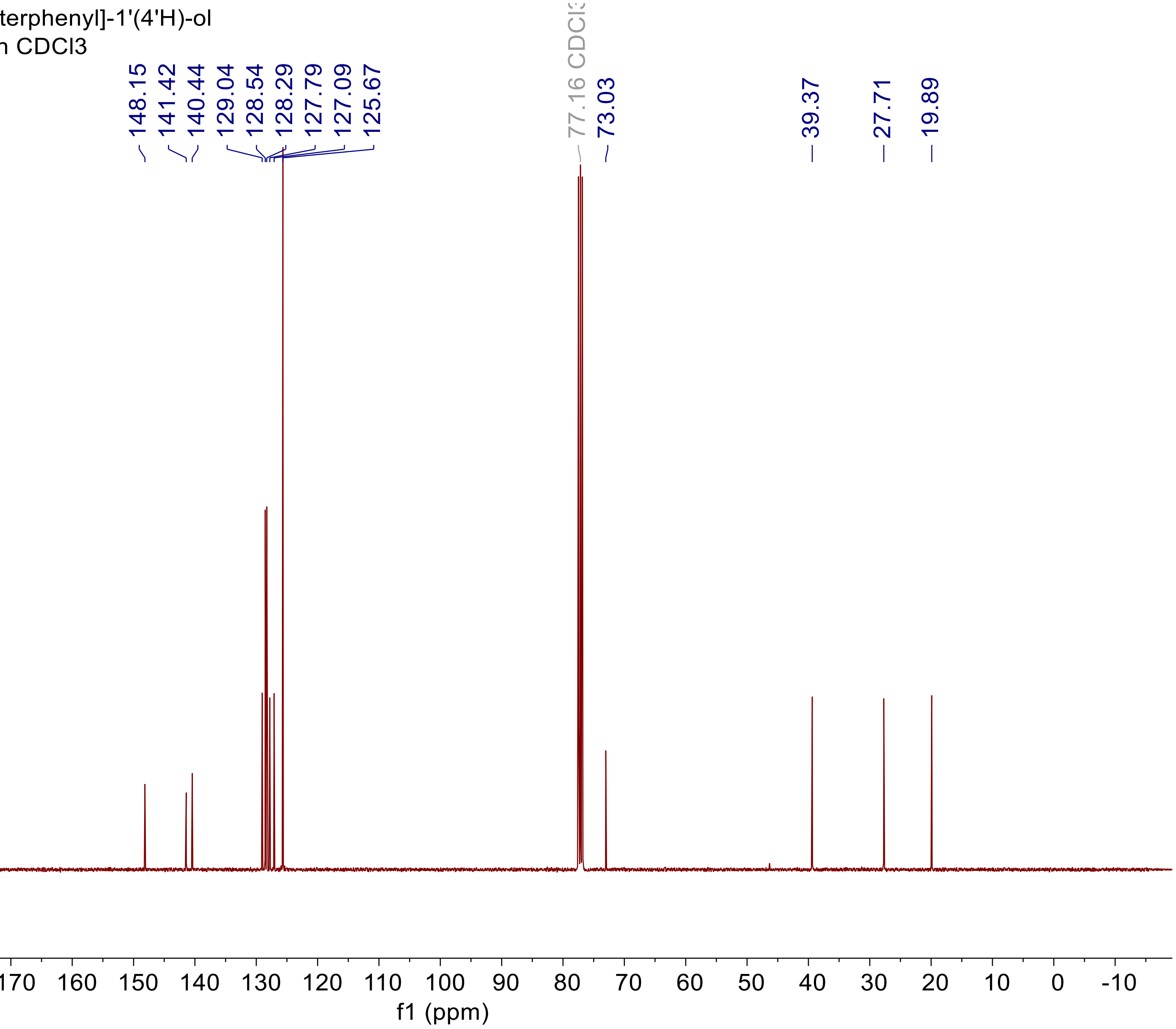

1e 3-methyl-3,4,5,6-tetrahydro-[1,1'-biphenyl]-3-ol
1H NMR at 400.15 MHz in CD2Cl2

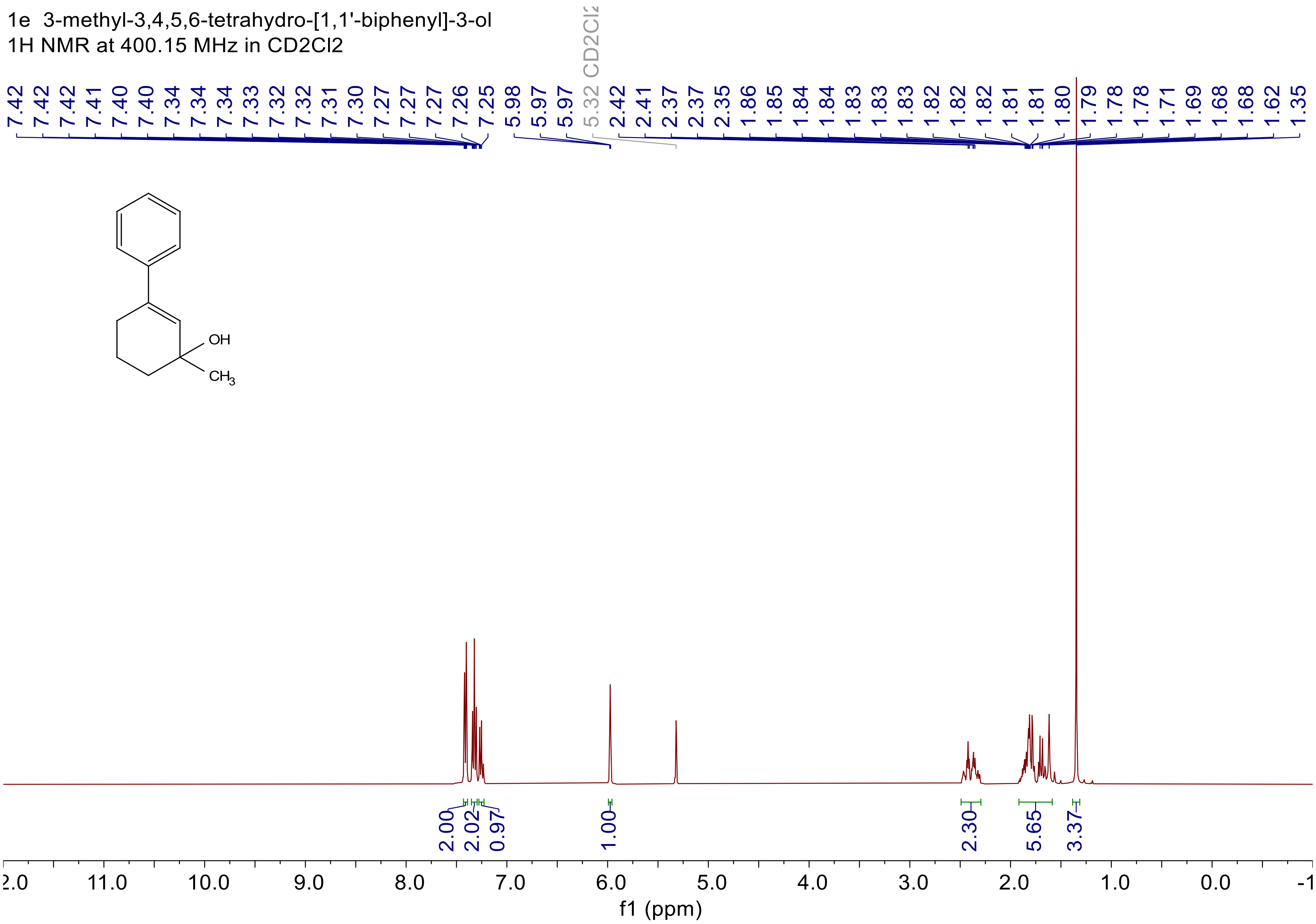

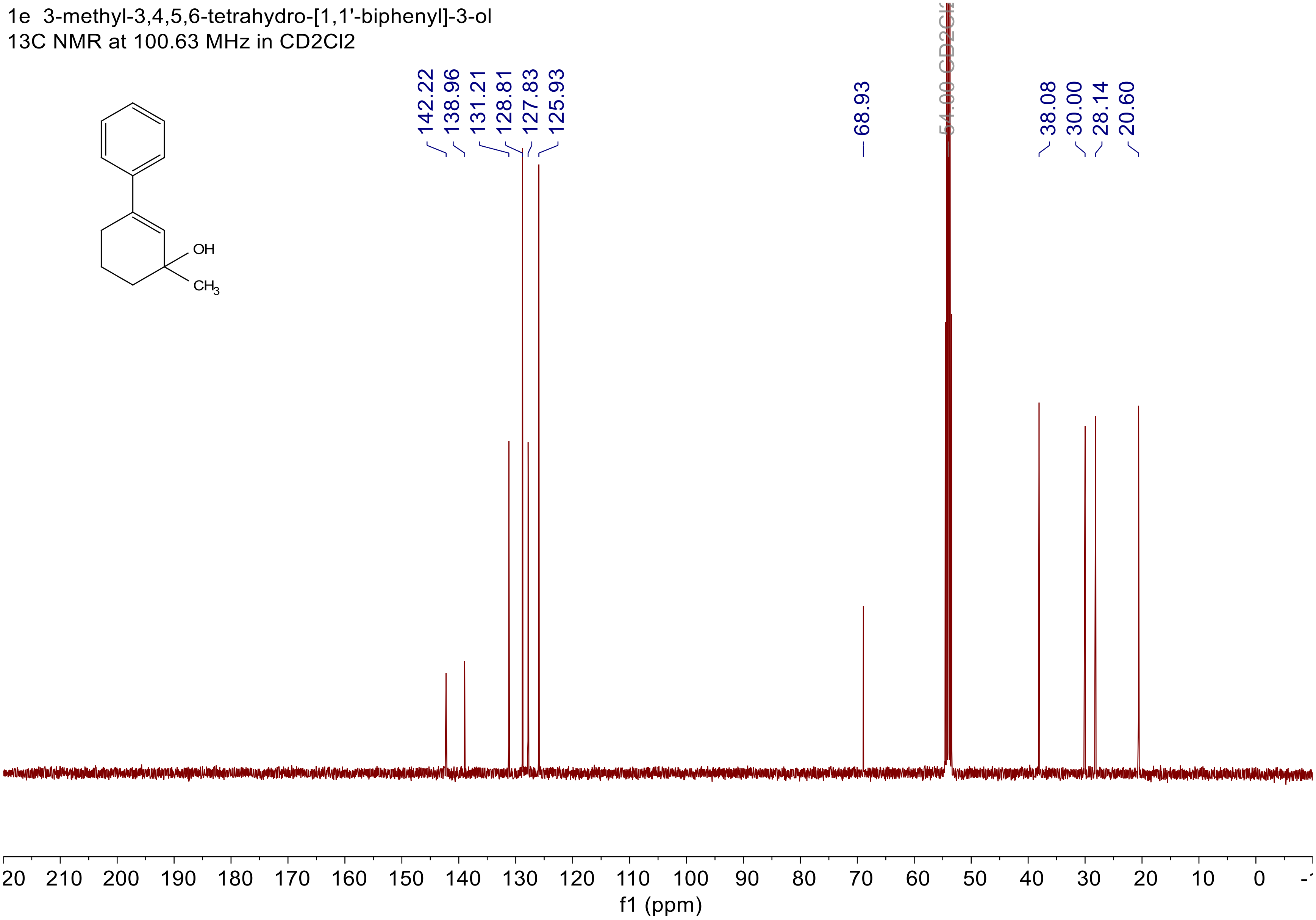
1e 3-methyl-3,4,5,6-tetrahydro-[1,1'-biphenyl]-3-ol
13C NMR at 100.63 MHz in CD2Cl2
OH
CH3
142.22
138.96
131.21
128.81
127.83
125.93
68.93
54.00 CD2Cl2
38.08
30.00
28.14
20.60
20
210
200
190
180
170
160
150
140
130
120
110
100
90
80
70
60
50
40
30
20
10
0
f1 (ppm)

1f 3-allyl-4'-(trifluoromethyl)-3,4,5,6-tetrahydro-[1,1'-biphenyl]-3-ol
1H NMR at 400.15 MHz in CDCl3

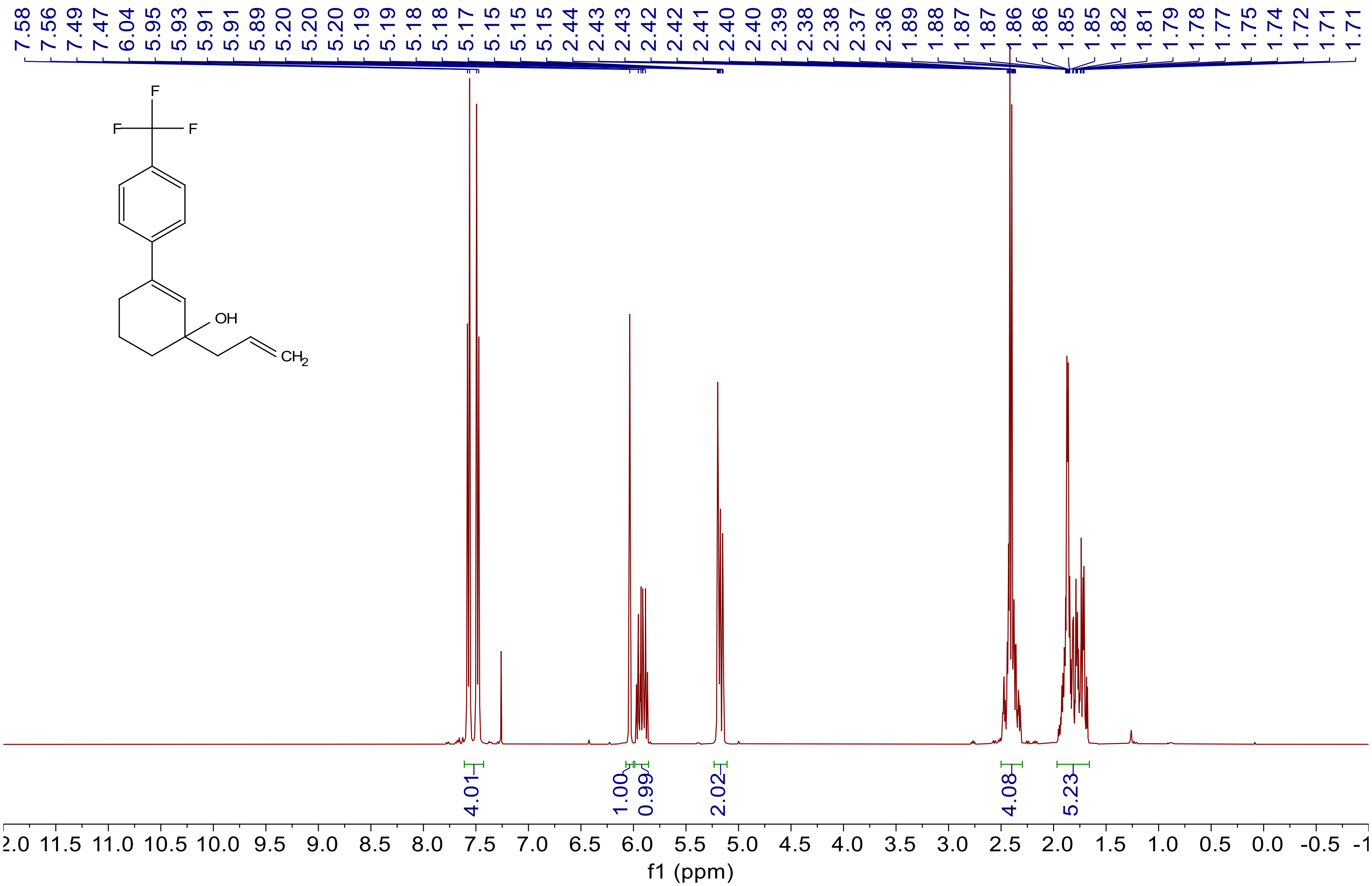

1f 3-allyl-4'-(trifluoromethyl)-3,4,5,6-tetrahydro-[1,1'-biphenyl]-3-ol
19F NMR at 376.48 MHz in CDCl3

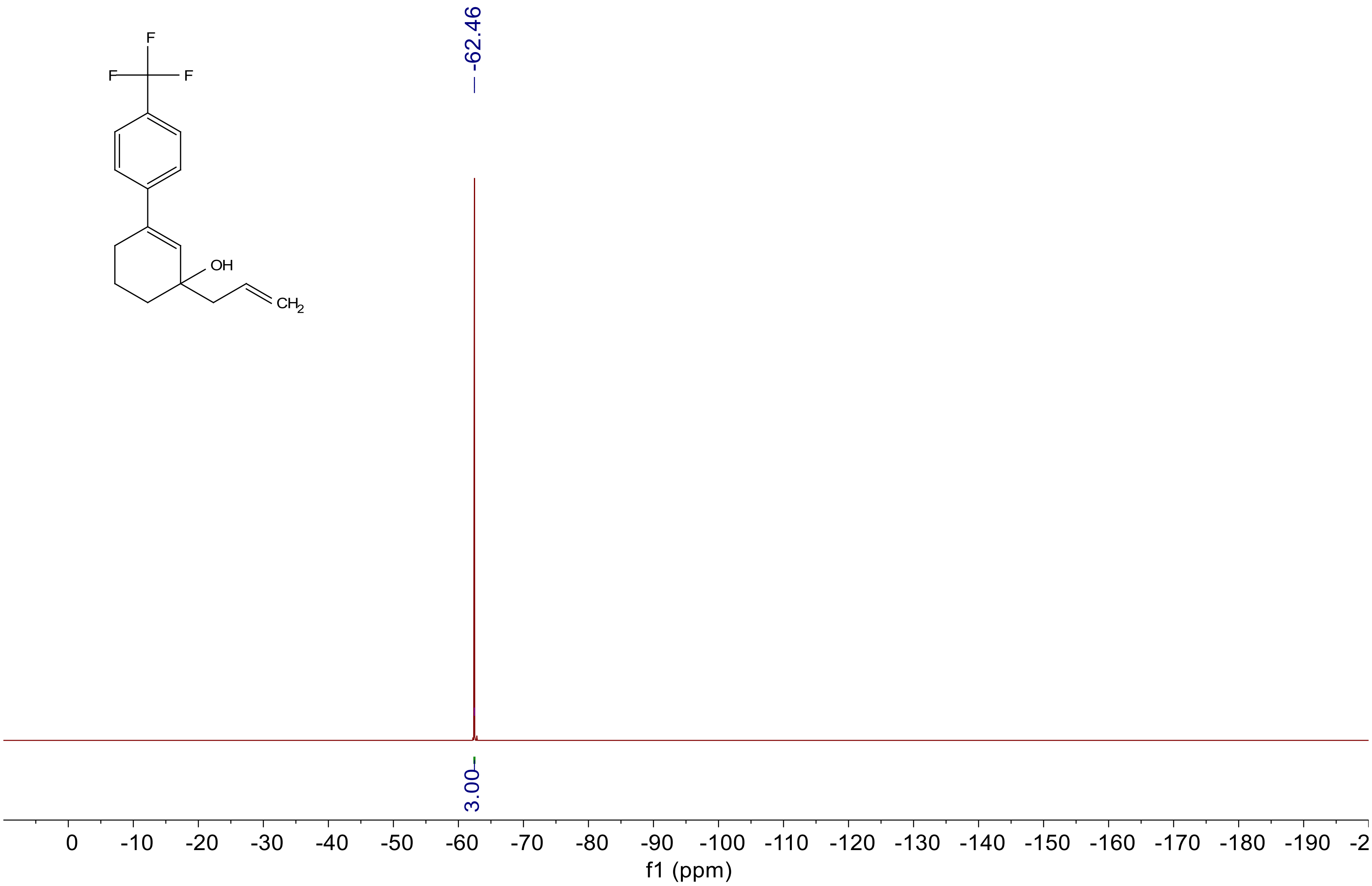

1f 3-allyl-4'-(trifluoromethyl)-3,4,5,6-tetrahydro-[1,1'-biphenyl]-3-ol

13C NMR at 100.63 MHz in $CDCl_3$

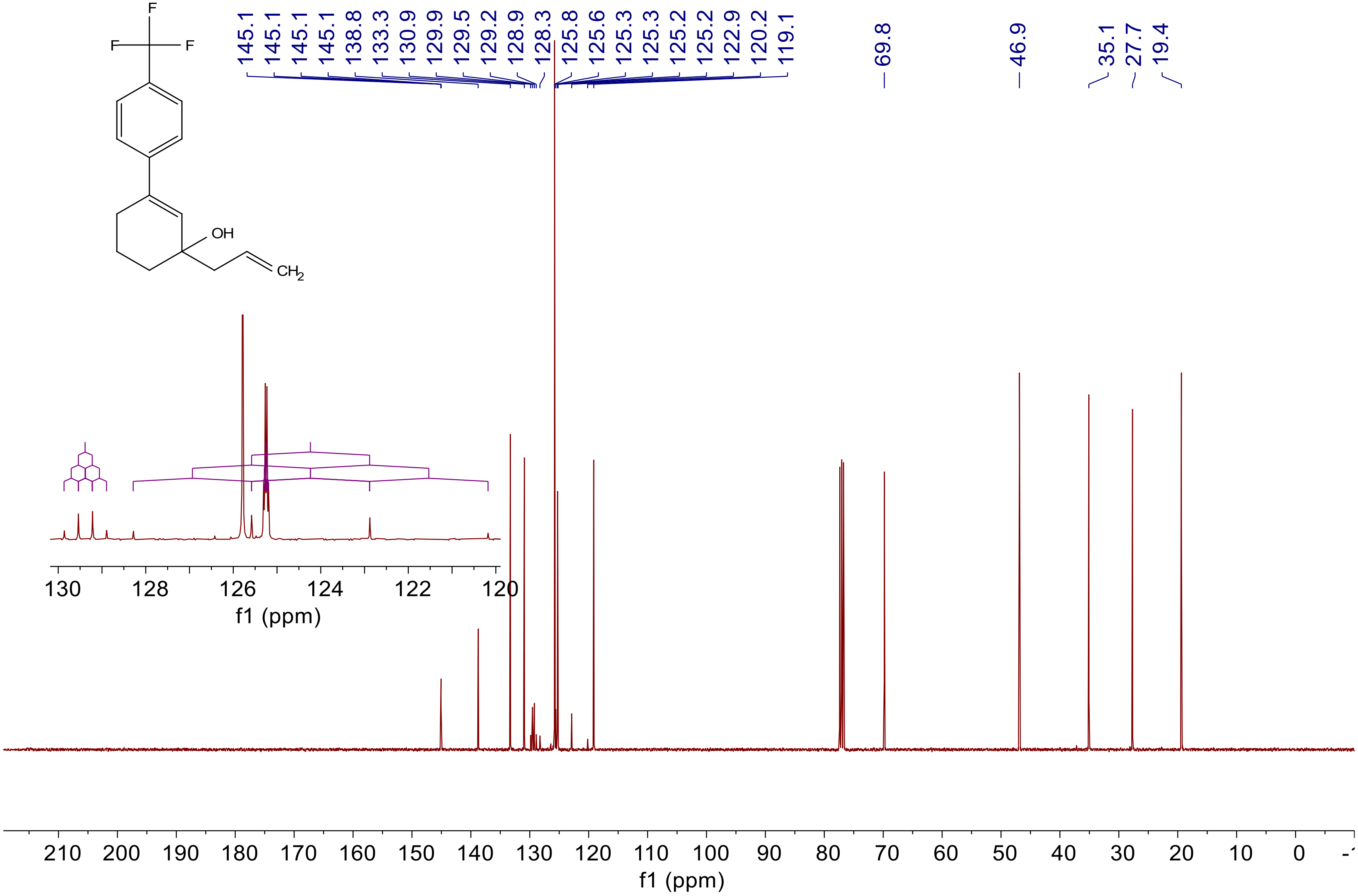

1g 3-(3-(dimethylamino)prop-1-yn-1-yl)-4'-(trifluoromethyl)-3,4,5,6-tetrahydro-[1,1'-biphenyl]-3-ol
1H NMR at 400.15 MHz in CD2Cl2

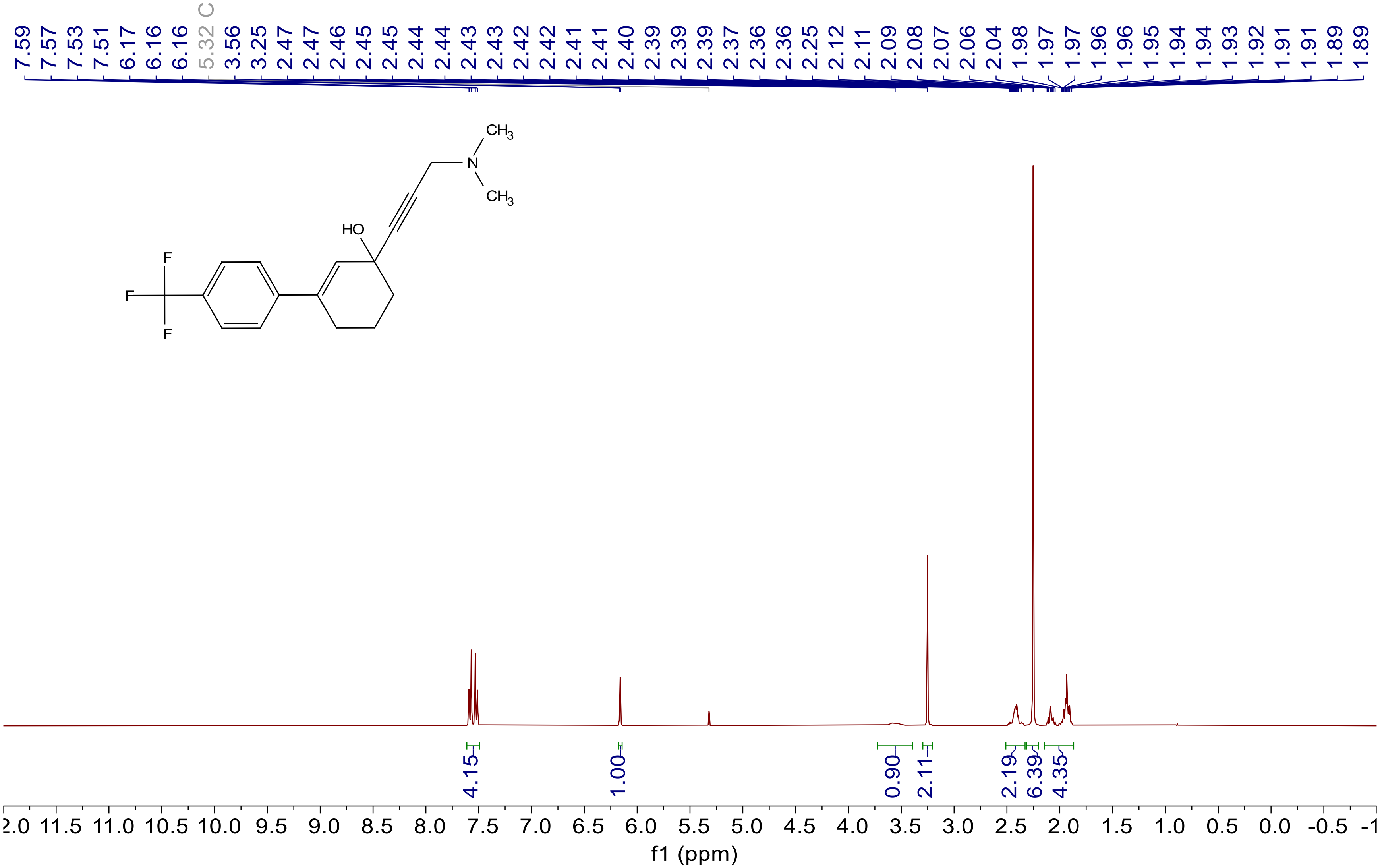

1g 3-(3-(dimethylamino)prop-1-yn-1-yl)-4'-(trifluoromethyl)-3,4,5,6-tetrahydro-[1,1'-biphenyl]-3-ol
19F NMR at 376.48 MHz in CD2Cl2

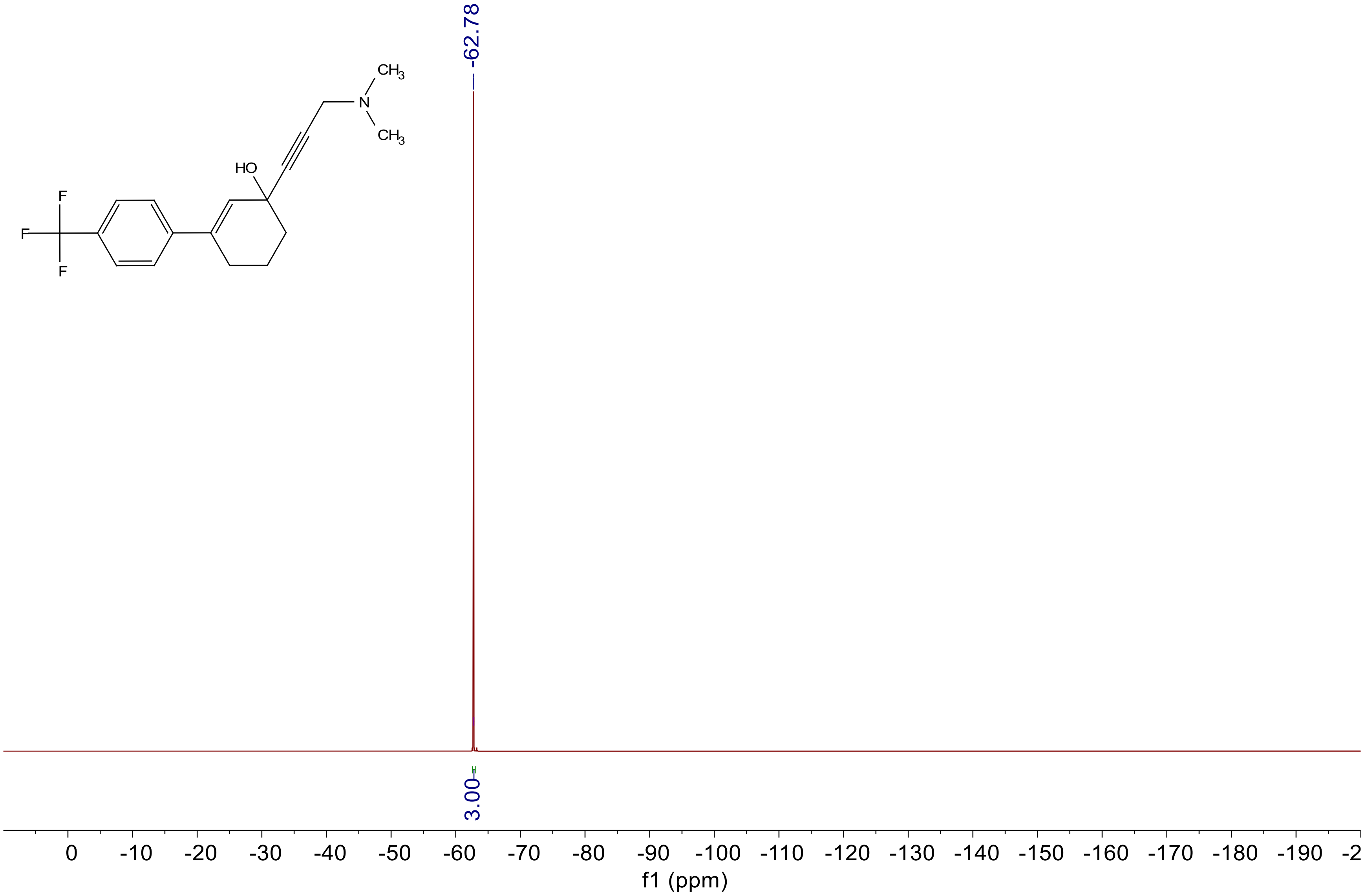

1g 3-(3-(dimethylamino)prop-1-yn-1-yl)-4'-(trifluoromethyl)-3,4,5,6-tetrahydro-[1,1'-biphenyl]-3-ol

13C NMR at 100.63 MHz in CD2Cl2

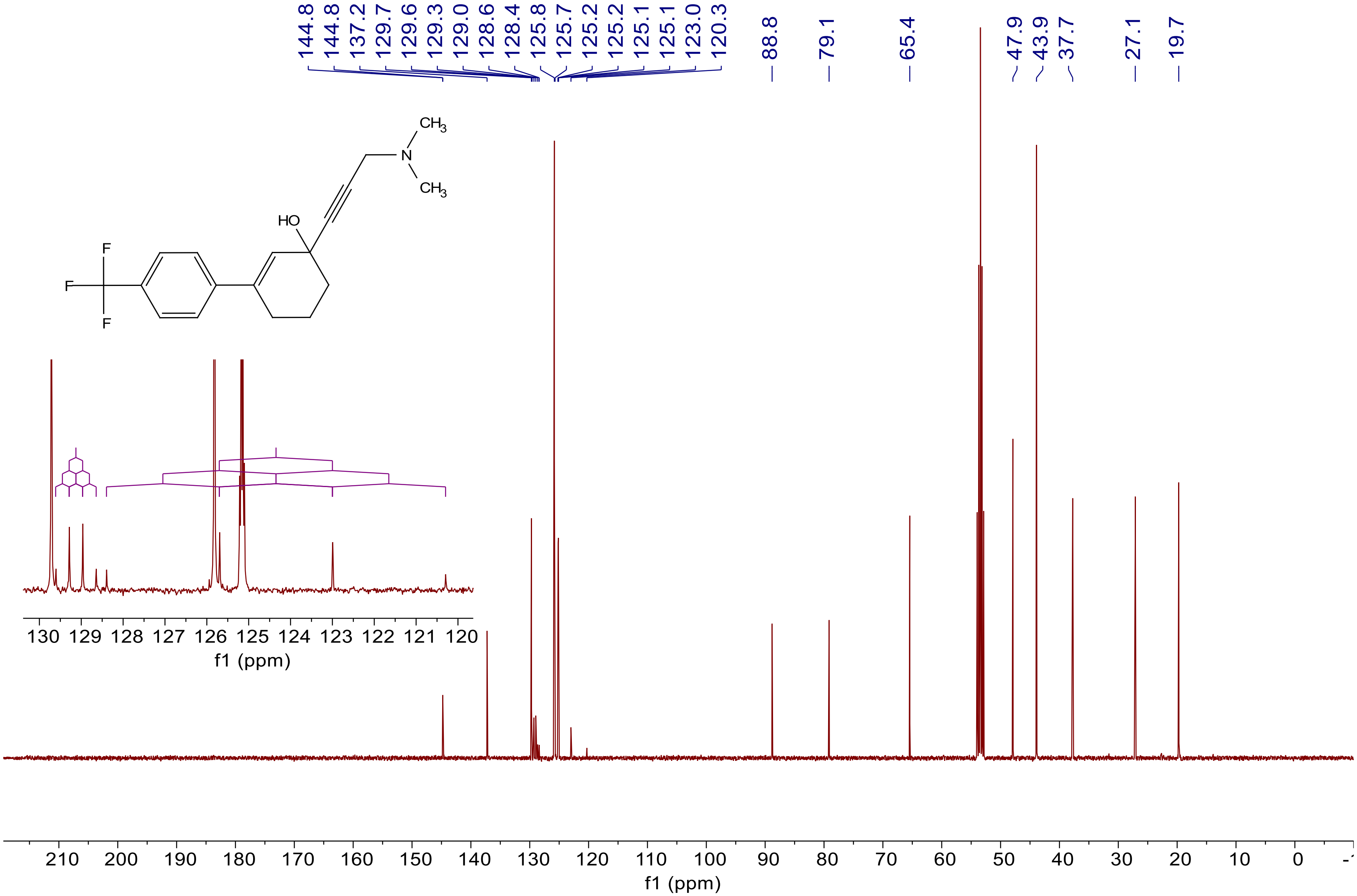

1g 3-(3-(dimethylamino)prop-1-yn-1-yl)-4'-(trifluoromethyl)-3,4,5,6-tetrahydro-[1,1'-biphenyl]-3-ol

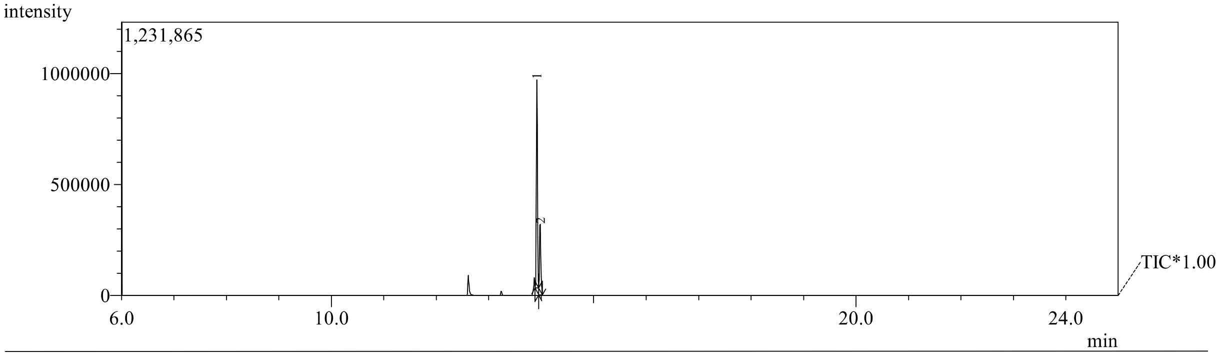

Spectrum

Line#:1 R.Time:13.9(Scan#:951)
MassPeaks:174
RawMode:Averaged 13.9-13.9(950-952) BasePeak:250(28515)
BG Mode:Calc. from Peak

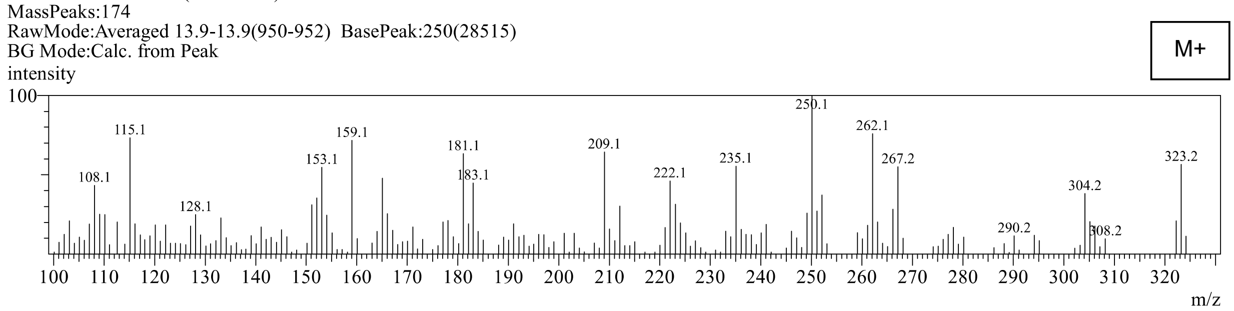

Line#:2 R.Time:14.0(Scan#:959)
MassPeaks:100
RawMode:Averaged 14.0-14.0(958-960) BasePeak:305(16380)
BG Mode:Calc. from Peak

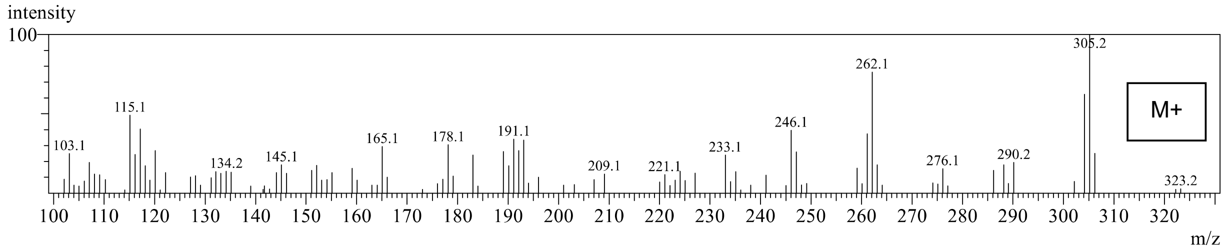

1h 3-(prop-1-yn-1-yl)-3,4,5,6-tetrahydro-[1,1'-biphenyl]-3-ol
1H NMR at 400.15 MHz in CD2Cl2

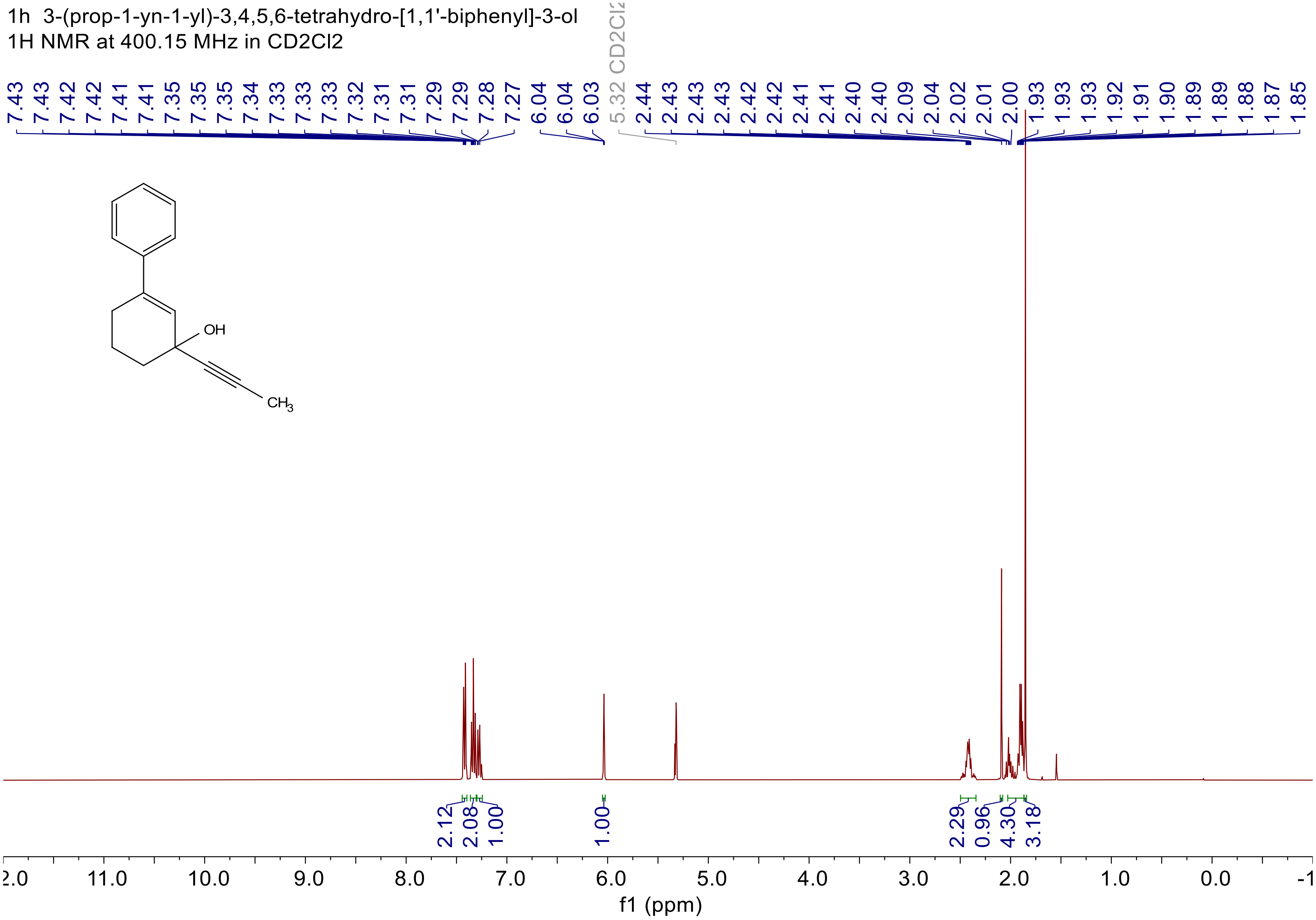

E22

1h 3-(prop-1-yn-1-yl)-3,4,5,6-tetrahydro-[1,1'-biphenyl]-3-ol
13C NMR at 100.63 MHz in CD2Cl2

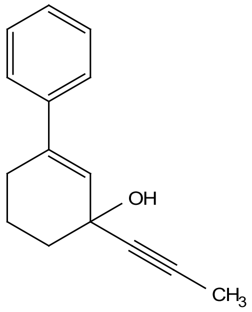

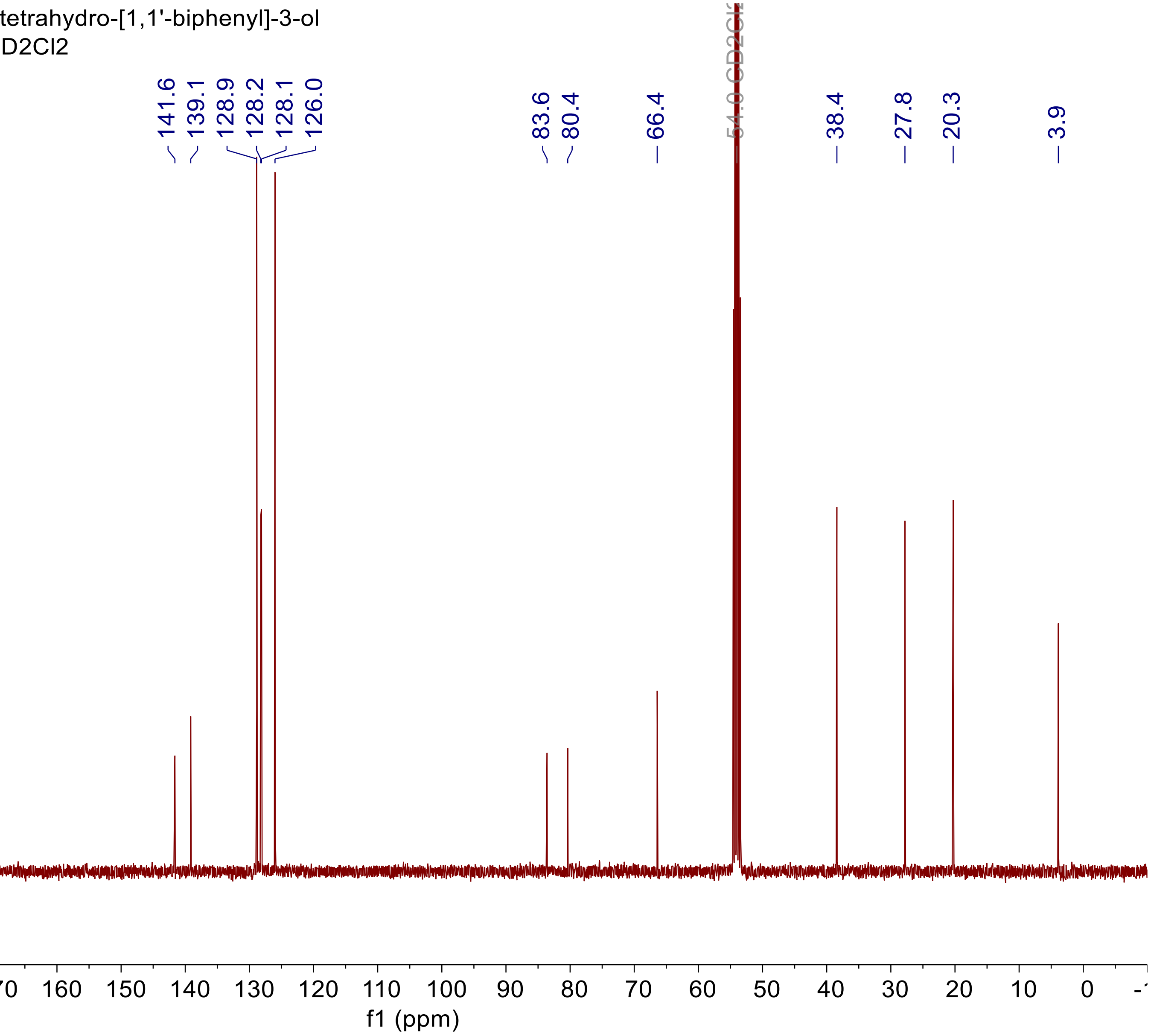

1i 3-ethynyl-4'-(trifluoromethyl)-3,4,5,6-tetrahydro-[1,1'-biphenyl]-3-ol
1H NMR at 400.15 MHz in CD2Cl2

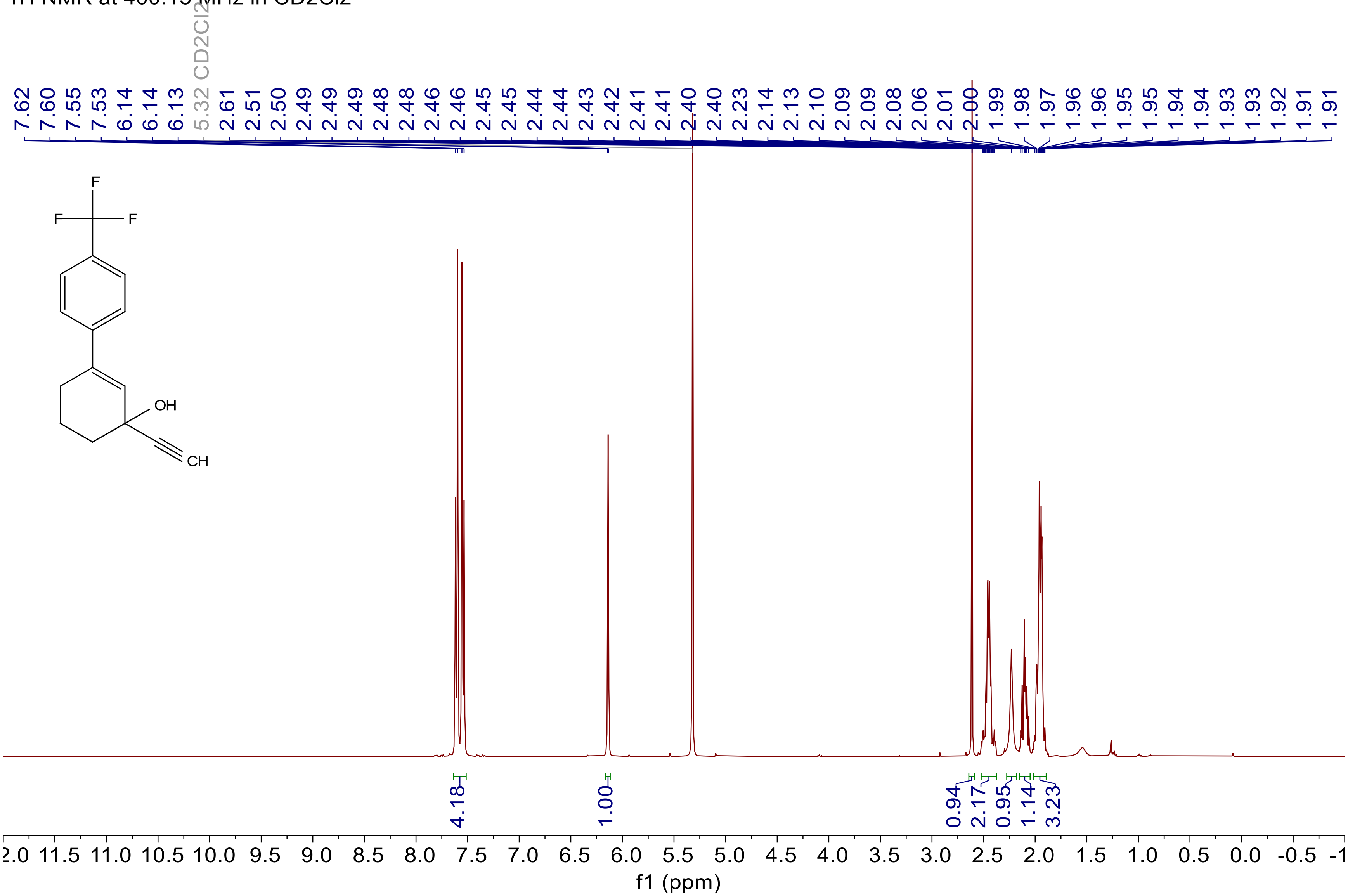

1i 3-ethynyl-4'-(trifluoromethyl)-3,4,5,6-tetrahydro-[1,1'-biphenyl]-3-ol
19F NMR at 376.48 MHz in CD2Cl2

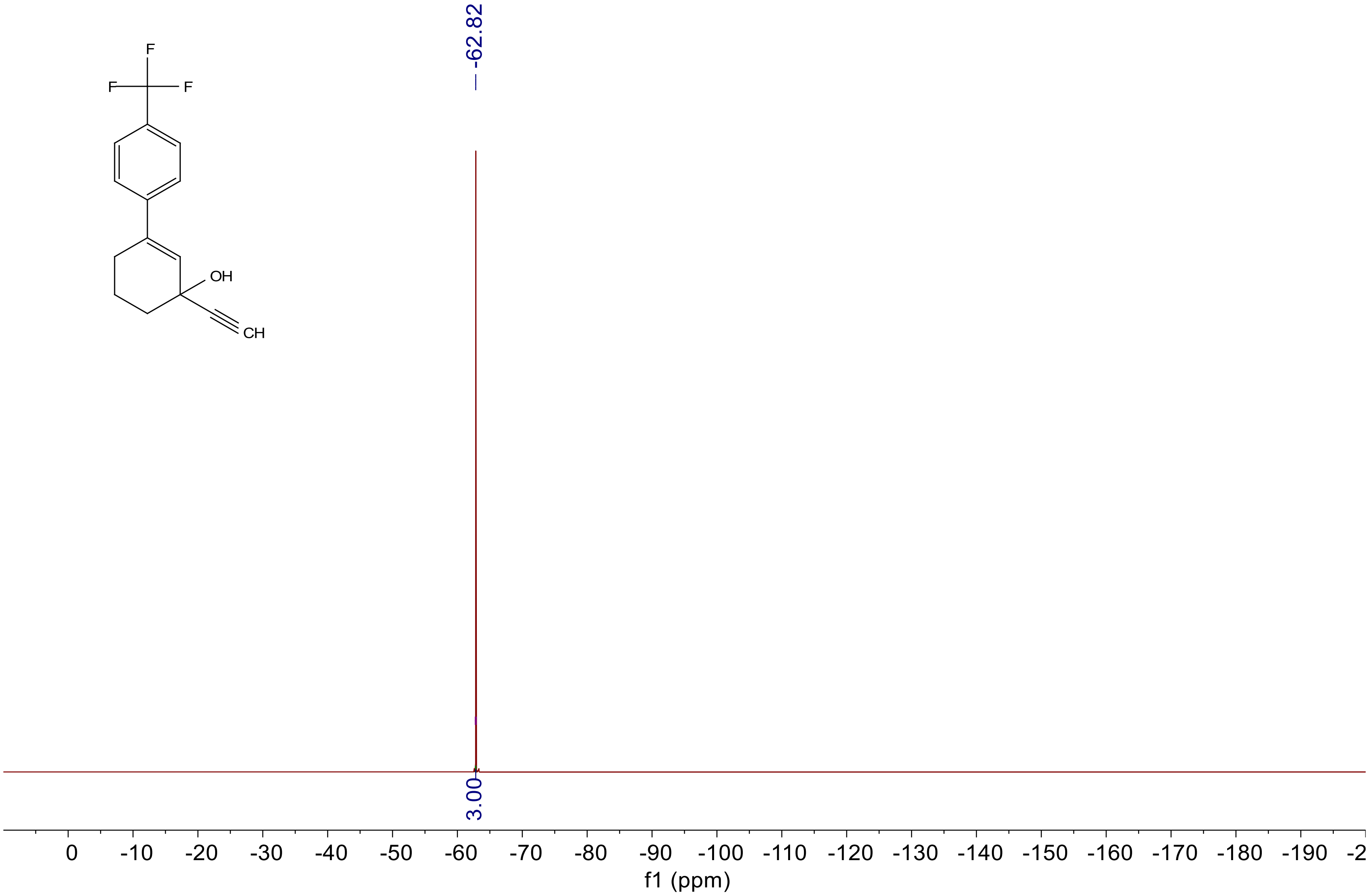

1i 3-ethynyl-4'-(trifluoromethyl)-3,4,5,6-tetrahydro-[1,1'-biphenyl]-3-ol
13C NMR at 100.63 MHz in CD2Cl2

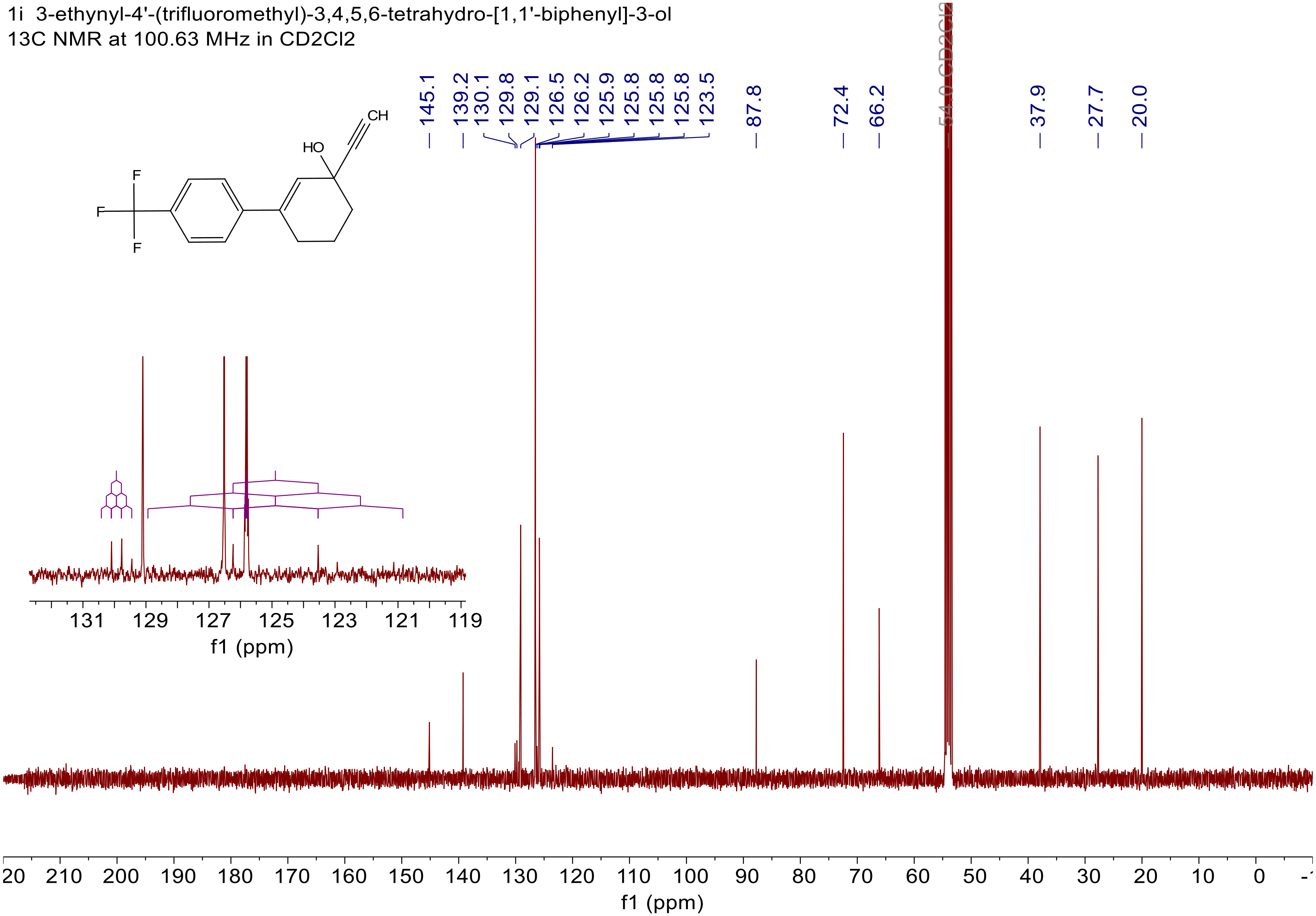

1i 3-ethynyl-4'-(trifluoromethyl)-3,4,5,6-tetrahydro-[1,1'-biphenyl]-3-ol

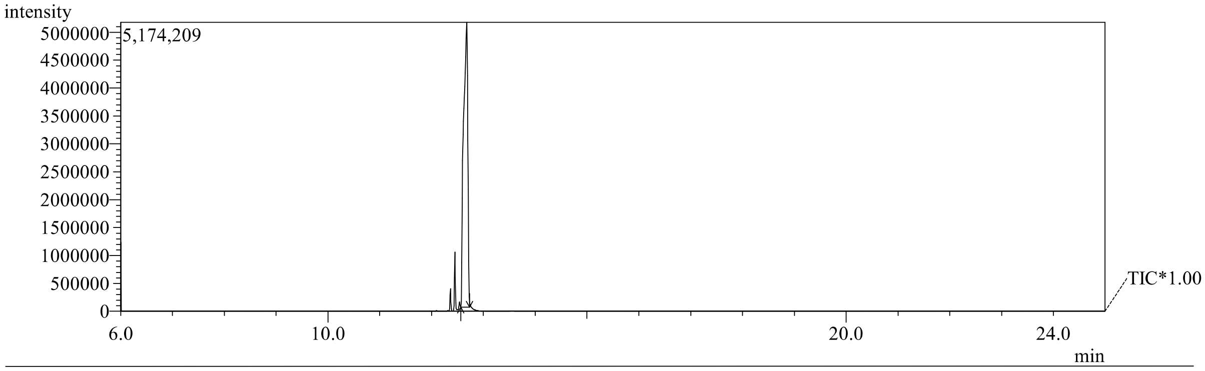

Spectrum

MassPeaks:152
RawMode:Averaged 12.7-12.7(802-804) BasePeak:238(459568)
BG Mode:Calc. from Peak

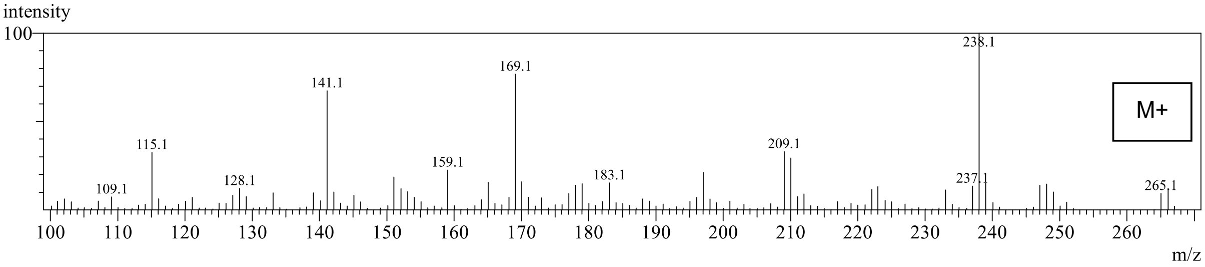

1j 4'-methyl-3,4,5,6-tetrahydro-[1,1'-biphenyl]-3-ol
1H NMR at 400.15 MHz in CDCl3

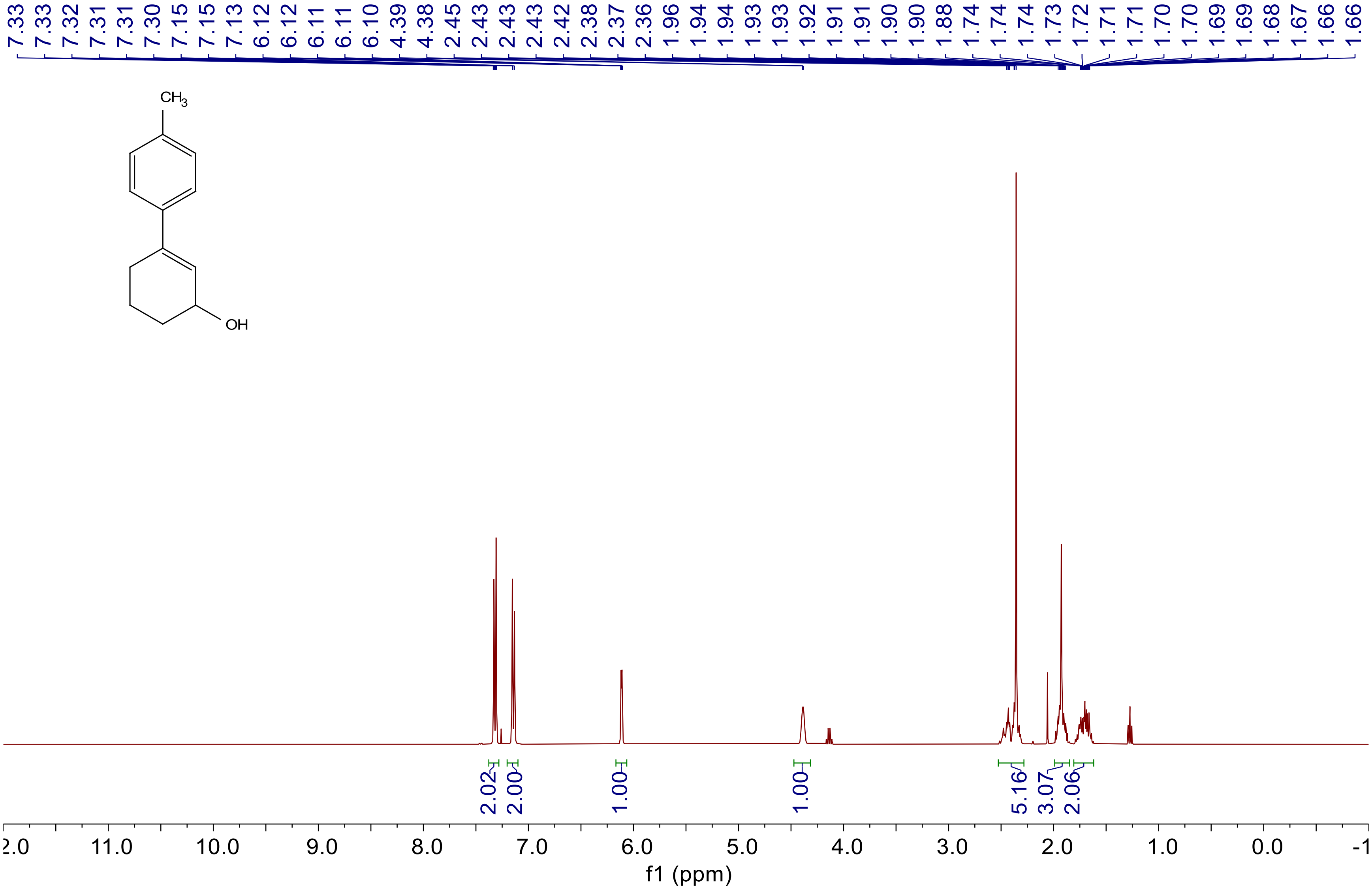

1j 4'-methyl-3,4,5,6-tetrahydro-[1,1'-biphenyl]-3-ol
13C NMR at 100.63 MHz in CDCl3

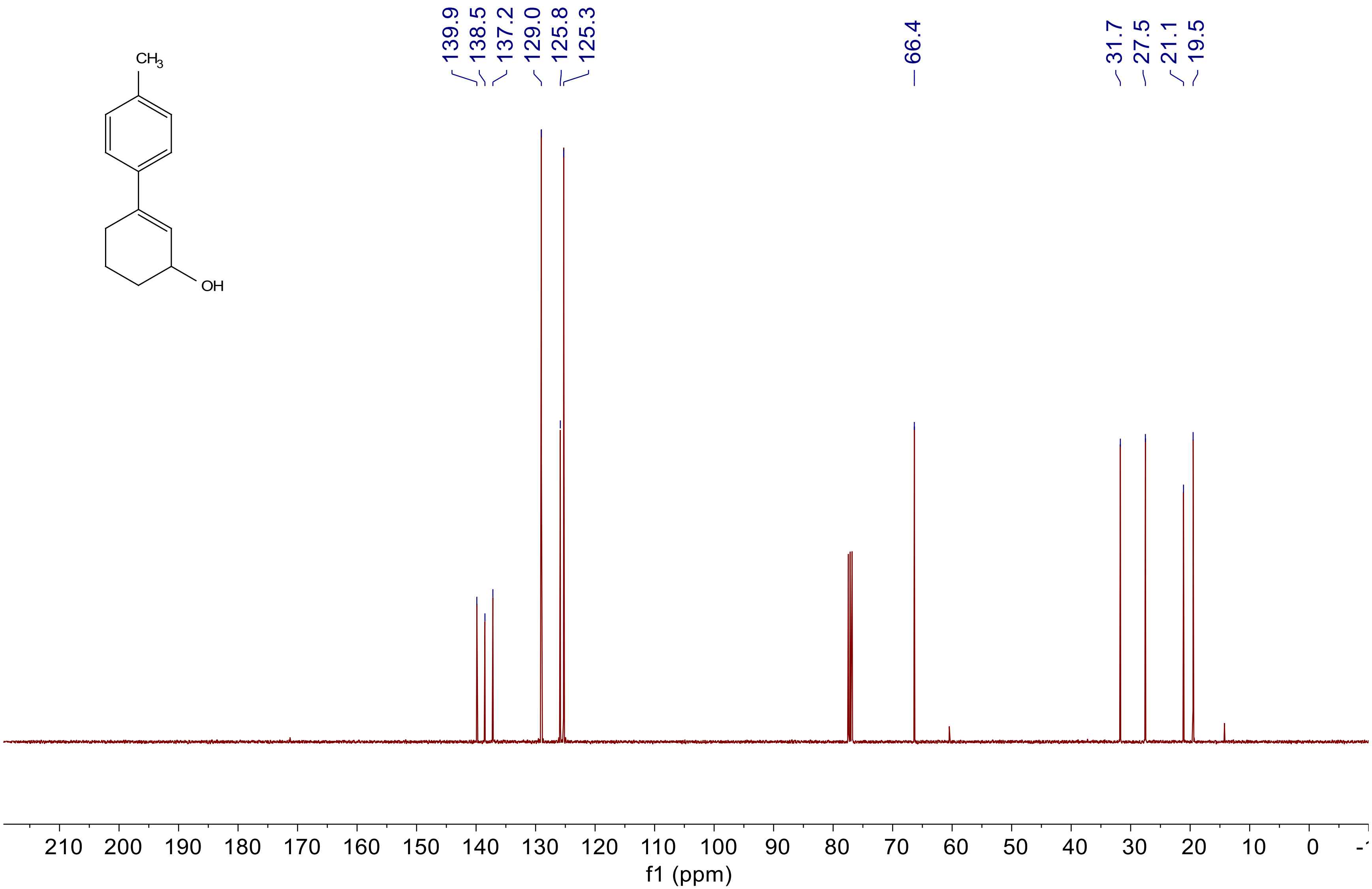

1k 4'-(trifluoromethyl)-3,4,5,6-tetrahydro-[1,1'-biphenyl]-3-ol
1H NMR at 400.15 MHz in CD3CN

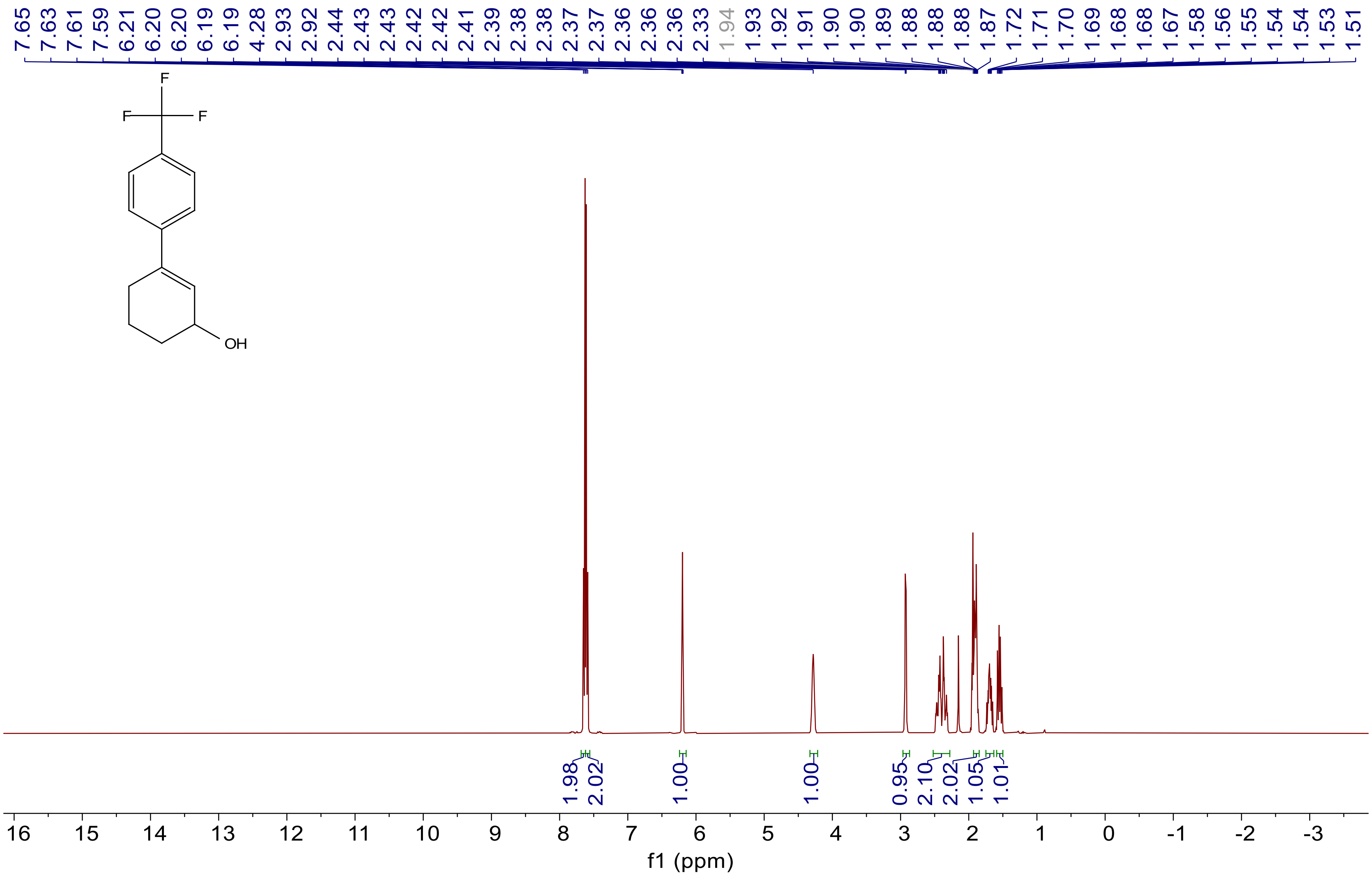

1k 4'-(trifluoromethyl)-3,4,5,6-tetrahydro-[1,1'-biphenyl]-3-ol
19F NMR at 376.48 MHz in CD3CN

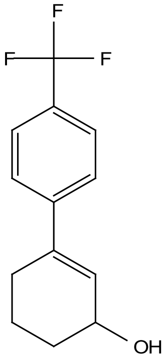

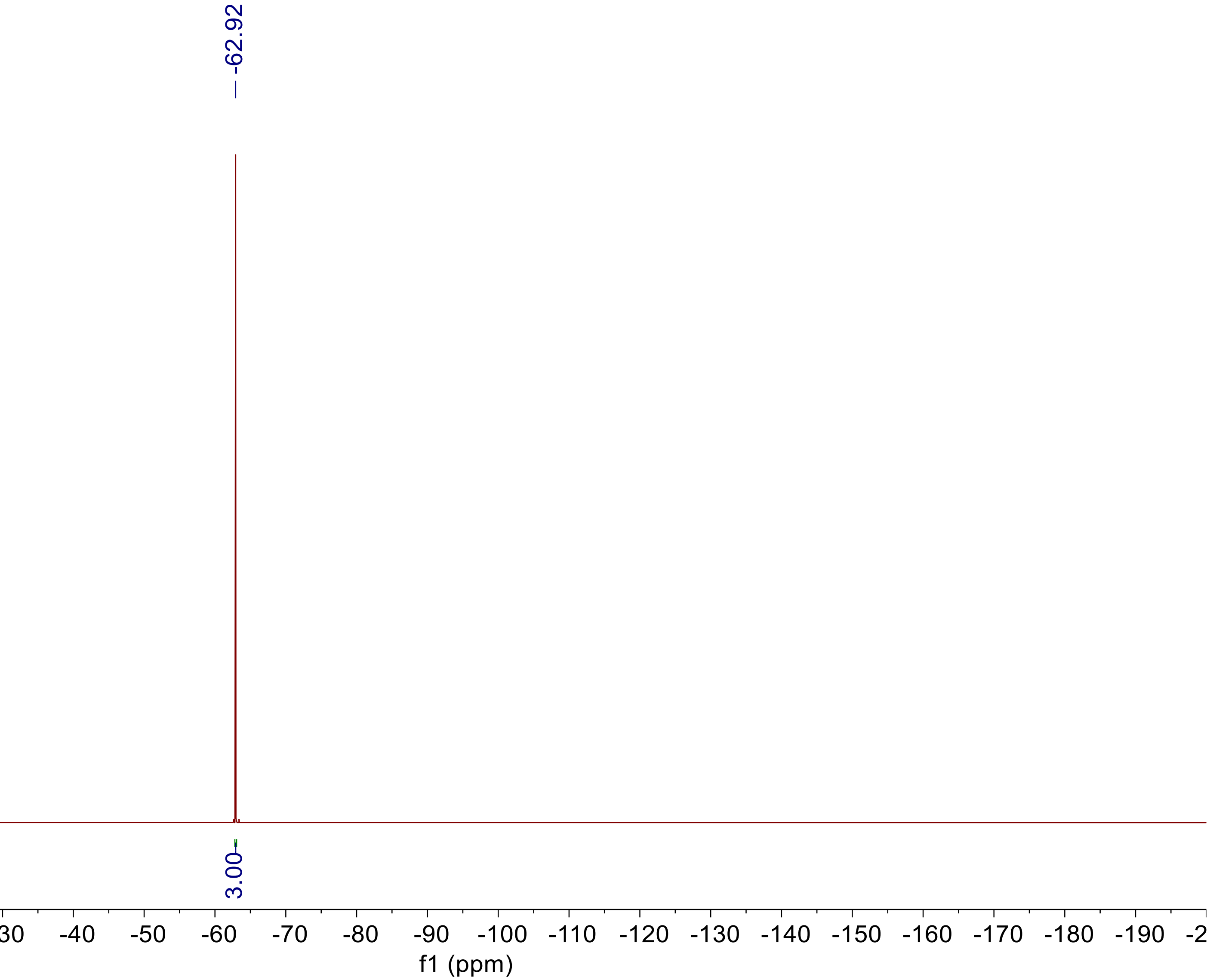

1k 4'-(trifluoromethyl)-3,4,5,6-tetrahydro-[1,1'-biphenyl]-3-ol

13C NMR at 100.63 MHz in CD3CN

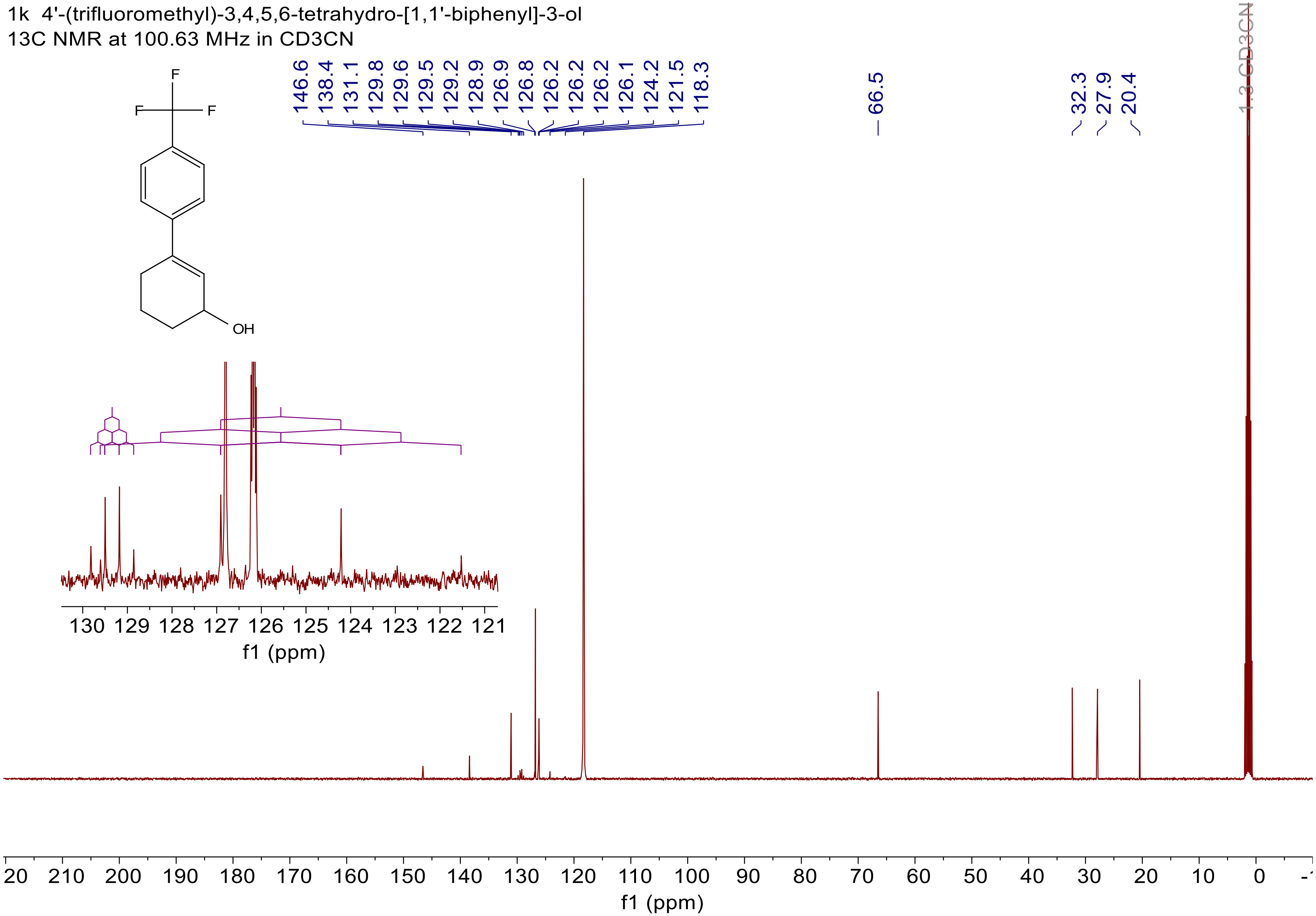

1k 4'-(trifluoromethyl)-3,4,5,6-tetrahydro-[1,1'-biphenyl]-3-ol

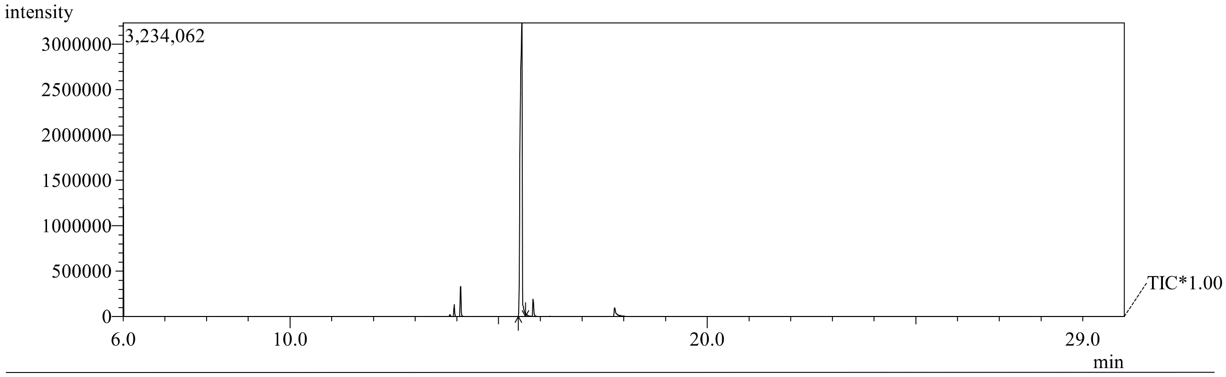

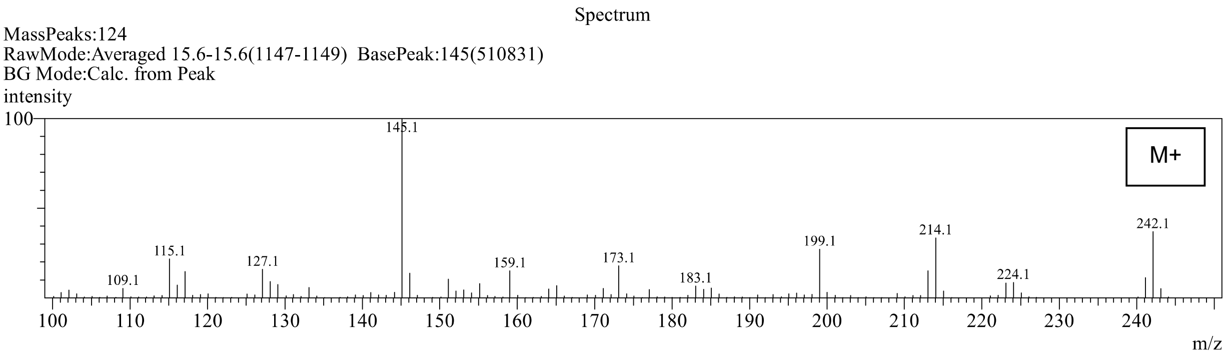

1l 3,4,5,6-tetrahydro-[1,1'-biphenyl]-3-ol
1H NMR at 400.15 MHz in CD2Cl2

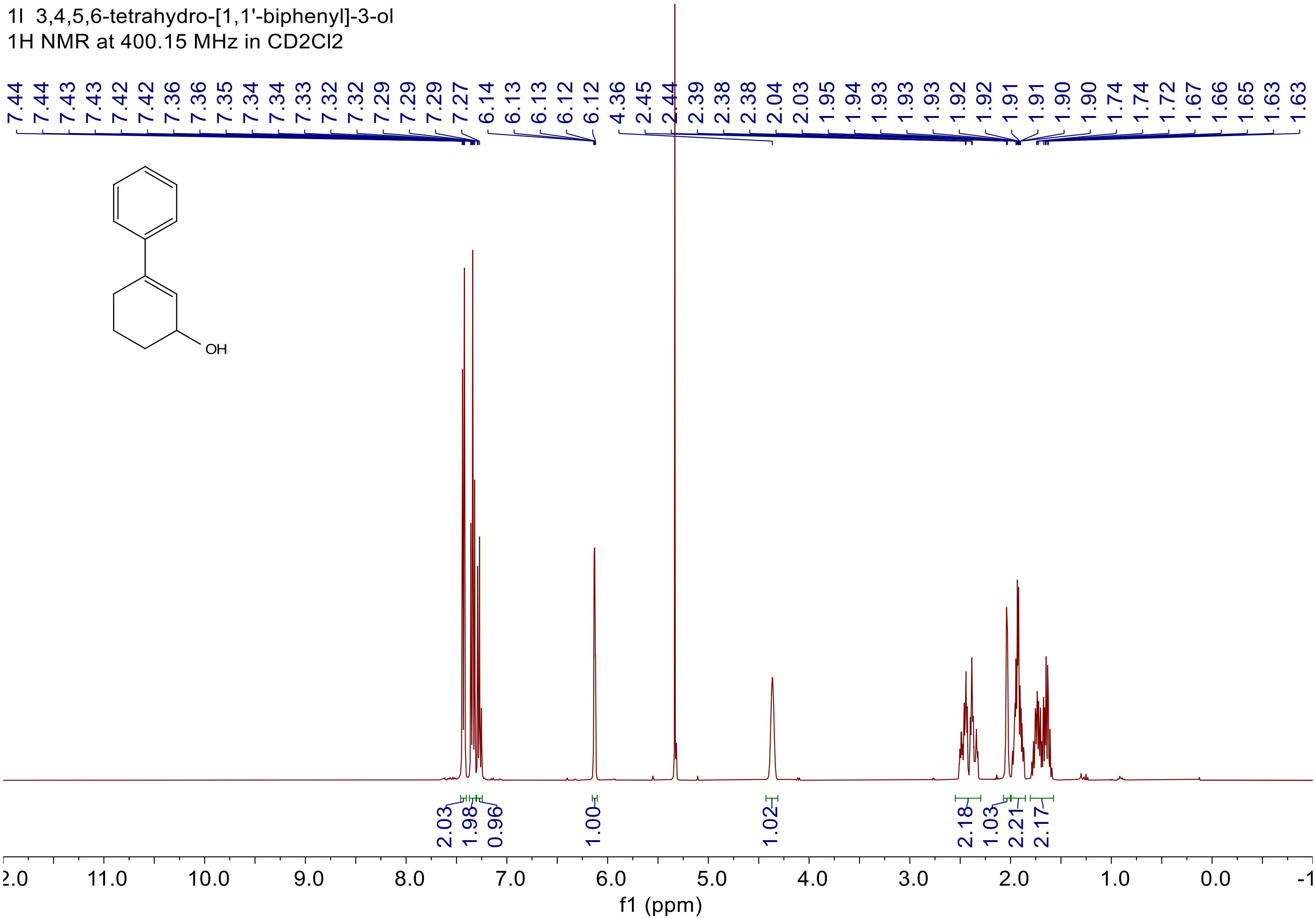

1l 3,4,5,6-tetrahydro-[1,1'-biphenyl]-3-ol
13C NMR at 100.63 MHz in CD2Cl2

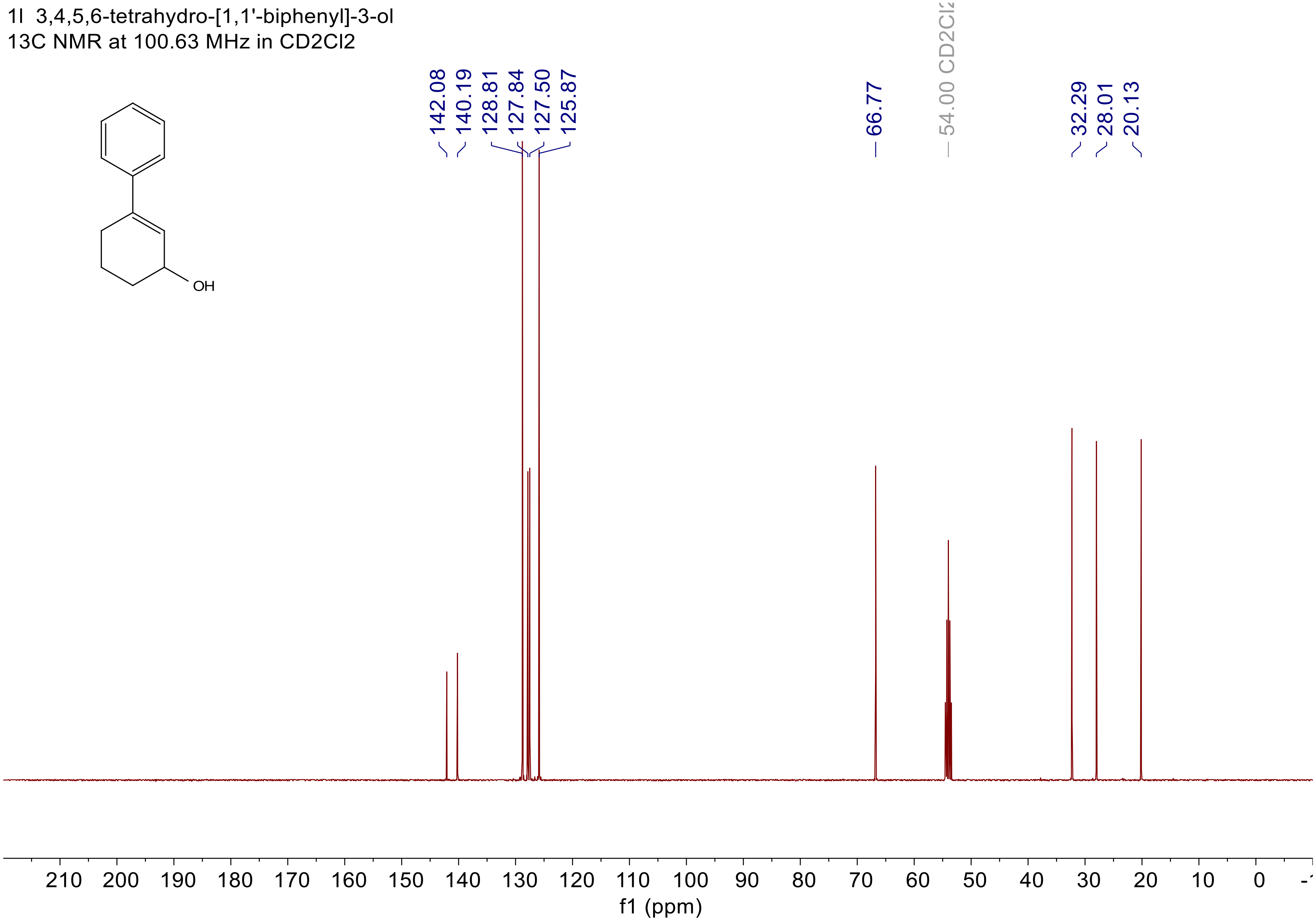

1m 4'-methoxy-3,4,5,6-tetrahydro-[1,1'-biphenyl]-3-ol
1H NMR at 400.15 MHz in CD2Cl2

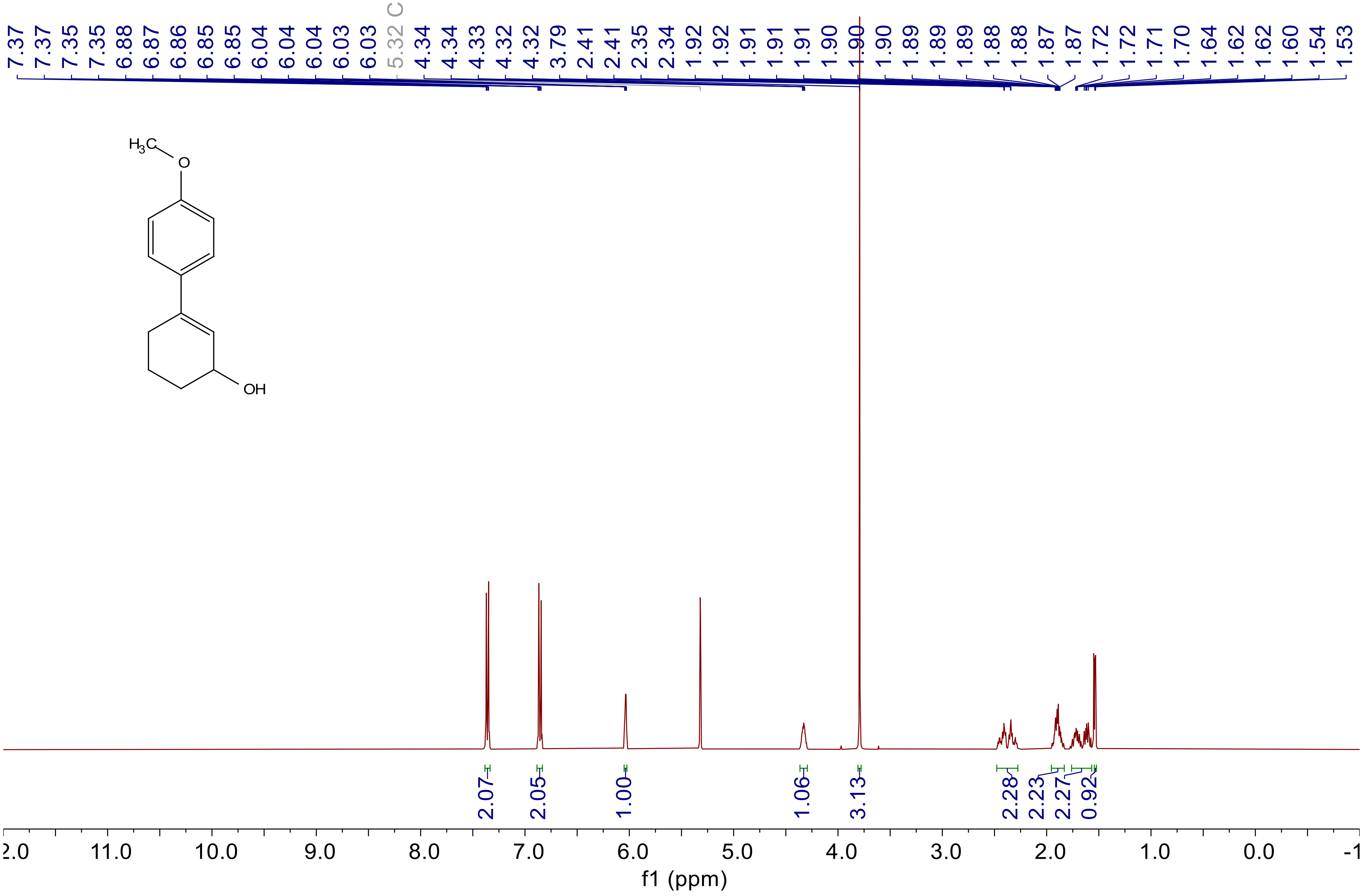

1m 4'-methoxy-3,4,5,6-tetrahydro-[1,1'-biphenyl]-3-ol
13C NMR at 100.63 MHz in CD2Cl2

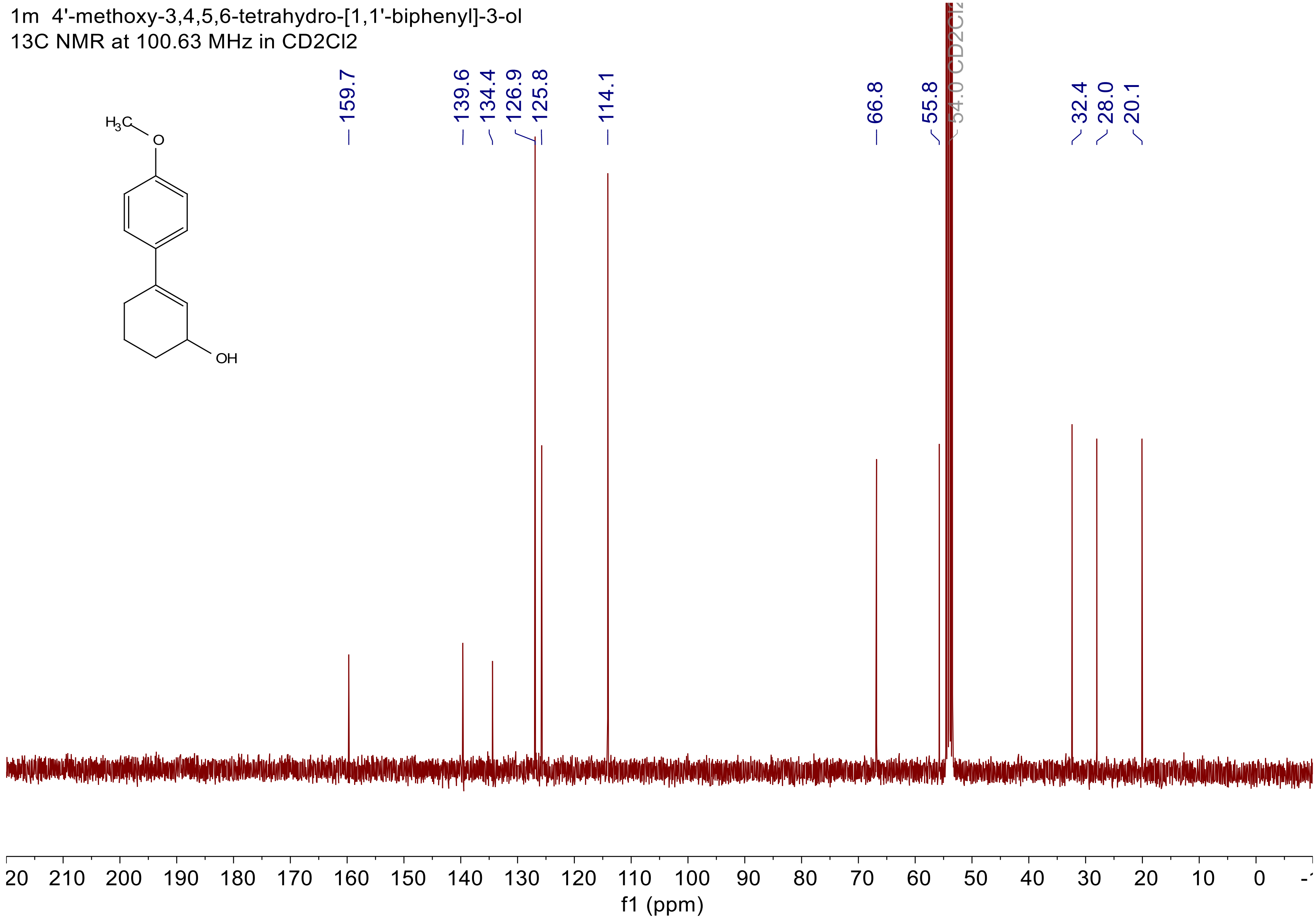

1n 3-(furan-2-yl)cyclohex-2-en-1-ol
1H NMR at 598.93 MHz in CD3CN

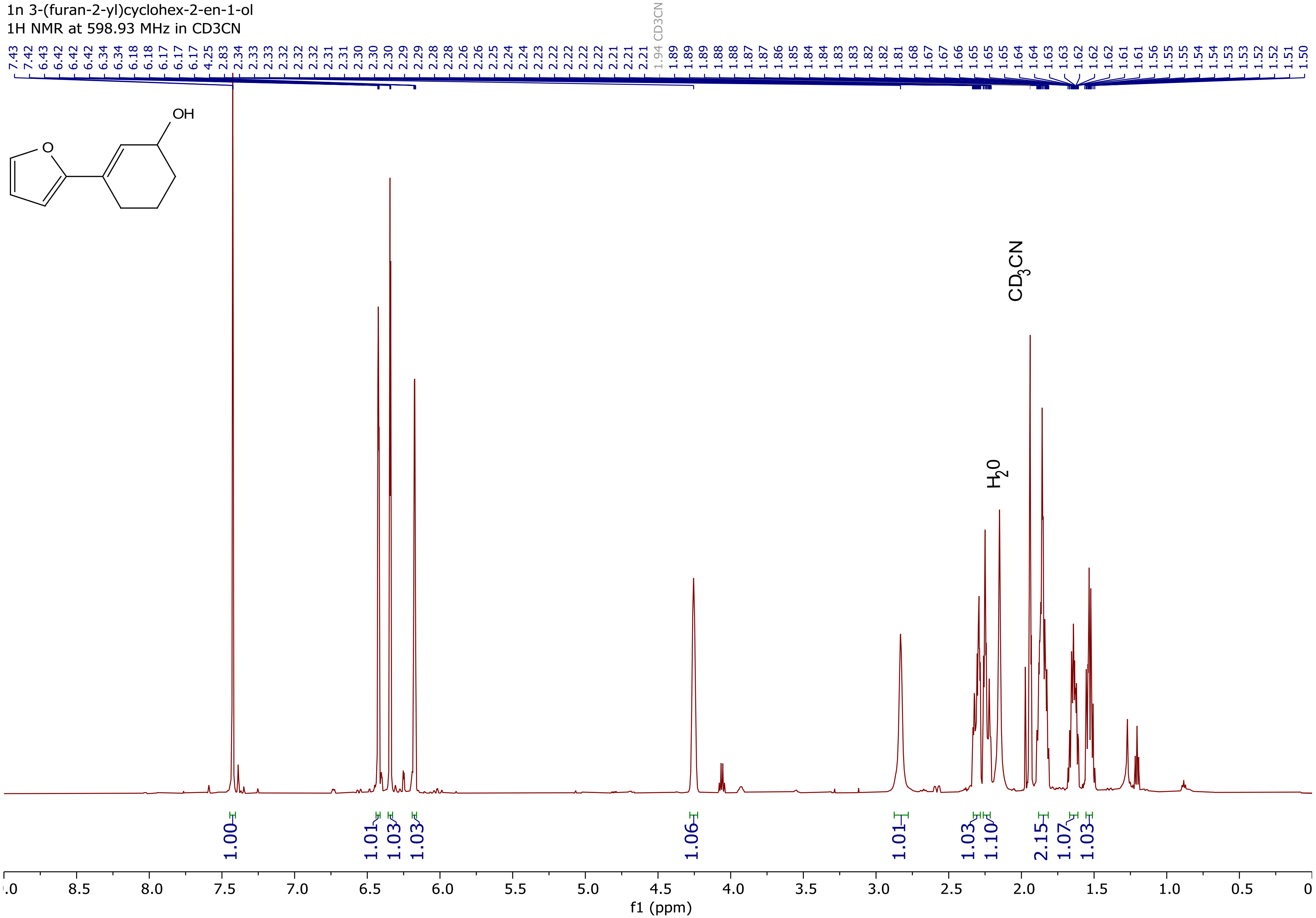

1n 3-(furan-2-yl)cyclohex-2-en-1-ol
13C NMR at 150.62 MHz in CD3CN

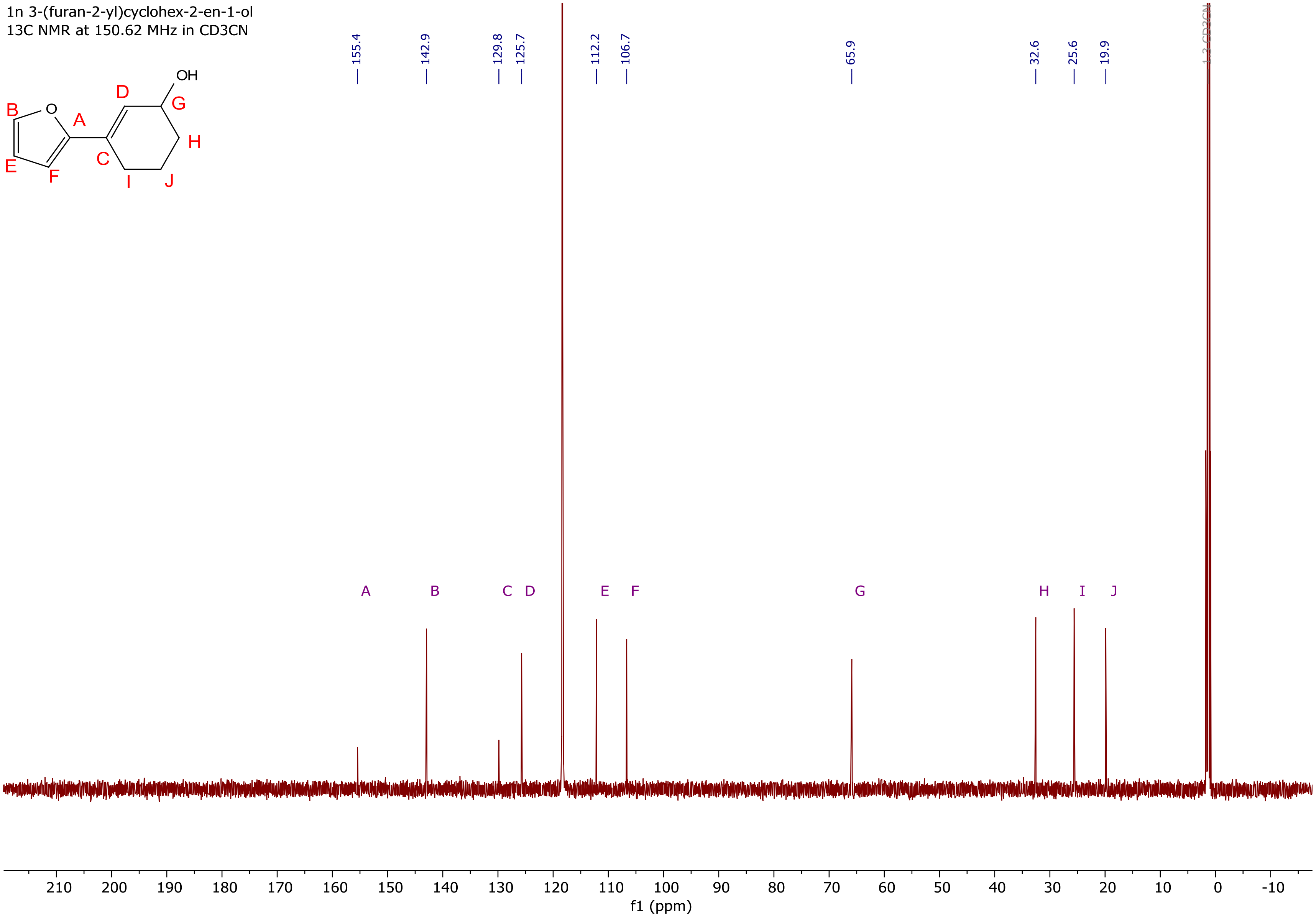

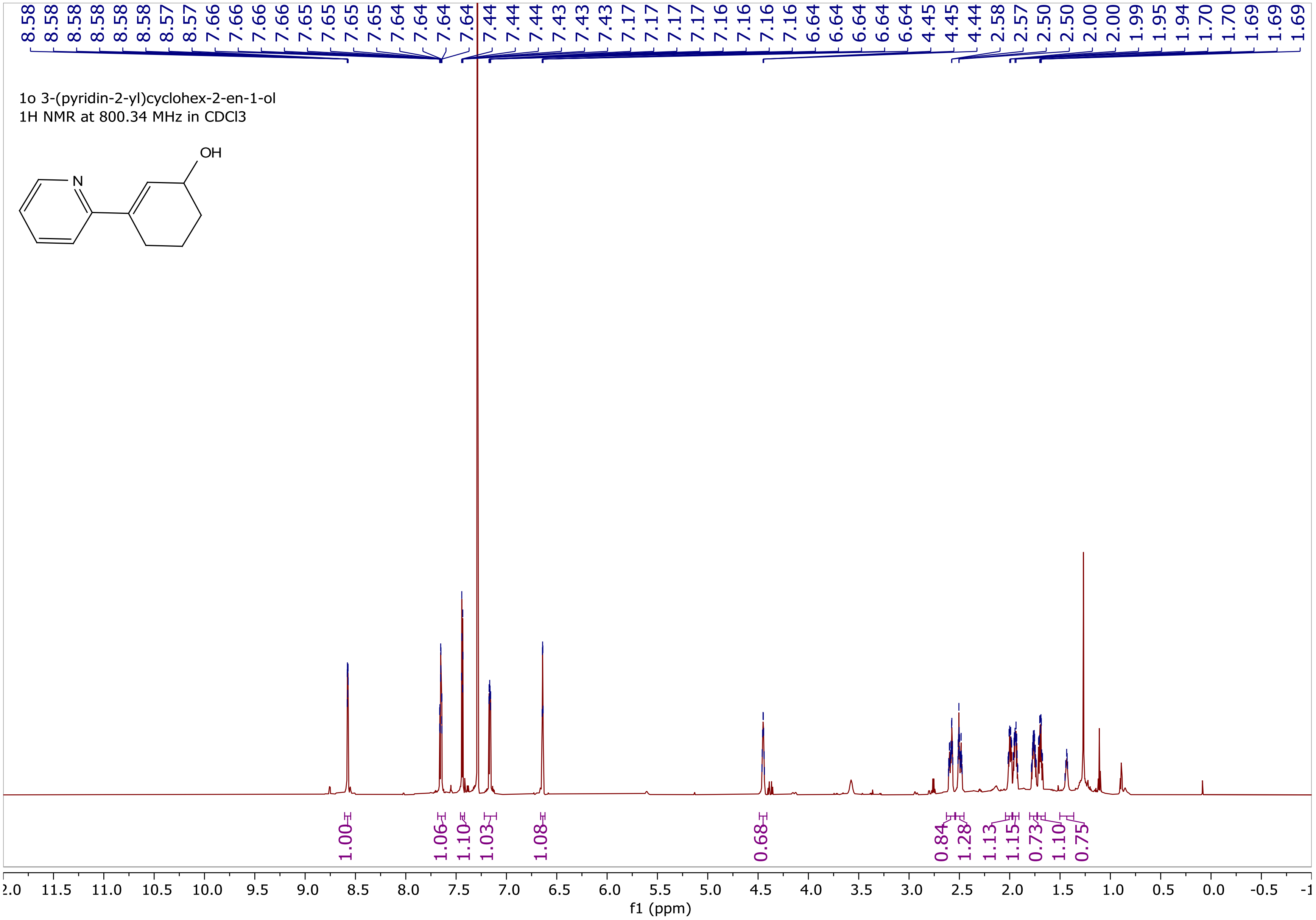

1o 3-(pyridin-2-yl)cyclohex-2-en-1-ol
1H NMR at 800.34 MHz in CDCl3
OH
N
8.58 8.58 8.58 8.58 8.58 8.58 8.57 8.57 7.66 7.66 7.66 7.66 7.65 7.65 7.65 7.65 7.64 7.64 7.64 7.64 7.44 7.44 7.44 7.43 7.43 7.43 7.17 7.17 7.17 7.17 7.16 7.16 7.16 7.16 6.64 6.64 6.64 6.64 6.64 4.45 4.45 4.44 2.58 2.57 2.50 2.50 2.00 2.00 1.99 1.95 1.94 1.70 1.70 1.69 1.69 1.69
1.00
1.06
1.10
1.03
1.08
0.68
0.84
1.28
1.13
1.15
0.73
1.10
0.75
f1 (ppm)

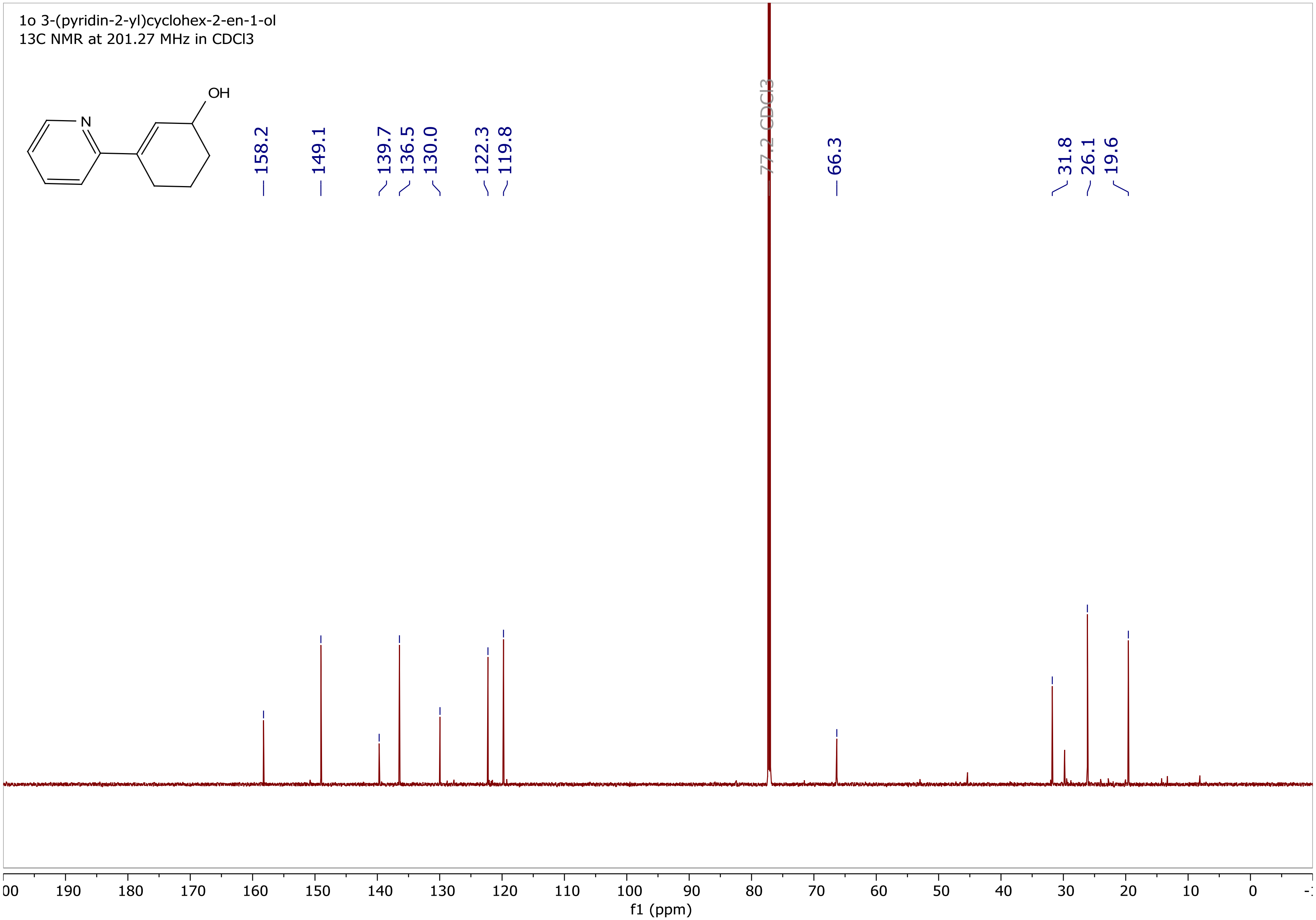

1o 3-(pyridin-2-yl)cyclohex-2-en-1-ol
13C NMR at 201.27 MHz in CDCl3
OH
N
158.2
149.1
139.7
136.5
130.0
122.3
119.8
77.2 CDCl3
66.3
31.8
26.1
19.6
00
190
180
170
160
150
140
130
120
110
100
90
80
70
60
50
40
30
20
10
0
f1 (ppm)

1p 1,3-dibutylcyclohex-2-en-1-ol
1H NMR at 400.15 MHz in CDCl3

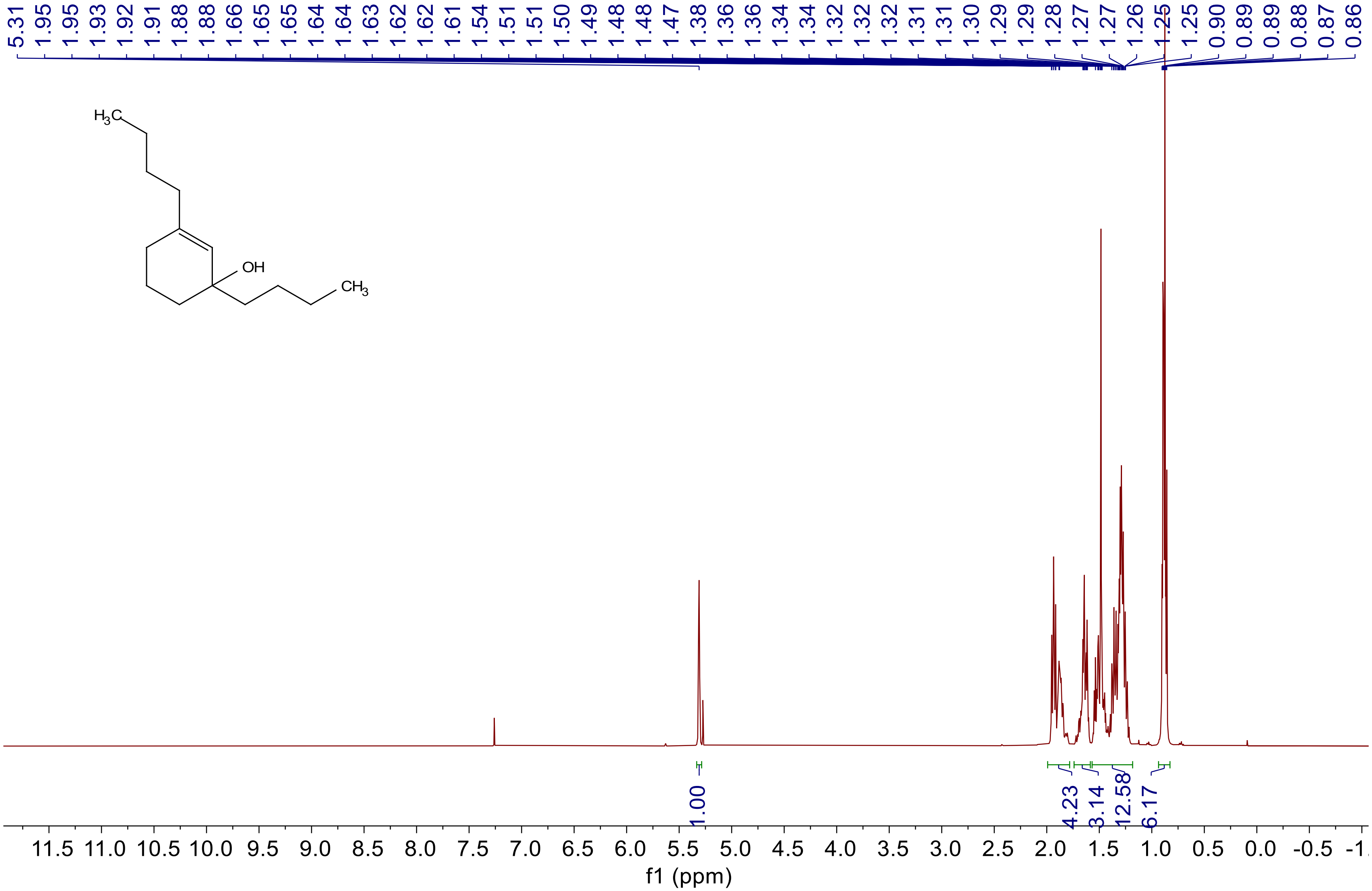

1p 1,3-dibutylcyclohex-2-en-1-ol
13C NMR at 100.63 MHz in CDCl3

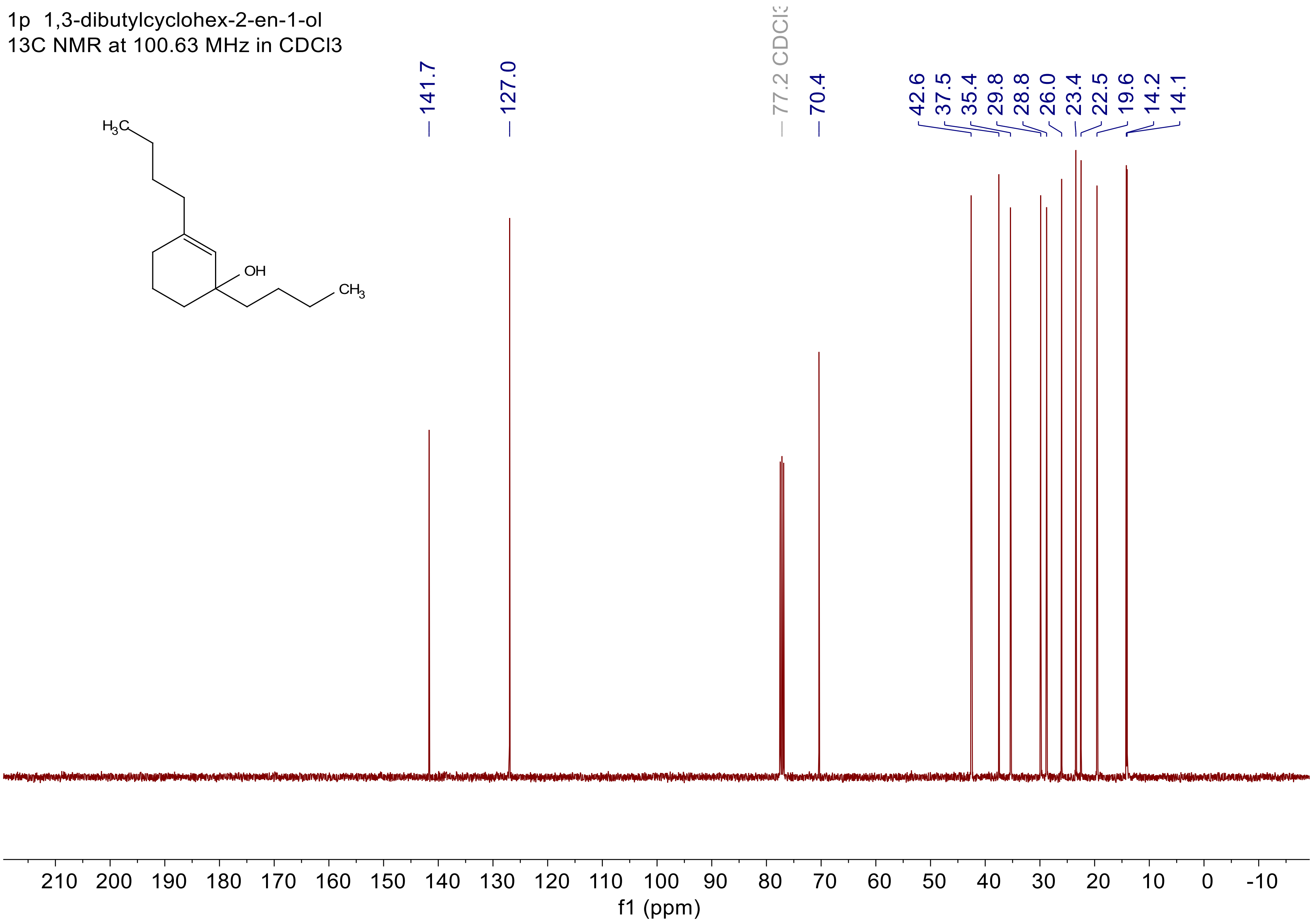

1q 3-hydroxycyclohex-1-ene-1-carbonitrile
1H NMR at 400.15 MHz in CD3CN

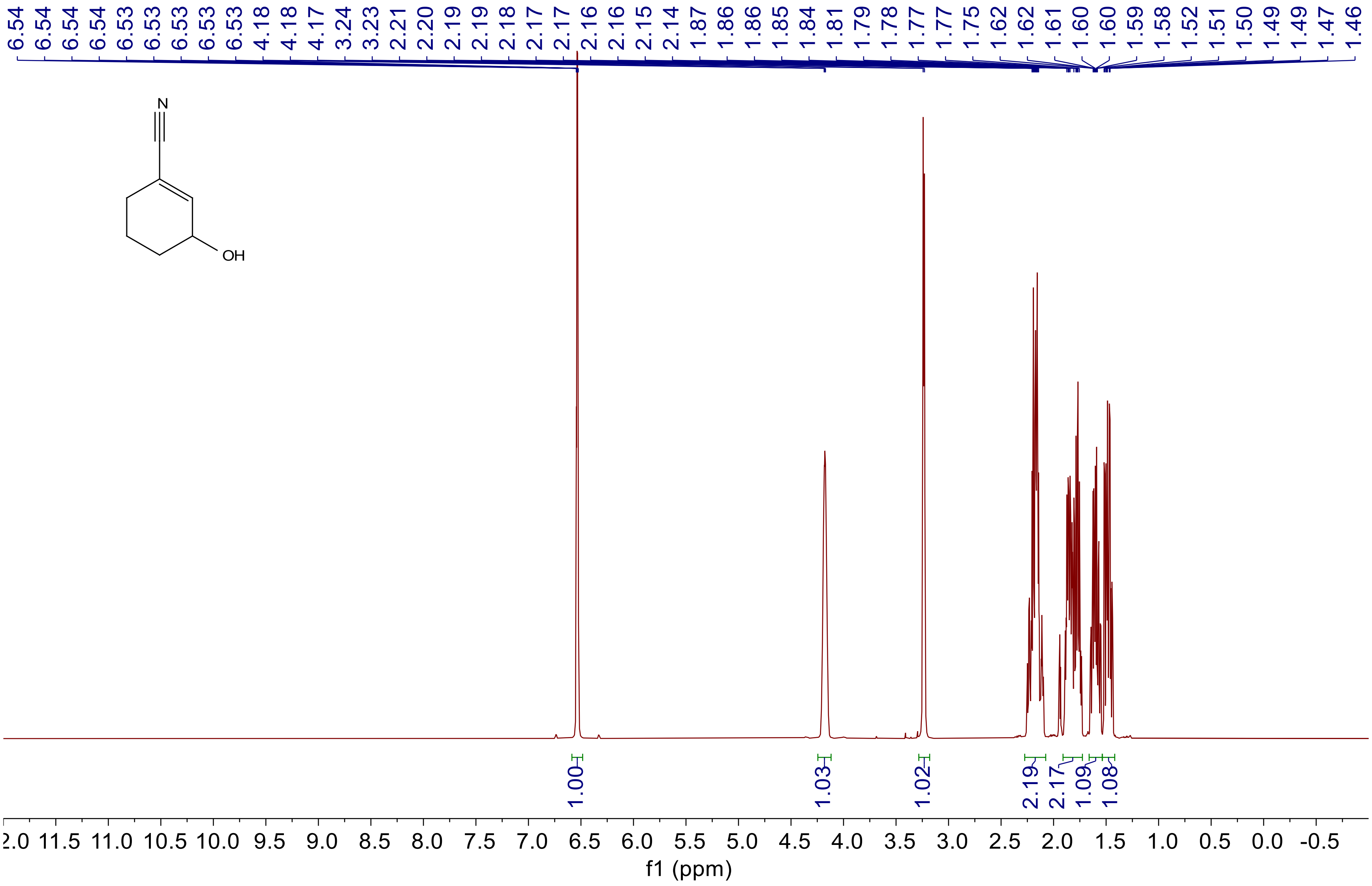

1q 3-hydroxycyclohex-1-ene-1-carbonitrile
13C NMR at 100.63 MHz in CD3CN

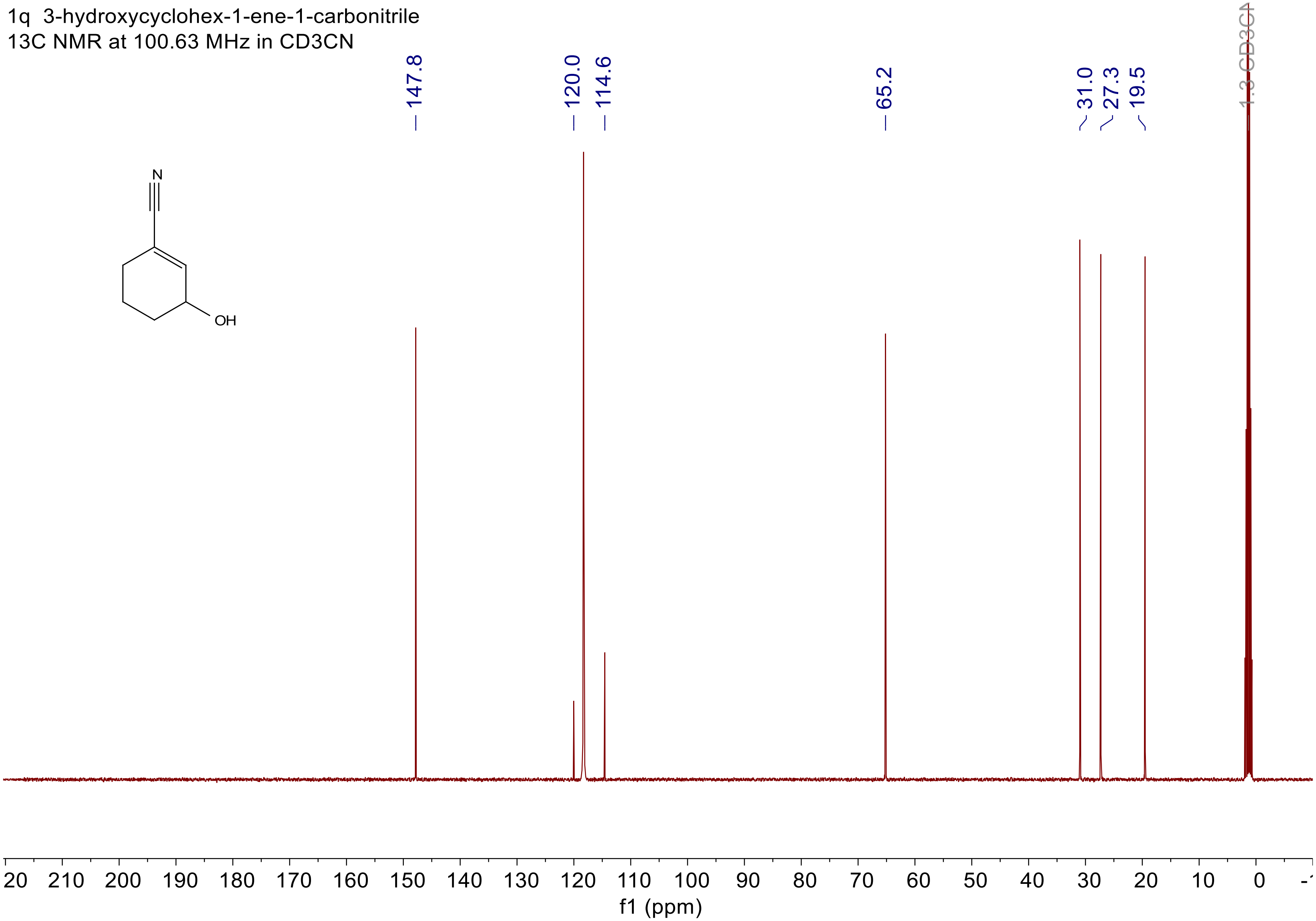

1q 3-hydroxycyclohex-1-ene-1-carbonitrile

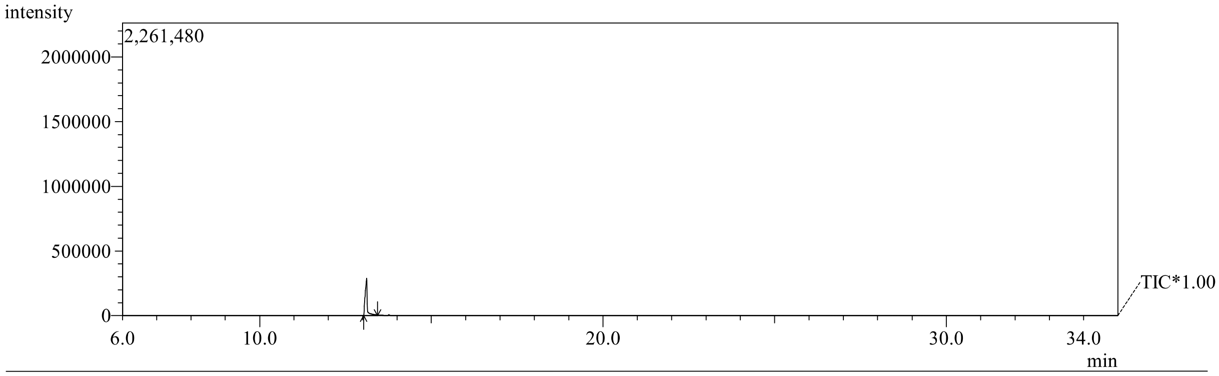

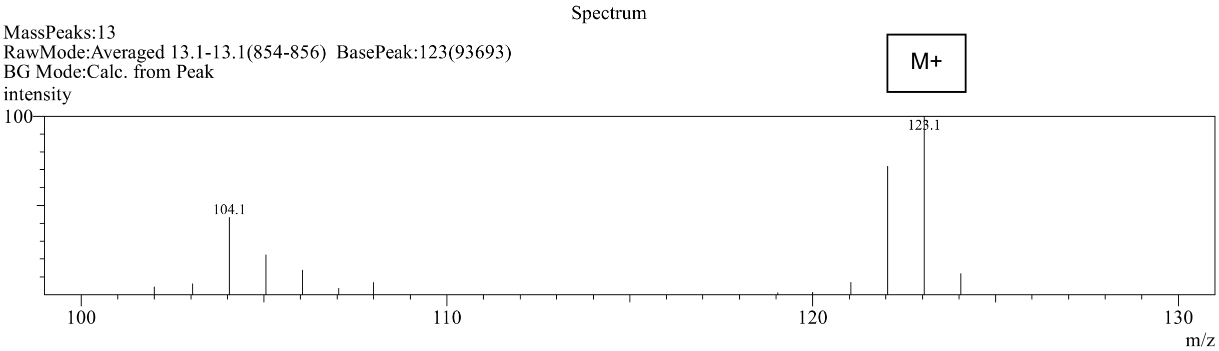

1r 4''-methoxy-4-(trifluoromethyl)-5',6'-dihydro-[1,1':3',1''-terphenyl]-1'(4'H)-ol
1H NMR at 400.15 MHz in CDCl3

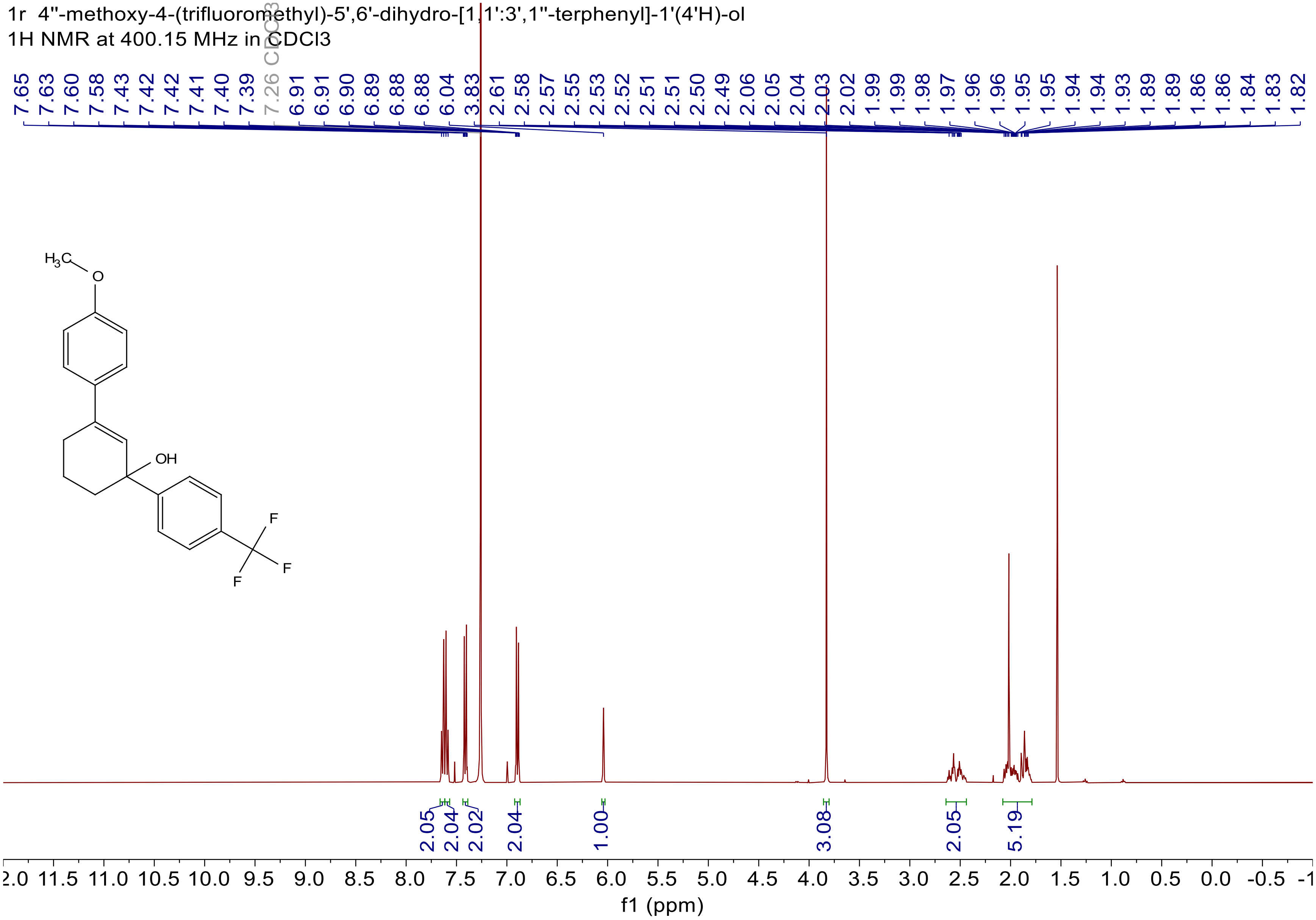

1r 4''-methoxy-4-(trifluoromethyl)-5',6'-dihydro-[1,1':3',1''-terphenyl]-1'(4'H)-ol
19F NMR at 376.48 MHz in CDCl3

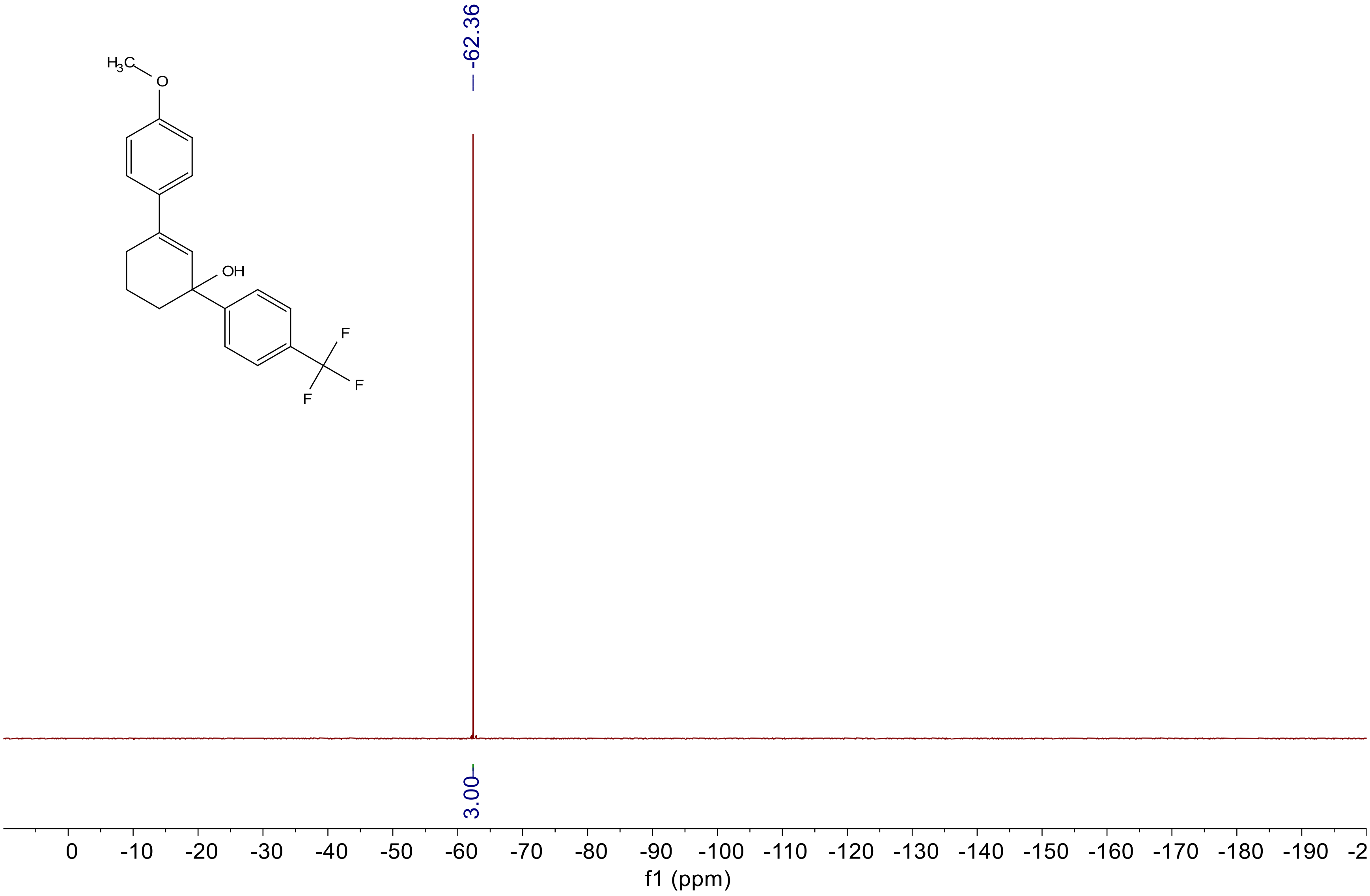

1r 4''-methoxy-4-(trifluoromethyl)-5',6'-dihydro-[1,1':3',1''-terphenyl]-1'(4'H)-ol
13C NMR at 100.63 MHz in CDCl3

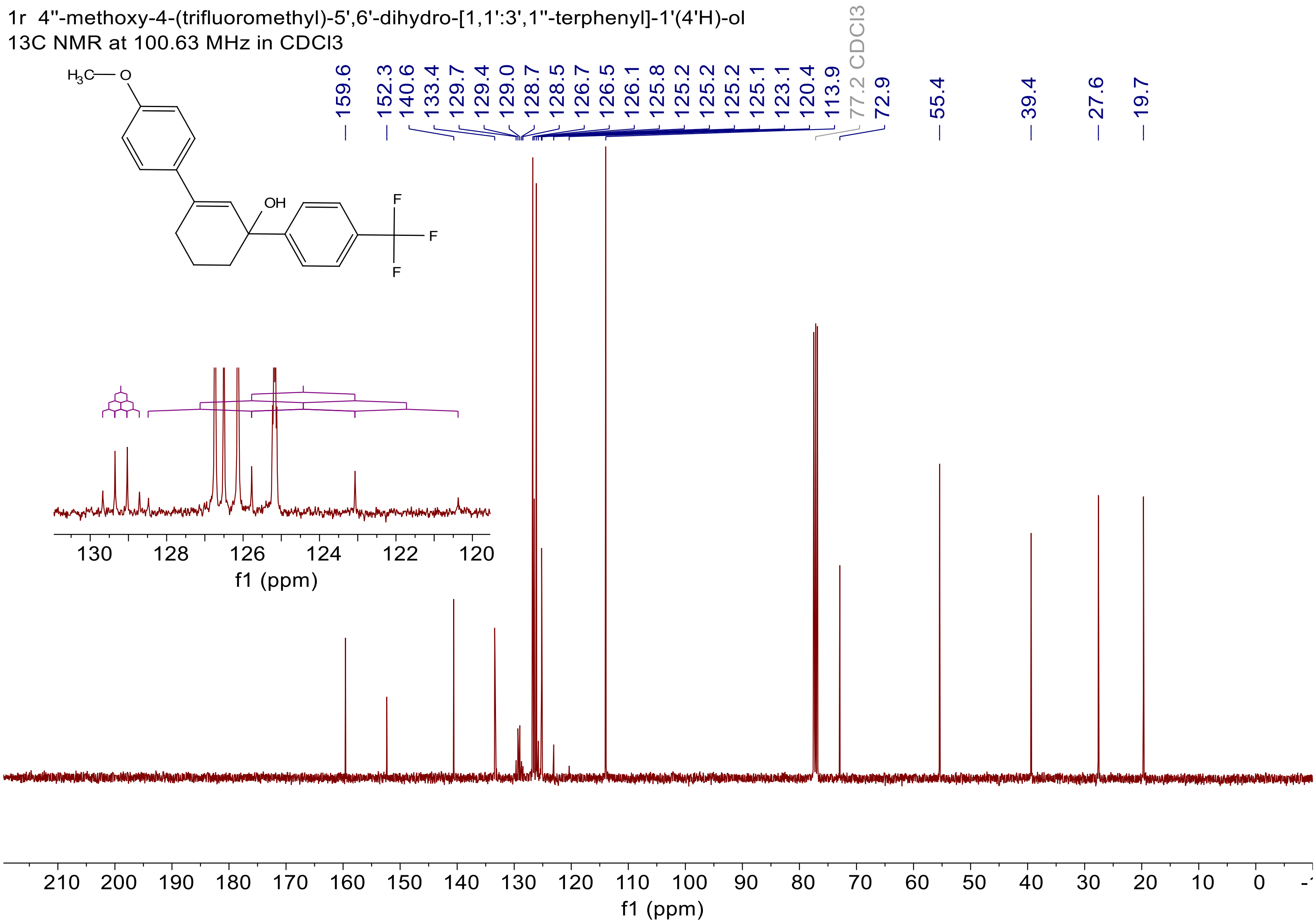

1s 4-methoxy-4''-(trifluoromethyl)-5',6'-dihydro-[1,1':3',1''-terphenyl]-1'(4'H)-ol
1H NMR at 400.15 MHz in CD3CN

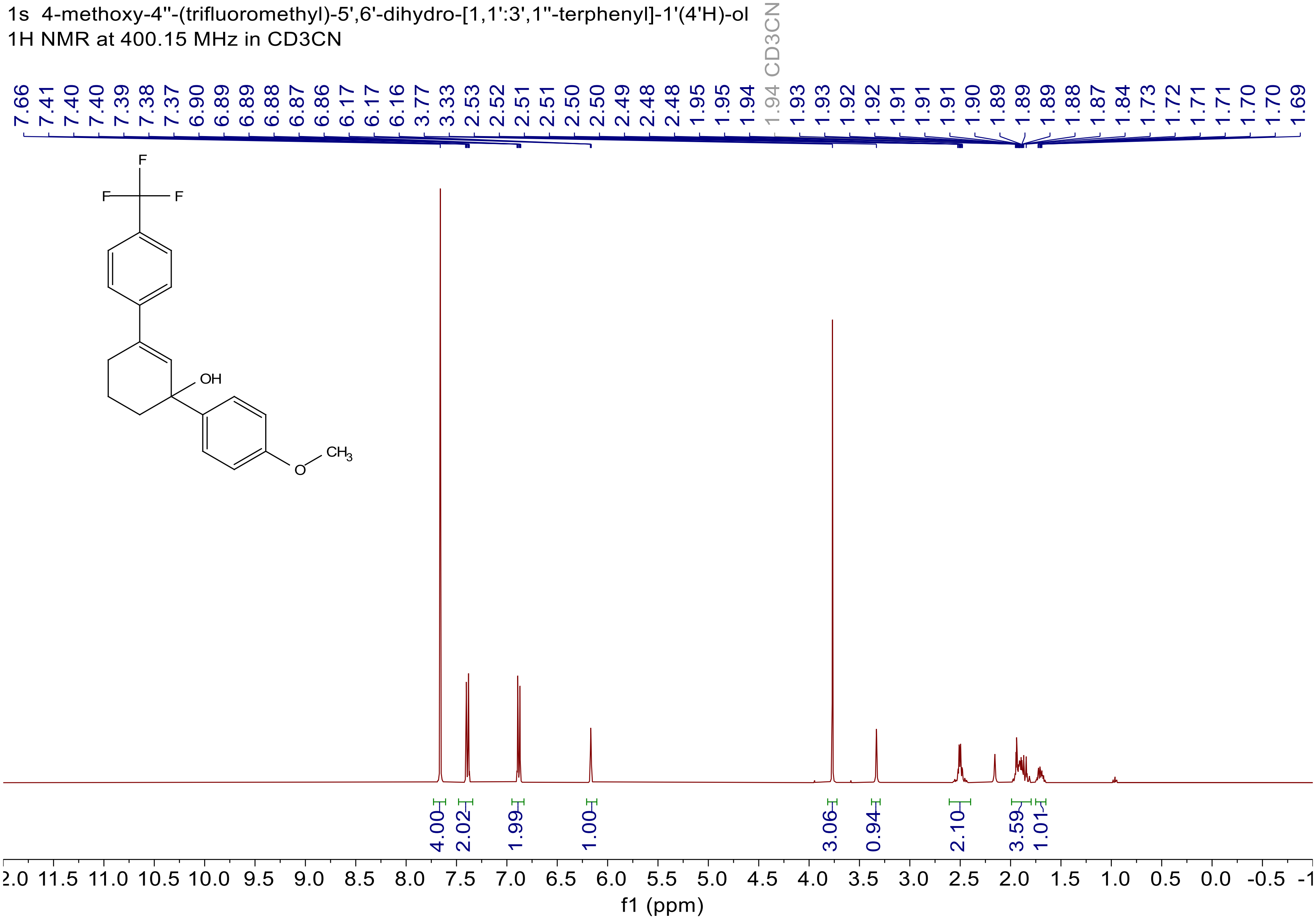

1s 4-methoxy-4''-(trifluoromethyl)-5',6'-dihydro-[1,1':3',1''-terphenyl]-1'(4'H)-ol
19F NMR at 376.48 MHz in CD3CN

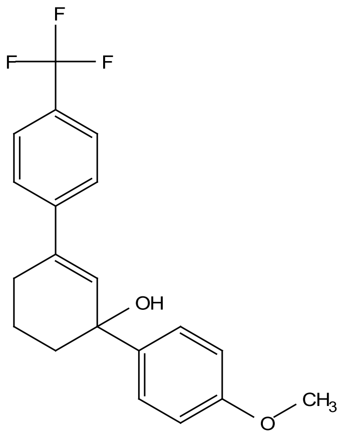

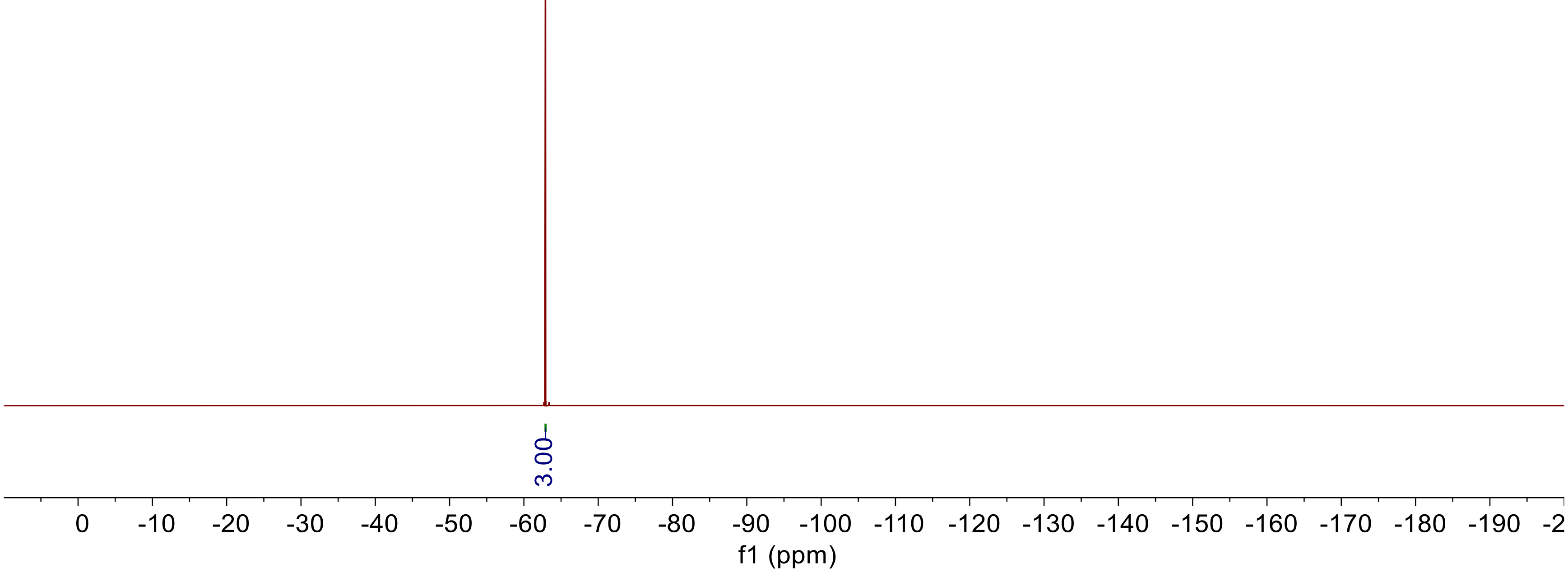

1s 4-methoxy-4''-(trifluoromethyl)-5',6'-dihydro-[1,1':3',1''-terphenyl]-1'(4'H)-ol
13C NMR at 100.63 MHz in CD3CN

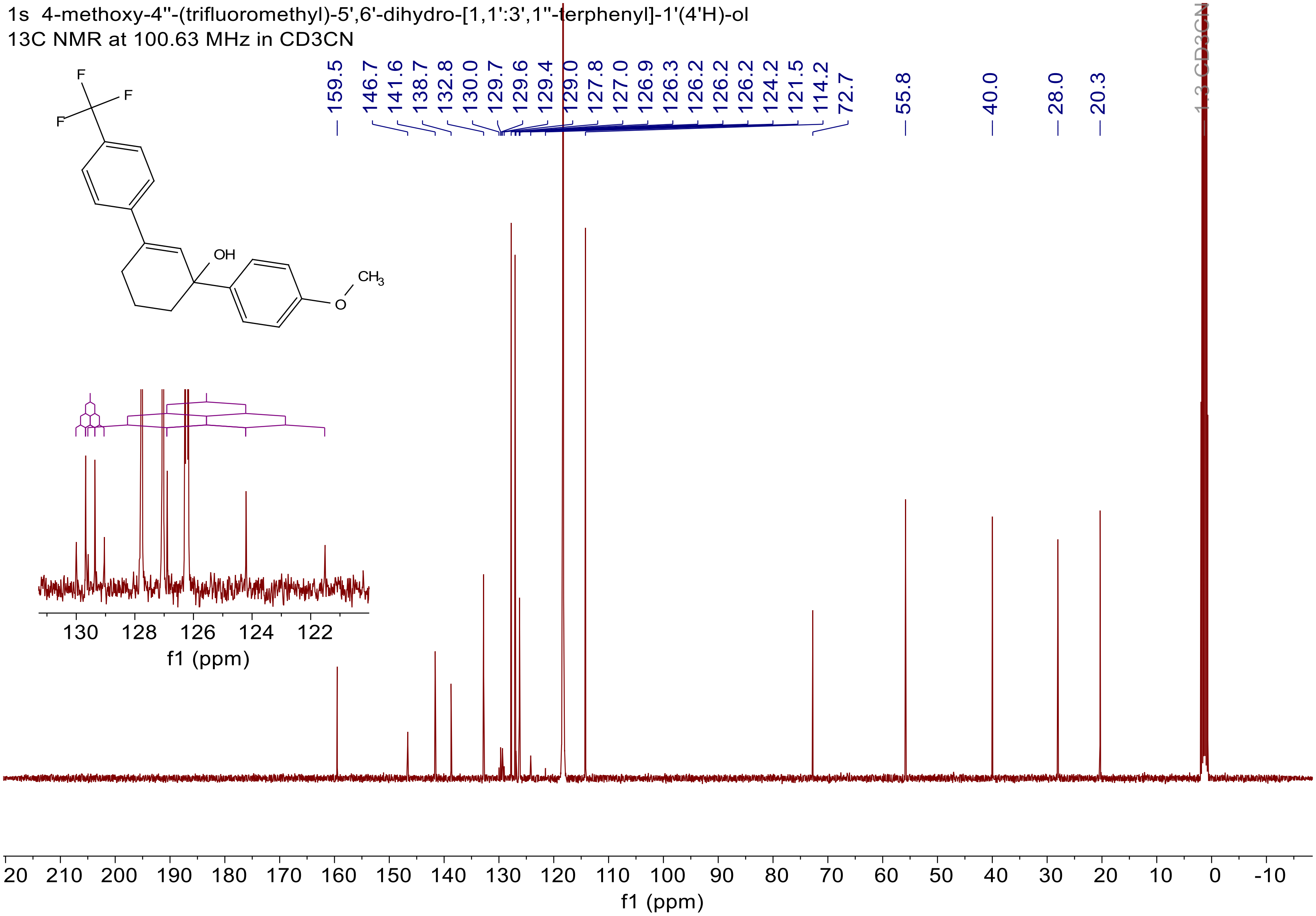

2a 1,5-bis(4-(trifluoromethyl)phenyl)hex-5-en-1-one
1H NMR at 400.15 MHz in CD3CN

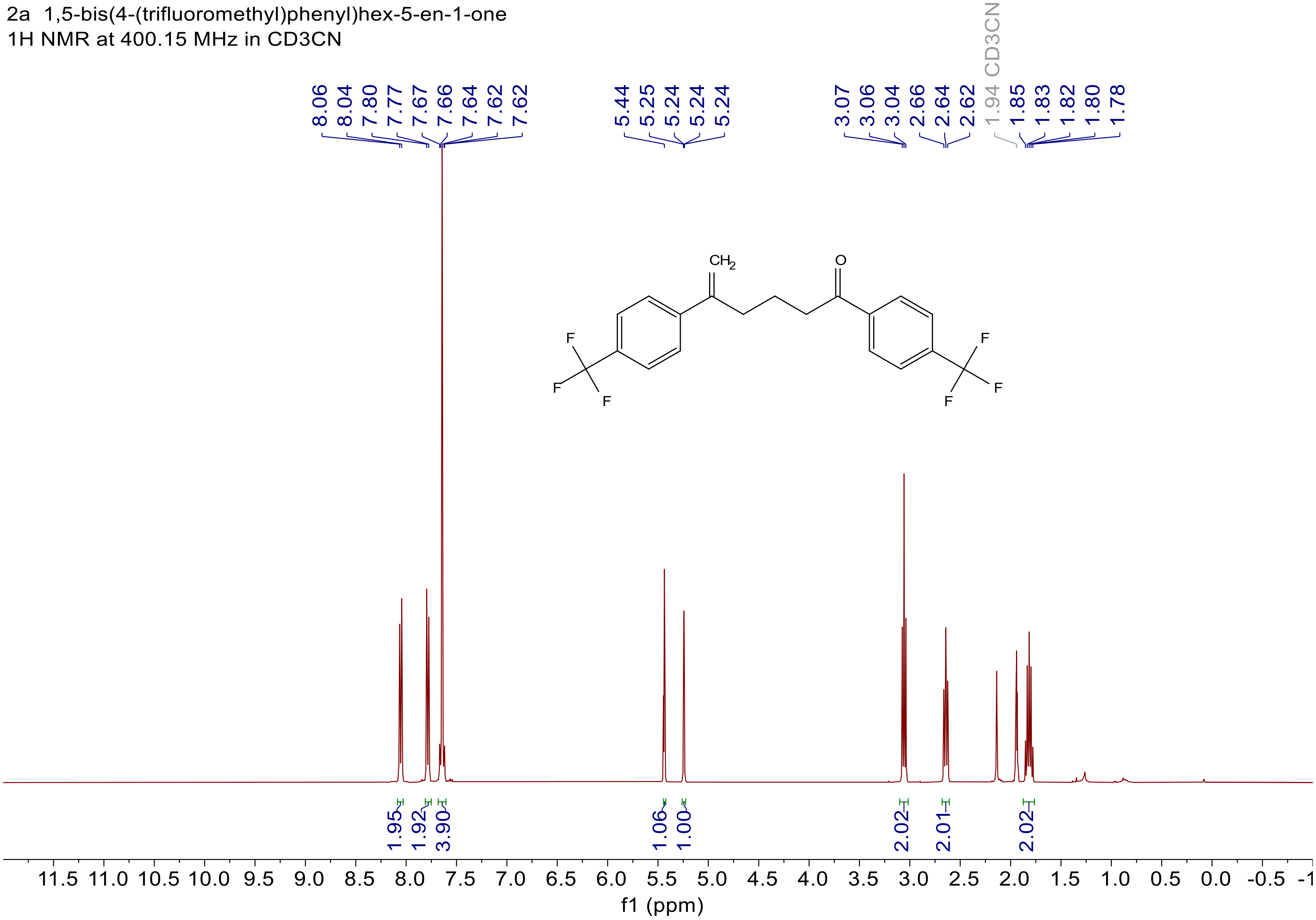

2a 1,5-bis(4-(trifluoromethyl)phenyl)hex-5-en-1-one
19F NMR at 376.48 MHz in CD3CN

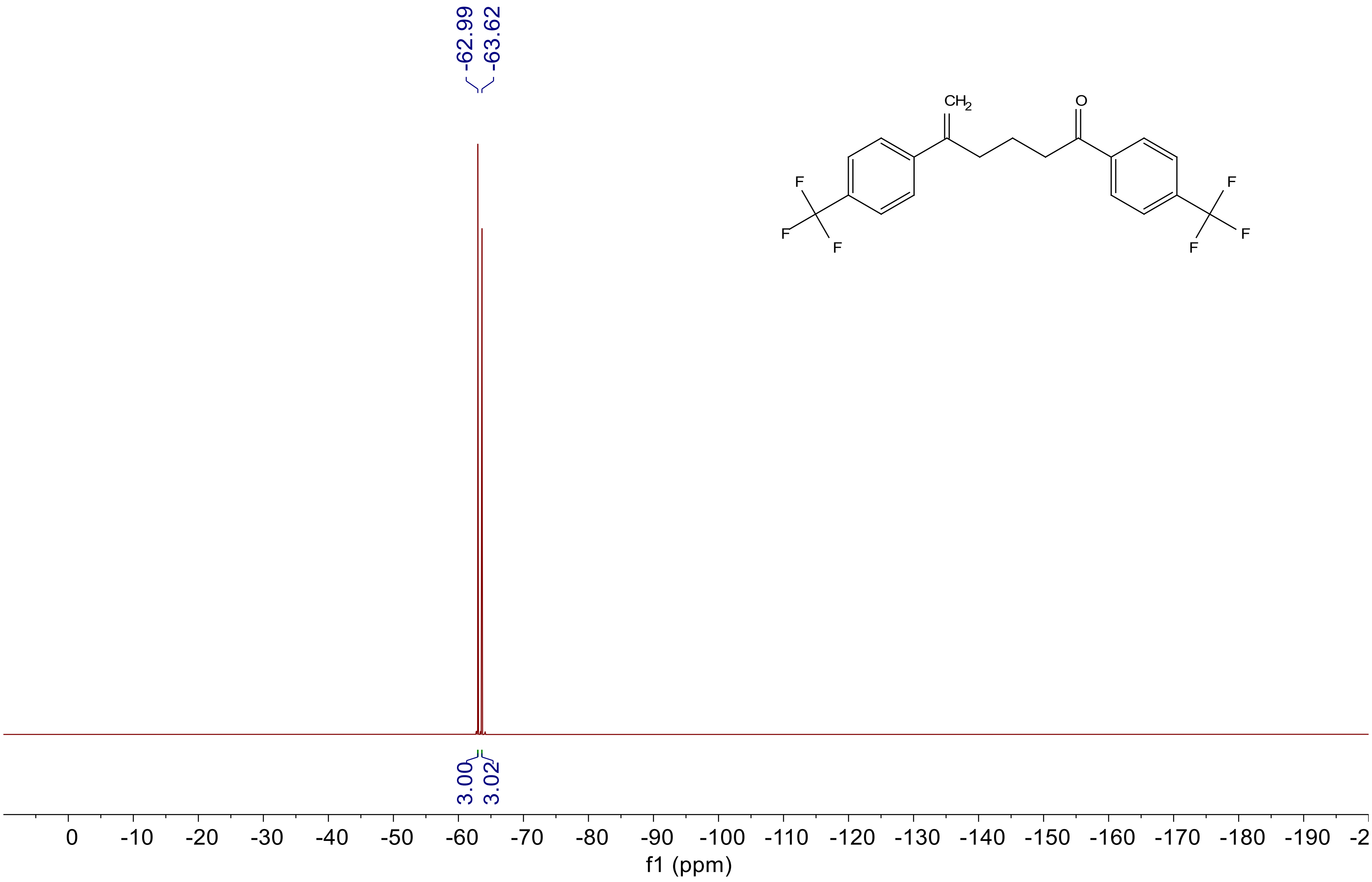

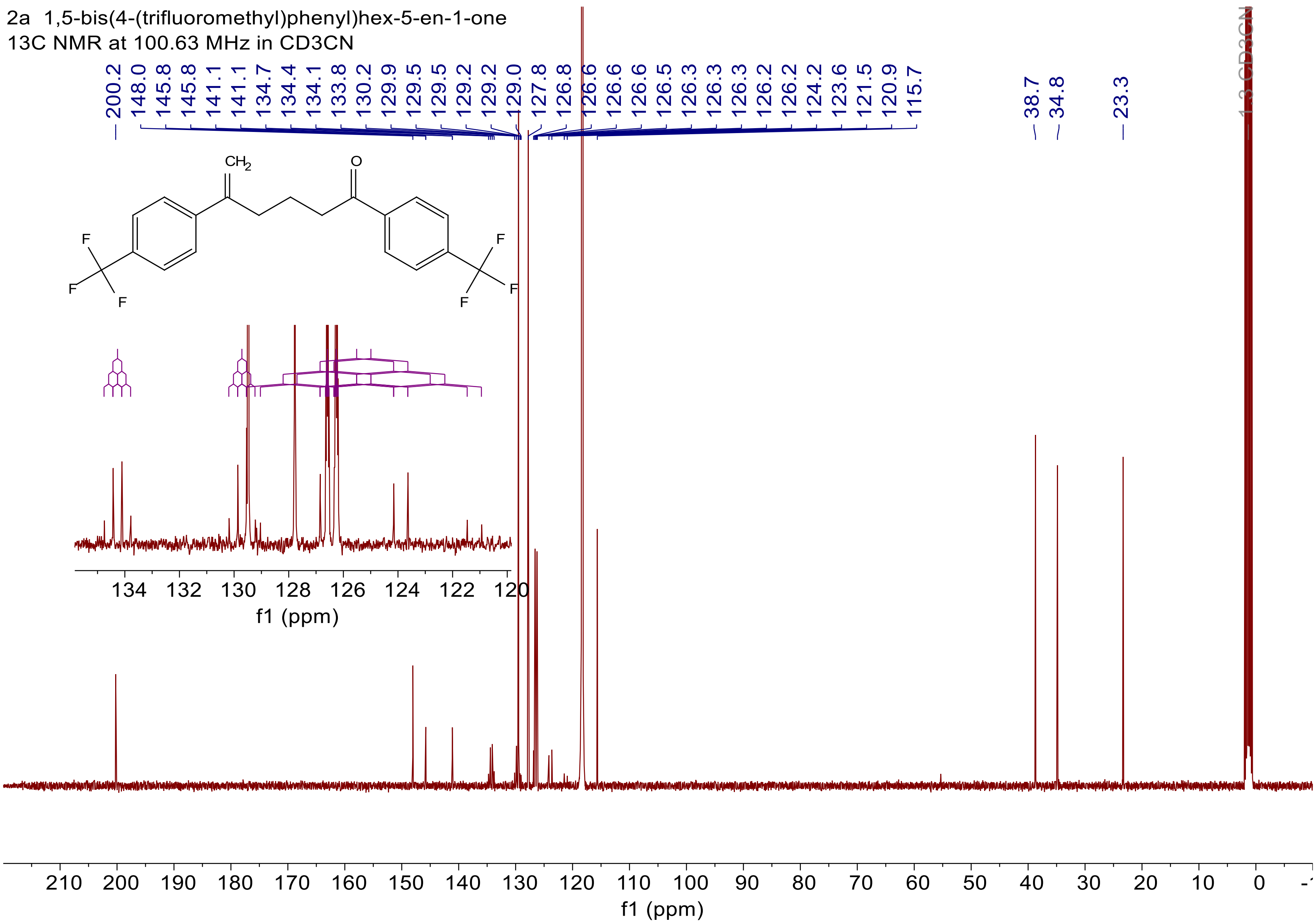

2a 1,5-bis(4-(trifluoromethyl)phenyl)hex-5-en-1-one
13C NMR at 100.63 MHz in CD3CN
200.2
148.0
145.8
145.8
141.1
141.1
134.7
134.4
134.1
133.8
130.2
129.9
129.5
129.5
129.2
129.2
129.0
127.8
126.8
126.6
126.6
126.6
126.5
126.3
126.3
126.3
126.2
126.2
124.2
123.6
121.5
120.9
115.7
38.7
34.8
23.3
1.3 CD3CN
CH2
O
F
F
F
F
F
F
134 132 130 128 126 124 122 120
f1 (ppm)
210 200 190 180 170 160 150 140 130 120 110 100 90 80 70 60 50 40 30 20 10 0
f1 (ppm)

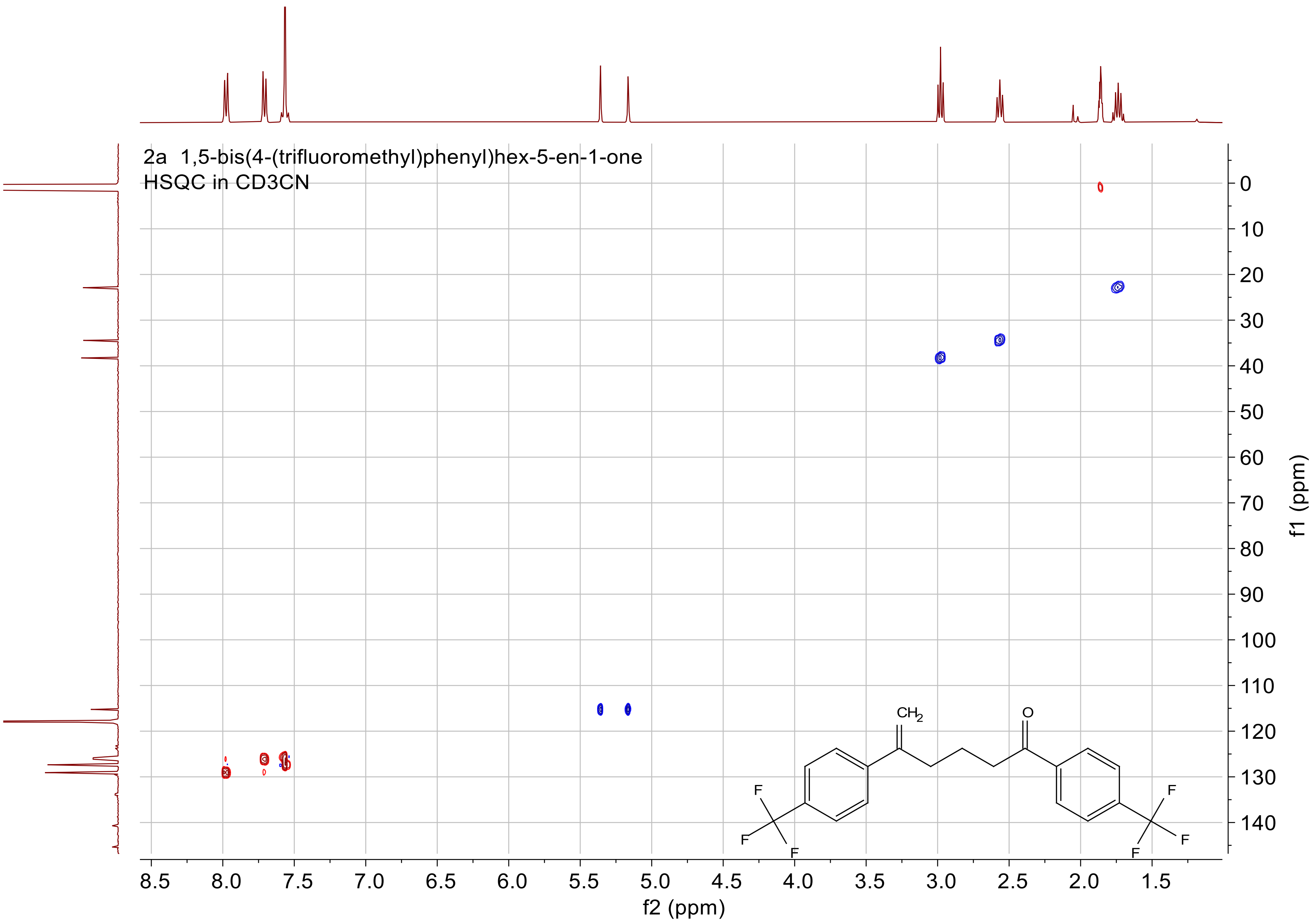
2a 1,5-bis(4-(trifluoromethyl)phenyl)hex-5-en-1-one
HSQC in CD3CN
f2 (ppm)
f1 (ppm)
CH2
O
F
F
F
F
F
F

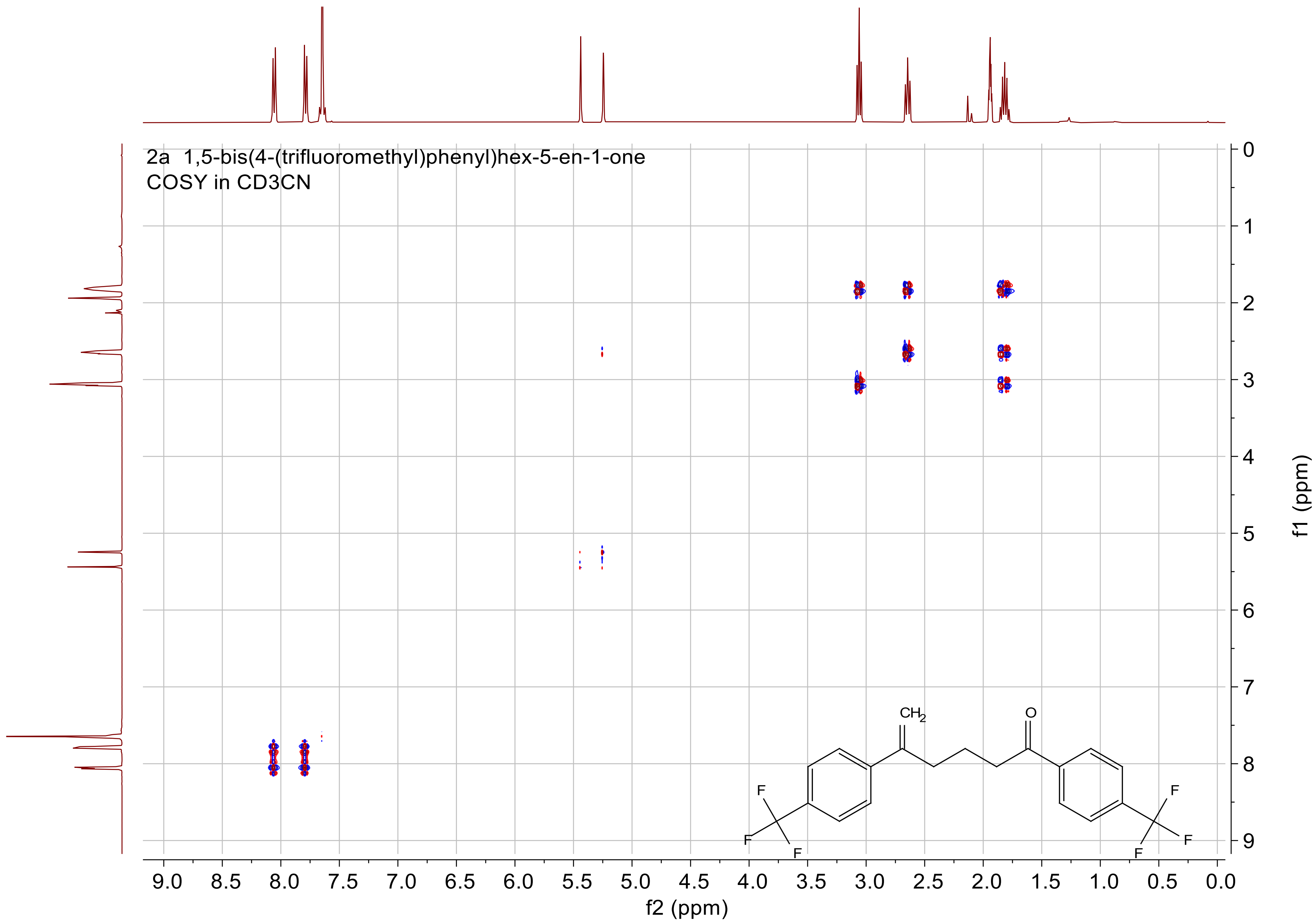
2a 1,5-bis(4-(trifluoromethyl)phenyl)hex-5-en-1-one
COSY in CD3CN
f2 (ppm)
f1 (ppm)
CH2
O
F
F
F
F
F
F

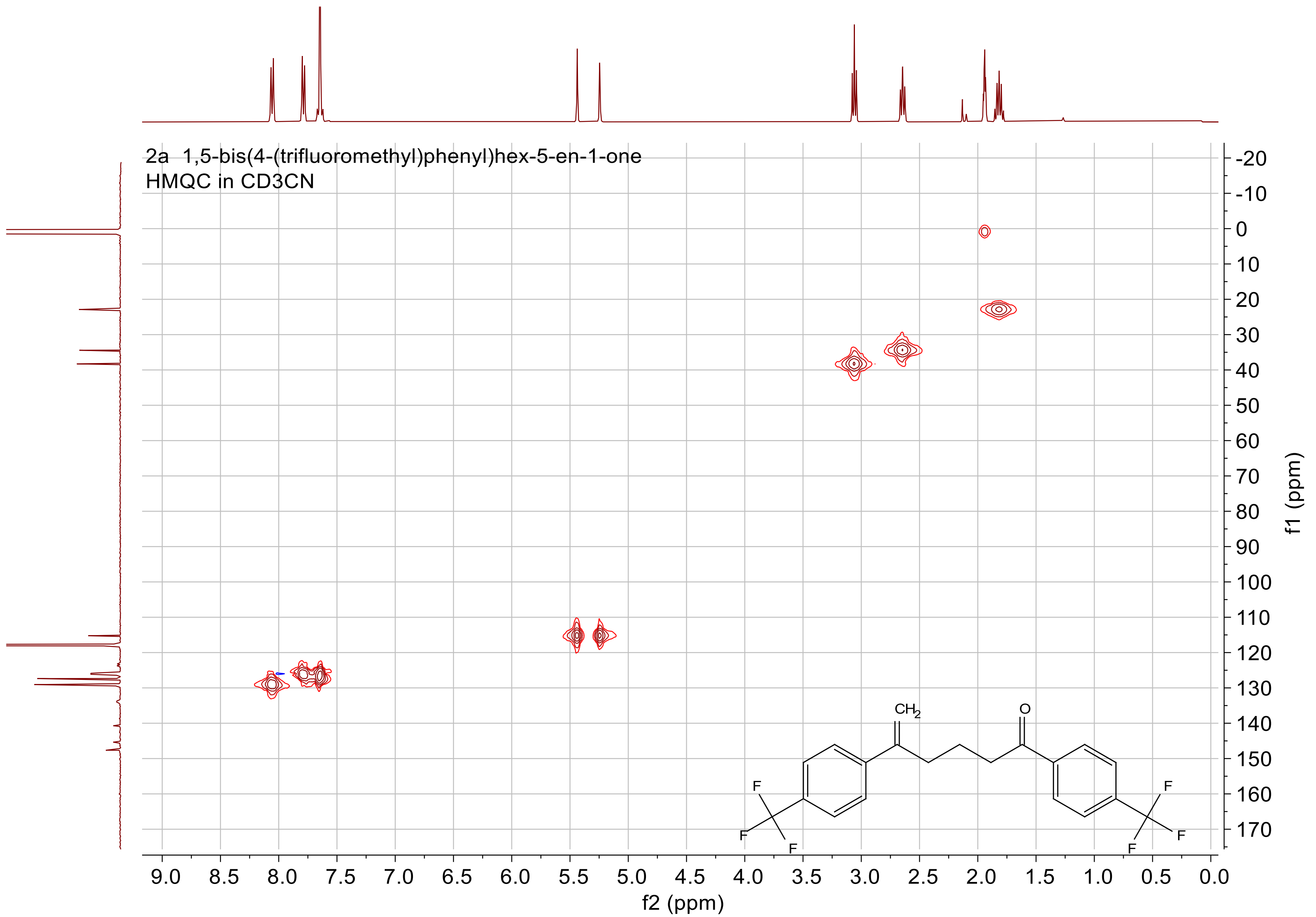
2a 1,5-bis(4-(trifluoromethyl)phenyl)hex-5-en-1-one
HMQC in CD3CN
f1 (ppm)
f2 (ppm)

2a 1,5-bis(4-(trifluoromethyl)phenyl)hex-5-en-1-one

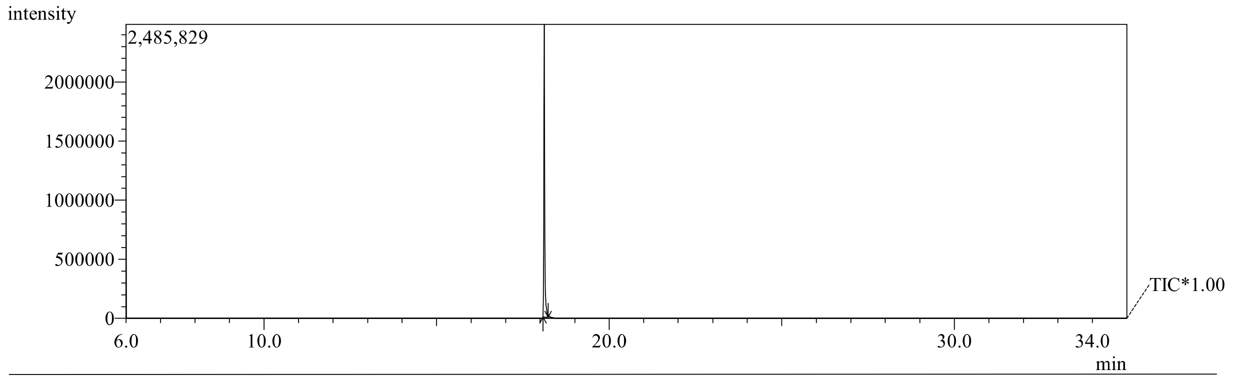

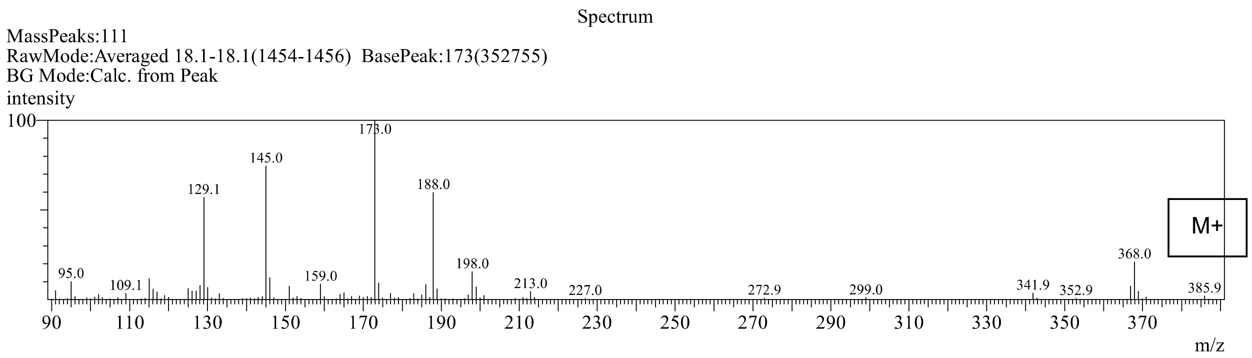

2b 1-phenyl-5-(4-(trifluoromethyl)phenyl)hex-5-en-1-one
1H NMR at 400.15 MHz in CD3CN

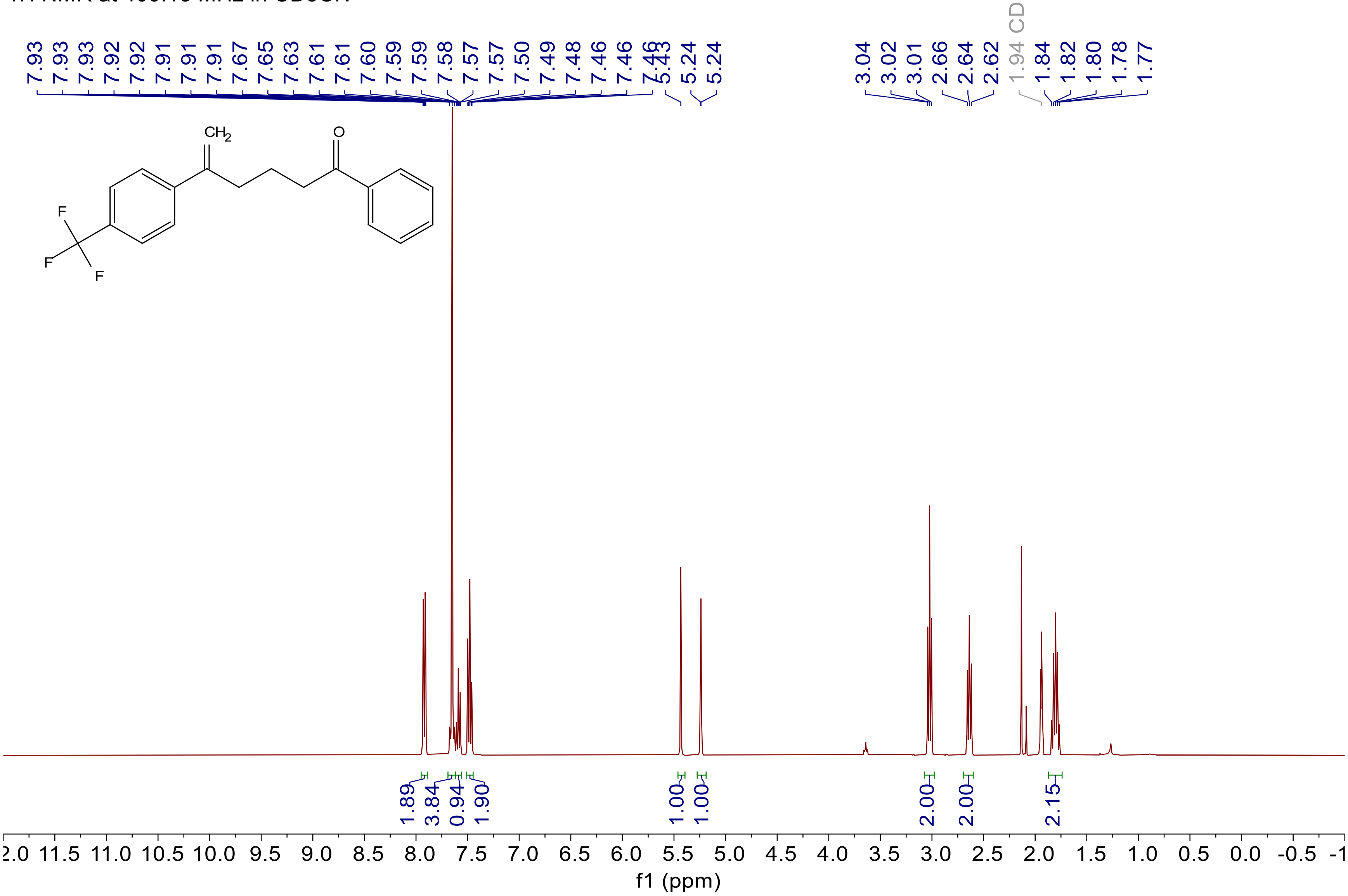

2b 1-phenyl-5-(4-(trifluoromethyl)phenyl)hex-5-en-1-one
19F NMR at 376.48 MHz in CD3CN

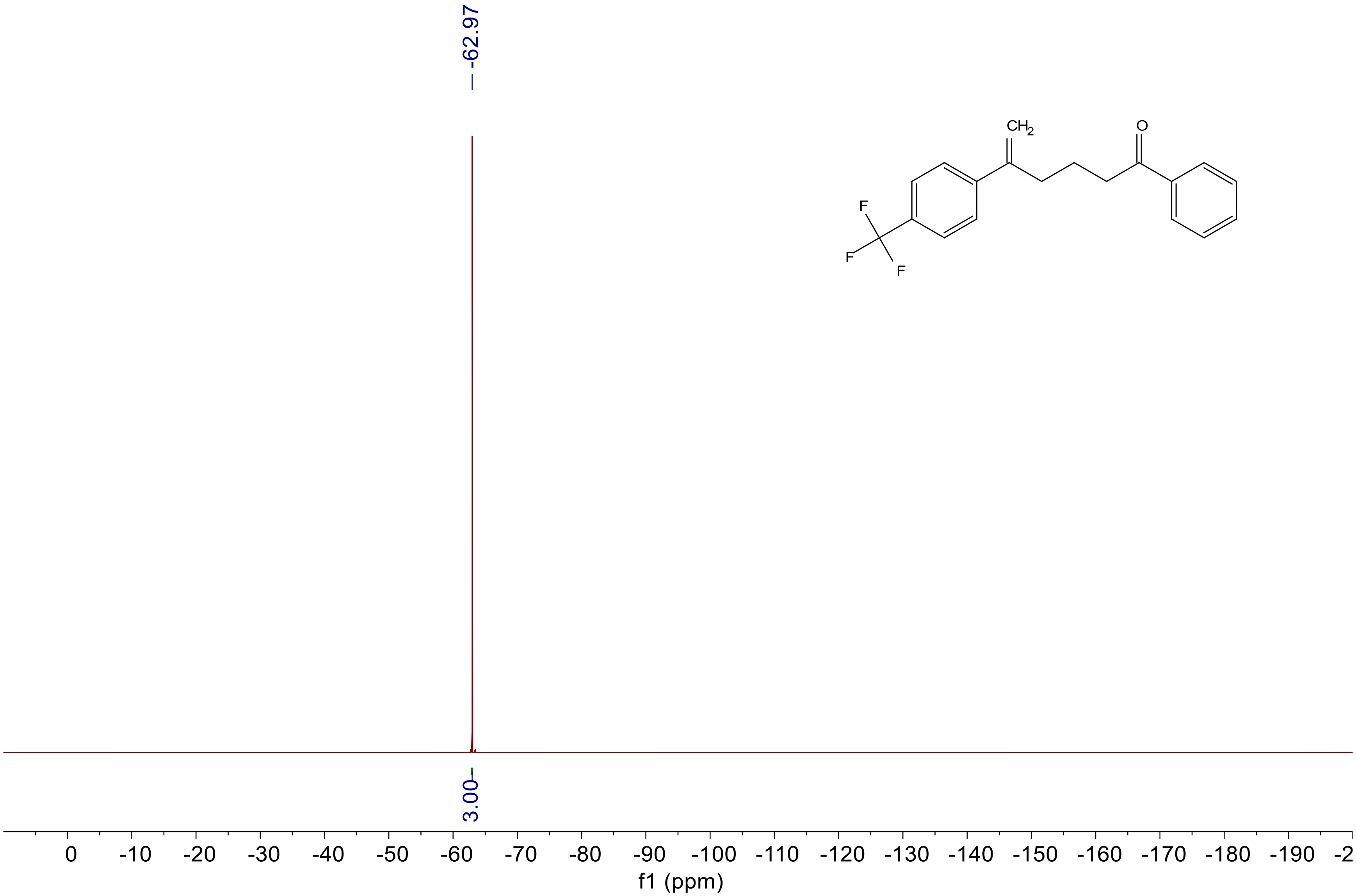

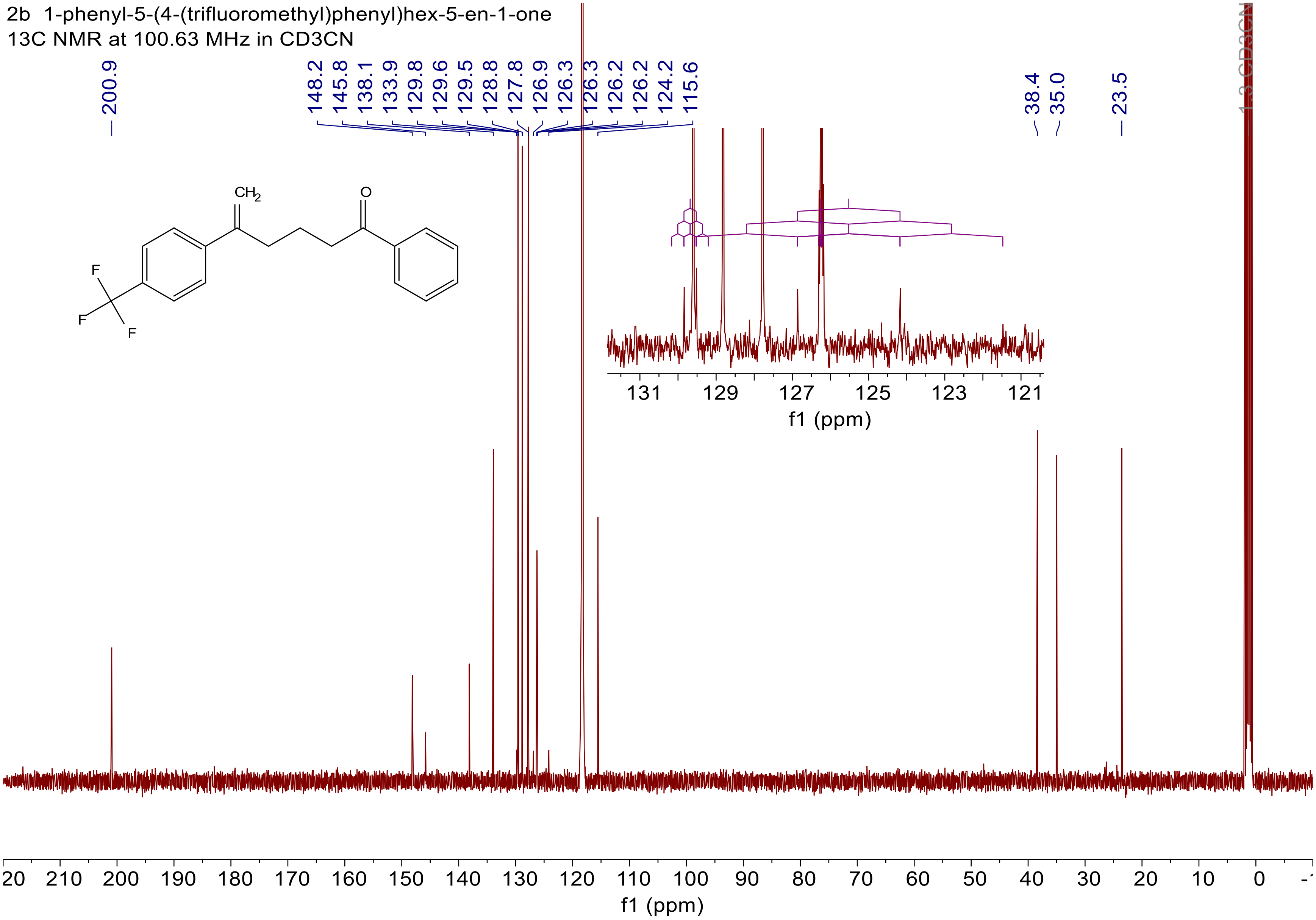
2b 1-phenyl-5-(4-(trifluoromethyl)phenyl)hex-5-en-1-one
13C NMR at 100.63 MHz in CD3CN
200.9
148.2
145.8
138.1
133.9
129.8
129.6
129.5
128.8
127.8
126.9
126.3
126.3
126.2
126.2
124.2
115.6
38.4
35.0
23.5
1.3 CD3CN
CH2
O
F
F
F
131
129
127
125
123
121
f1 (ppm)
20
210
200
190
180
170
160
150
140
130
120
110
100
90
80
70
60
50
40
30
20
10
0
f1 (ppm)

2b 1-phenyl-5-(4-(trifluoromethyl)phenyl)hex-5-en-1-one

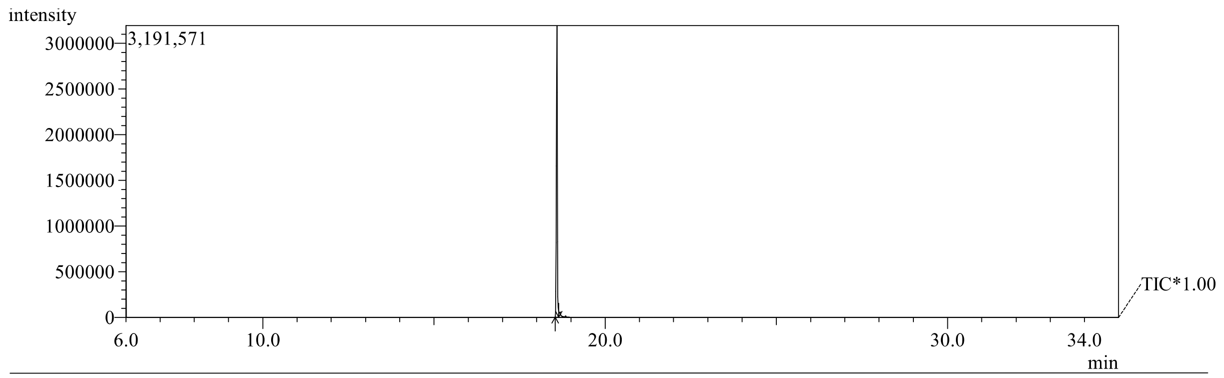

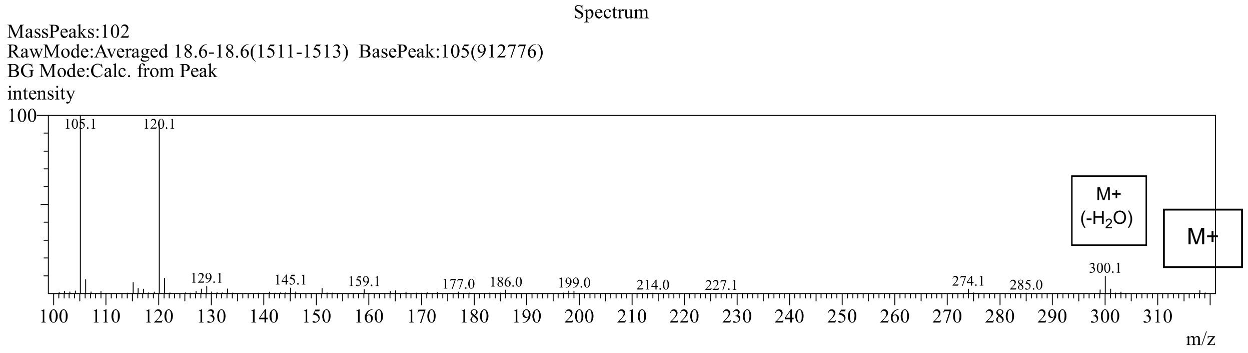

2c 5-phenyl-1-(4-(trifluoromethyl)phenyl)hex-5-en-1-one
1H NMR at 400.15 MHz in CD3CN

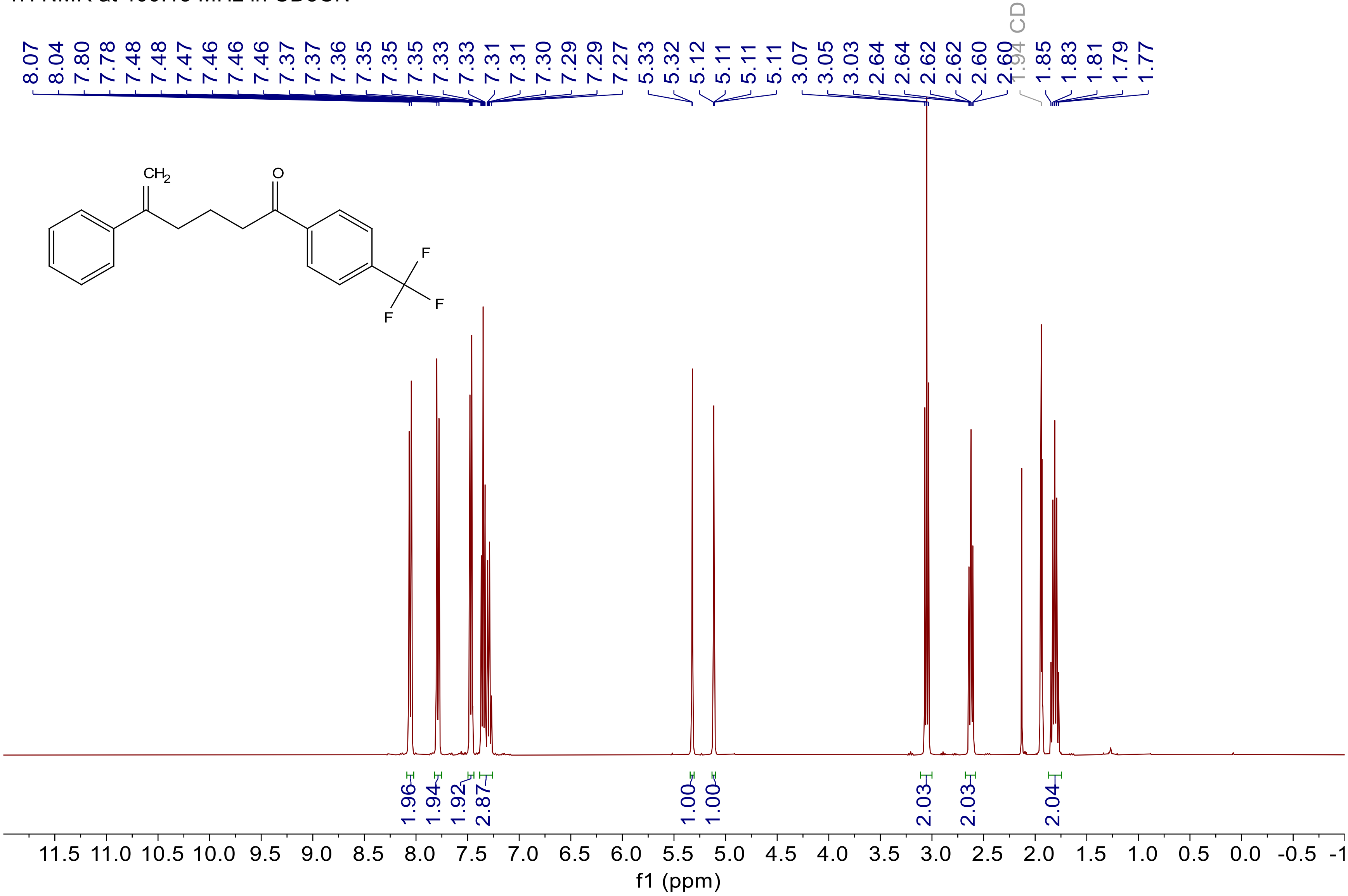

2c 5-phenyl-1-(4-(trifluoromethyl)phenyl)hex-5-en-1-one
19F NMR at 376.48 MHz in CD3CN

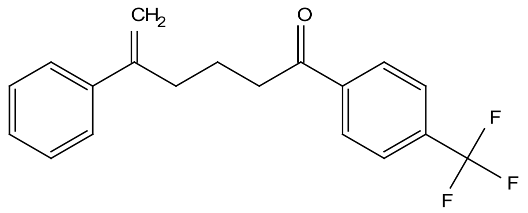

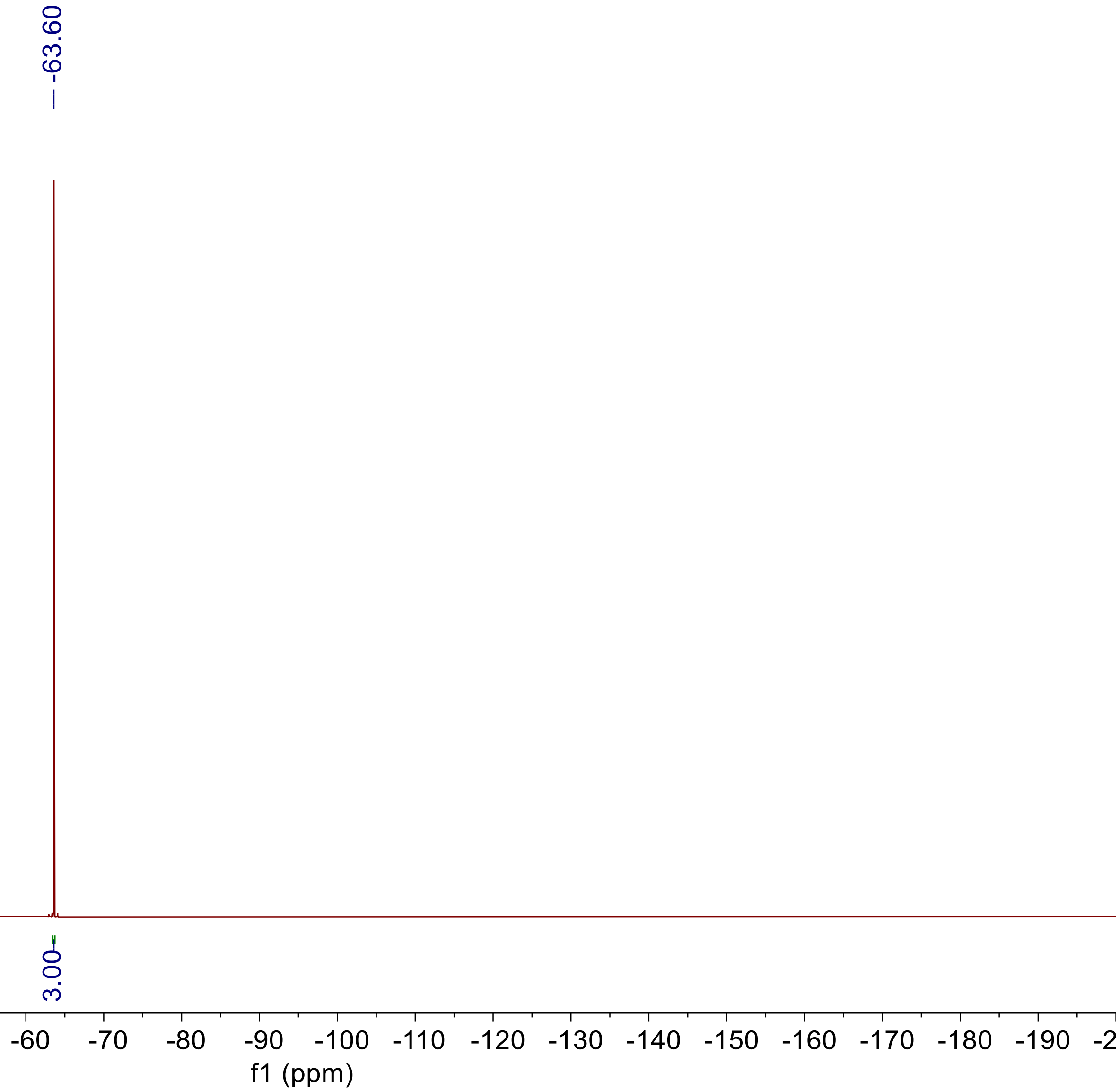

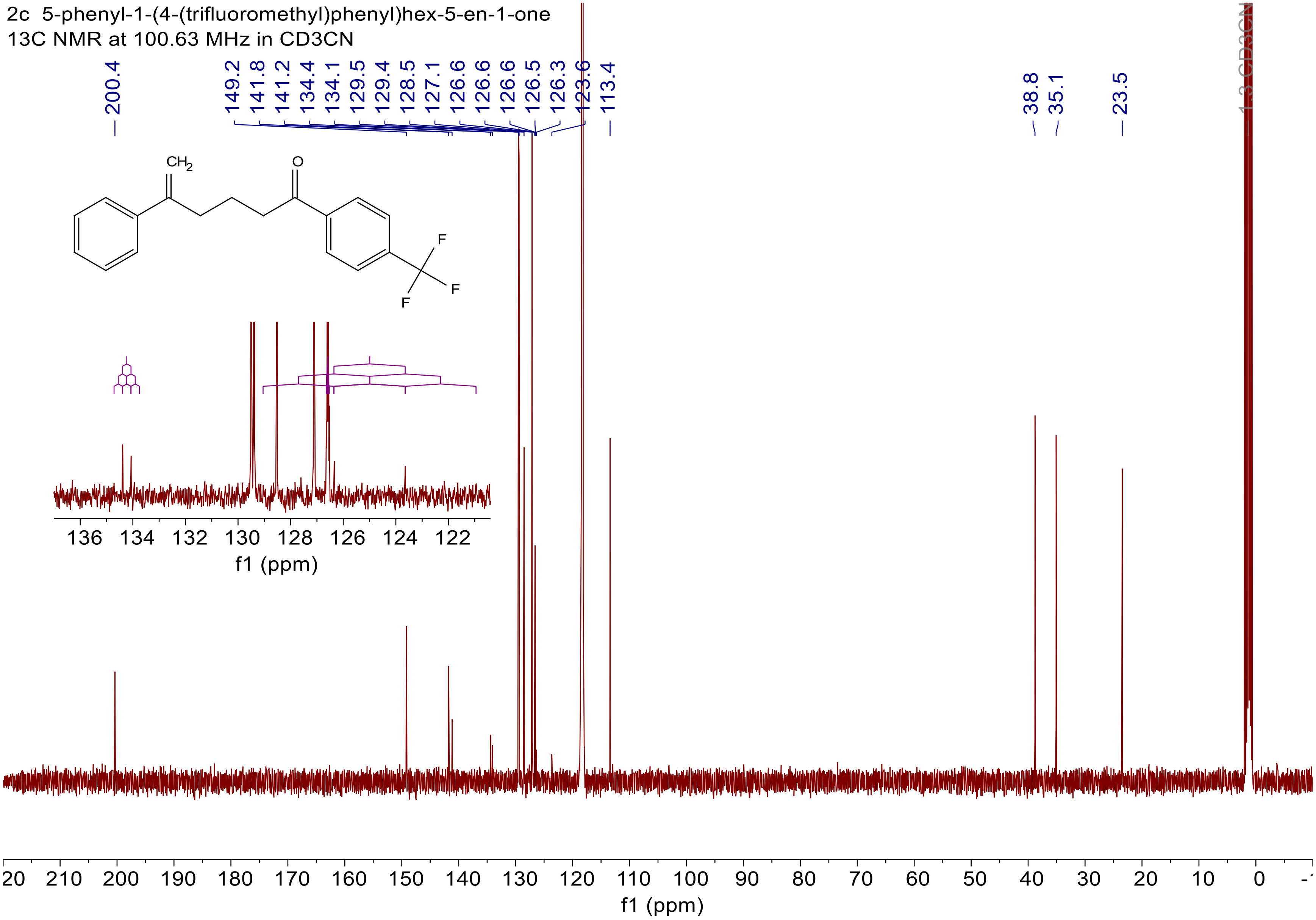

2c 5-phenyl-1-(4-(trifluoromethyl)phenyl)hex-5-en-1-one
13C NMR at 100.63 MHz in CD3CN
200.4
149.2
141.8
141.2
134.4
134.1
129.5
129.4
128.5
127.1
126.6
126.6
126.6
126.5
126.3
123.6
113.4
38.8
35.1
23.5
1.3 CD3CN
CH2
O
F
F
F
136 134 132 130 128 126 124 122
f1 (ppm)
20 210 200 190 180 170 160 150 140 130 120 110 100 90 80 70 60 50 40 30 20 10 0
f1 (ppm)

2c 5-phenyl-1-(4-(trifluoromethyl)phenyl)hex-5-en-1-one

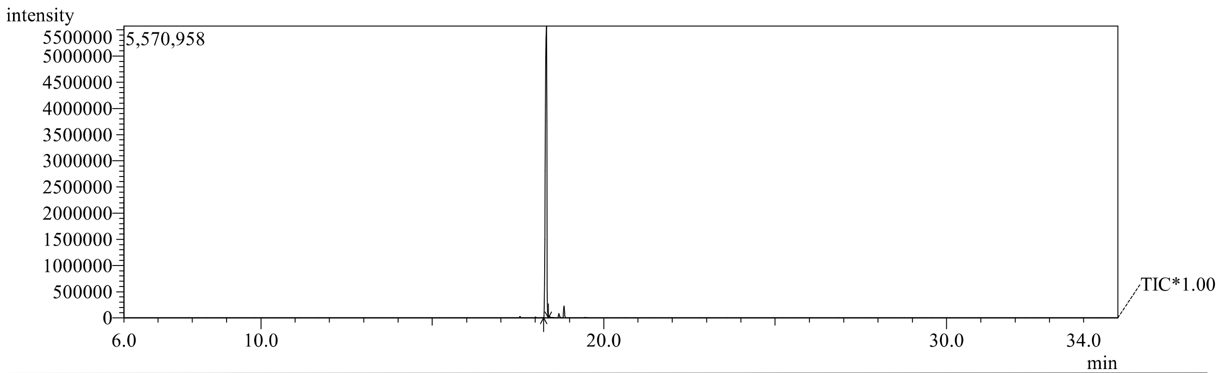

Spectrum

MassPeaks:145
RawMode:Averaged 18.3-18.3(1480-1482) BasePeak:130(807206)
BG Mode:Calc. from Peak

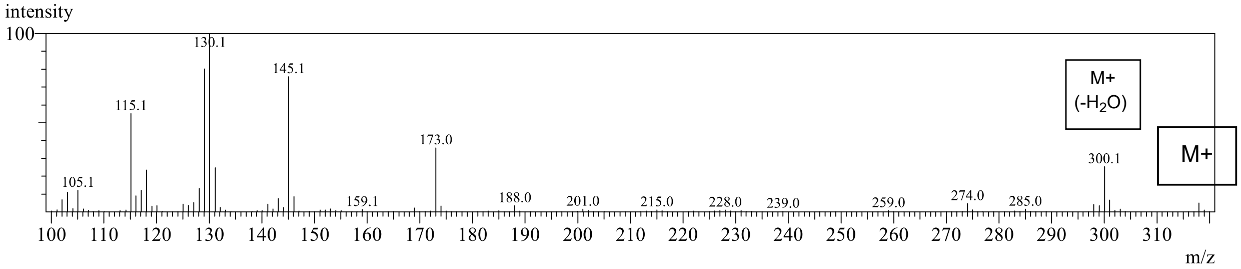

2d 1,5-diphenylhex-5-en-1-one
1H NMR at 400.15 MHz in CDCl3

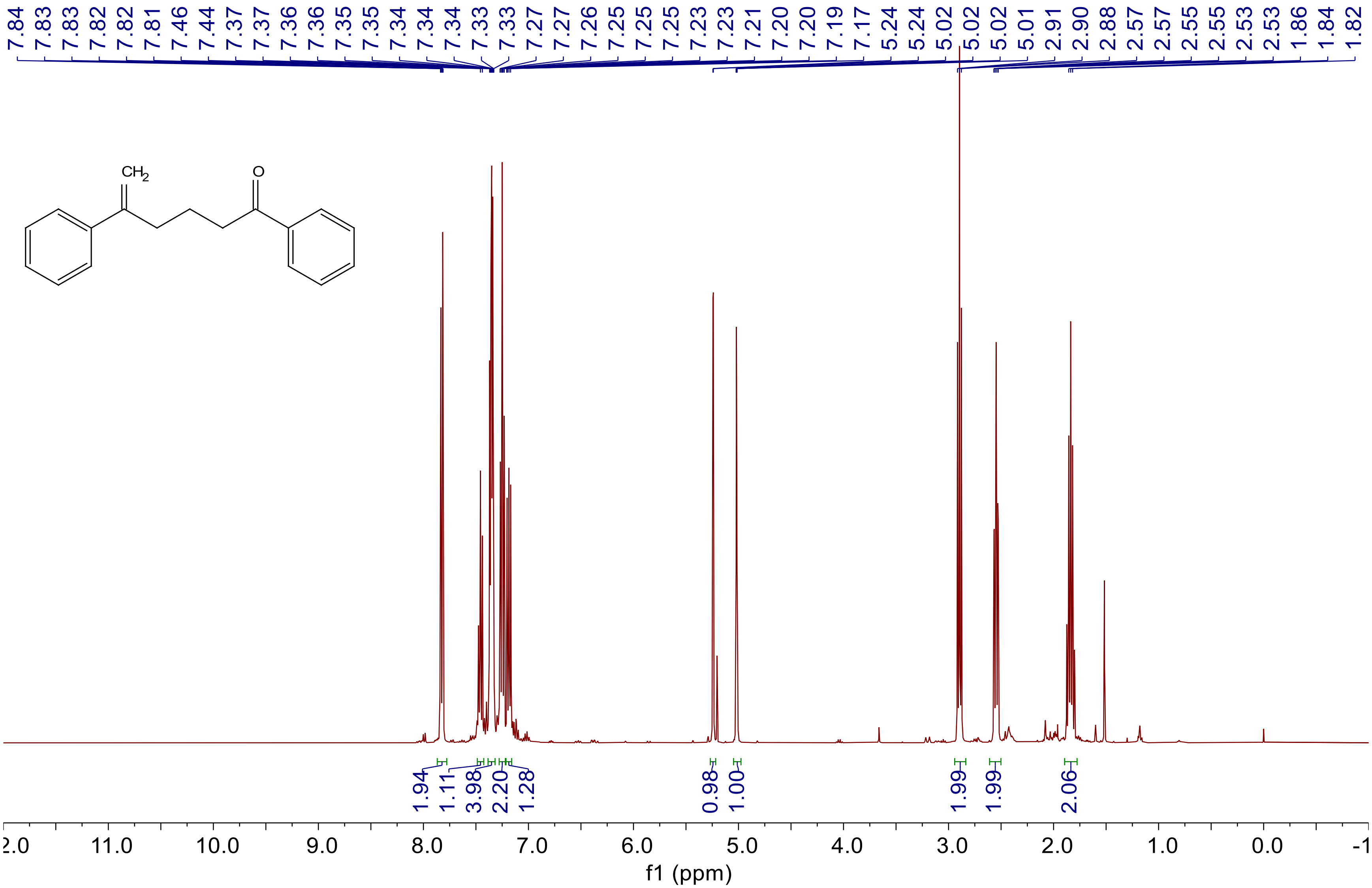

2d 1,5-diphenylhex-5-en-1-one
13C NMR at 100.63 MHz in CDCl3

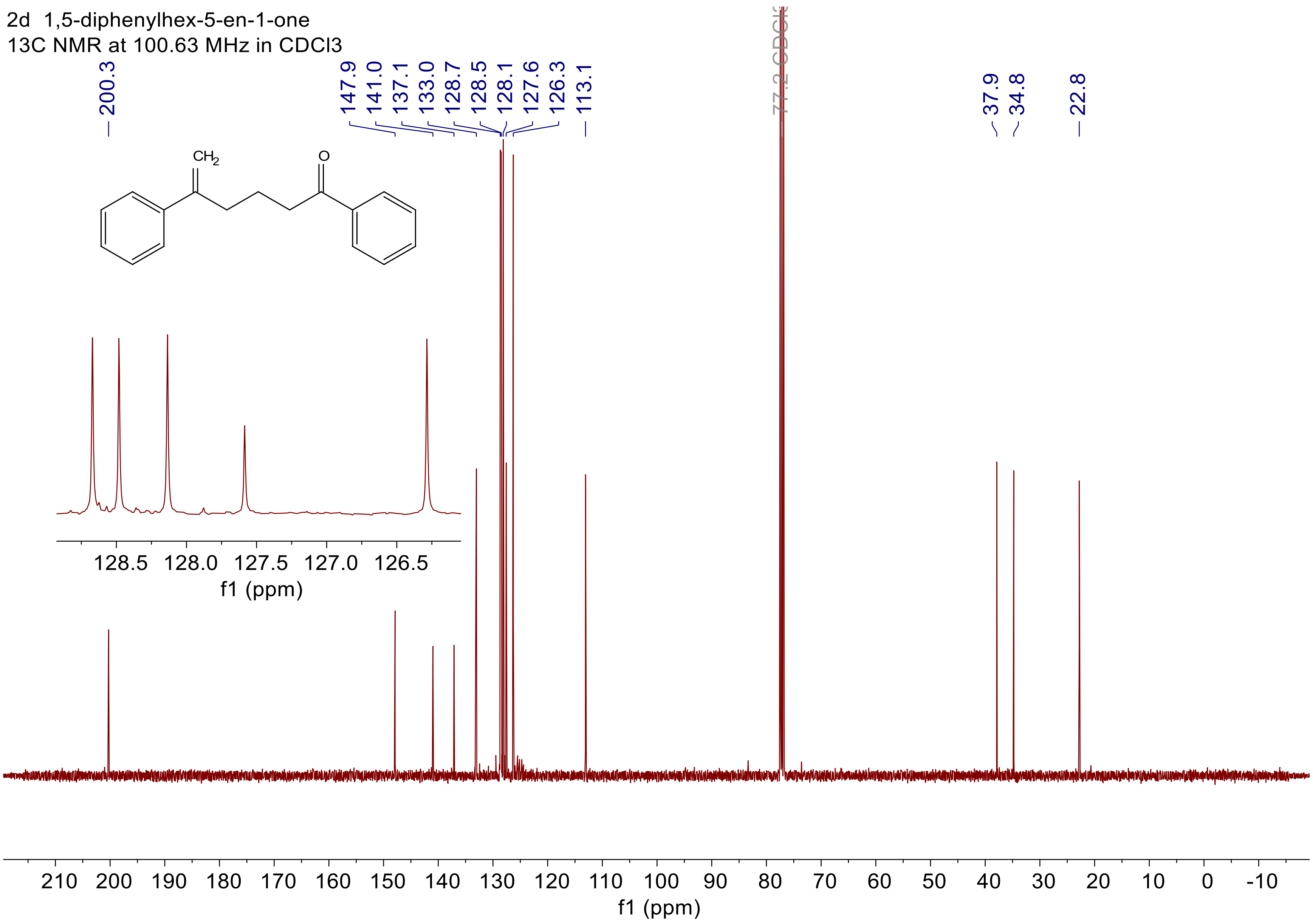

2d 1,5-diphenylhex-5-en-1-one

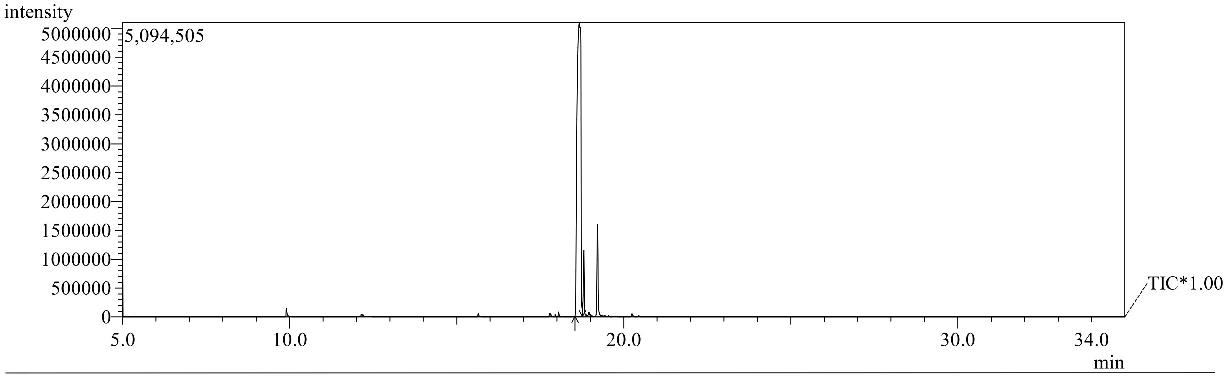

Spectrum

MassPeaks:90
RawMode:Averaged 18.7-18.7(1641-1643) BasePeak:105(1160473)
BG Mode:Calc. from Peak

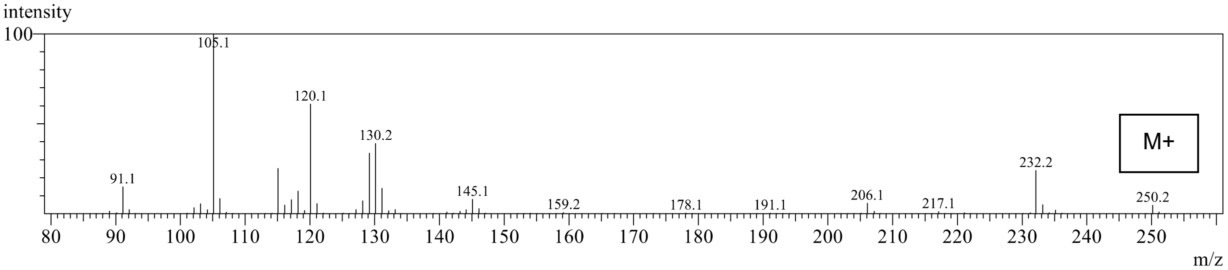

2e 6-phenylhept-6-en-2-one
1H NMR at 400.15 MHz in CD2Cl2

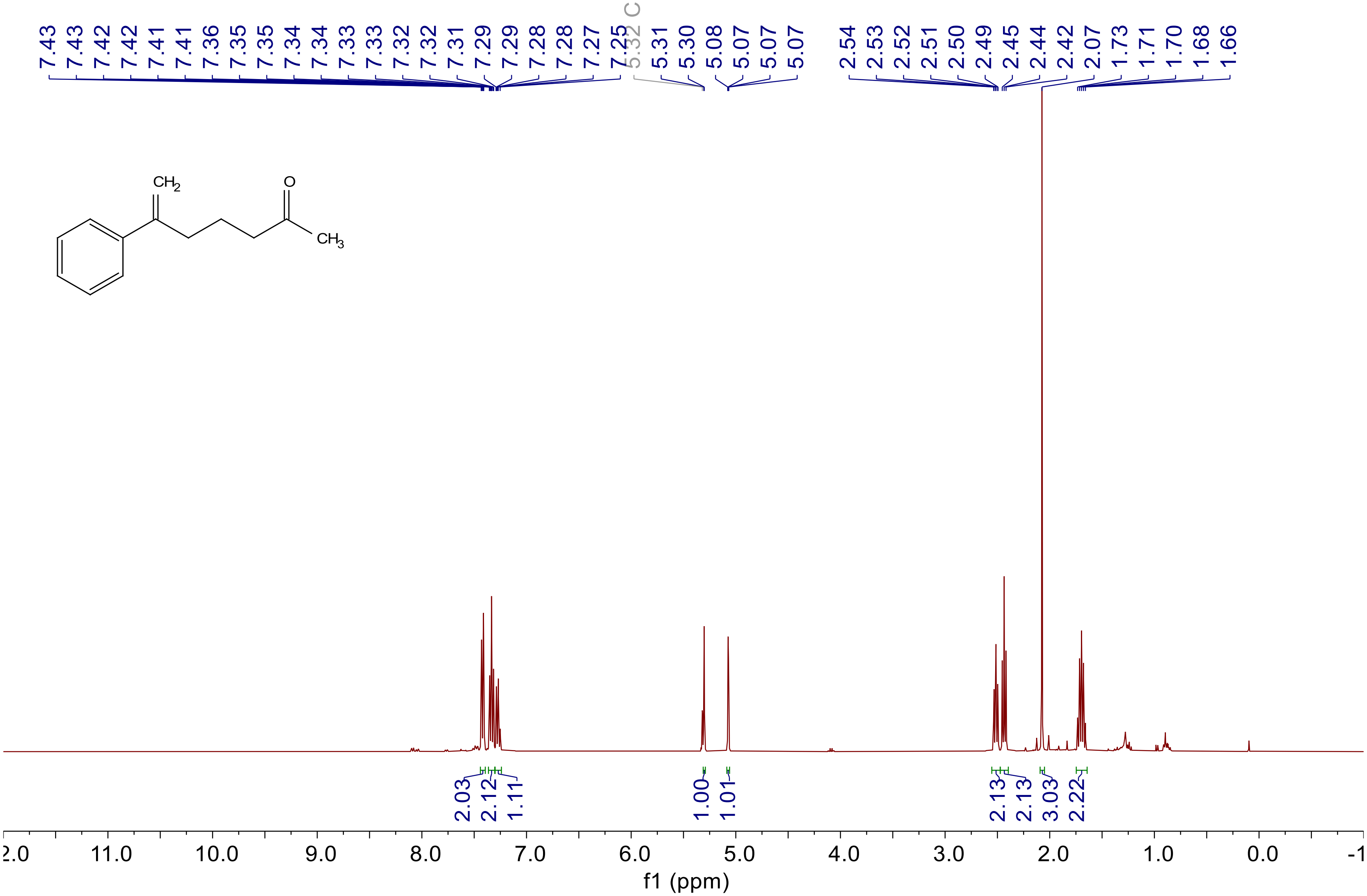

2e 6-phenylhept-6-en-2-one
13C NMR at 100.63 MHz in CD2Cl2

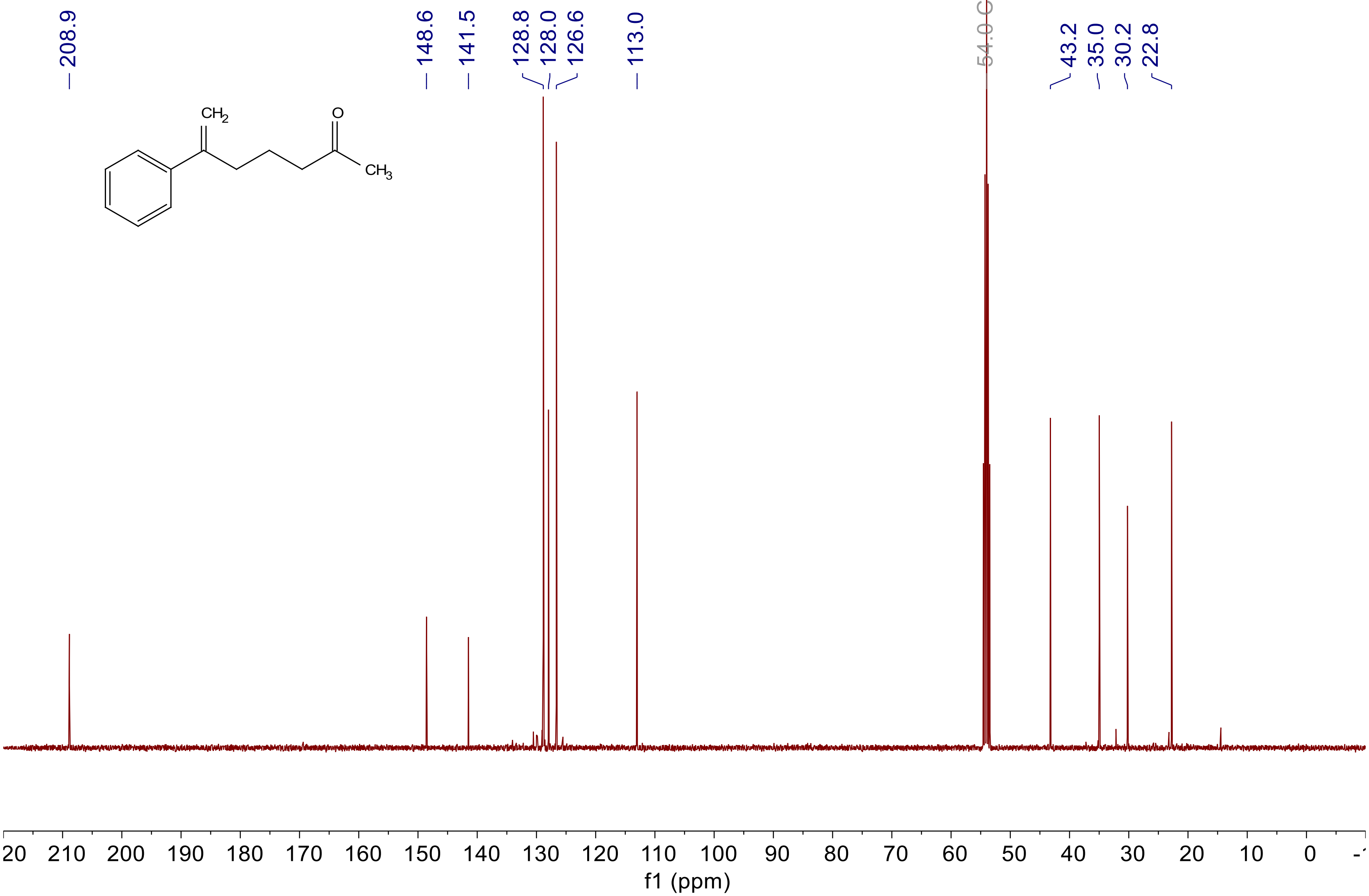

2e 6-phenylhept-6-en-2-one

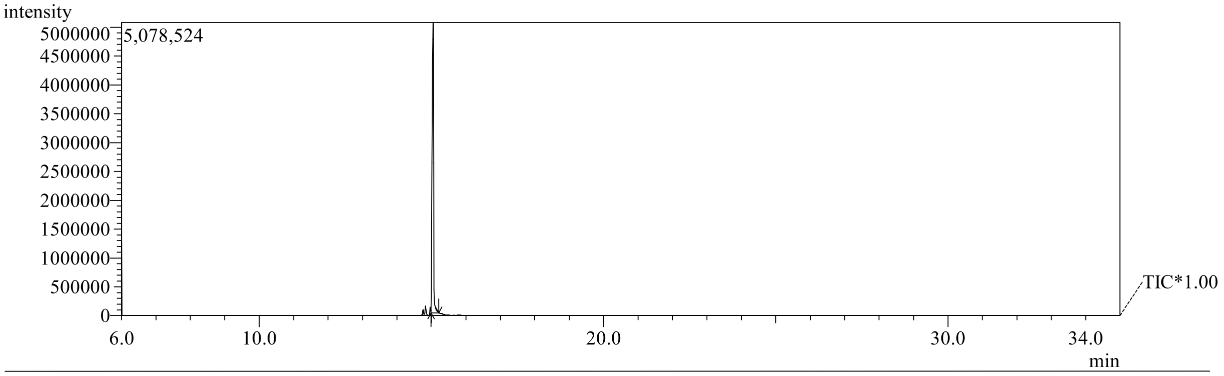

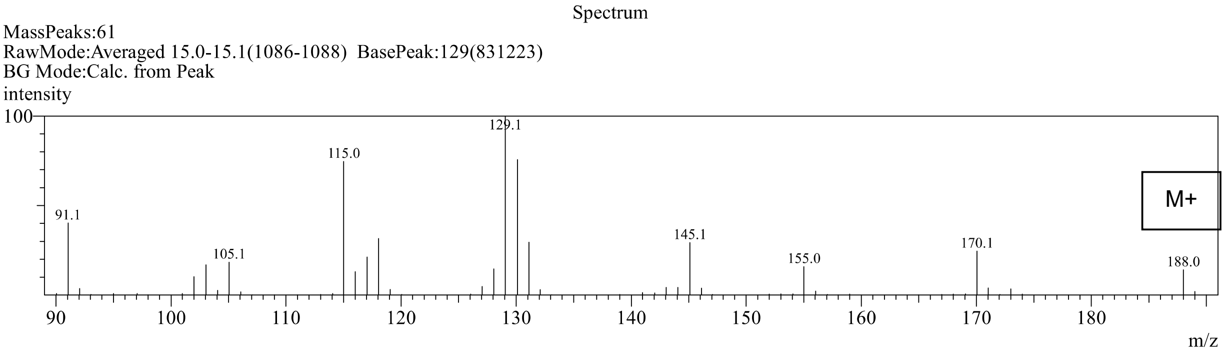

2f 8-(4-(trifluoromethyl)phenyl)nona-1,8-dien-4-one
1H NMR at 400.15 MHz in CDCl3

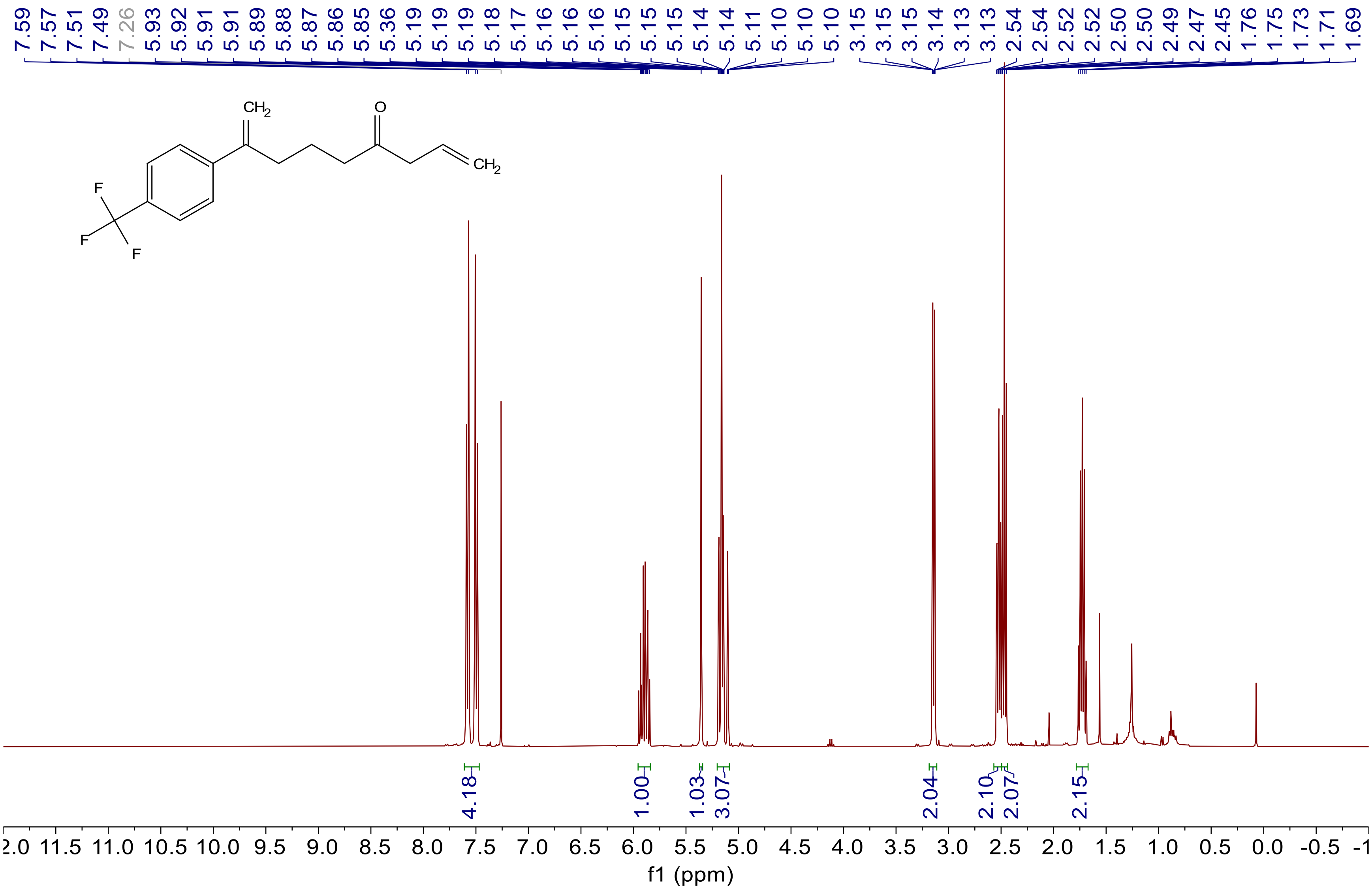

2f 8-(4-(trifluoromethyl)phenyl)nona-1,8-dien-4-one
19F NMR at 376.48 MHz in CDCl3

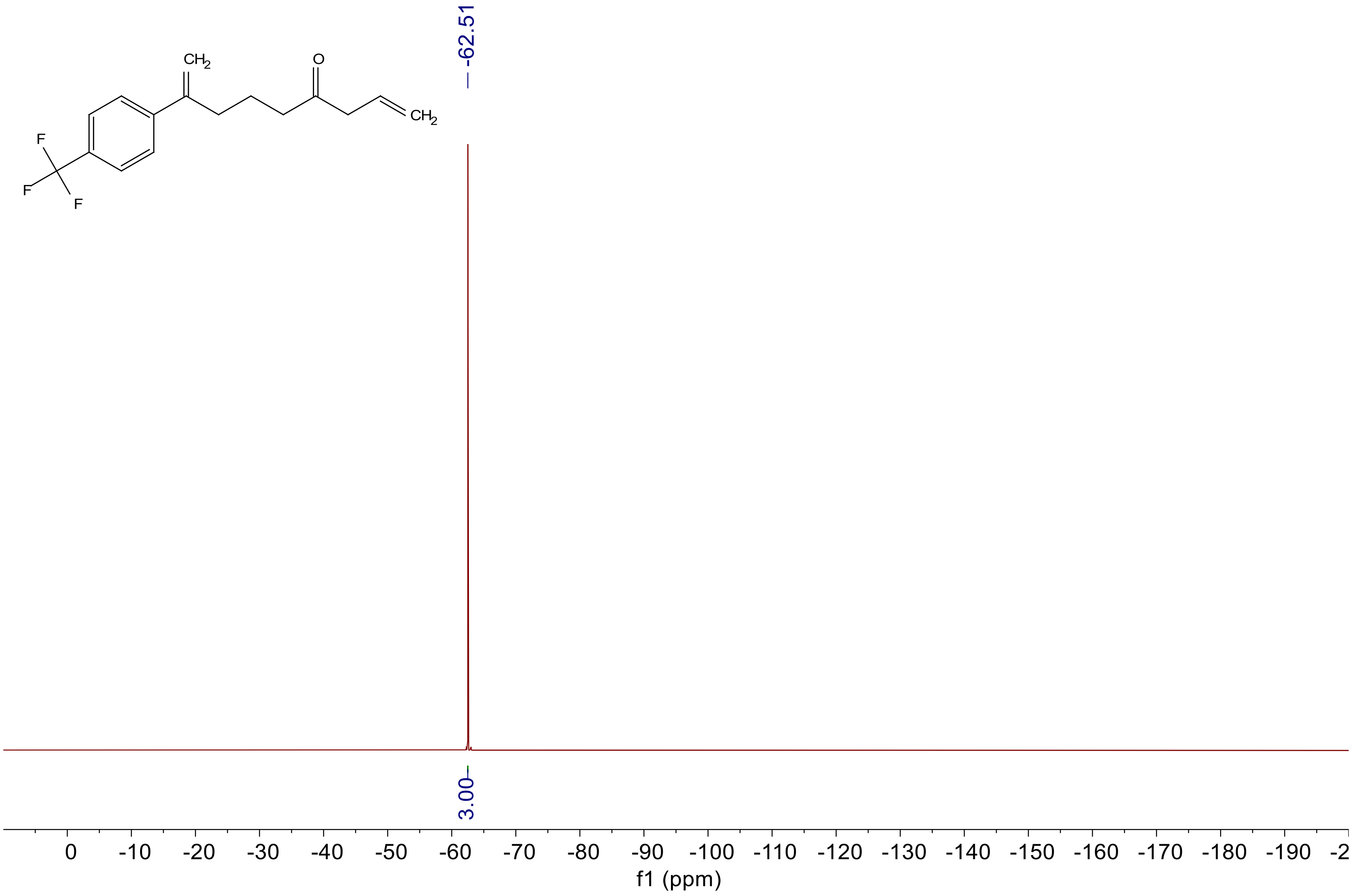

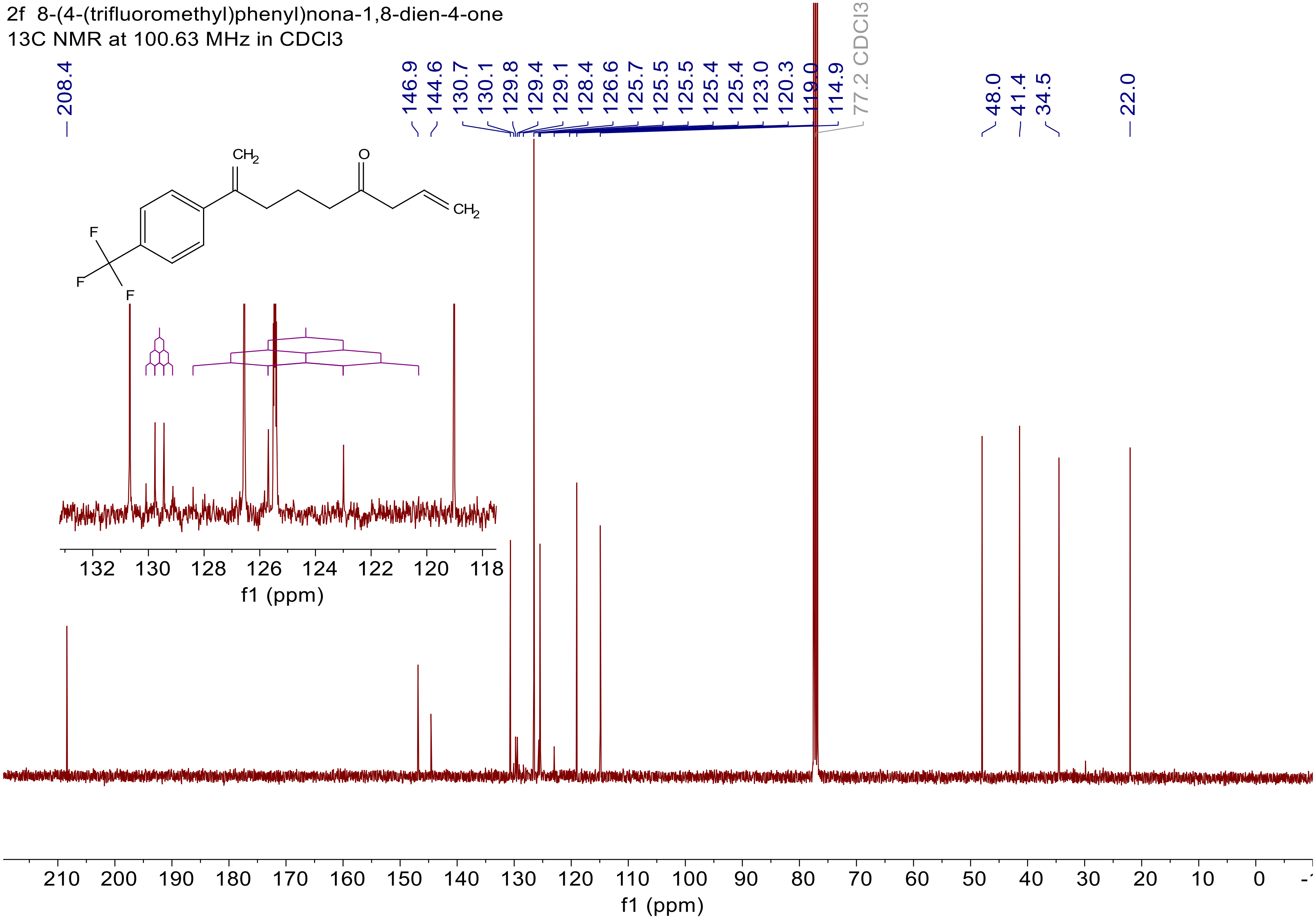

2f 8-(4-(trifluoromethyl)phenyl)nona-1,8-dien-4-one
13C NMR at 100.63 MHz in CDCl3
208.4
146.9
144.6
130.7
130.1
129.8
129.4
129.1
128.4
126.6
125.7
125.5
125.5
125.4
125.4
123.0
120.3
119.0
114.9
77.2 CDCl3
48.0
41.4
34.5
22.0
f1 (ppm)

2f 8-(4-(trifluoromethyl)phenyl)nona-1,8-dien-4-one

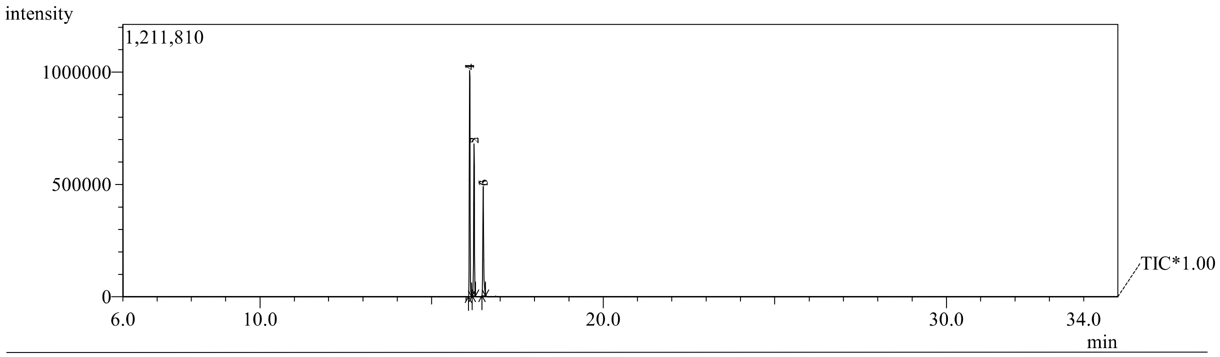

Spectrum

Line#:1 R.Time:16.1(Scan#:1214)
MassPeaks:100
RawMode:Averaged 16.1-16.1(1213-1215) BasePeak:241(97489)
BG Mode:Calc. from Peak

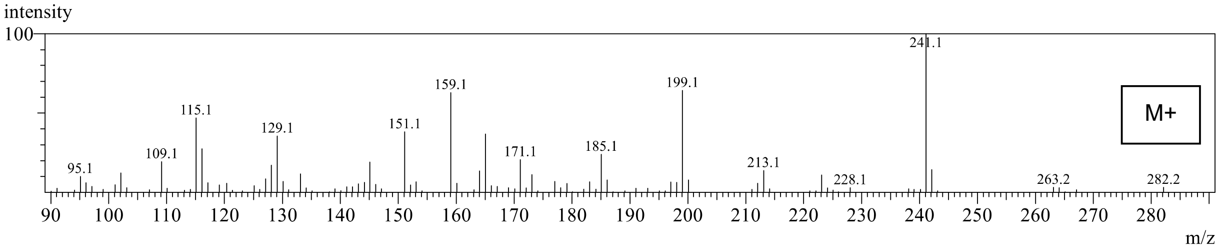

Line#:2 R.Time:16.2(Scan#:1229)
MassPeaks:111
RawMode:Averaged 16.2-16.2(1228-1230) BasePeak:109(37876)
BG Mode:Calc. from Peak

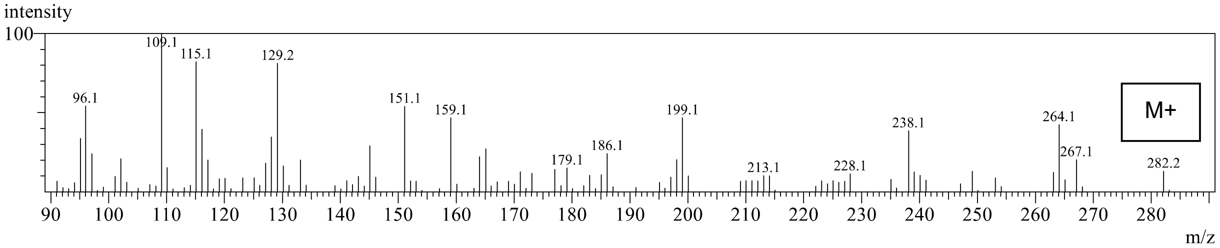

Line#:3 R.Time:16.5(Scan#:1261)
MassPeaks:97
RawMode:Averaged 16.5-16.5(1260-1262) BasePeak:109(25485)
BG Mode:Calc. from Peak

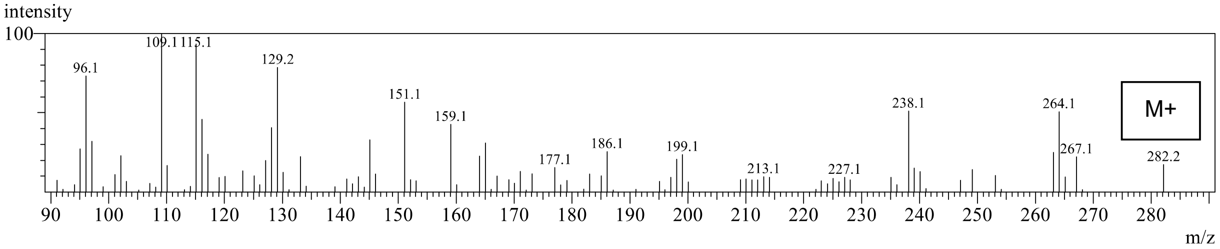

2g 1-(dimethylamino)-8-(4-(trifluoromethyl)phenyl)non-8-en-2-yn-4-one
1H NMR at 400.15 MHz in CD3CN

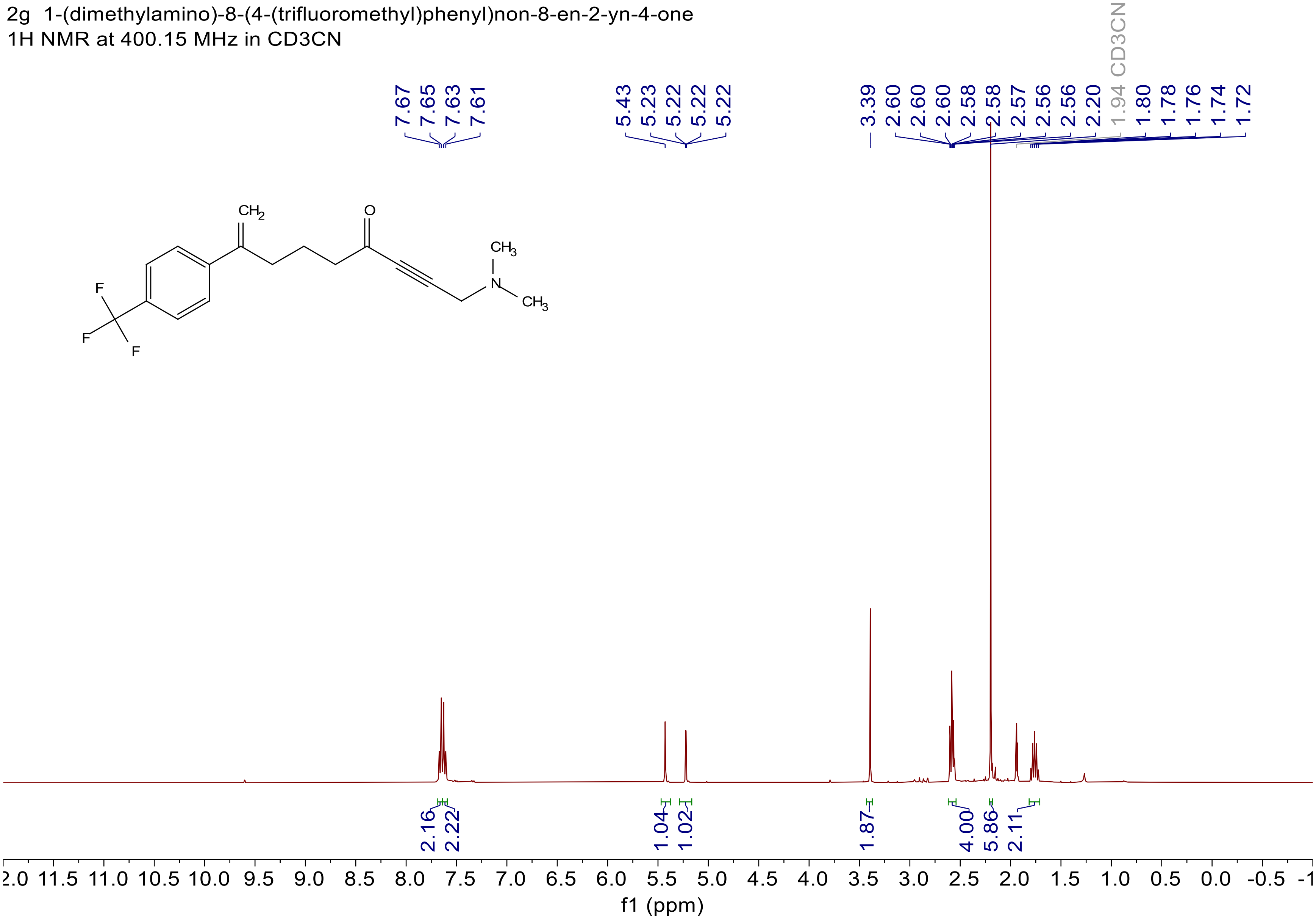

1k 4'-(trifluoromethyl)-3,4,5,6-tetrahydro-[1,1'-biphenyl]-3-ol
19F NMR at 376.48 MHz in CD3CN

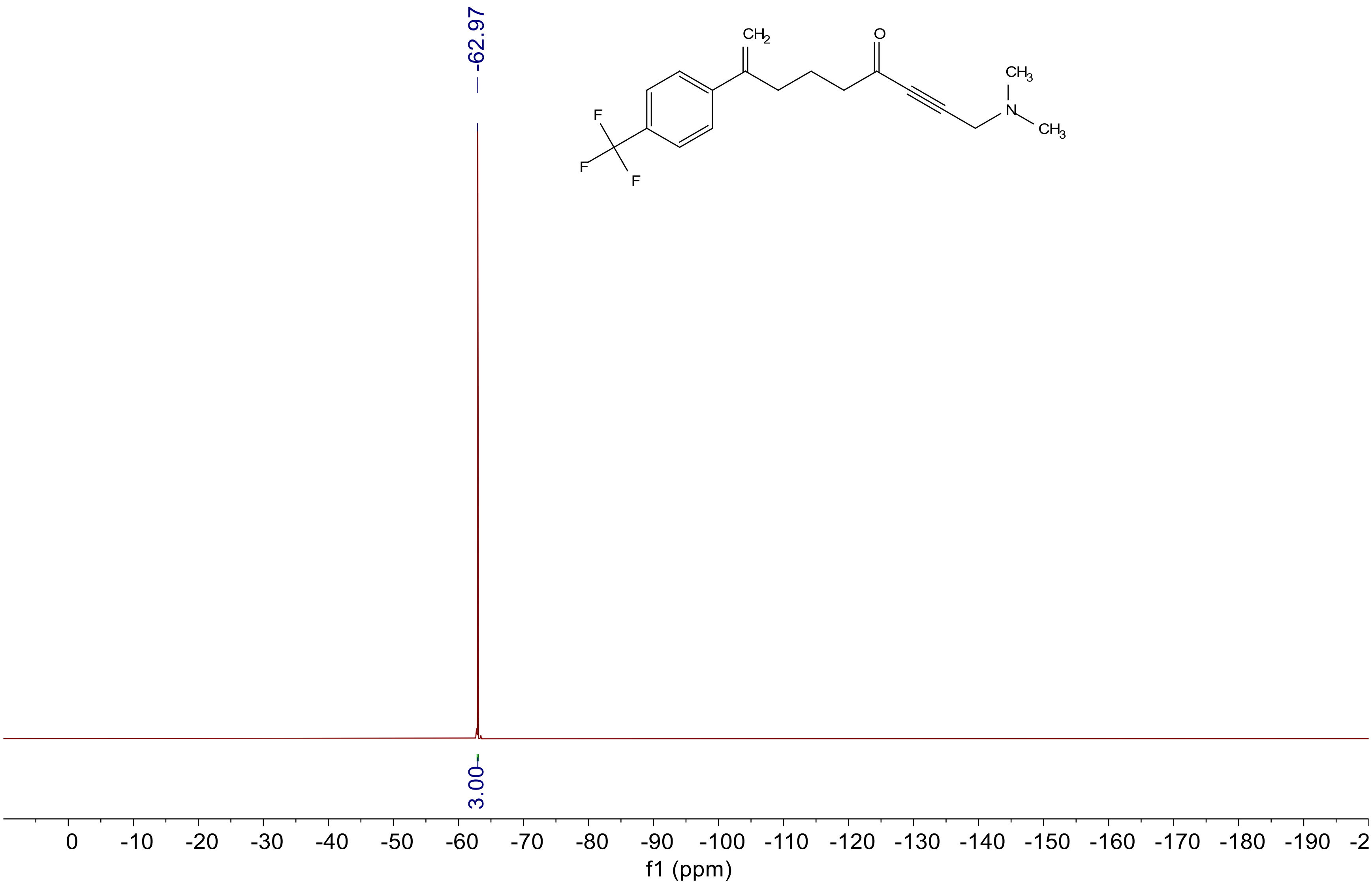

2g 1-(dimethylamino)-8-(4-(trifluoromethyl)phenyl)non-8-en-2-yn-4-one
13C NMR at 100.63 MHz in CD3CN

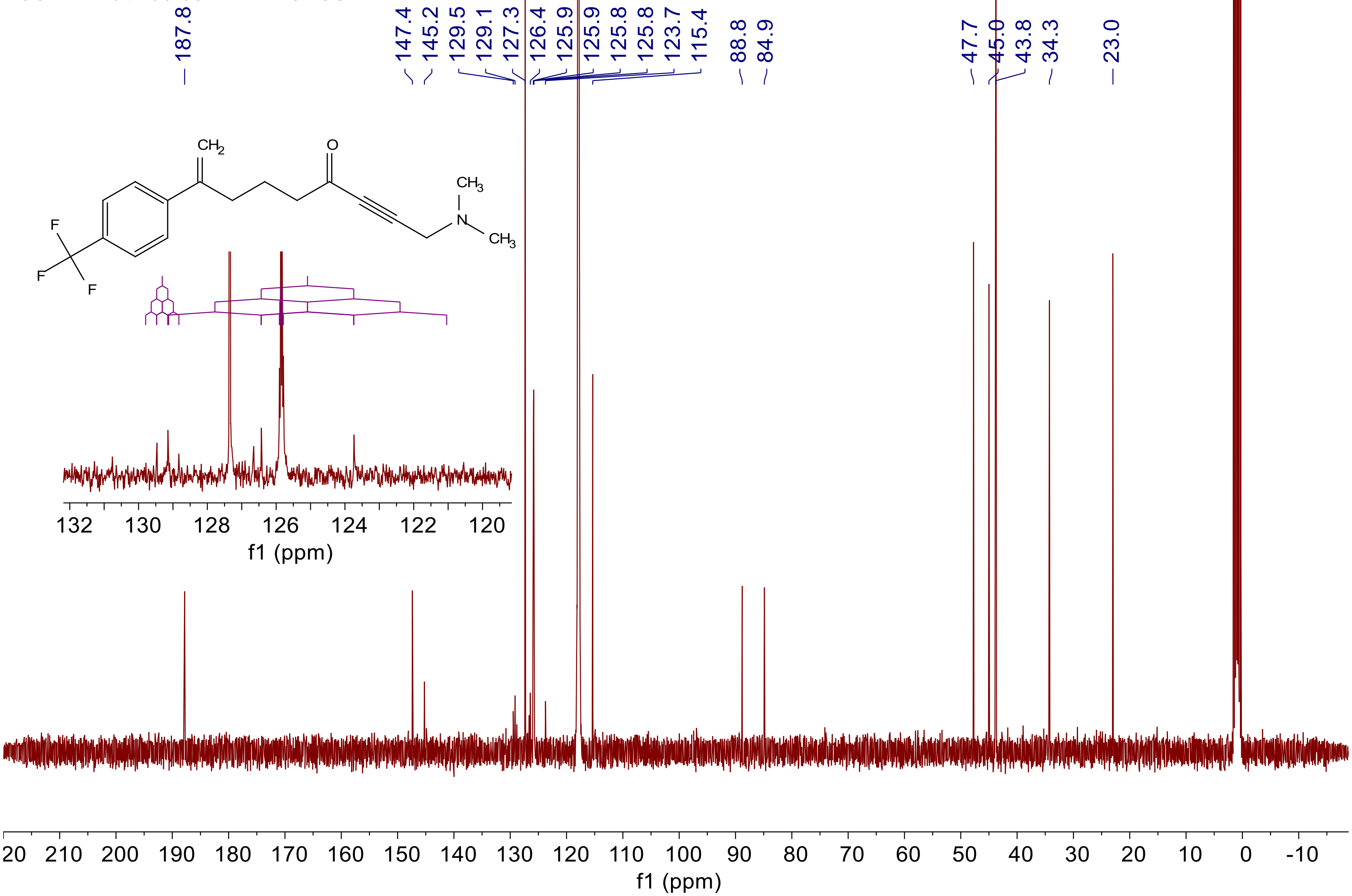

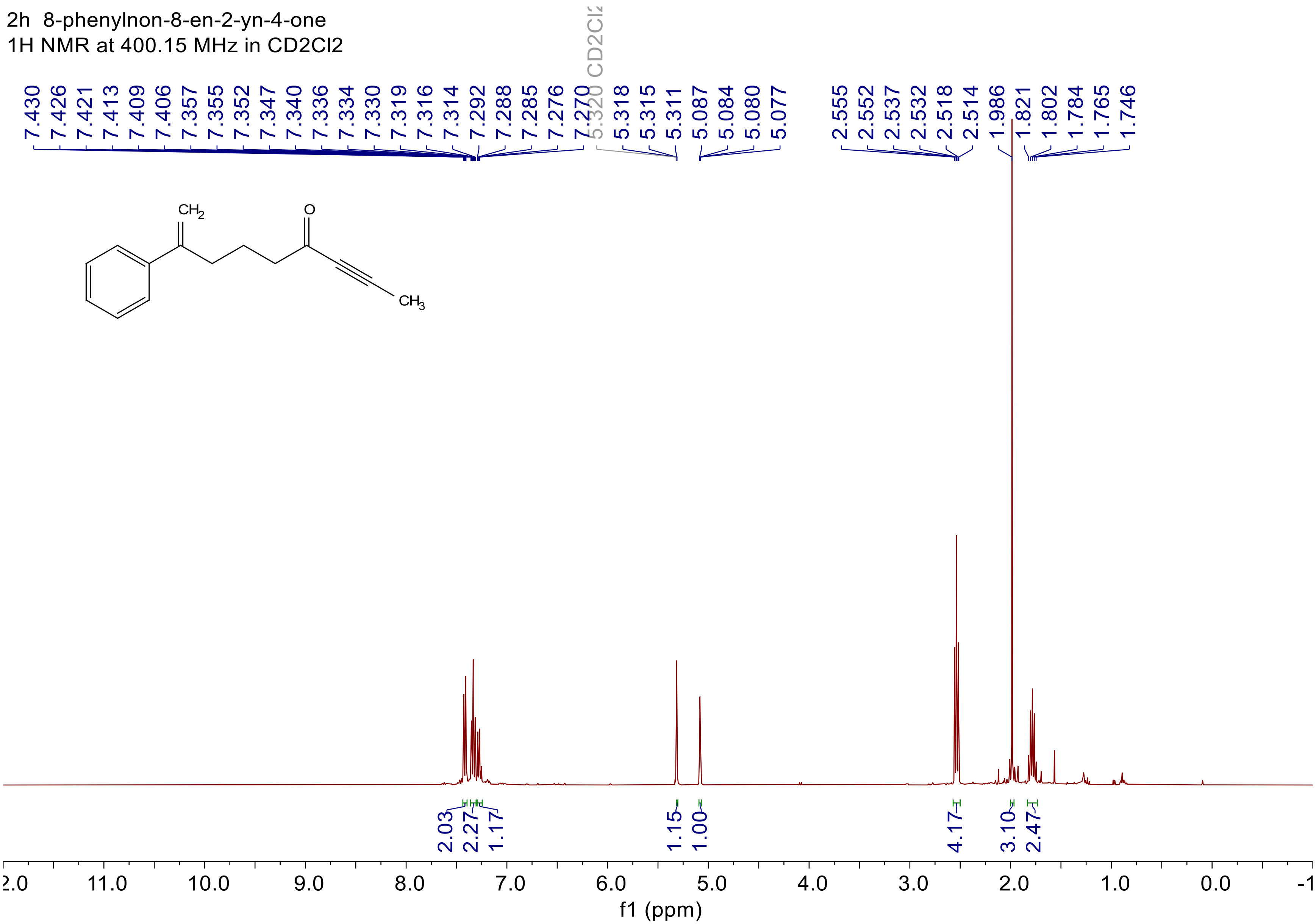
2h 8-phenylnon-8-en-2-yn-4-one
1H NMR at 400.15 MHz in CD2Cl2
7.430 7.426 7.421 7.413 7.409 7.406 7.357 7.355 7.352 7.347 7.340 7.336 7.334 7.330 7.319 7.316 7.314 7.292 7.288 7.285 7.276 7.270
5.320 CD2Cl2
5.318 5.315 5.311 5.087 5.084 5.080 5.077
2.555 2.552 2.537 2.532 2.518 2.514 1.986 1.821 1.802 1.784 1.765 1.746
CH2
O
CH3
2.03 2.27 1.17
1.15 1.00
4.17
3.10 2.47
f1 (ppm)

2h 8-phenylnon-8-en-2-yn-4-one
13C NMR at 100.63 MHz in CD2Cl2

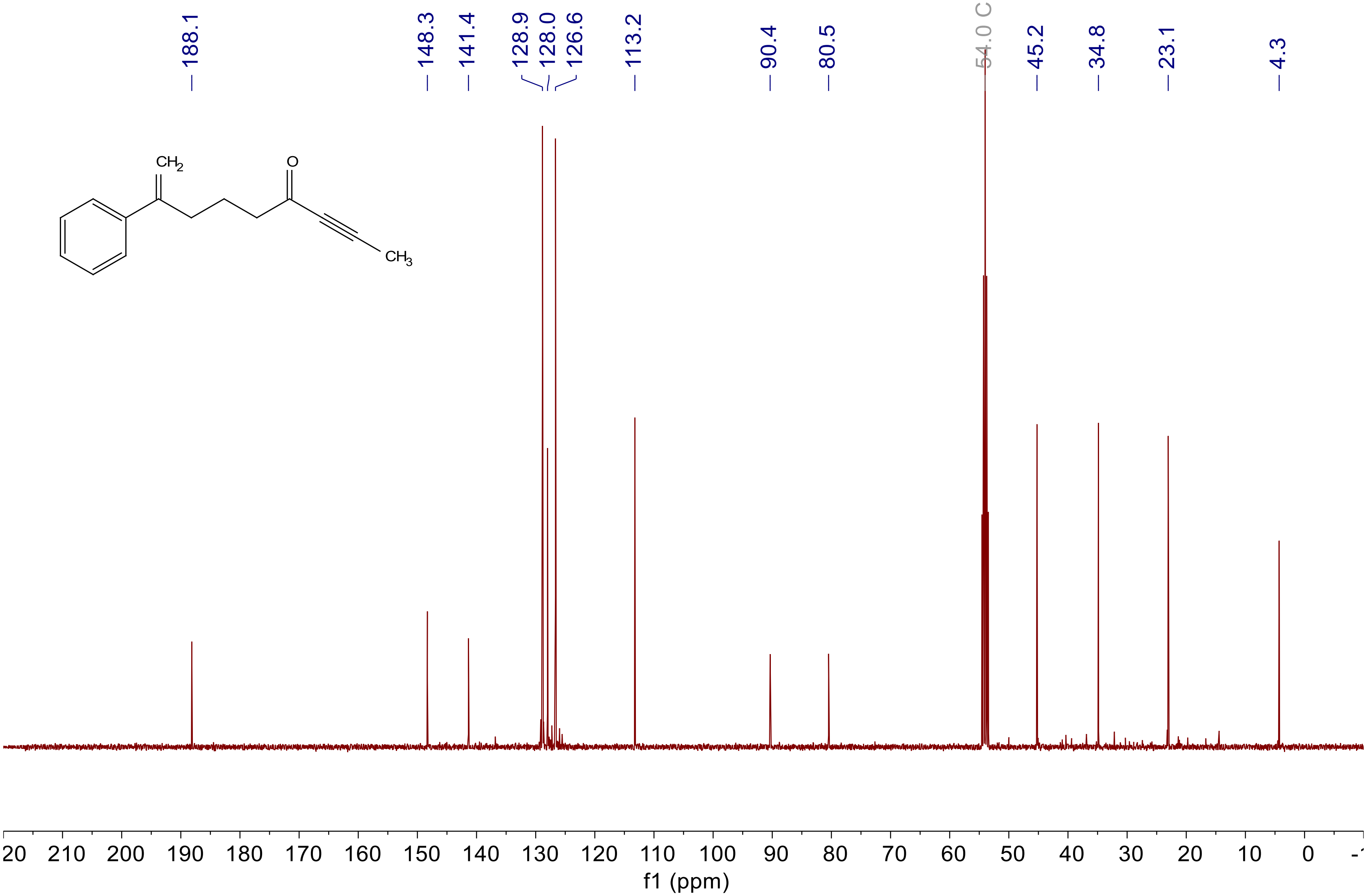

2h 8-phenylnon-8-en-2-yn-4-one

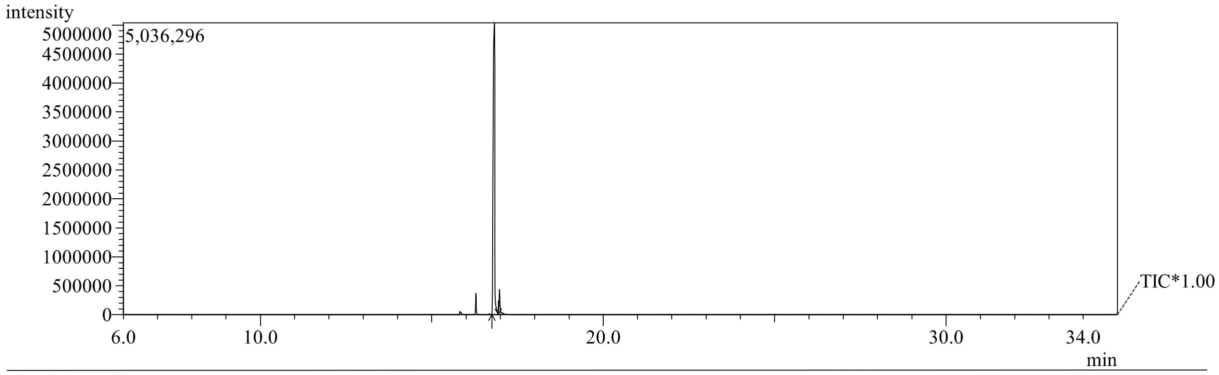

Spectrum

MassPeaks:95
RawMode:Averaged 16.8-16.8(1299-1301) BasePeak:129(598851)
BG Mode:Calc. from Peak

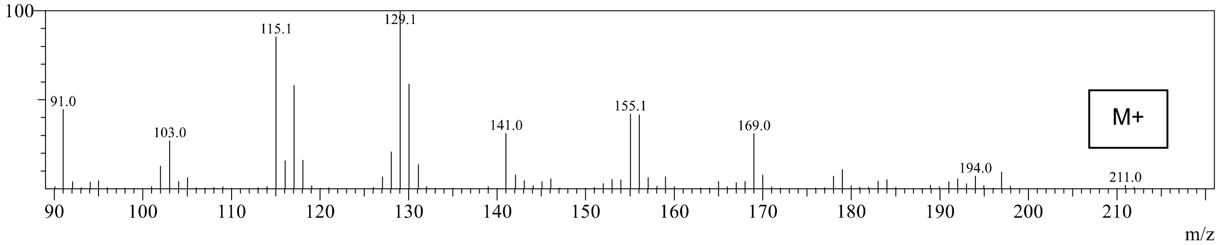

2i 7-(4-(trifluoromethyl)phenyl)oct-7-en-1-yn-3-one
1H NMR at 400.15 MHz in CD2Cl2

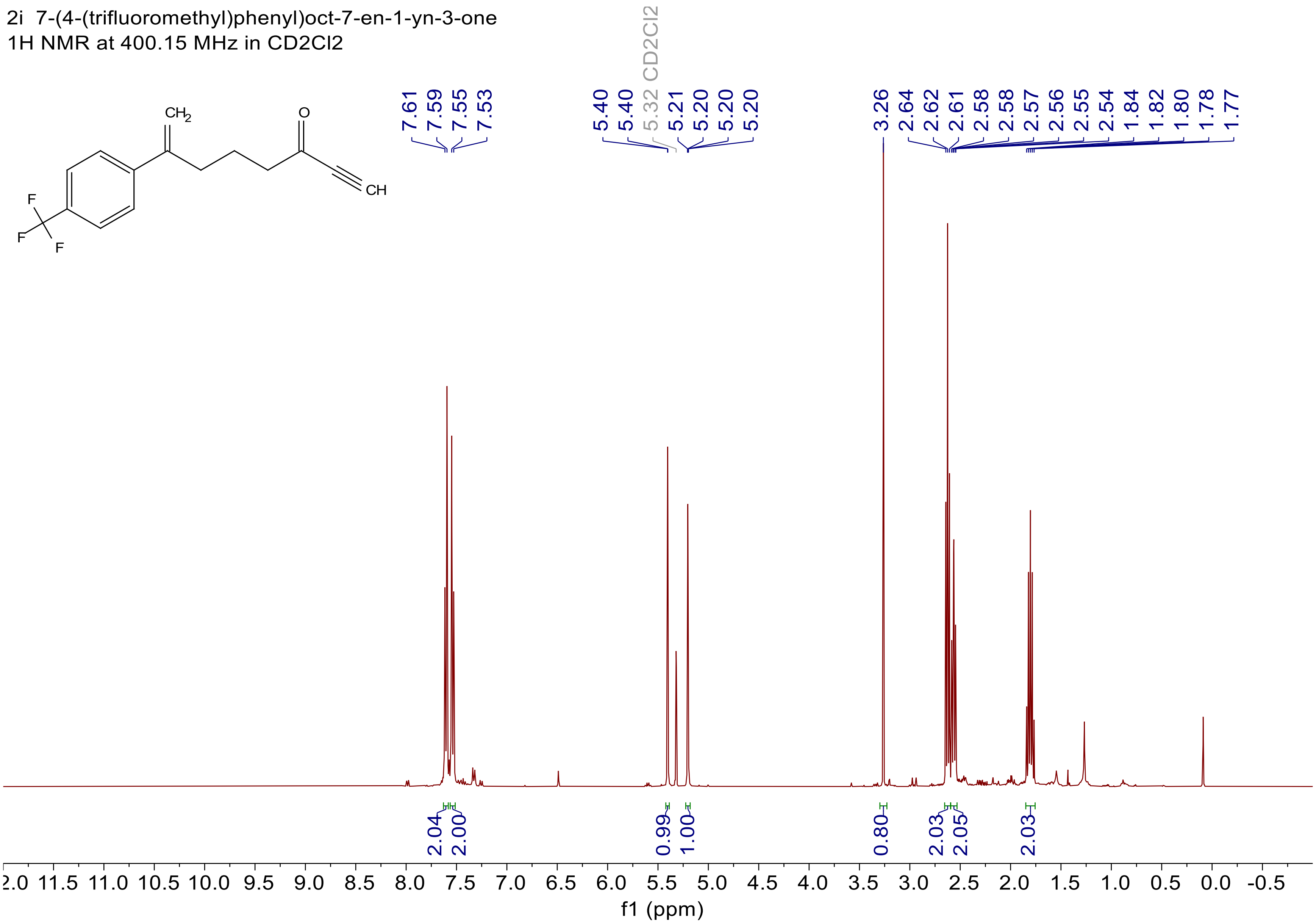

2i 7-(4-(trifluoromethyl)phenyl)oct-7-en-1-yn-3-one
19F NMR at 376.48 MHz in CD2Cl2

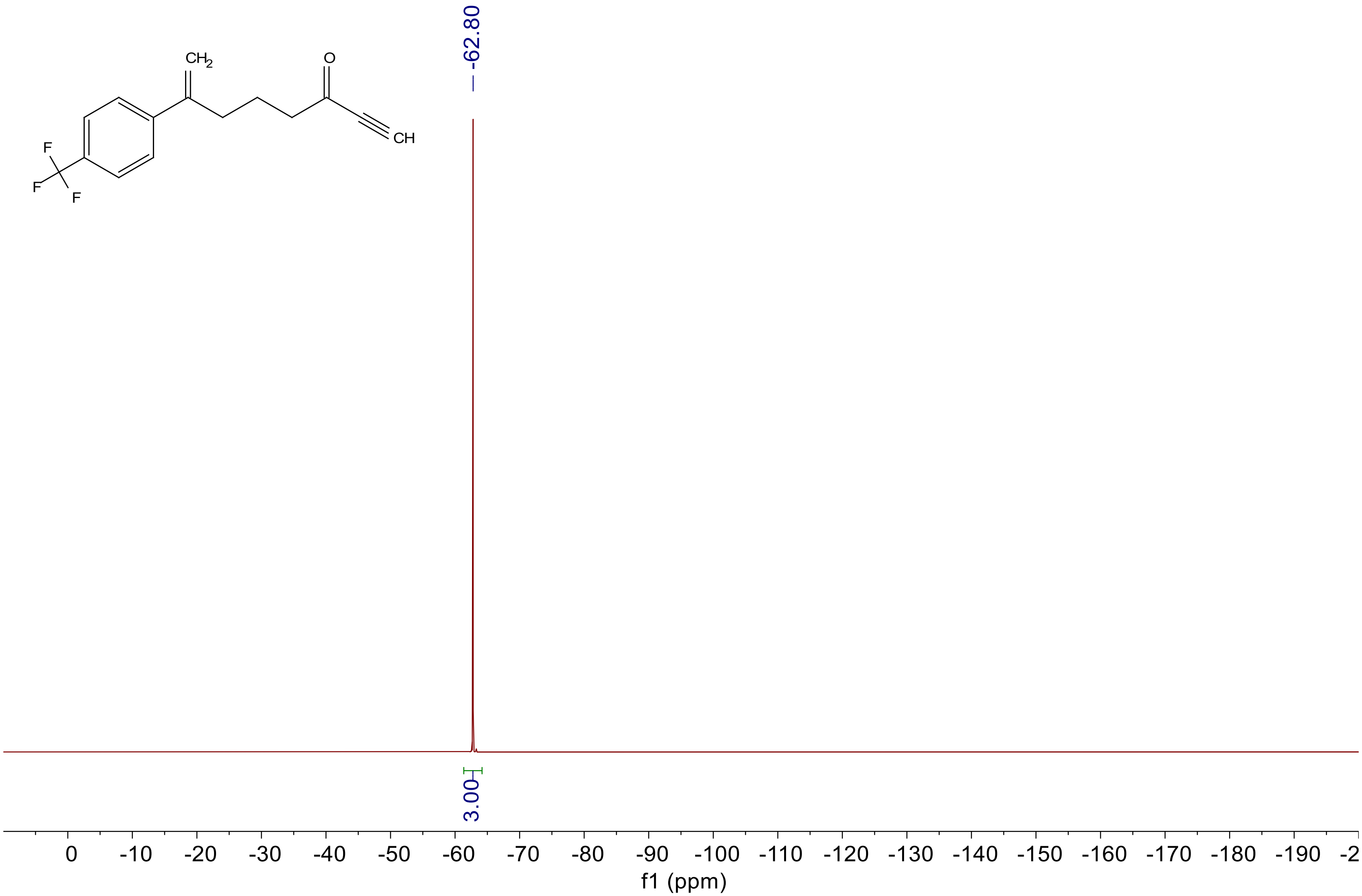

2i 7-(4-(trifluoromethyl)phenyl)oct-7-en-1-yn-3-one
13C NMR at 100.63 MHz in CD2Cl2

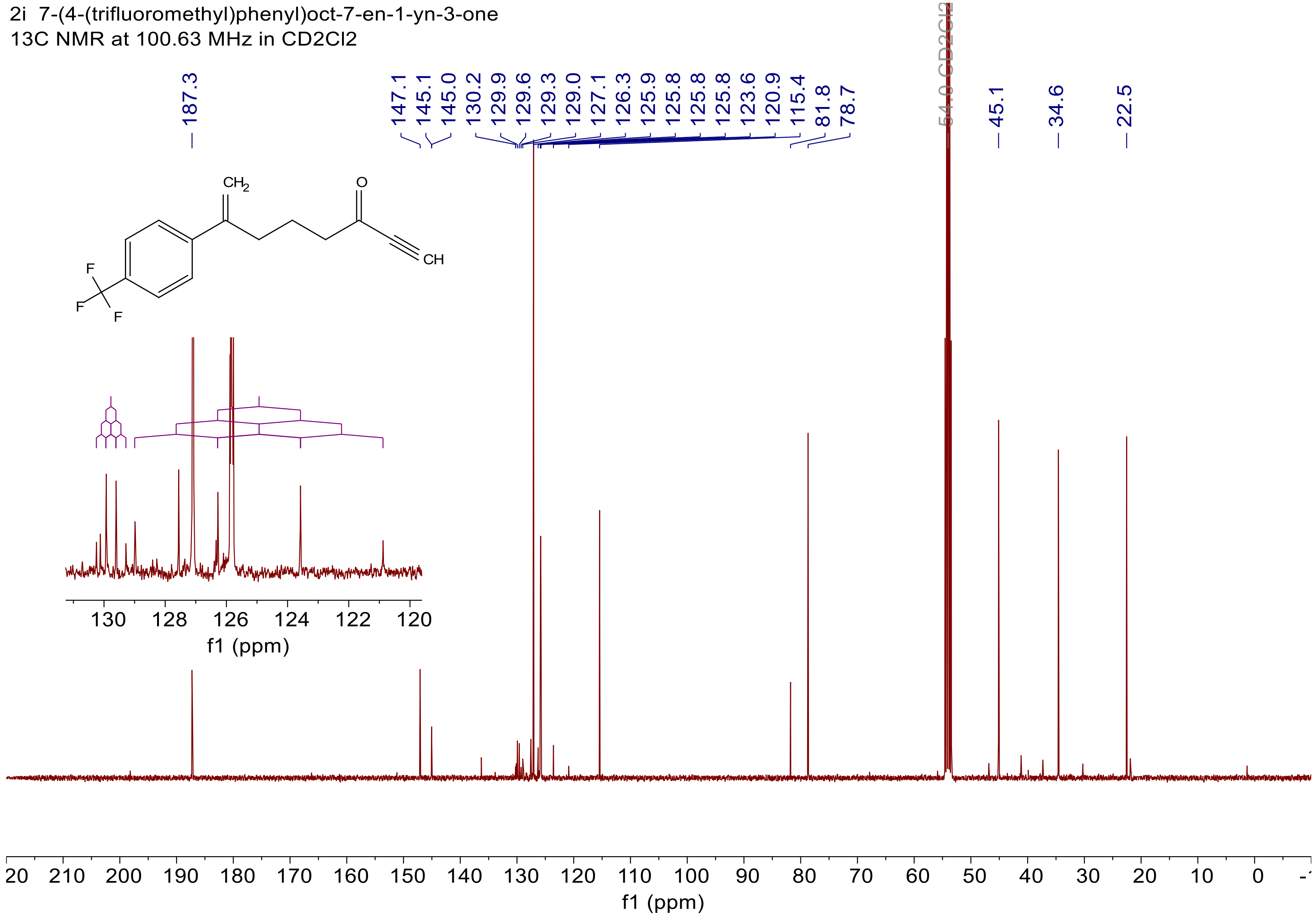

2i 7-(4-(trifluoromethyl)phenyl)oct-7-en-1-yn-3-one

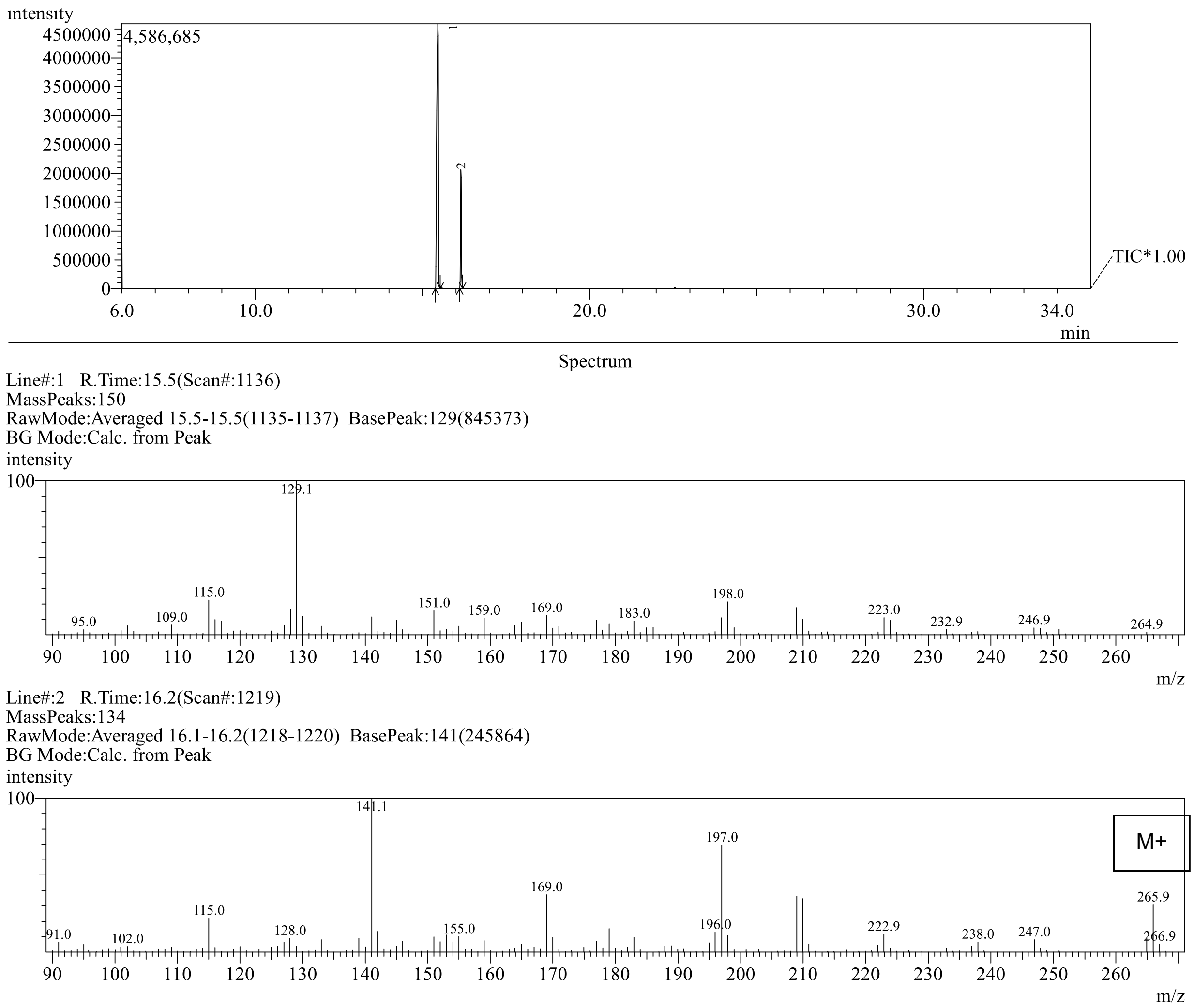

2j 5-(p-tolyl)hex-5-enal
1H NMR at 400.15 MHz in CDCl3

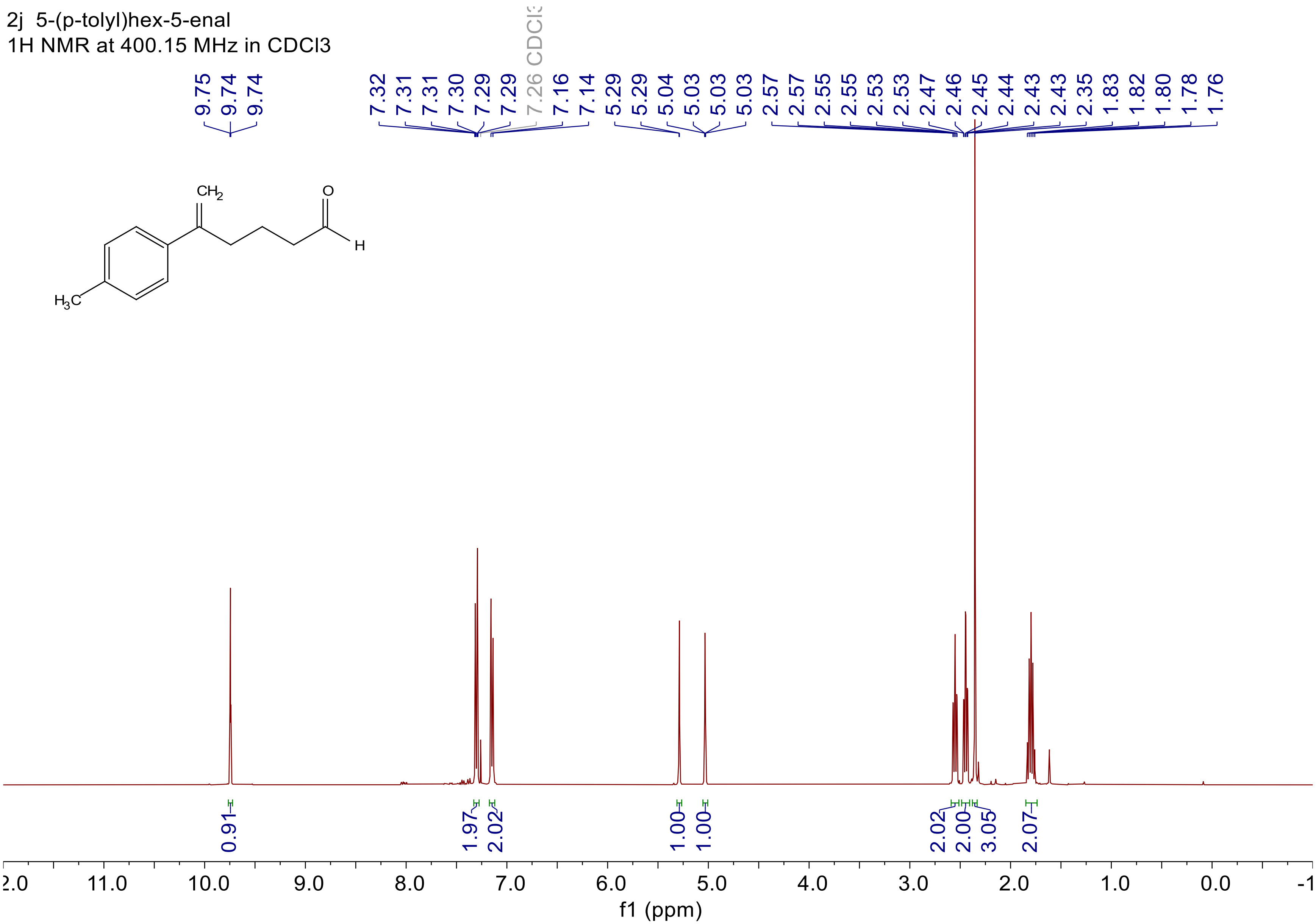

2j 5-(p-tolyl)hex-5-enal
13C NMR at 100.63 MHz in CDCl3

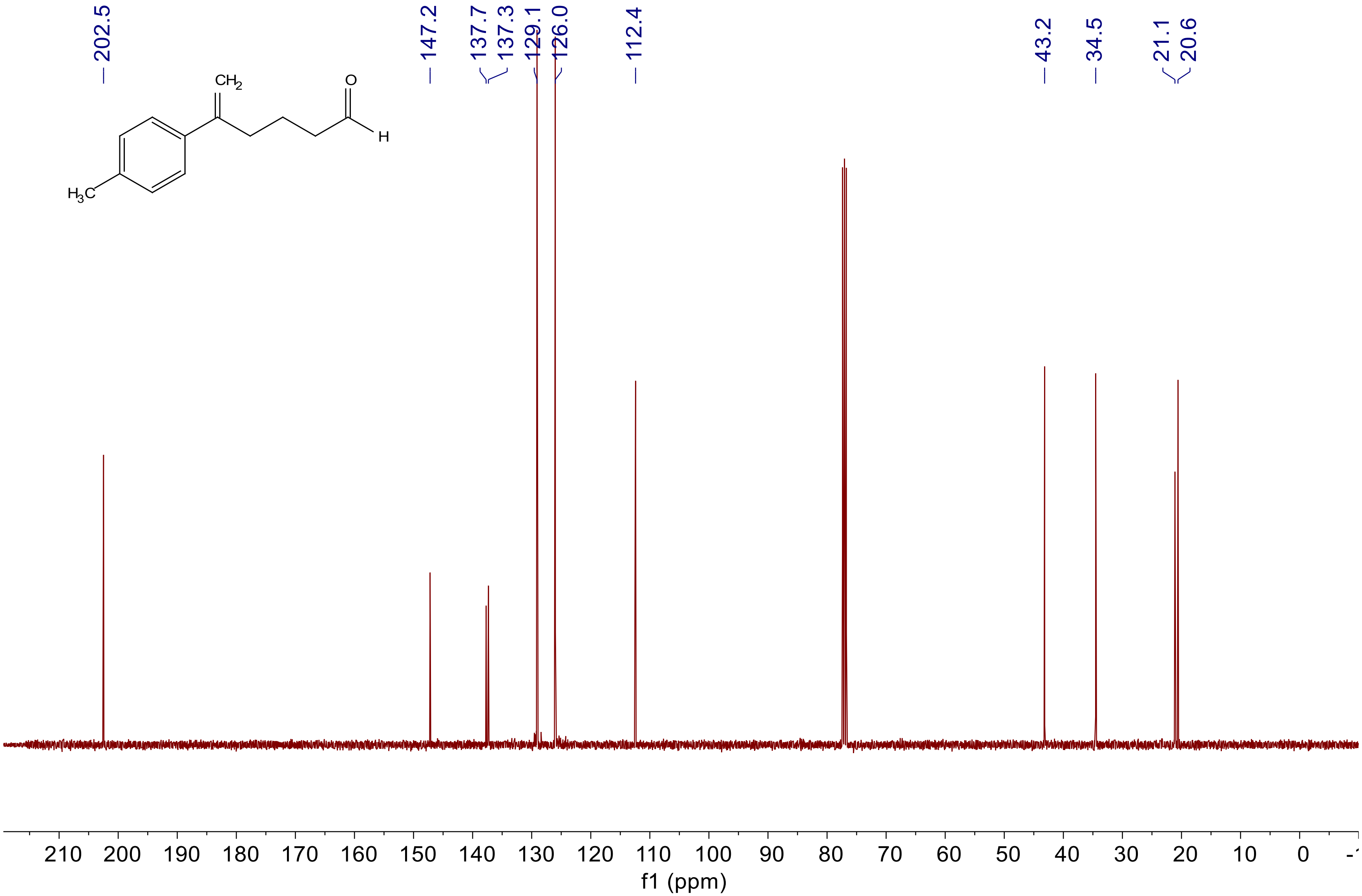

2j 5-(p-tolyl)hex-5-enal

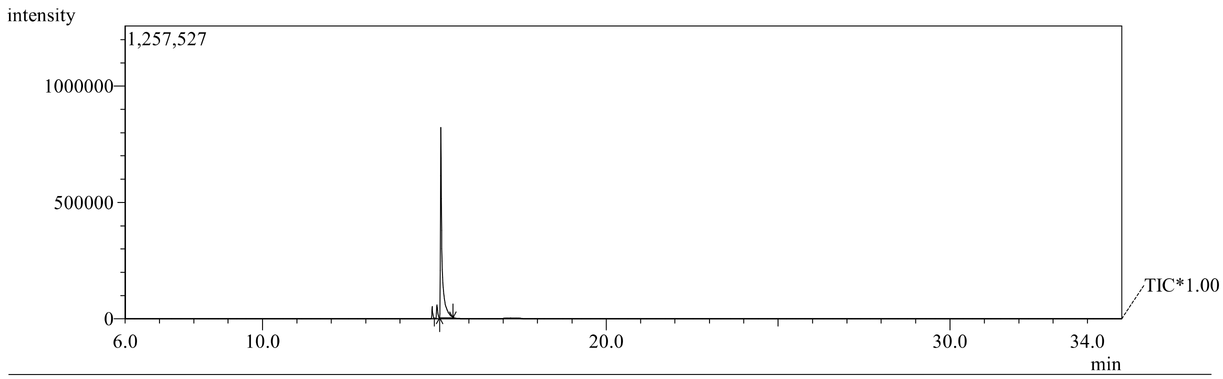

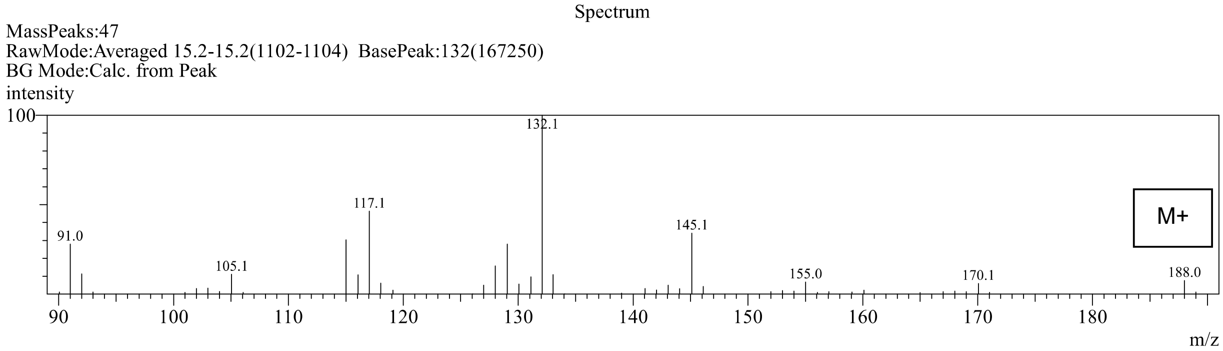

2k 5-(4-(trifluoromethyl)phenyl)hex-5-enal
1H NMR at 400.15 MHz in CD2Cl2

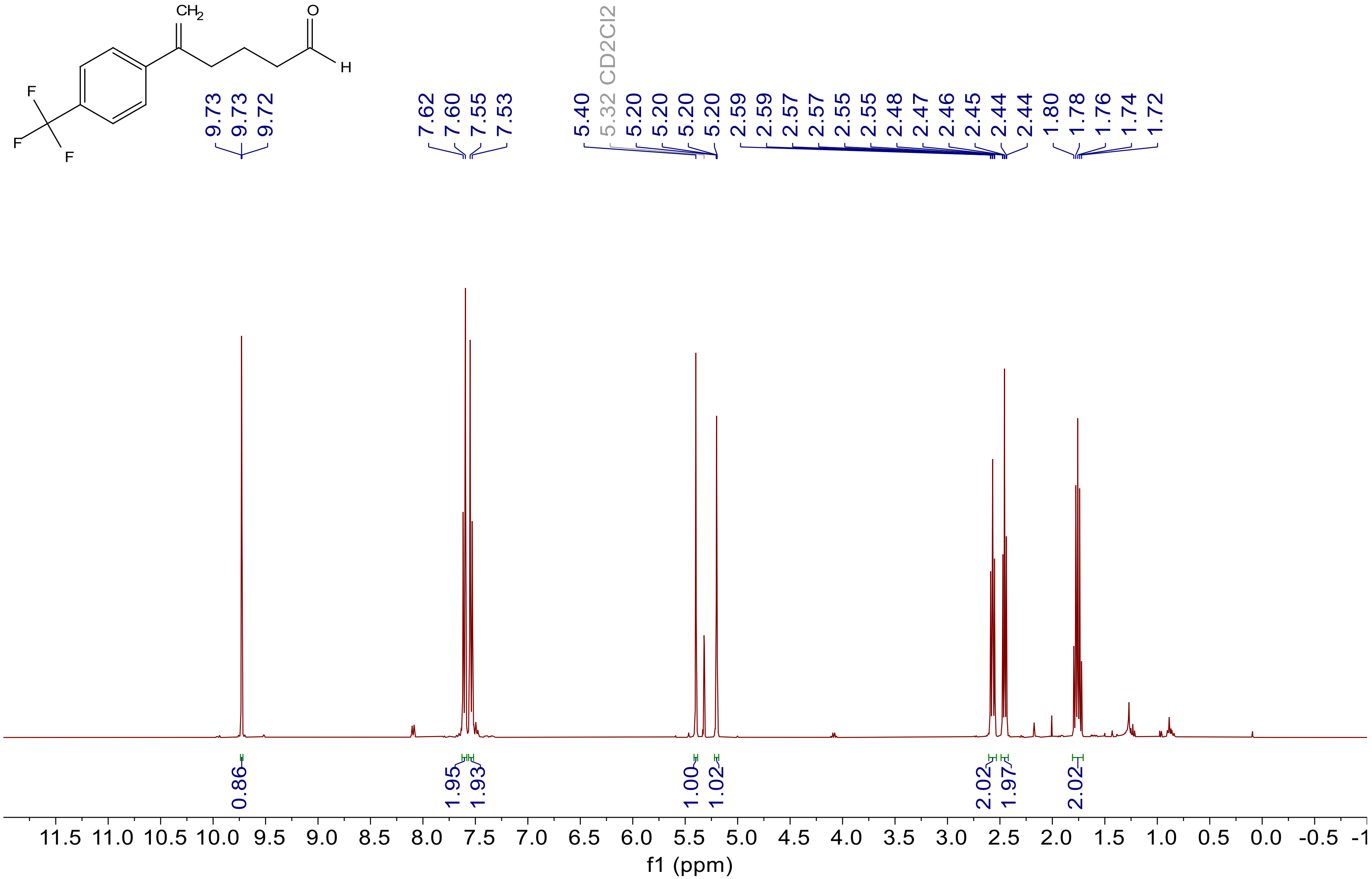

2k 5-(4-(trifluoromethyl)phenyl)hex-5-enal
19F NMR at 376.48 MHz in CD2Cl2

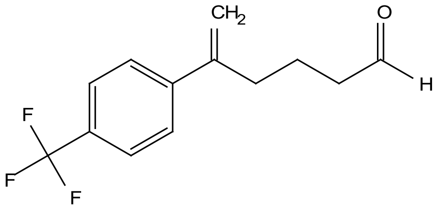

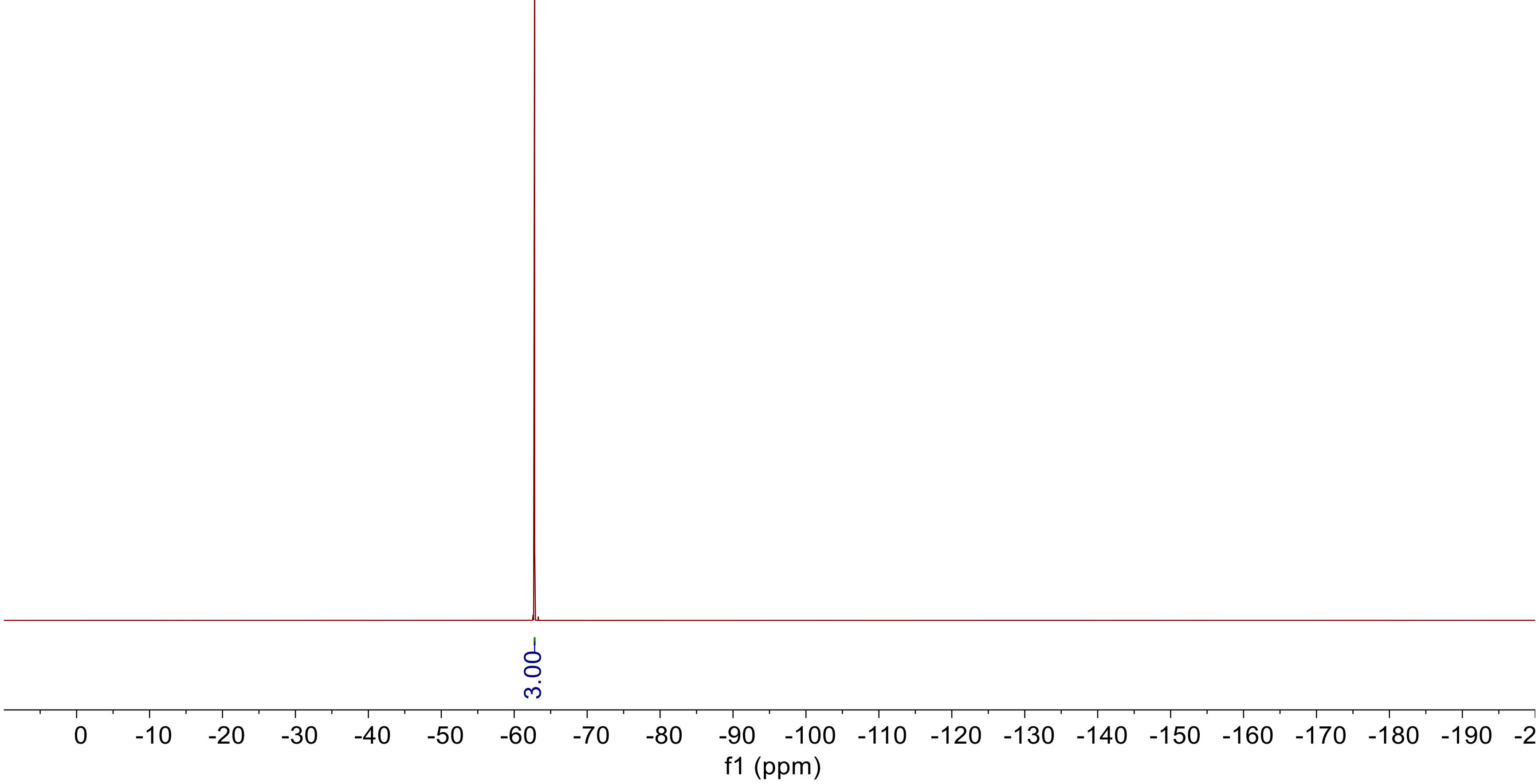

2k 5-(4-(trifluoromethyl)phenyl)hex-5-enal
13C NMR at 100.63 MHz in CD2Cl2

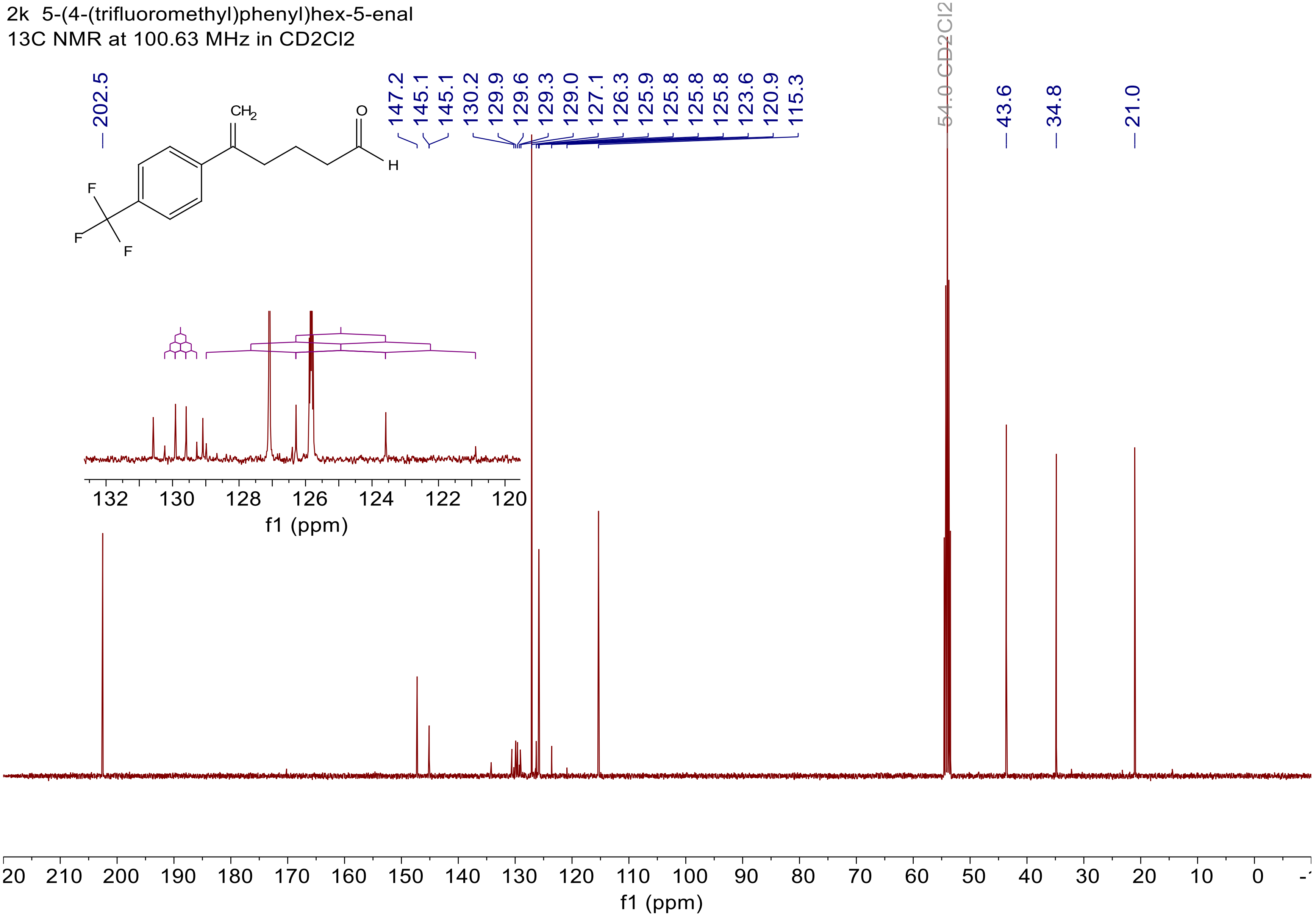

2k 5-(4-(trifluoromethyl)phenyl)hex-5-enal

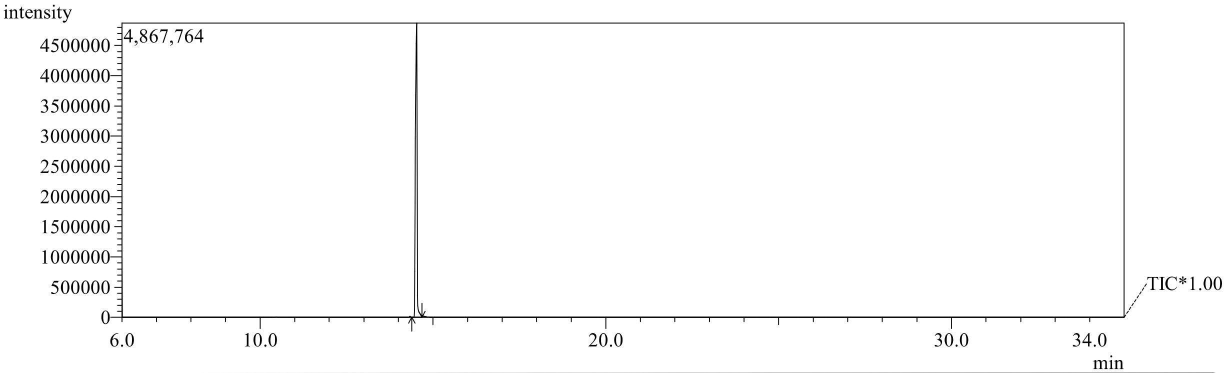

Spectrum

MassPeaks:127
RawMode:Averaged 14.5-14.5(1024-1026) BasePeak:186(547107)
BG Mode:Calc. from Peak

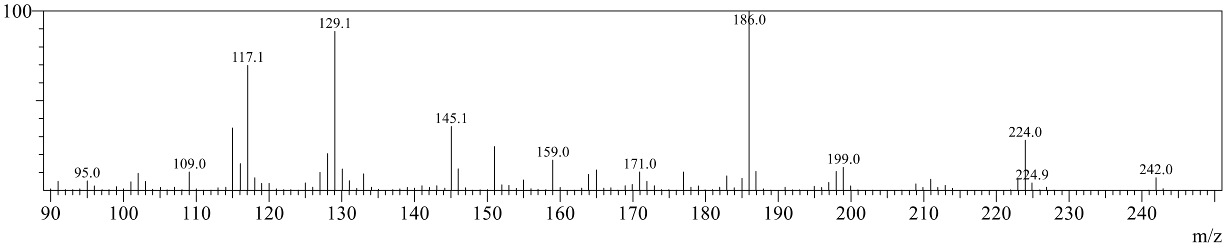

2l 5-phenylhex-5-enal
1H NMR at 400.15 MHz in CD2Cl2

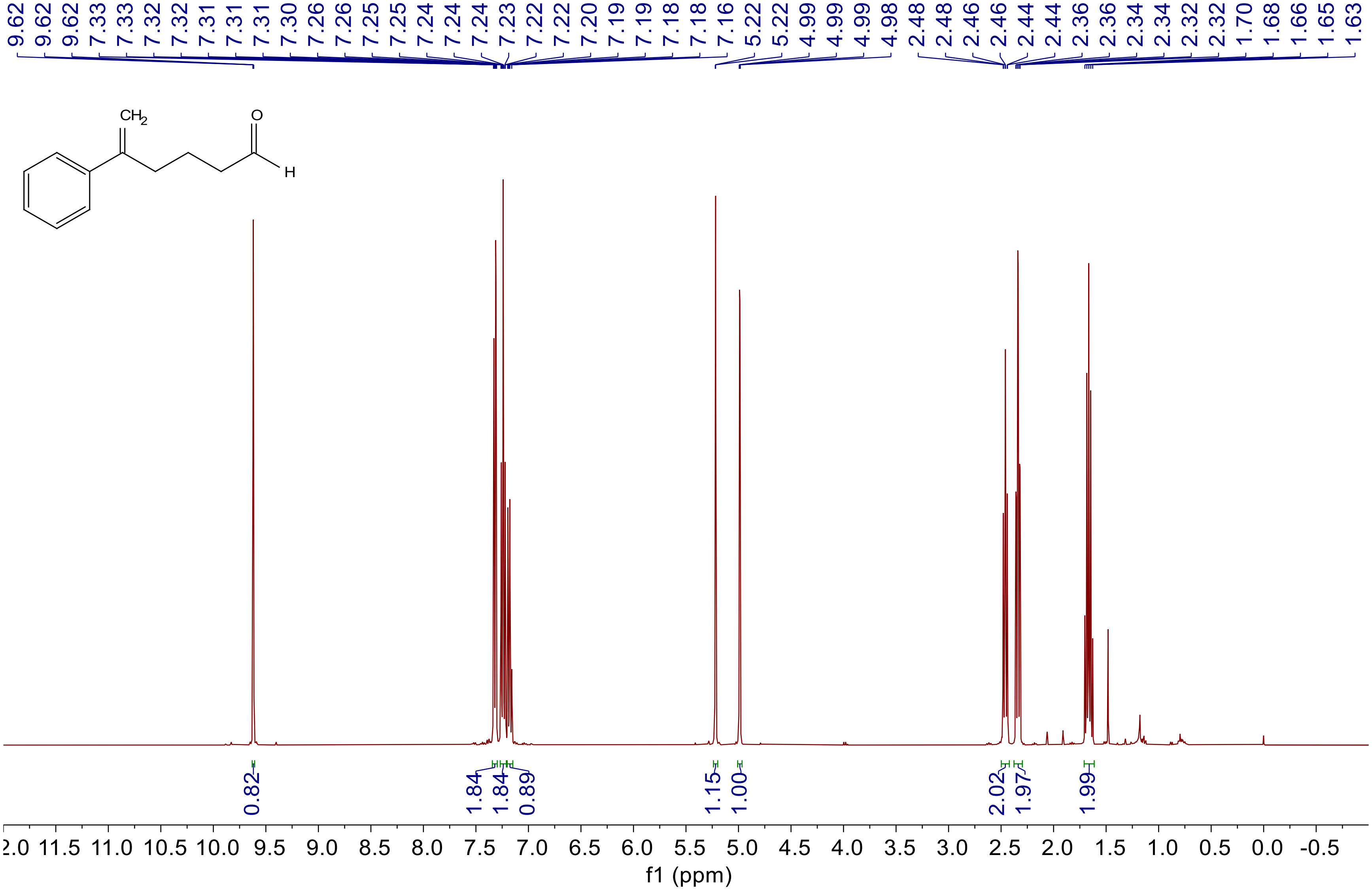

2l 5-phenylhex-5-enal
13C NMR at 100.63 MHz in CD2Cl2

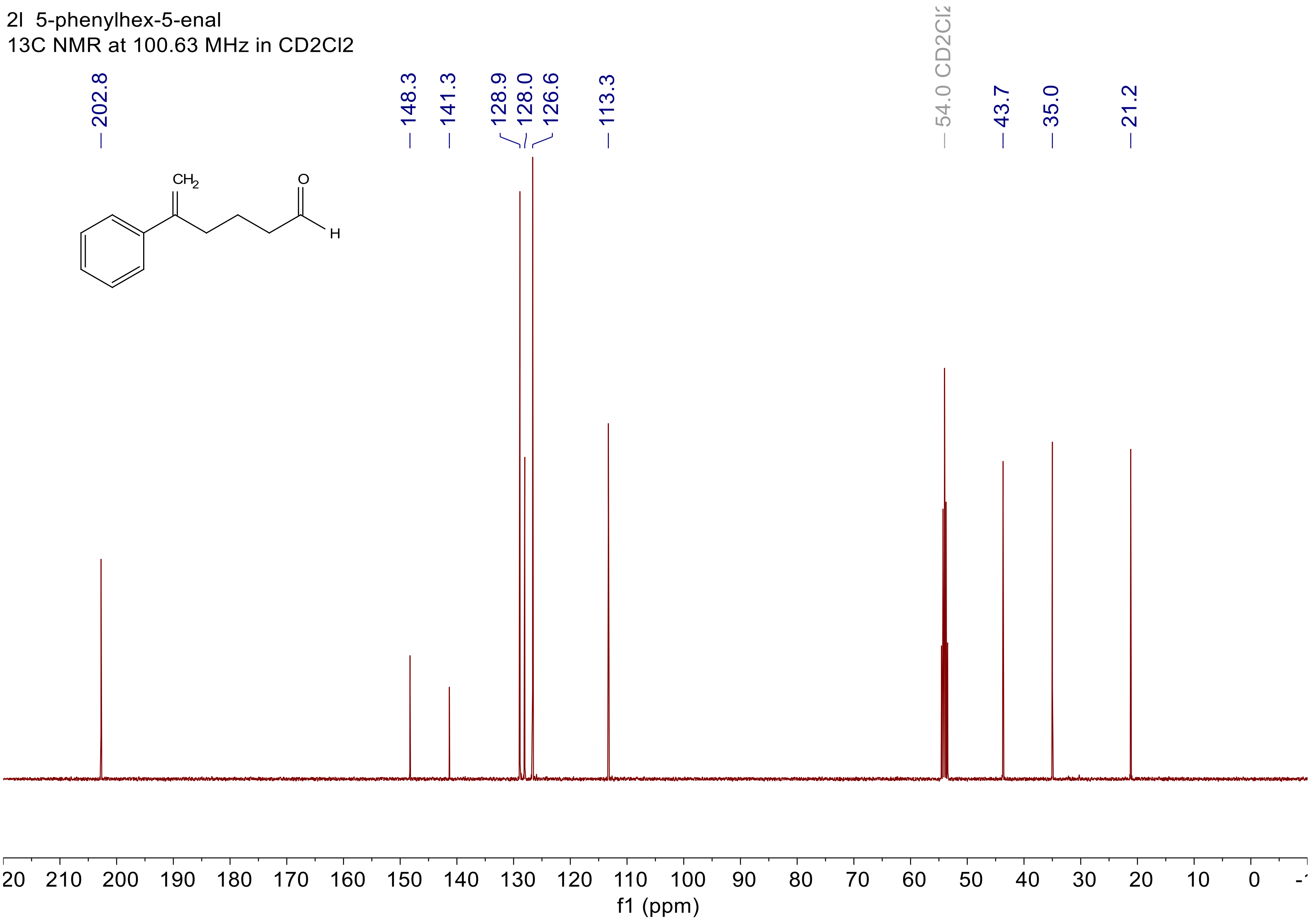

2l 5-phenylhex-5-enal

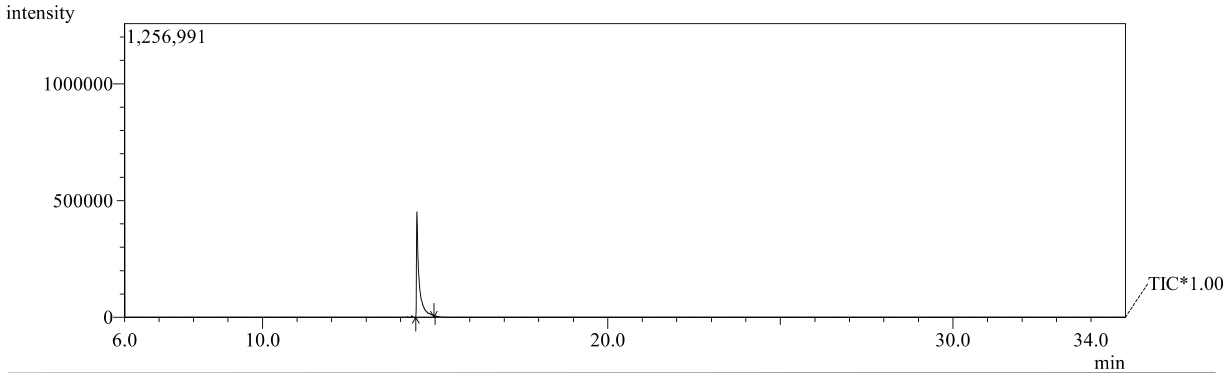

Spectrum
MassPeaks:31
RawMode:Averaged 14.5-14.5(1017-1019) BasePeak:118(104711)
BG Mode:Calc. from Peak

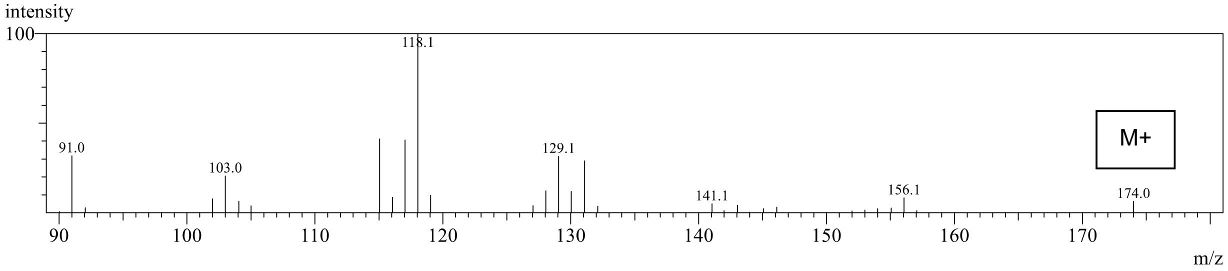

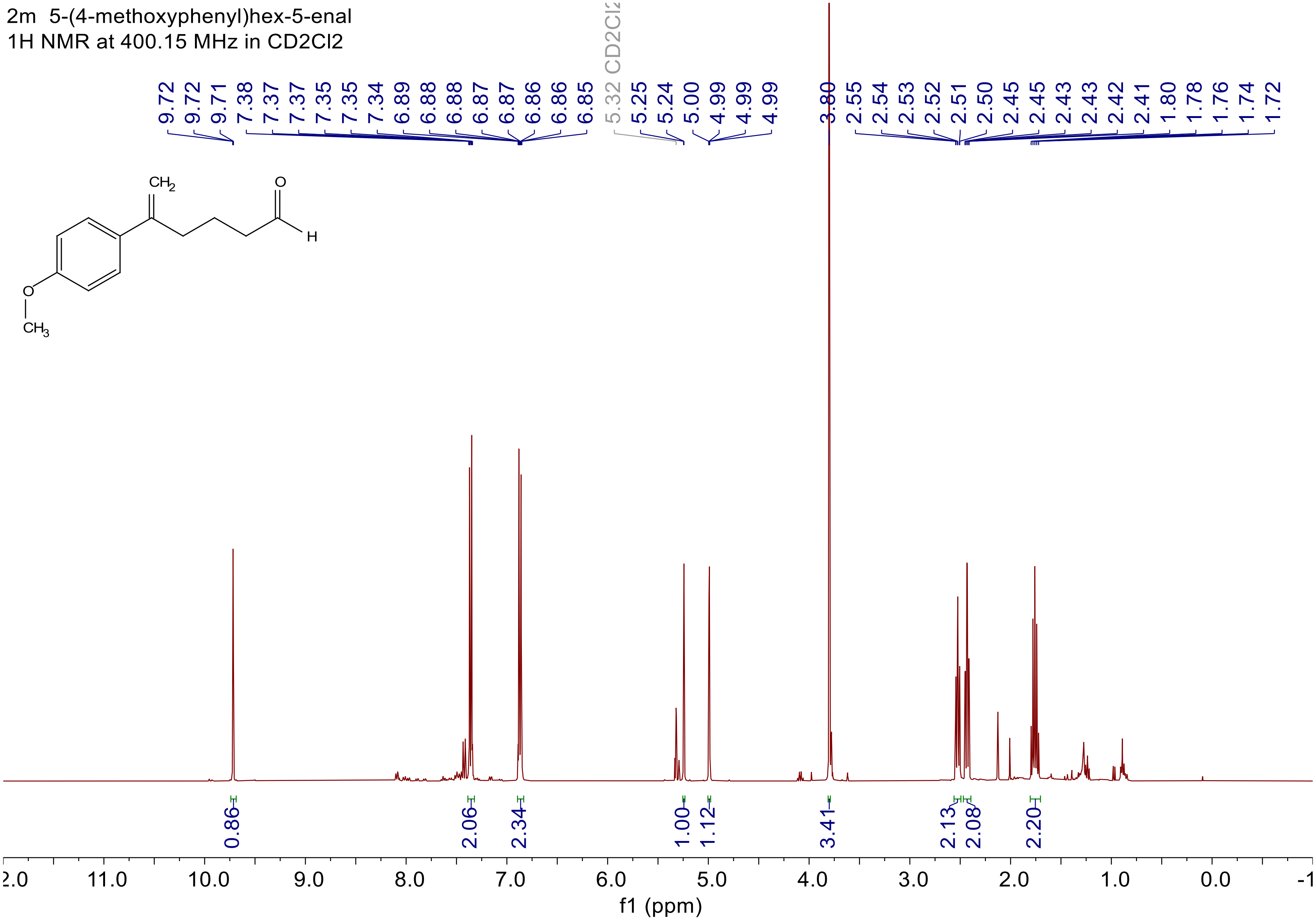
2m 5-(4-methoxyphenyl)hex-5-enal
1H NMR at 400.15 MHz in CD2Cl2
CD2Cl2
9.72 9.72 9.71 7.38 7.37 7.37 7.35 7.35 7.34 6.89 6.88 6.88 6.87 6.87 6.86 6.86 6.85 5.32 5.25 5.24 5.00 4.99 4.99 4.99 3.80 2.55 2.54 2.53 2.52 2.51 2.50 2.45 2.45 2.43 2.43 2.42 2.41 1.80 1.78 1.76 1.74 1.72
CH2
O
H
O
CH3
0.86 2.06 2.34 1.00 1.12 3.41 2.13 2.08 2.20
11.0 10.0 9.0 8.0 7.0 6.0 5.0 4.0 3.0 2.0 1.0 0.0
f1 (ppm)

2m 5-(4-methoxyphenyl)hex-5-enal
13C NMR at 100.63 MHz in CD2Cl2

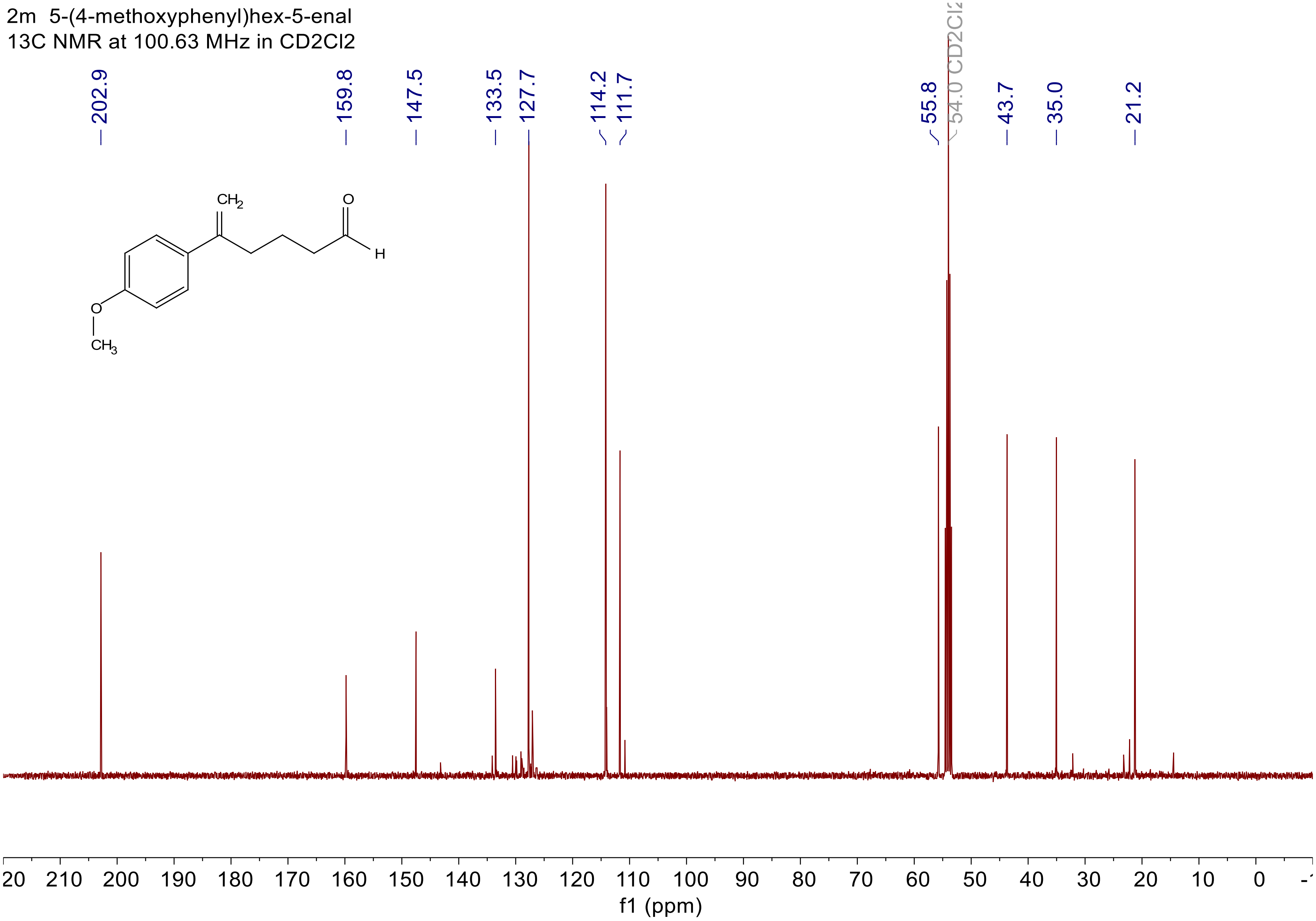

2n 5-(furan-2-yl)hex-5-enal
1H NMR at 598.93 MHz in CD3CN

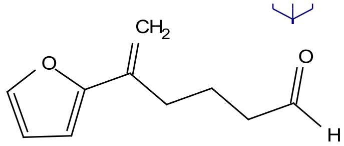

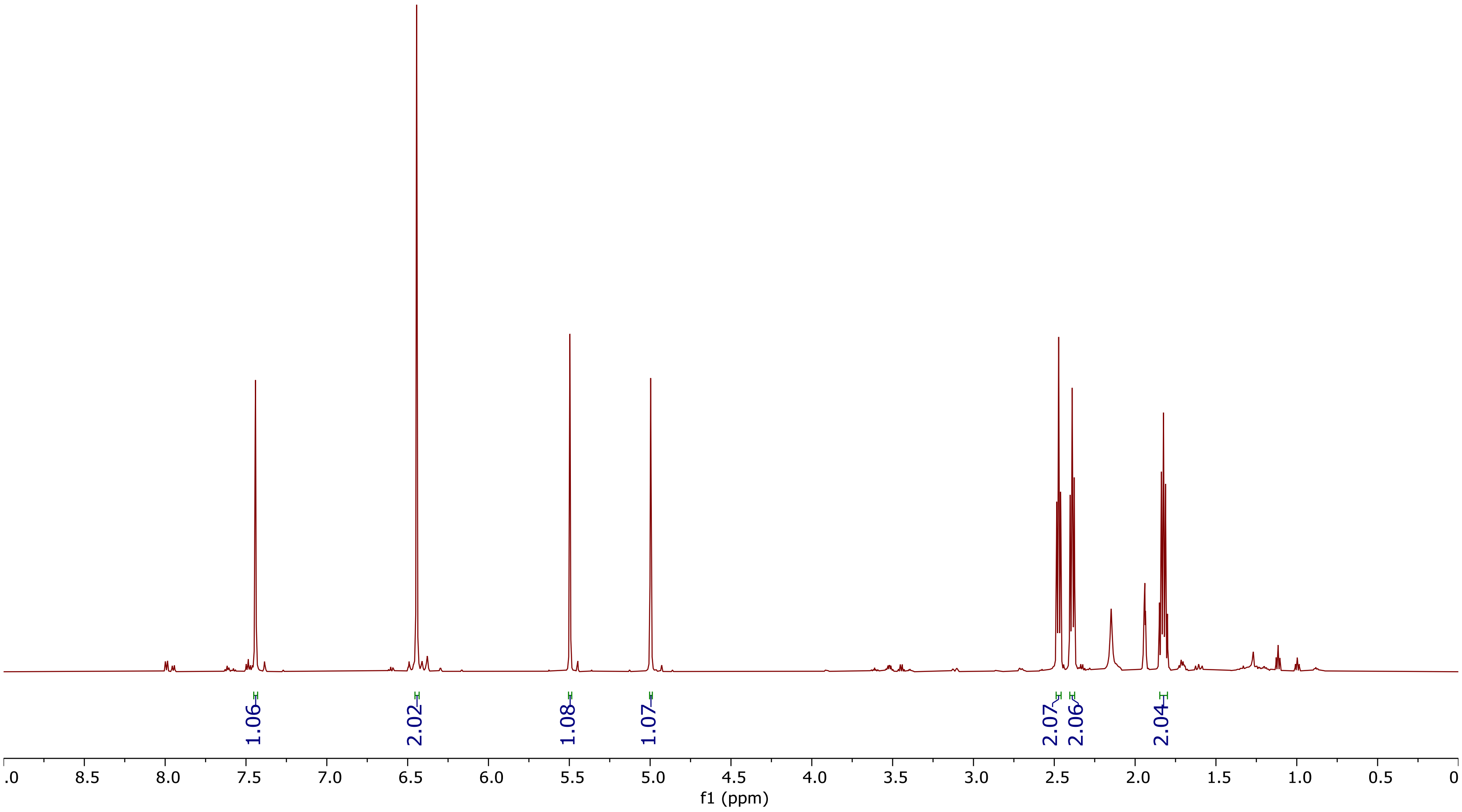

2n 5-(furan-2-yl)hex-5-enal
13C NMR at 150.62 MHz in CD3CN

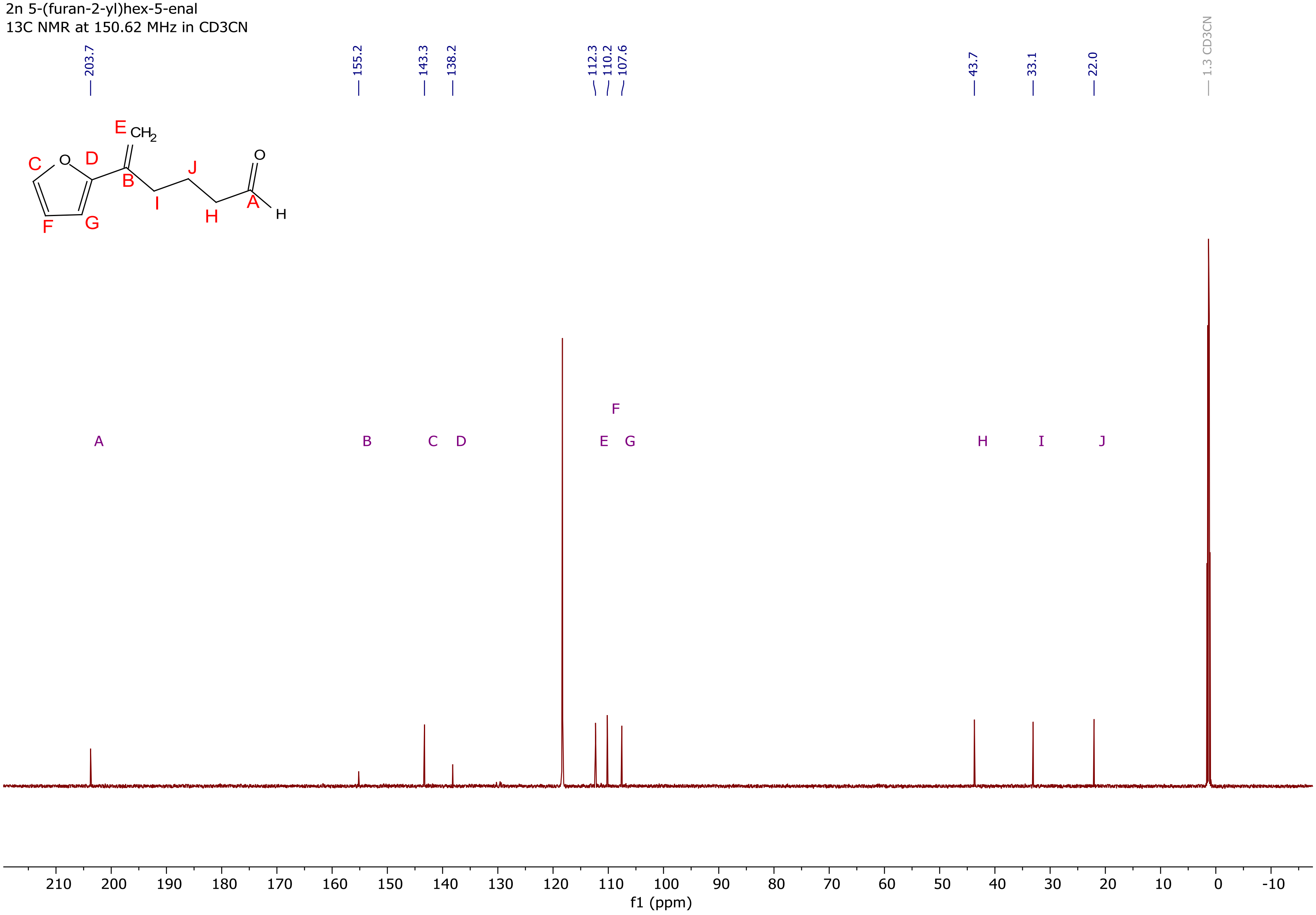

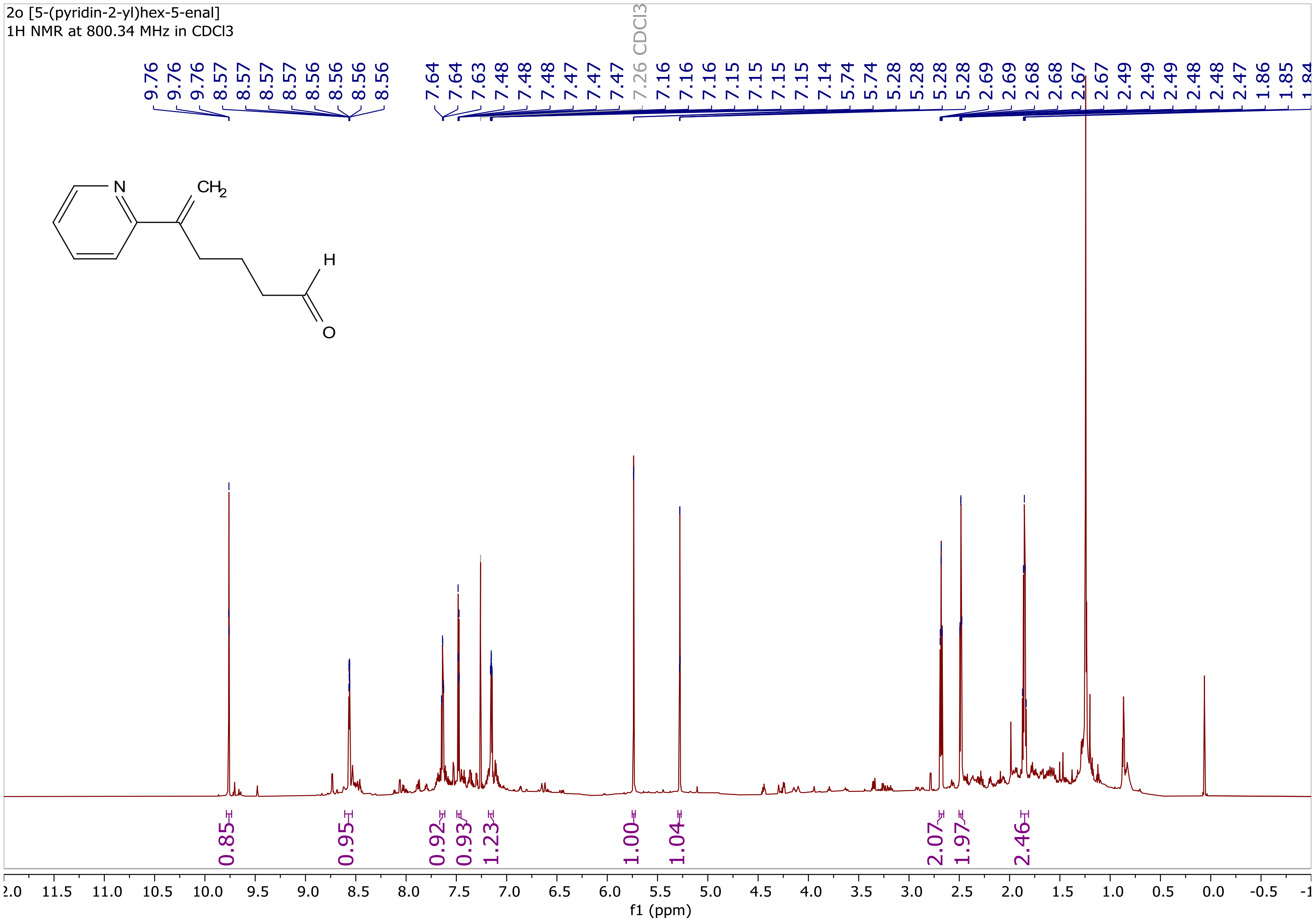
2o [5-(pyridin-2-yl)hex-5-enal]
1H NMR at 800.34 MHz in CDCl3
N
CH2
H
O
9.76 9.76 9.76 8.57 8.57 8.57 8.57 8.56 8.56 8.56 8.56
7.64 7.64 7.63 7.48 7.48 7.48 7.47 7.47 7.47 7.26 CDCl3 7.16 7.16 7.16 7.15 7.15 7.15 7.15 7.14 5.74 5.74 5.28 5.28 5.28 5.28 2.69 2.69 2.68 2.68 2.67 2.67 2.49 2.49 2.49 2.48 2.48 2.47 1.86 1.85
0.85 0.95 0.92 0.93 1.23 1.00 1.04 2.07 1.97 2.46
f1 (ppm)

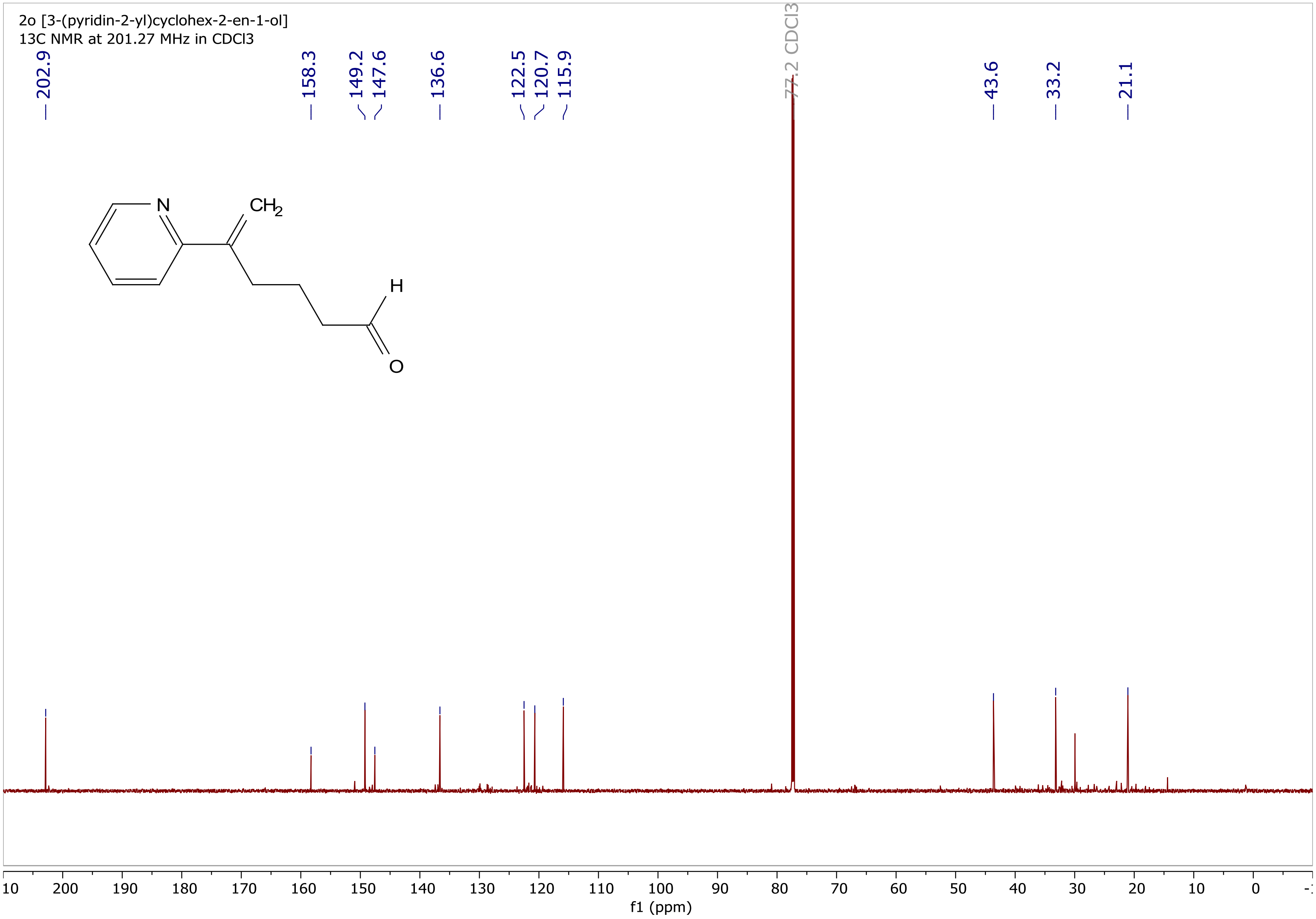
2o [3-(pyridin-2-yl)cyclohex-2-en-1-ol]
13C NMR at 201.27 MHz in CDCl3
202.9
158.3
149.2
147.6
136.6
122.5
120.7
115.9
77.2 CDCl3
43.6
33.2
21.1
N
CH2
H
O
10
200
190
180
170
160
150
140
130
120
110
100
90
80
70
60
50
40
30
20
10
0
f1 (ppm)

3 3-phenylcyclohept-2-en-1-ol
1H NMR at 400.15 MHz in CD2Cl2

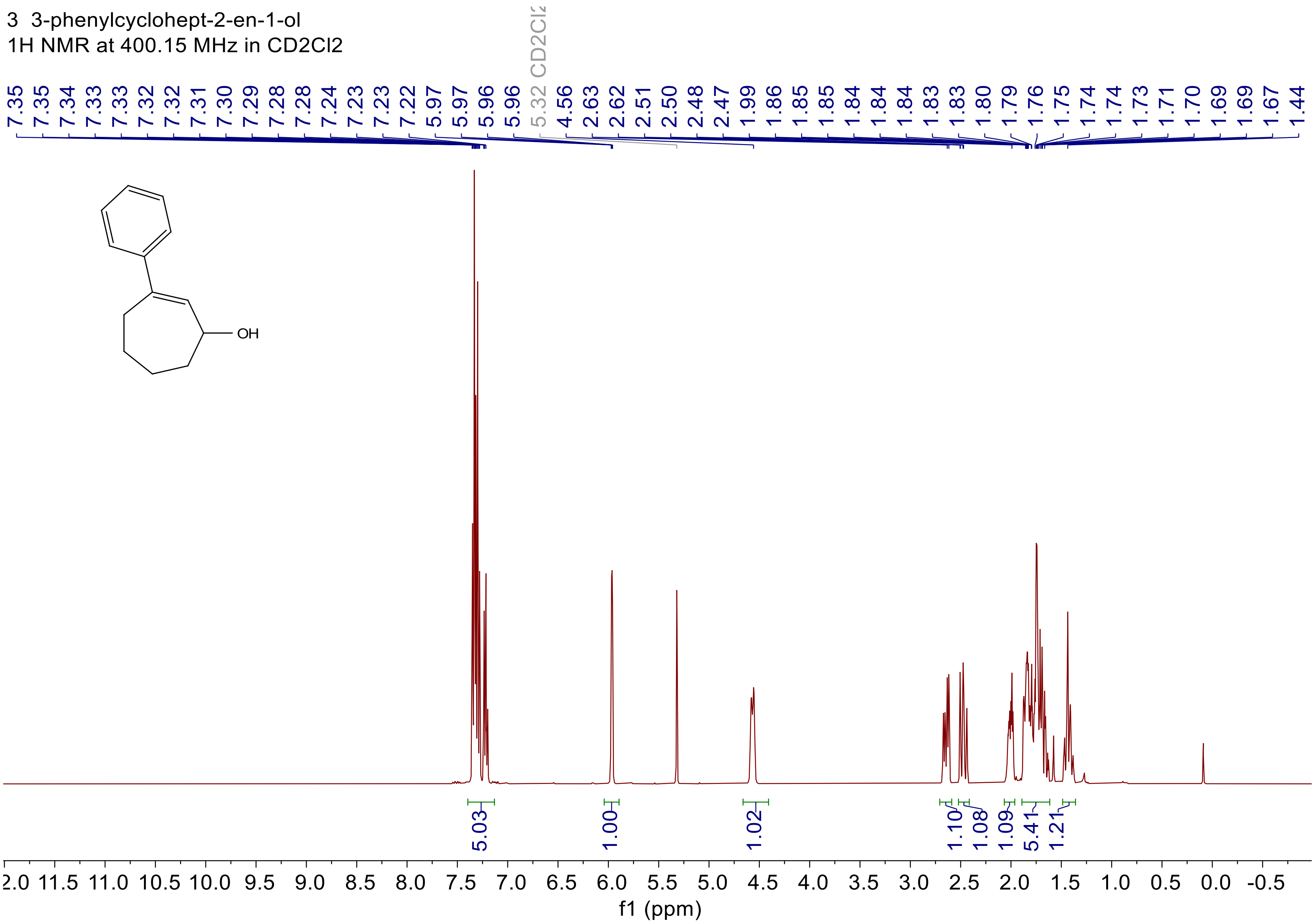

3 3-phenylcyclohept-2-en-1-ol
13C NMR at 100.63 MHz in CD2Cl2

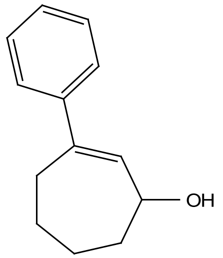

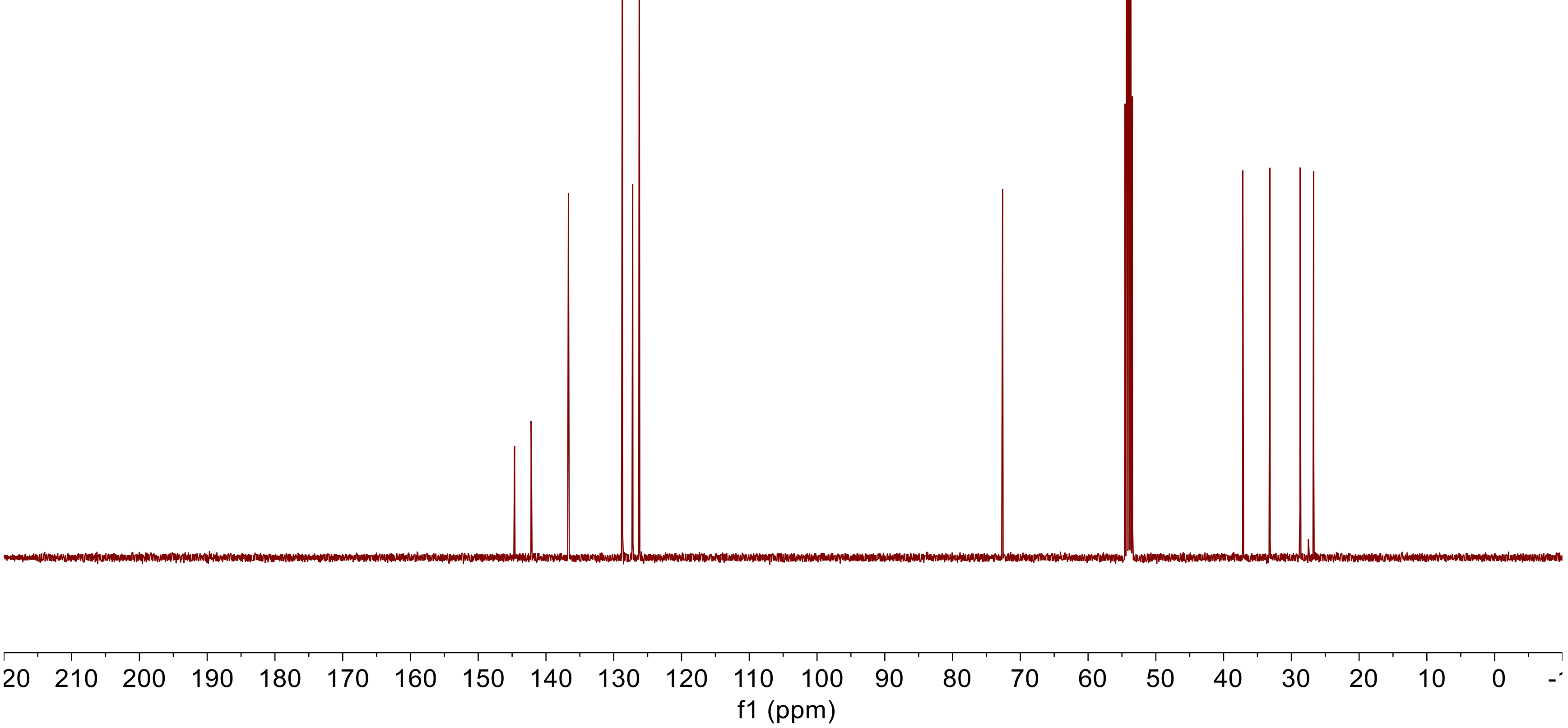

3 3-phenylcyclohept-2-en-1-ol

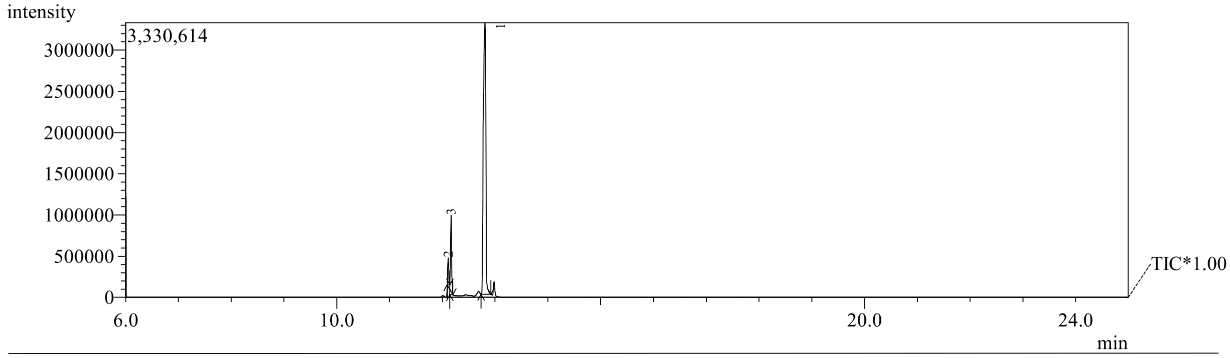

Spectrum

Line#:1 R.Time:12.8(Scan#:818)
MassPeaks:65
RawMode:Averaged 12.8-12.8(817-819) BasePeak:131(257264)
BG Mode:Calc. from Peak

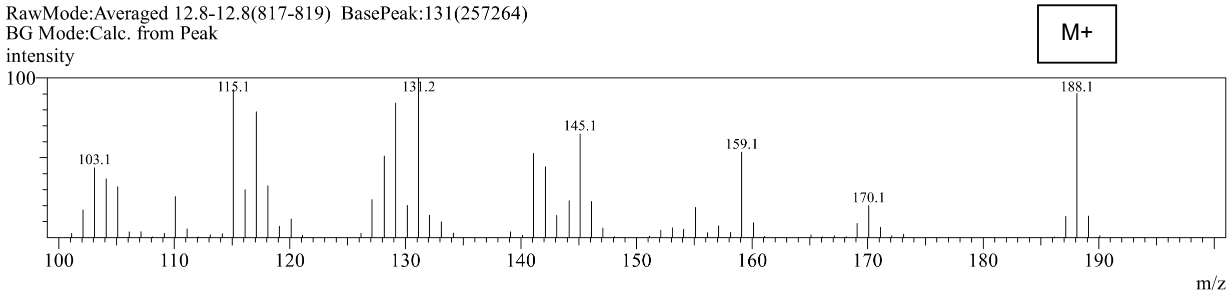

Line#:2 R.Time:12.1(Scan#:734)
MassPeaks:33
RawMode:Averaged 12.1-12.1(733-735) BasePeak:142(34518)
BG Mode:Calc. from Peak

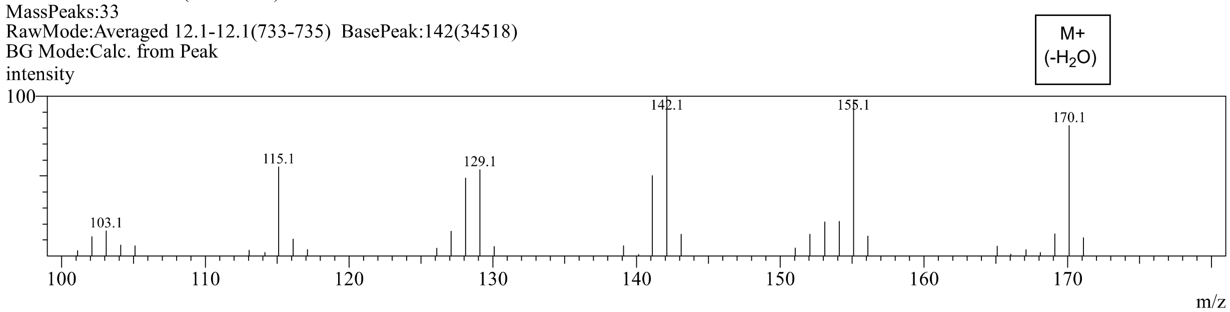

Line#:3 R.Time:12.2(Scan#:741)
MassPeaks:38
RawMode:Averaged 12.2-12.2(740-742) BasePeak:142(97041)
BG Mode:Calc. from Peak

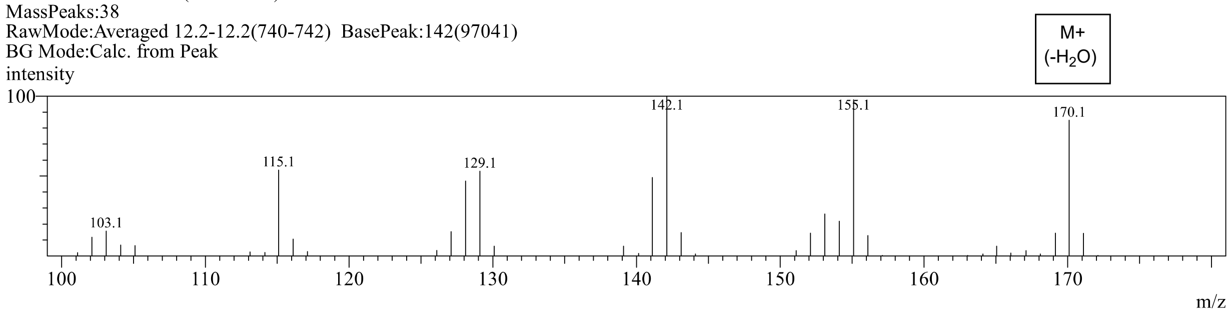

4 6-phenylhept-6-enal
1H NMR at 400.15 MHz in CDCl3

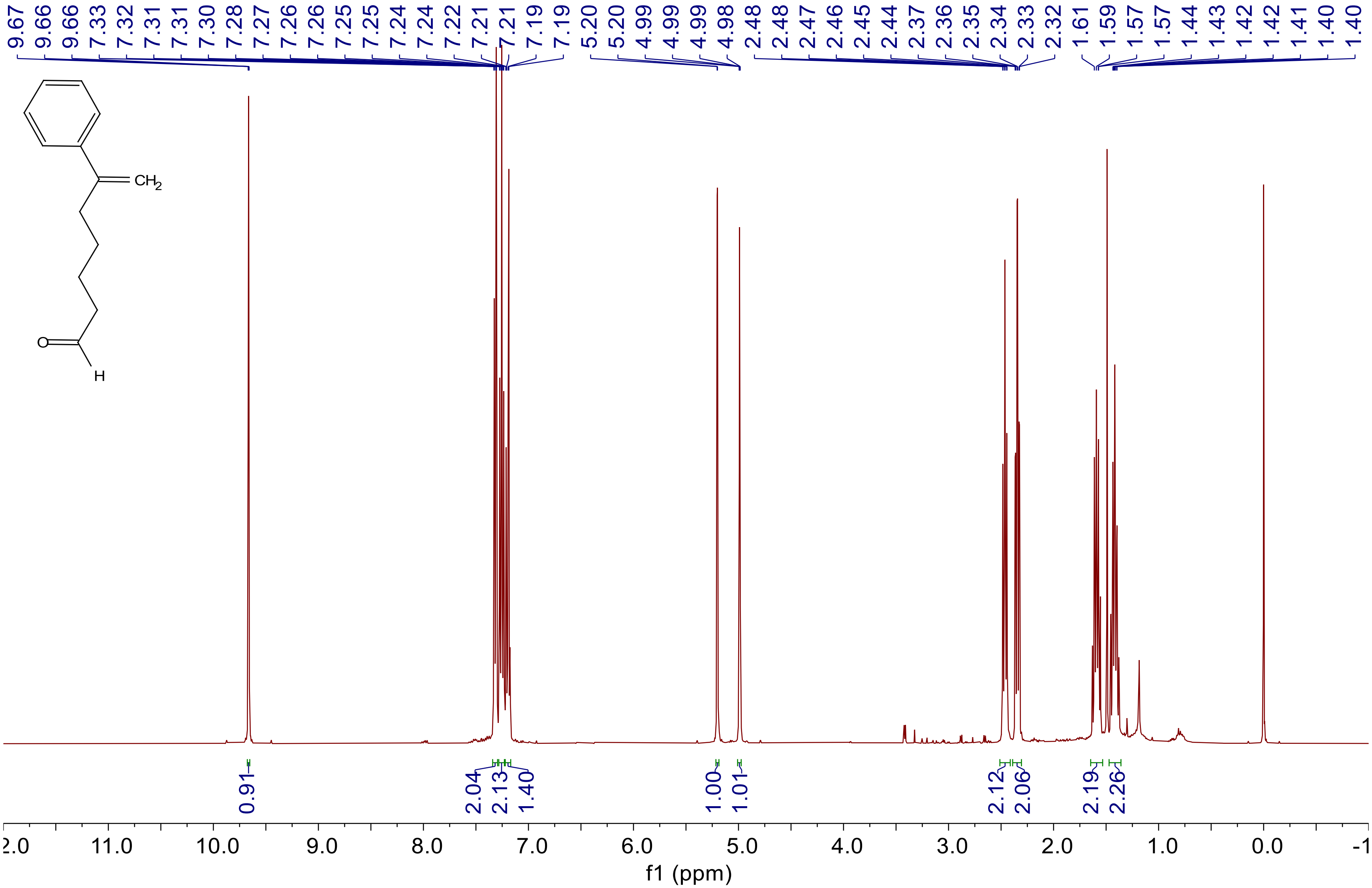

4 6-phenylhept-6-enal
13C NMR at 100.63 MHz in CDCl3

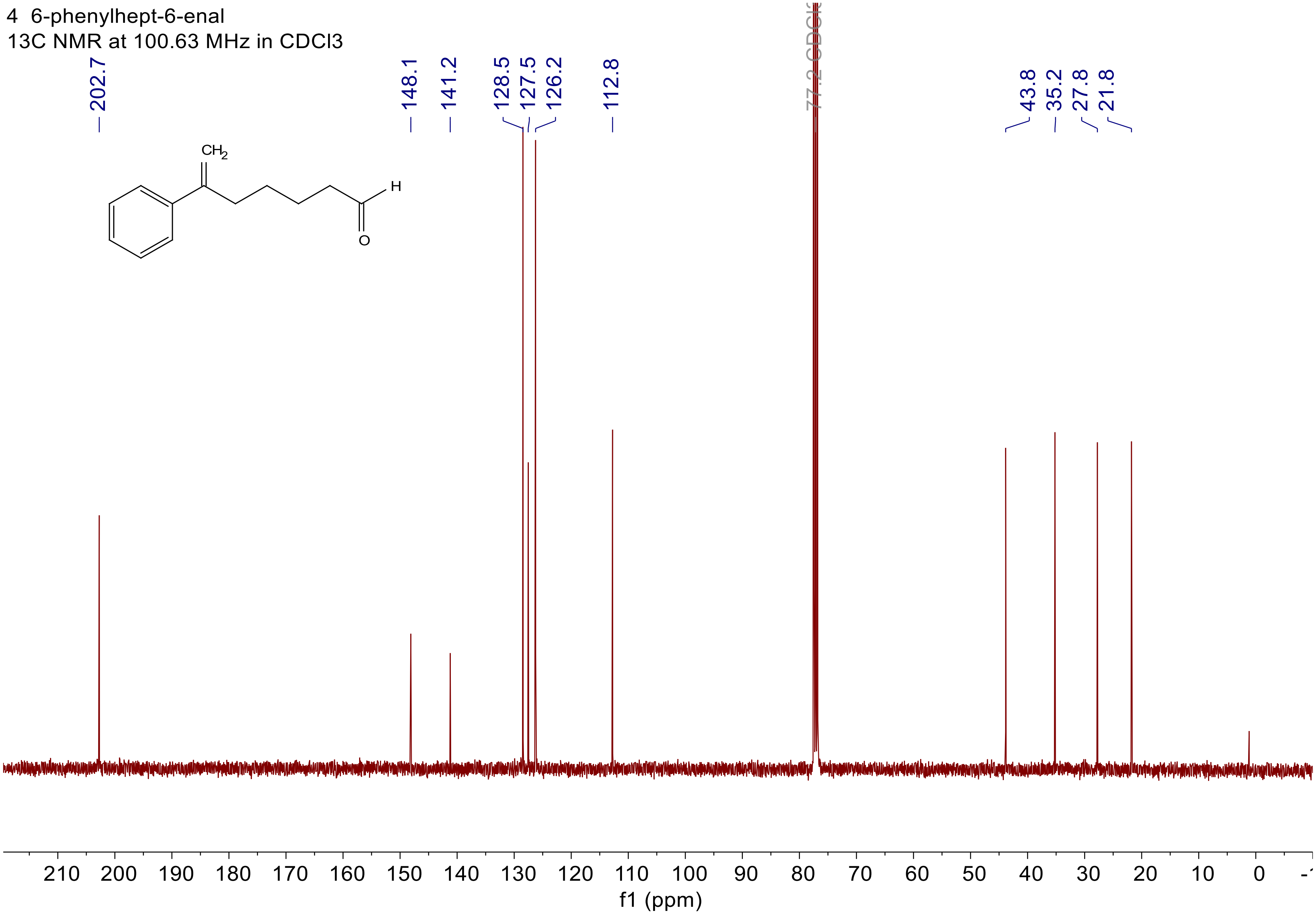

4 6-phenylhept-6-enal

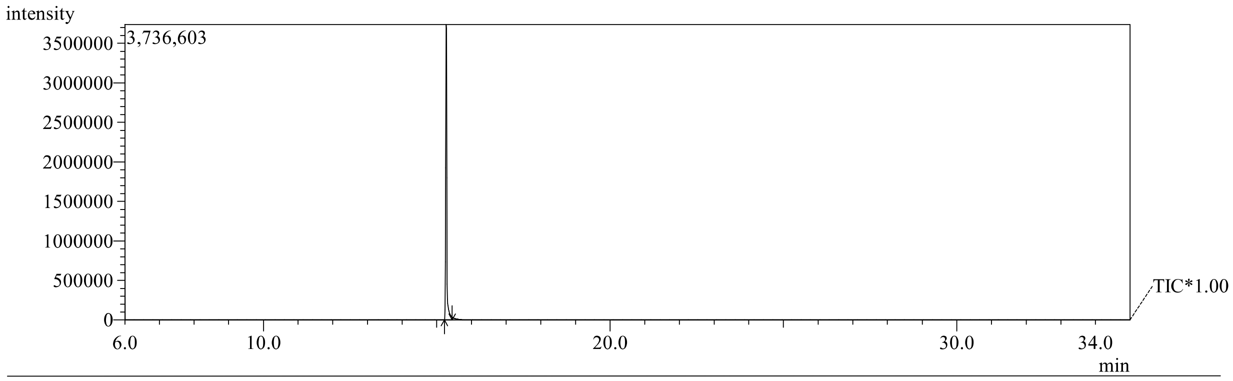

Spectrum

MassPeaks:51
RawMode:Averaged 15.3-15.3(1113-1115) BasePeak:118(525639)
BG Mode:Calc. from Peak

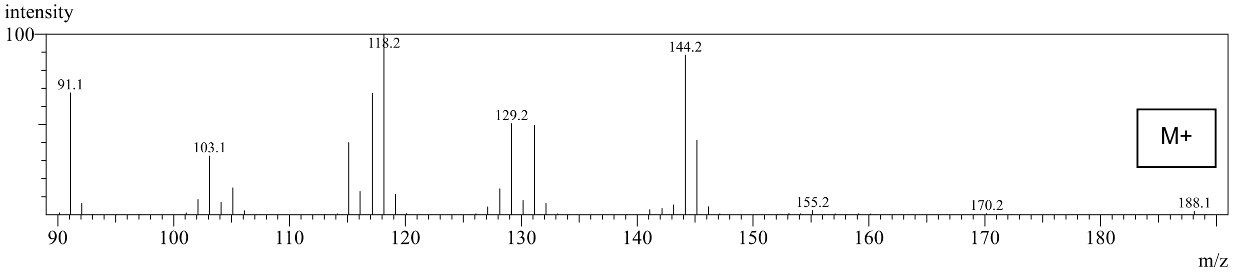

5 3-phenylcyclopent-2-en-1-ol
1H NMR at 400.15 MHz in CD3CN

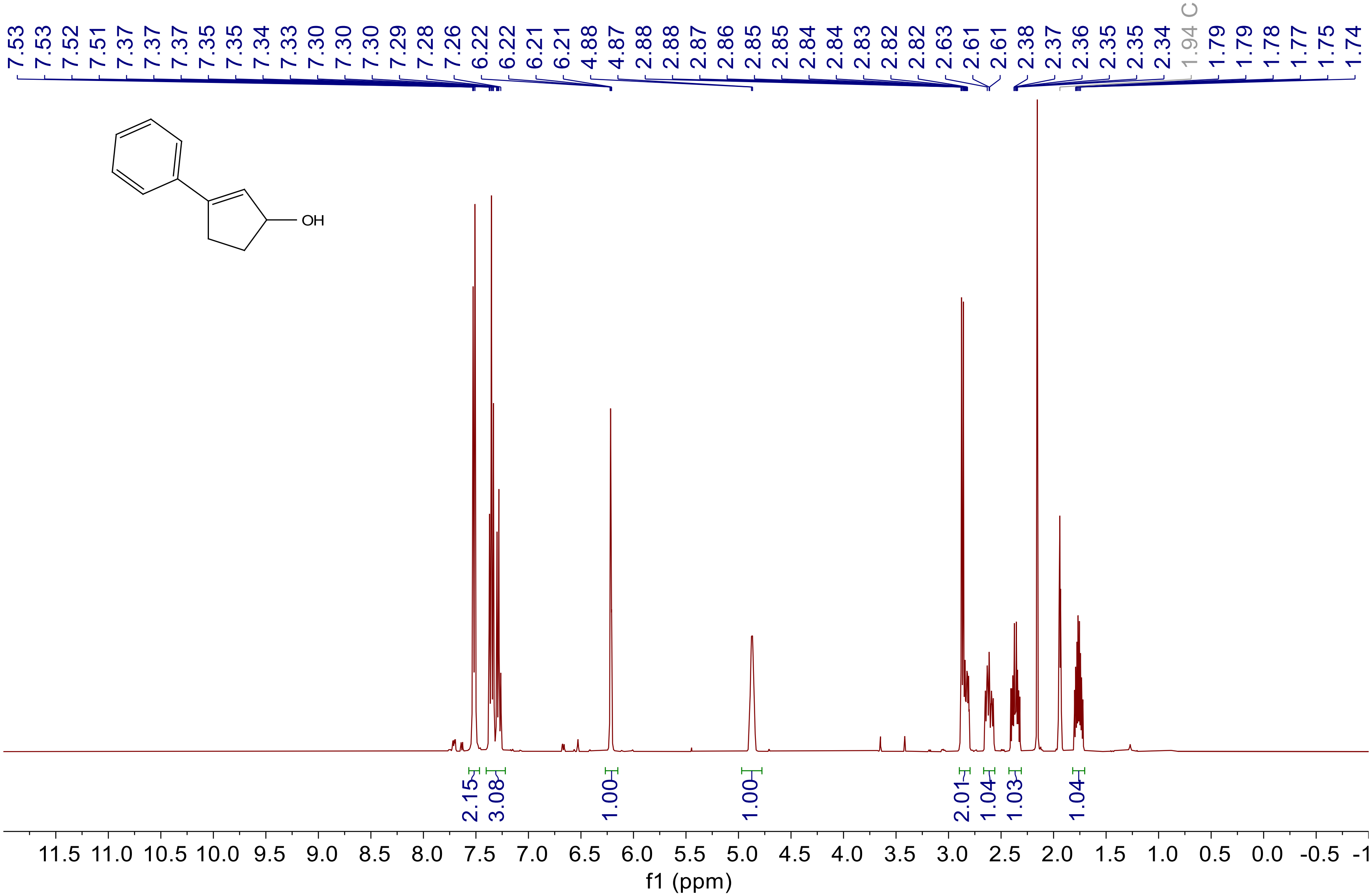

5 3-phenylcyclopent-2-en-1-ol
13C NMR at 100.63 MHz in CD3CN

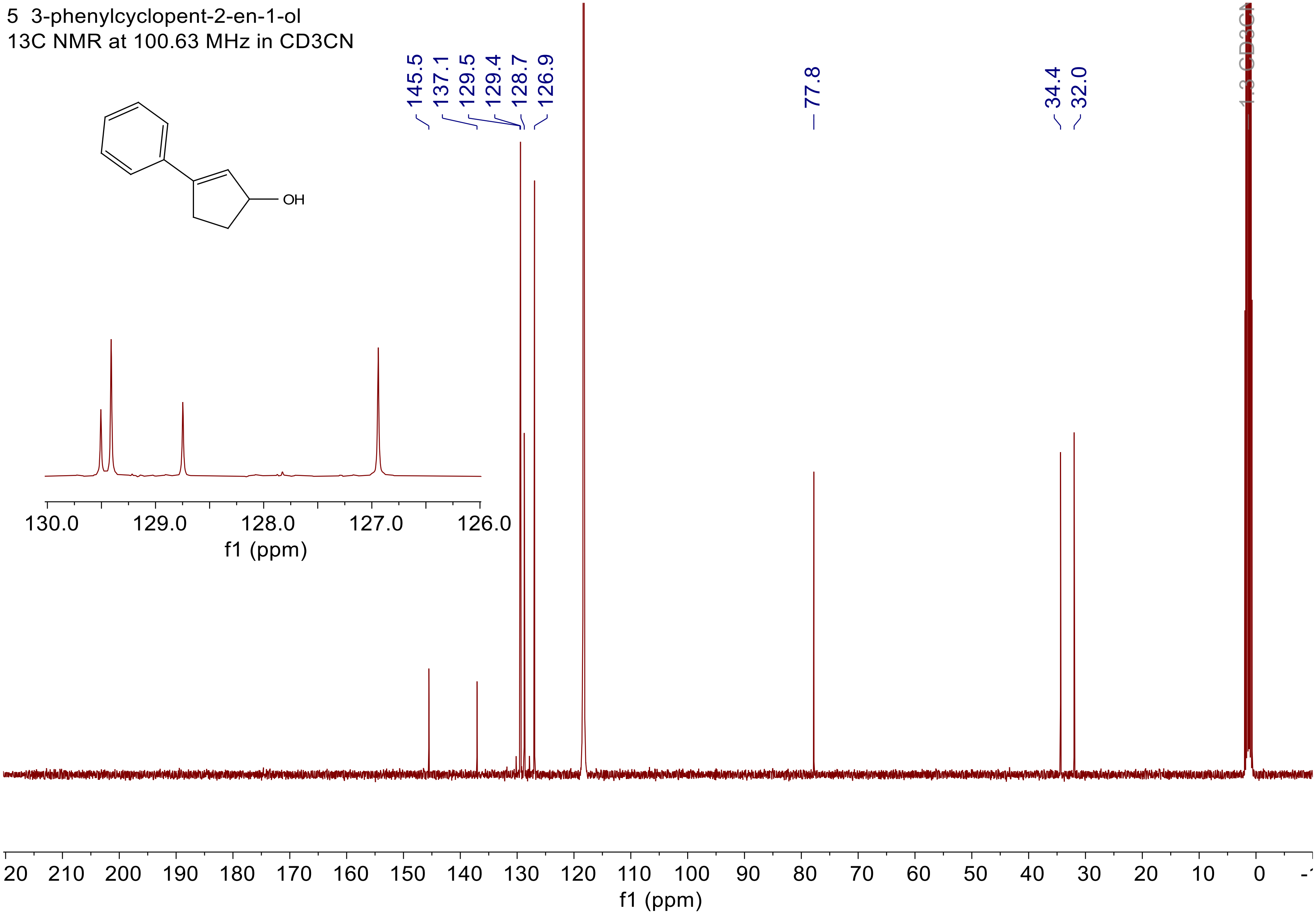

7 1',2',5',6'-tetrahydro-[1,1':3',1''-terphenyl]-5'-ol
1H NMR at 400.15 MHz in CD3CN

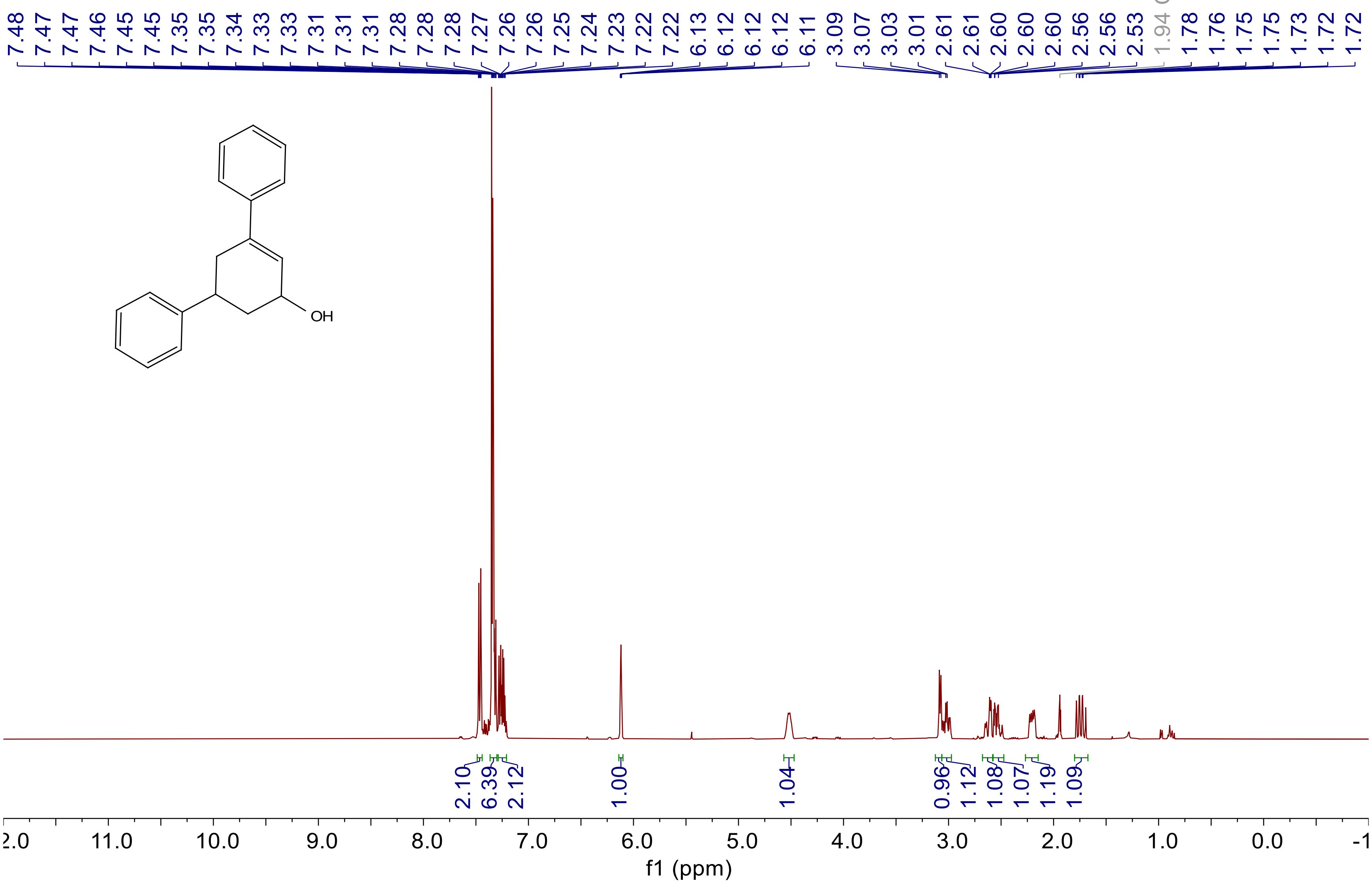

7 1',2',5',6'-tetrahydro-[1,1':3',1''-terphenyl]-5'-ol
13C NMR at 100.63 MHz in CD3CN

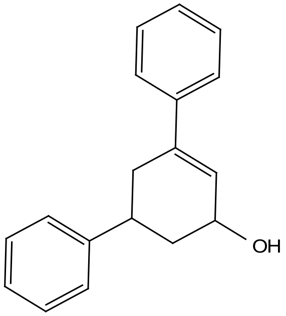

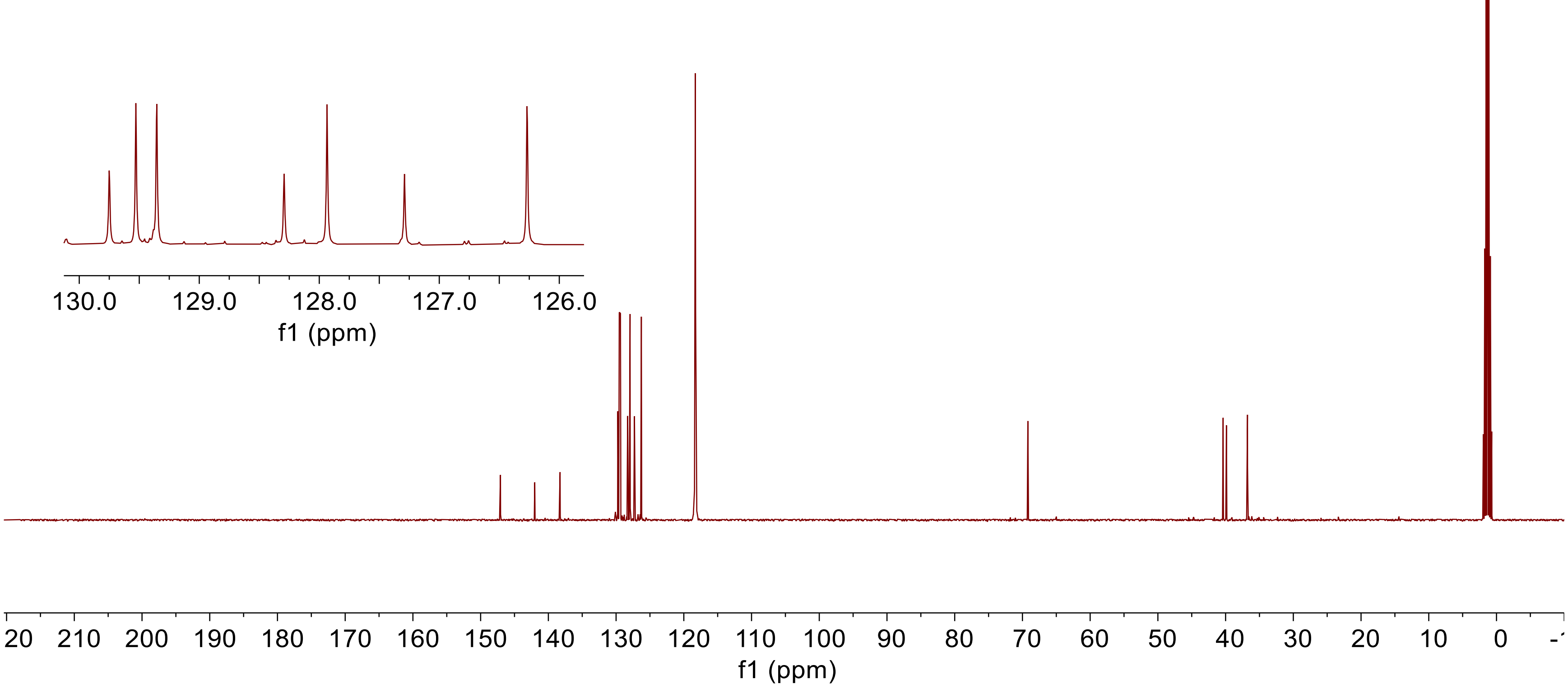

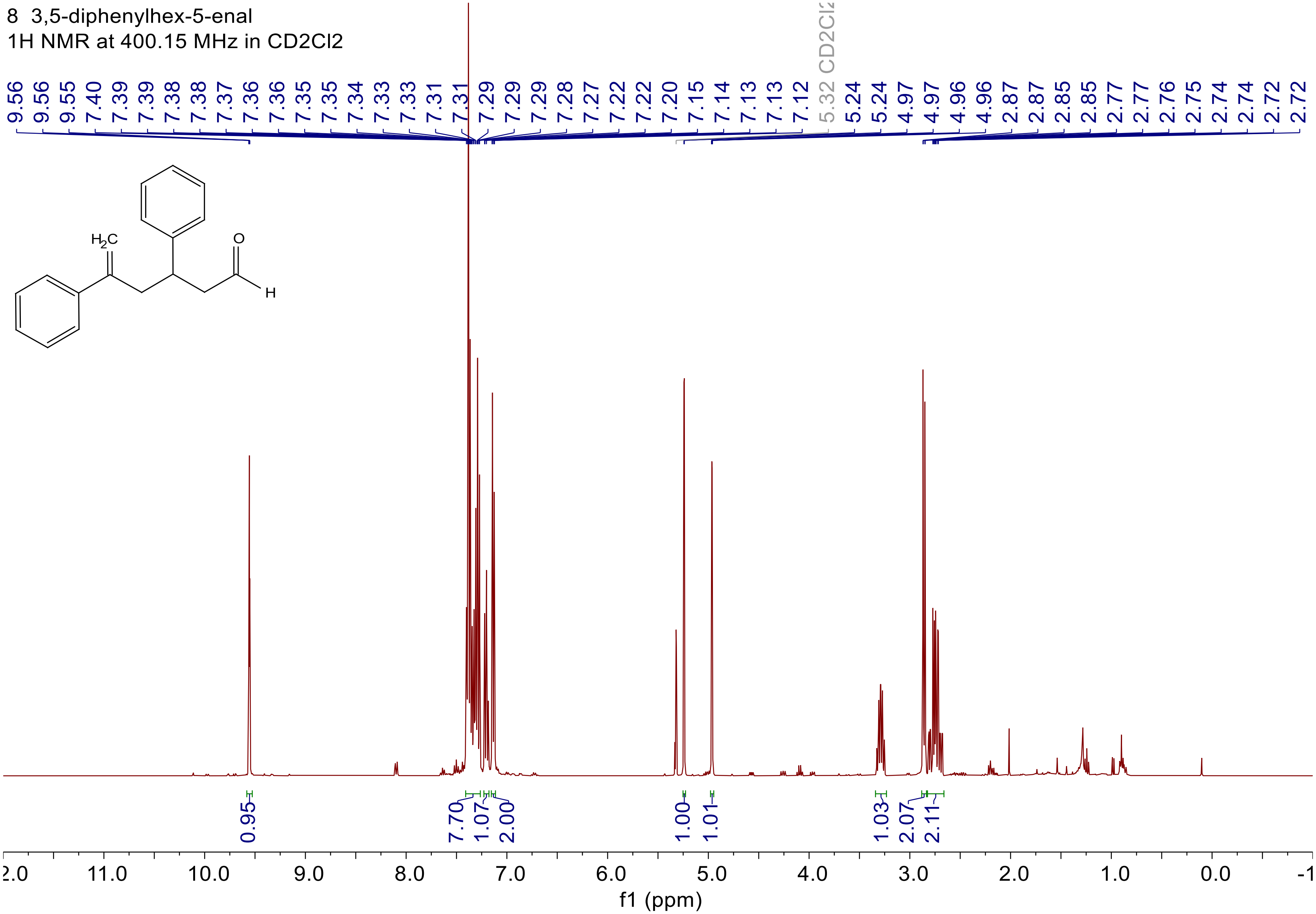
8 3,5-diphenylhex-5-enal
1H NMR at 400.15 MHz in CD2Cl2
9.56 9.56 9.55 7.40 7.39 7.39 7.38 7.38 7.37 7.36 7.36 7.35 7.35 7.34 7.33 7.33 7.31 7.31 7.29 7.29 7.29 7.28 7.27 7.22 7.22 7.20 7.15 7.14 7.13 7.13 7.12 5.32 CD2Cl2 5.24 5.24 4.97 4.97 4.96 4.96 2.87 2.87 2.85 2.85 2.77 2.77 2.76 2.75 2.74 2.74 2.72 2.72
H2C
O
H
0.95
7.70
1.07
2.00
1.00
1.01
1.03
2.07
2.11
f1 (ppm)

8 3,5-diphenylhex-5-enal
13C NMR at 100.63 MHz in CD2Cl2

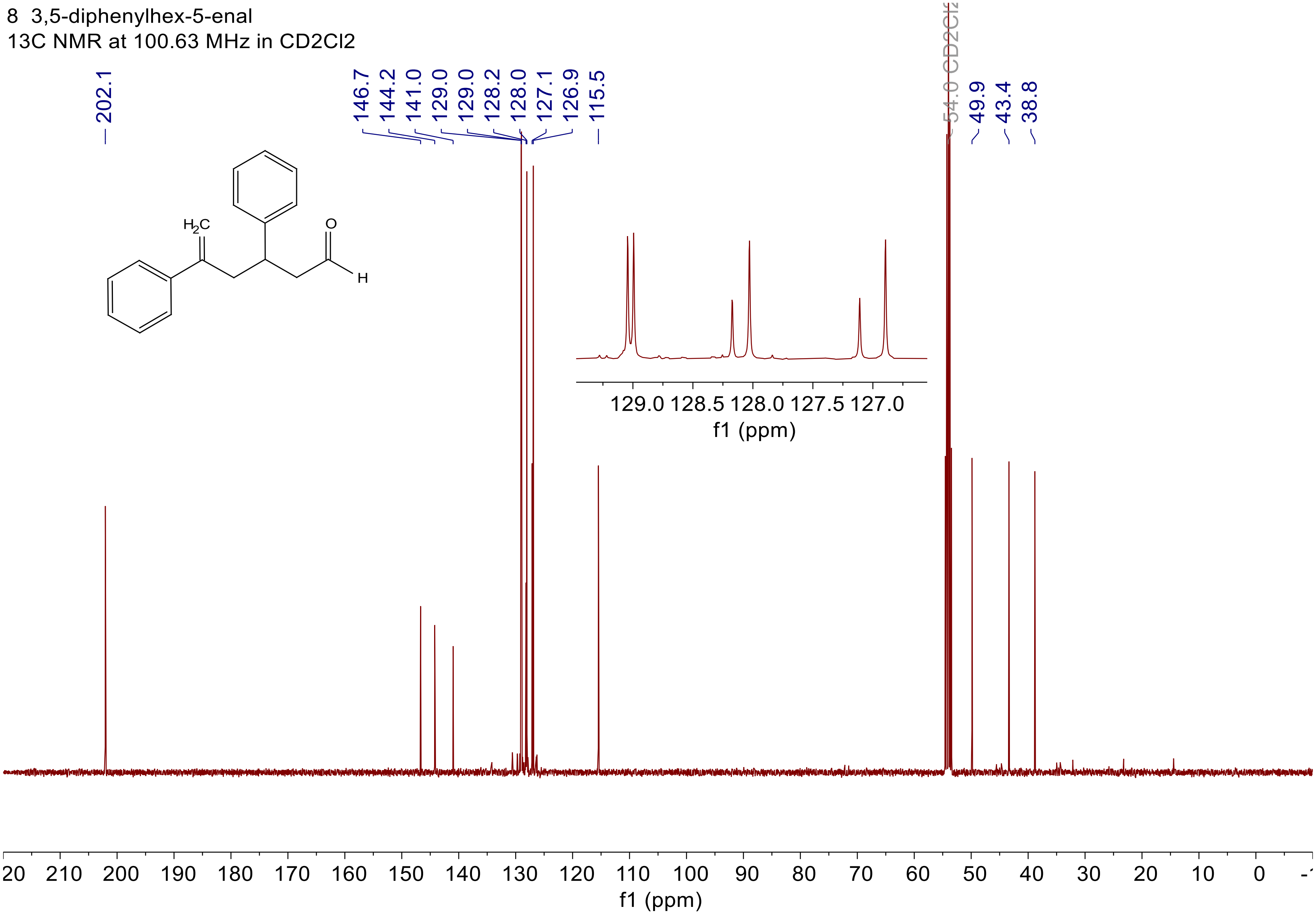

8 3,5-diphenylhex-5-enal

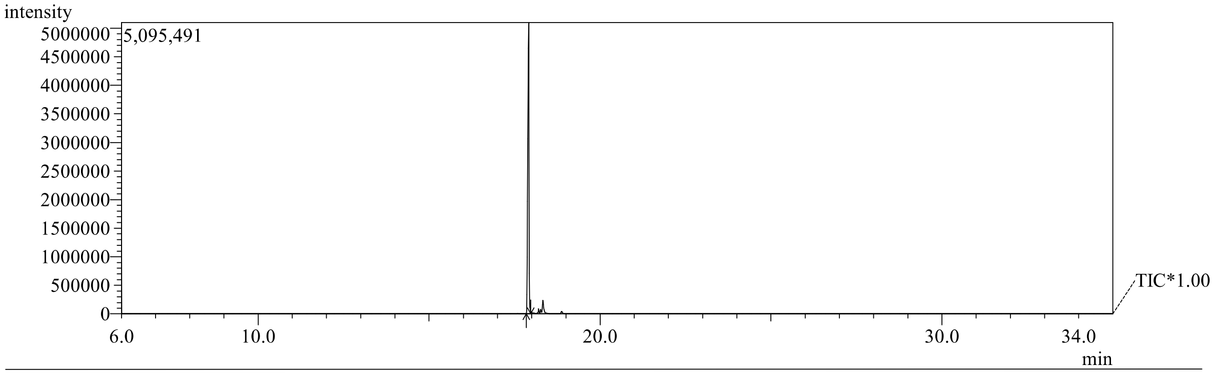

Spectrum

MassPeaks:108
RawMode:Averaged 17.9-17.9(1430-1432) BasePeak:105(1302493)
BG Mode:Calc. from Peak

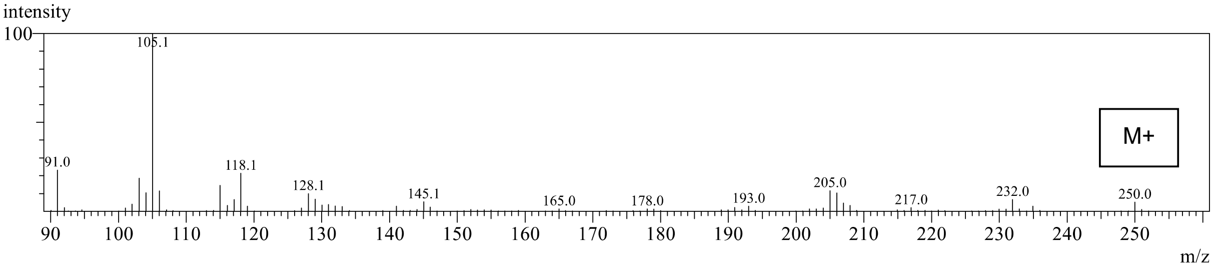

9 5'-phenyl-4-(trifluoromethyl)-3',4'-dihydro-[1,1':3',1''-terphenyl]-1'(2'H)-ol
1H NMR at 400.15 MHz in CD2Cl2

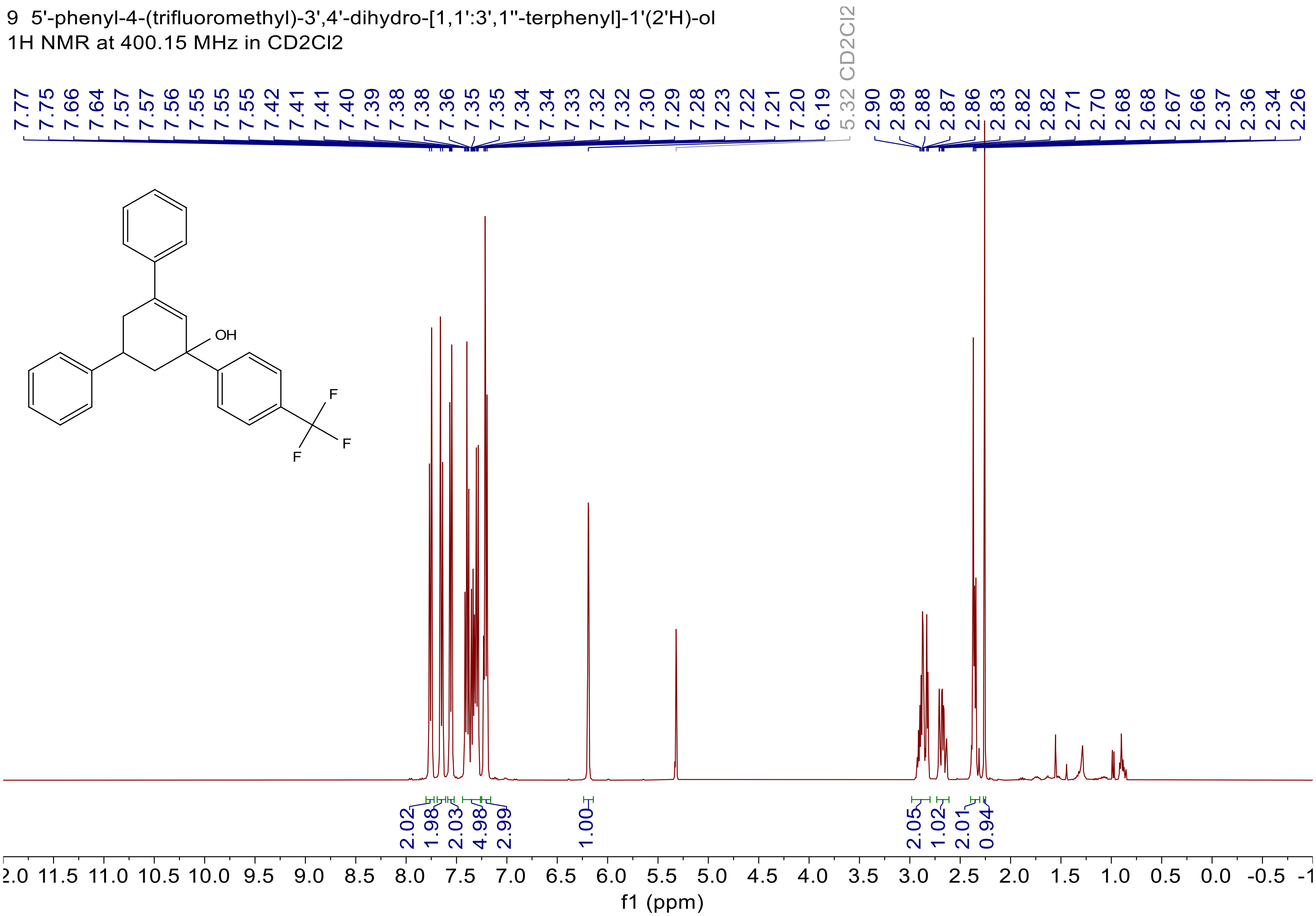

9 5'-phenyl-4-(trifluoromethyl)-3',4'-dihydro-[1,1':3',1''-terphenyl]-1'(2'H)-ol
19F NMR at 376.48 MHz in CD2Cl2

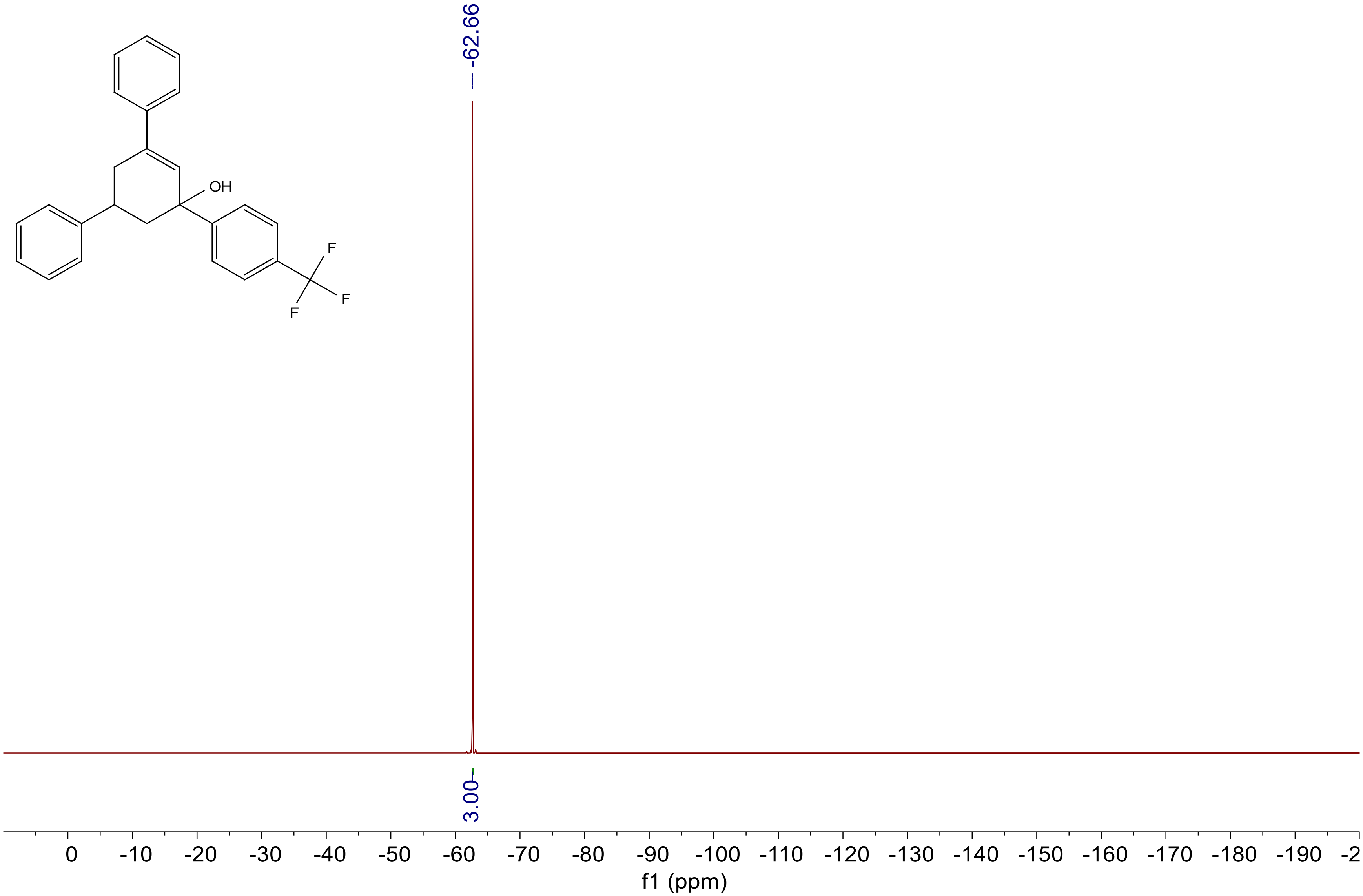

9 5'-phenyl-4-(trifluoromethyl)-3',4'-dihydro-[1,1':3',1''-terphenyl]-1'(2'H)-ol
13C NMR at 100.63 MHz in CD2Cl2

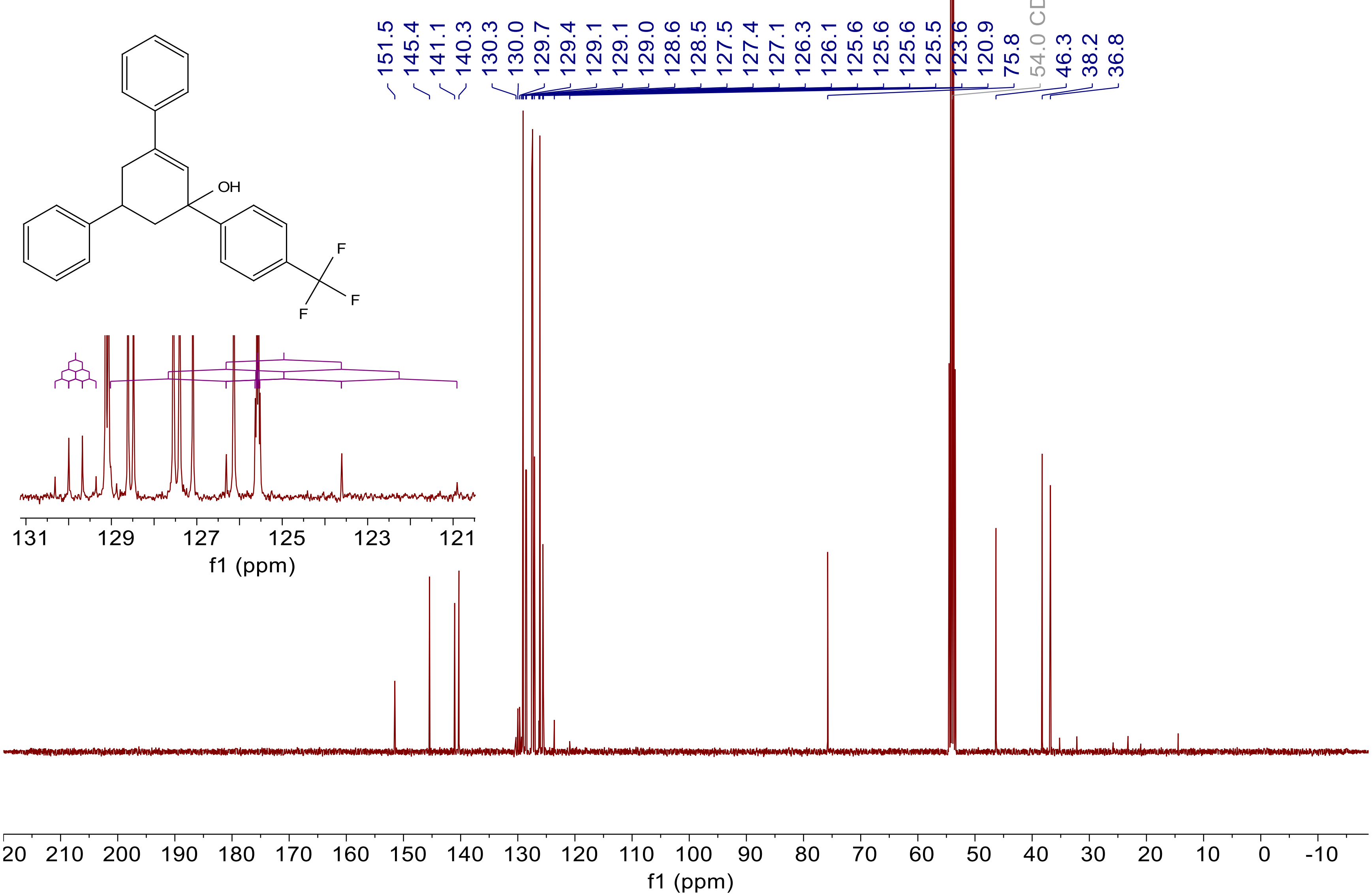

10 3,5-diphenyl-1-(4-(trifluoromethyl)phenyl)hex-5-en-1-one
1H NMR at 400.15 MHz in CD2Cl2

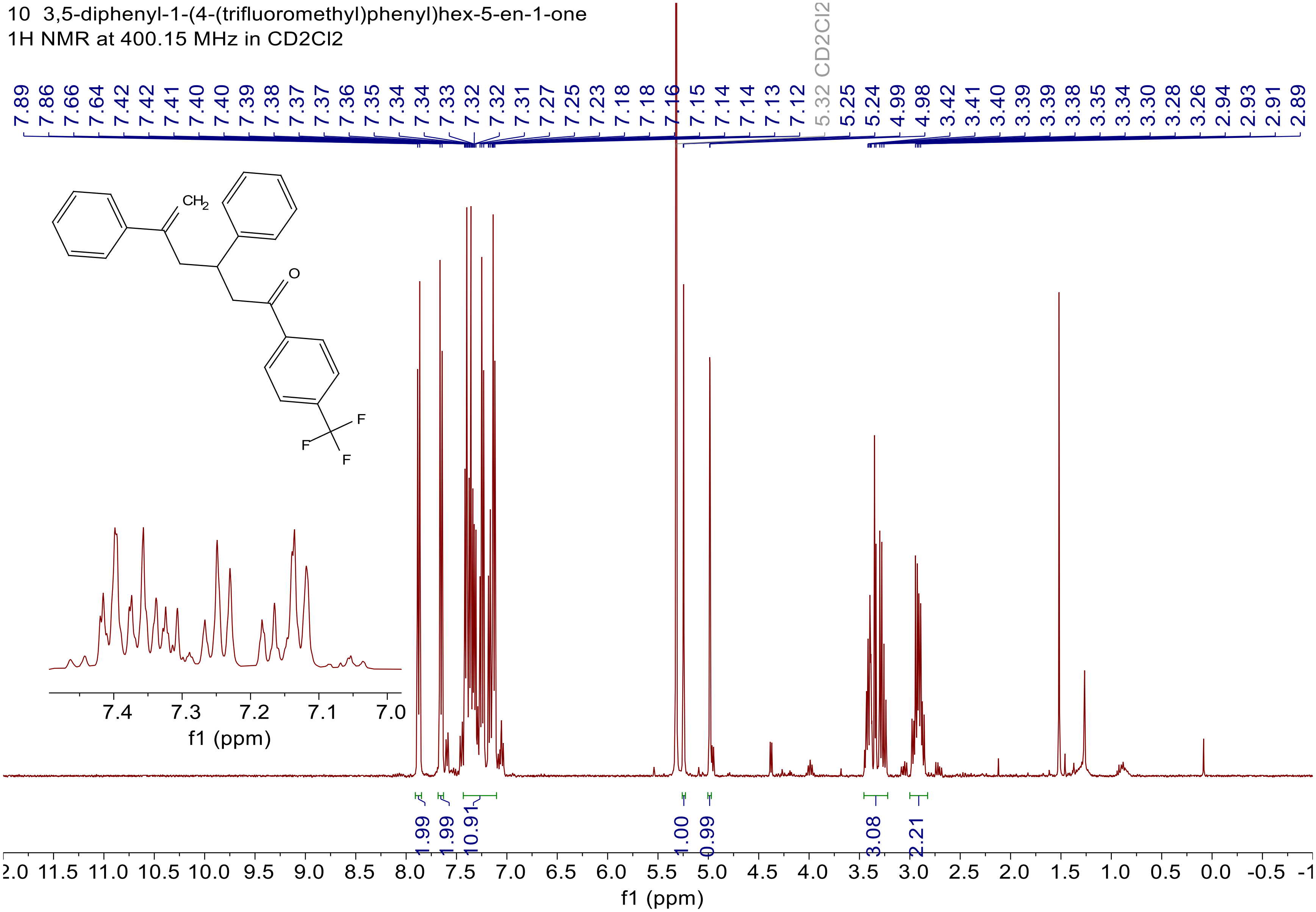

10 3,5-diphenyl-1-(4-(trifluoromethyl)phenyl)hex-5-en-1-one
19F NMR at 376.48 MHz in CD2Cl2

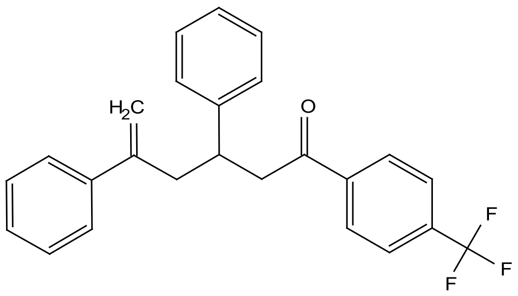

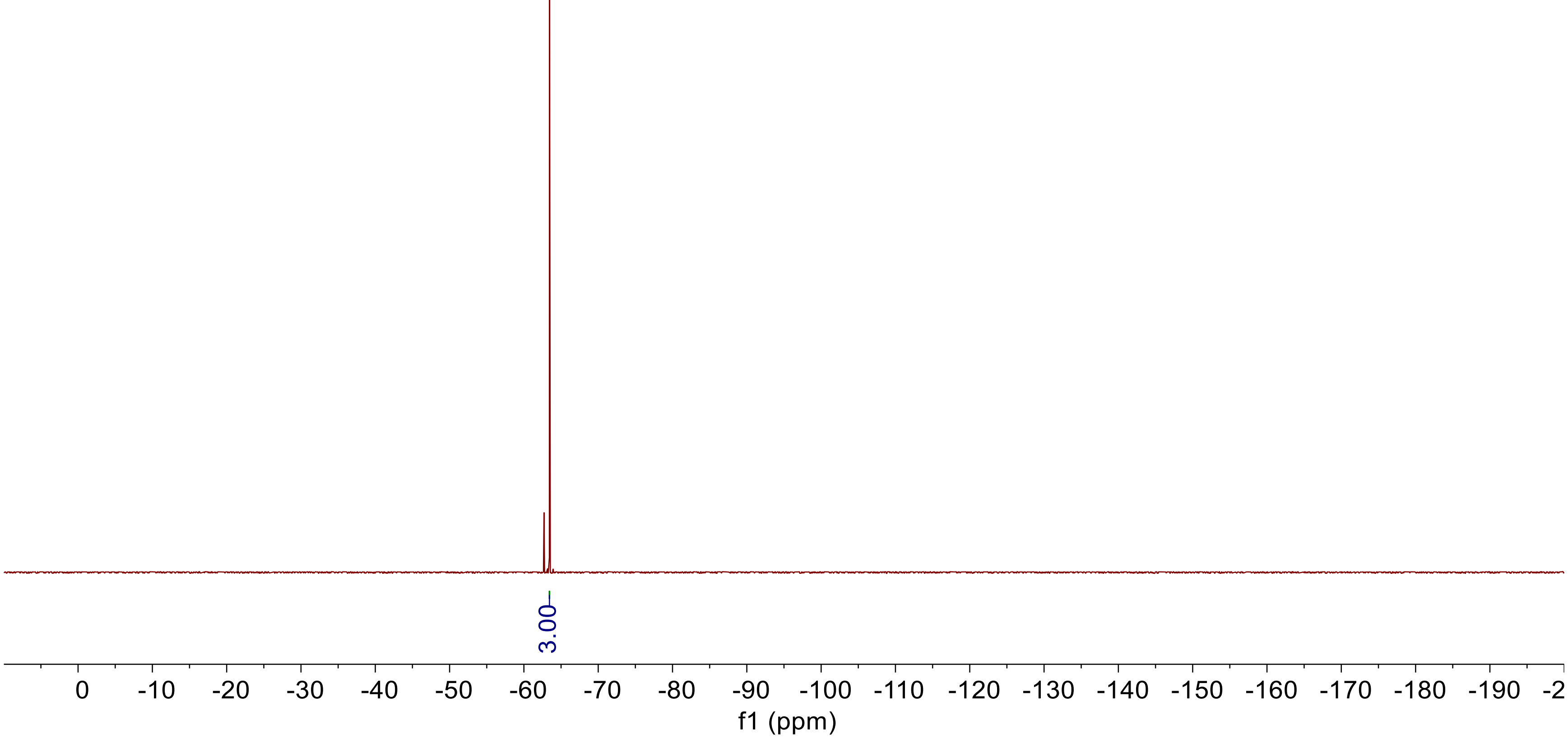

10 3,5-diphenyl-1-(4-(trifluoromethyl)phenyl)hex-5-en-1-one
13C NMR at 100.63 MHz in CD2Cl2

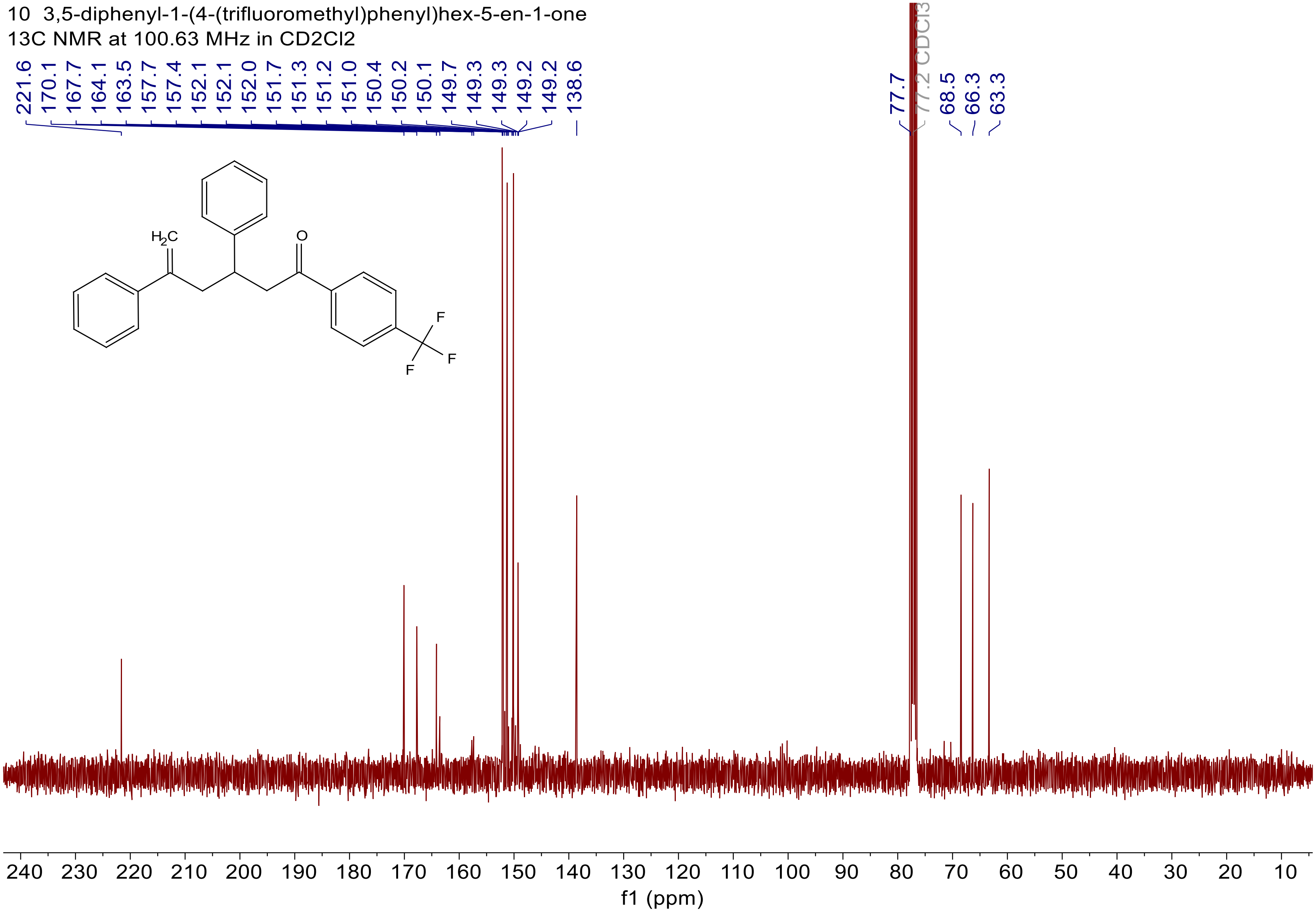

10 3,5-diphenyl-1-(4-(trifluoromethyl)phenyl)hex-5-en-1-one

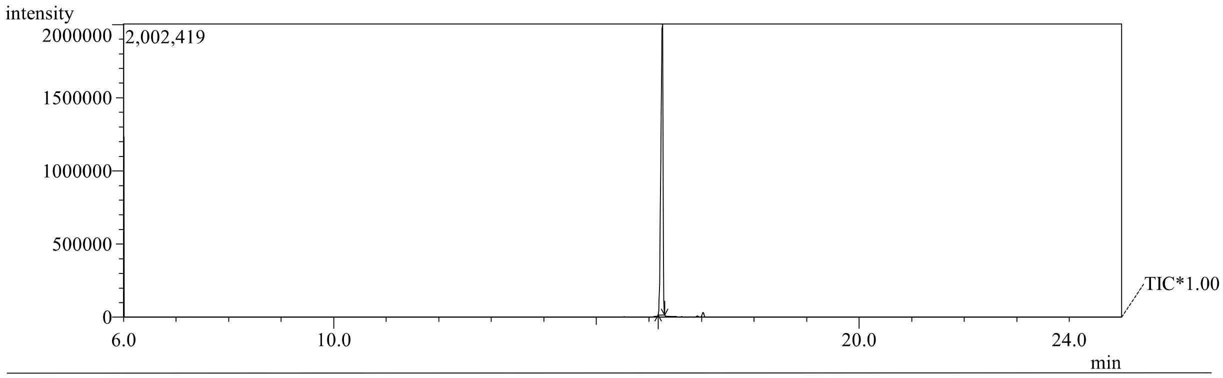

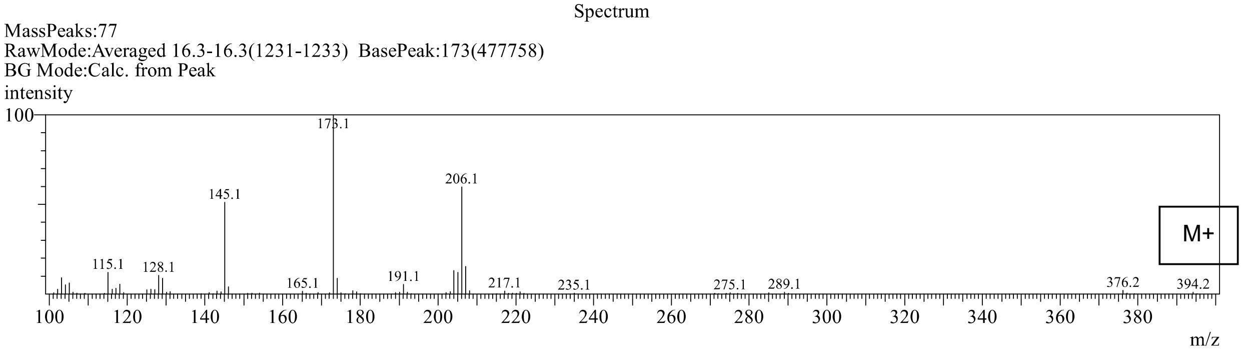

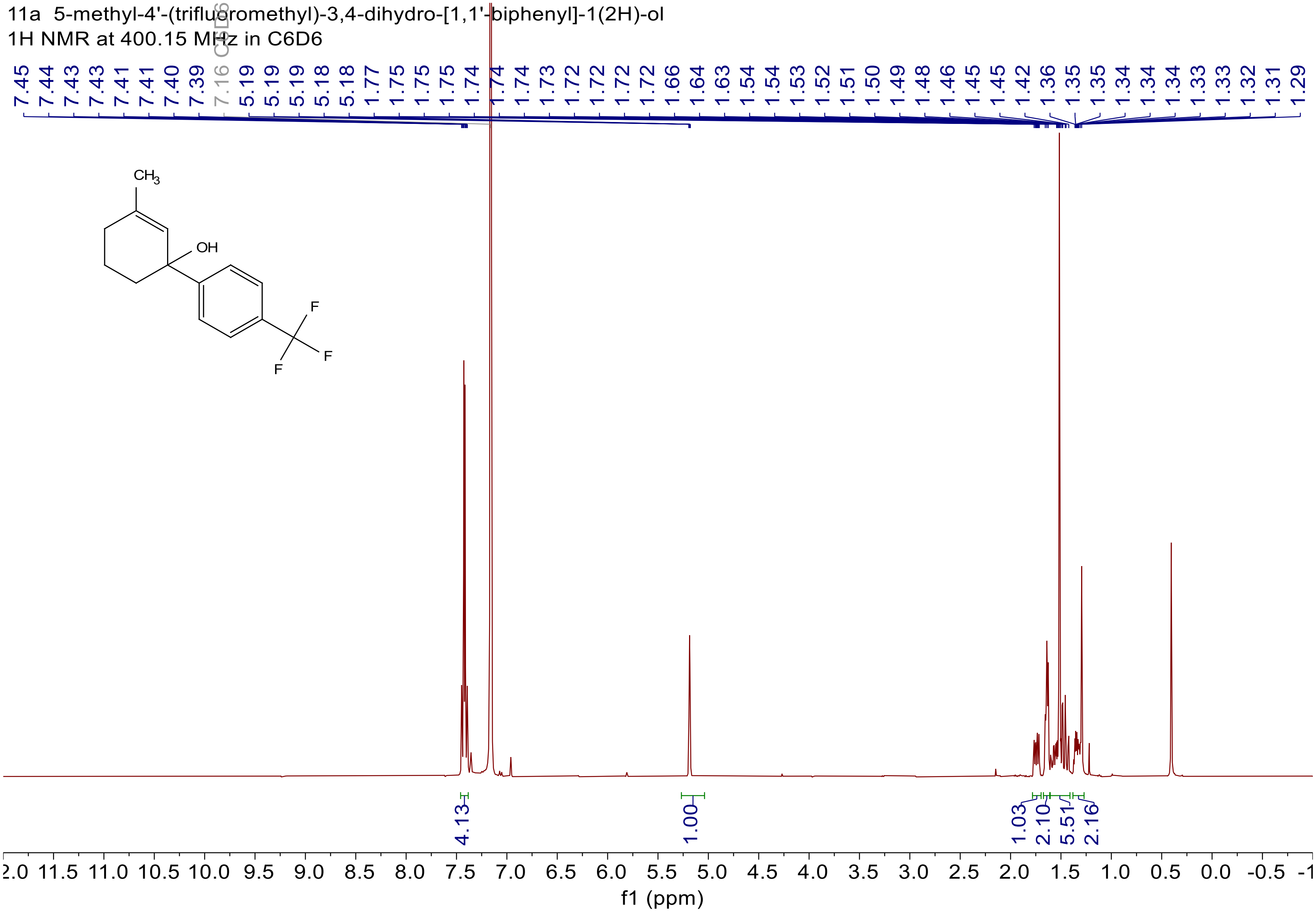
11a 5-methyl-4'-(trifluoromethyl)-3,4-dihydro-[1,1'-biphenyl]-1(2H)-ol
1H NMR at 400.15 MHz in C6D6
7.45 7.44 7.43 7.43 7.41 7.41 7.40 7.39 7.16 C6D6 5.19 5.19 5.19 5.18 5.18 1.77 1.75 1.75 1.75 1.74 1.74 1.74 1.73 1.72 1.72 1.72 1.72 1.66 1.64 1.63 1.54 1.54 1.53 1.52 1.51 1.50 1.49 1.48 1.46 1.45 1.45 1.42 1.36 1.35 1.35 1.34 1.34 1.34 1.33 1.33 1.32 1.31 1.29
CH3
OH
F
F
F
4.13
1.00
1.03
2.10
5.51
2.16
f1 (ppm)

11a 5-methyl-4'-(trifluoromethyl)-3,4-dihydro-[1,1'-biphenyl]-1(2H)-ol
19F NMR at 376.48 MHz in C6D6

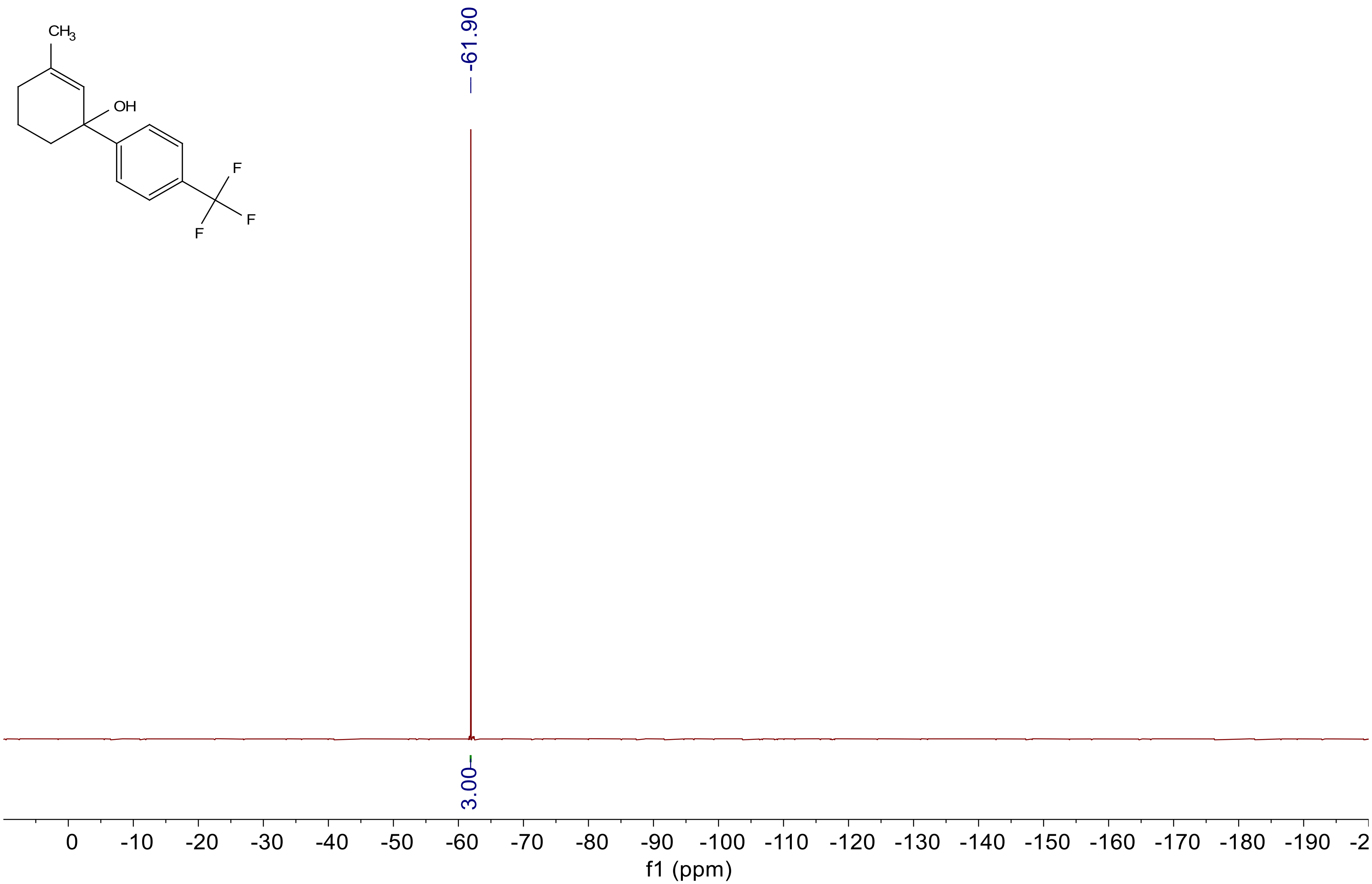

11a 5-methyl-4'-(trifluoromethyl)-3,4-dihydro-[1,1'-biphenyl]-1(2H)-ol
13C NMR at 100.63 MHz in C6D6

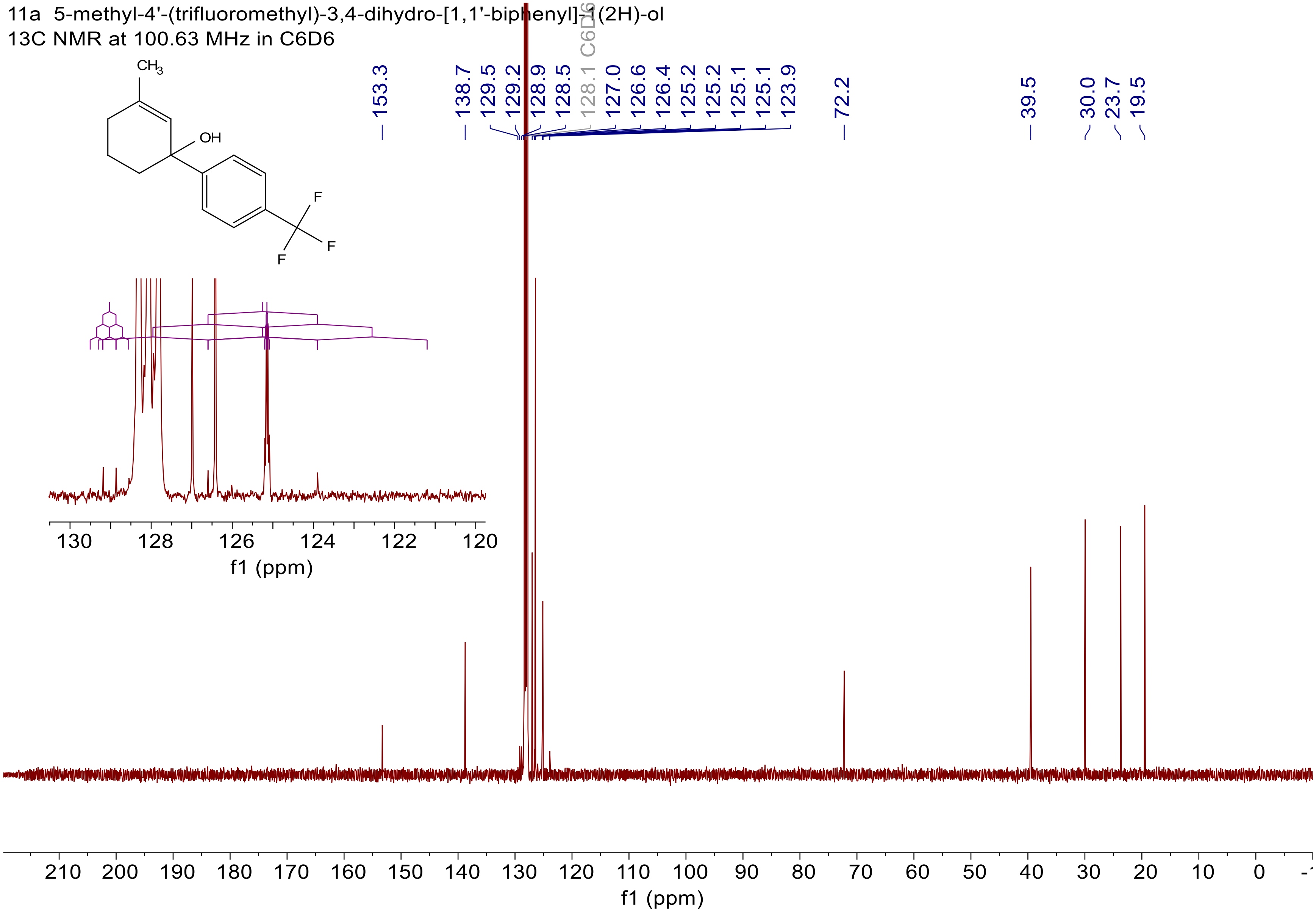

11a 5-methyl-4'-(trifluoromethyl)-3,4-dihydro-[1,1'-biphenyl]-1(2H)-ol

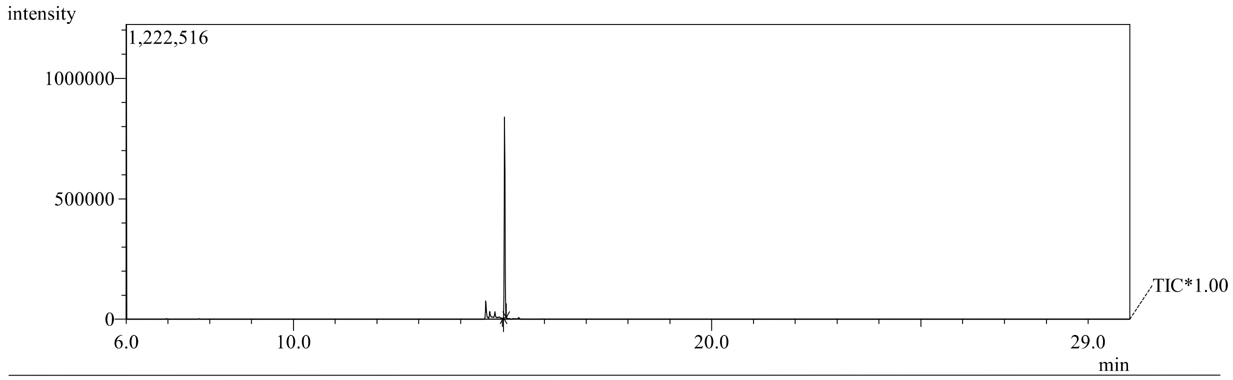

Spectrum

MassPeaks:104
RawMode:Averaged 15.0-15.1(1086-1088) BasePeak:159(85360)
BG Mode:Calc. from Peak

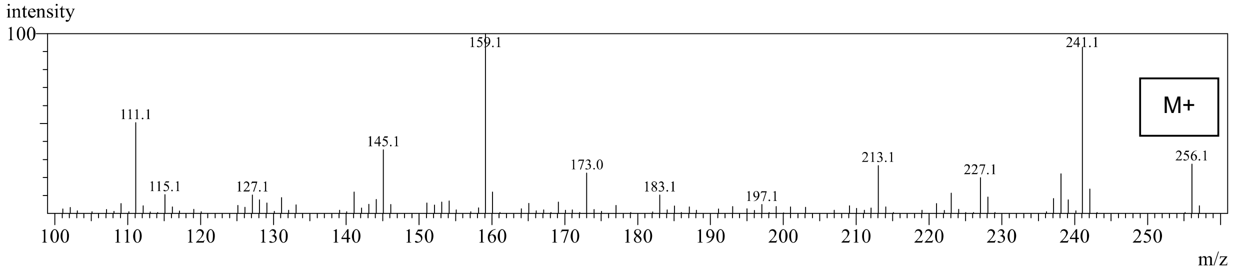

11b 3-methyl-4'-(trifluoromethyl)-3,4,5,6-tetrahydro-[1,1'-biphenyl]-3-ol
1H NMR at 400.15 MHz in CDCl3

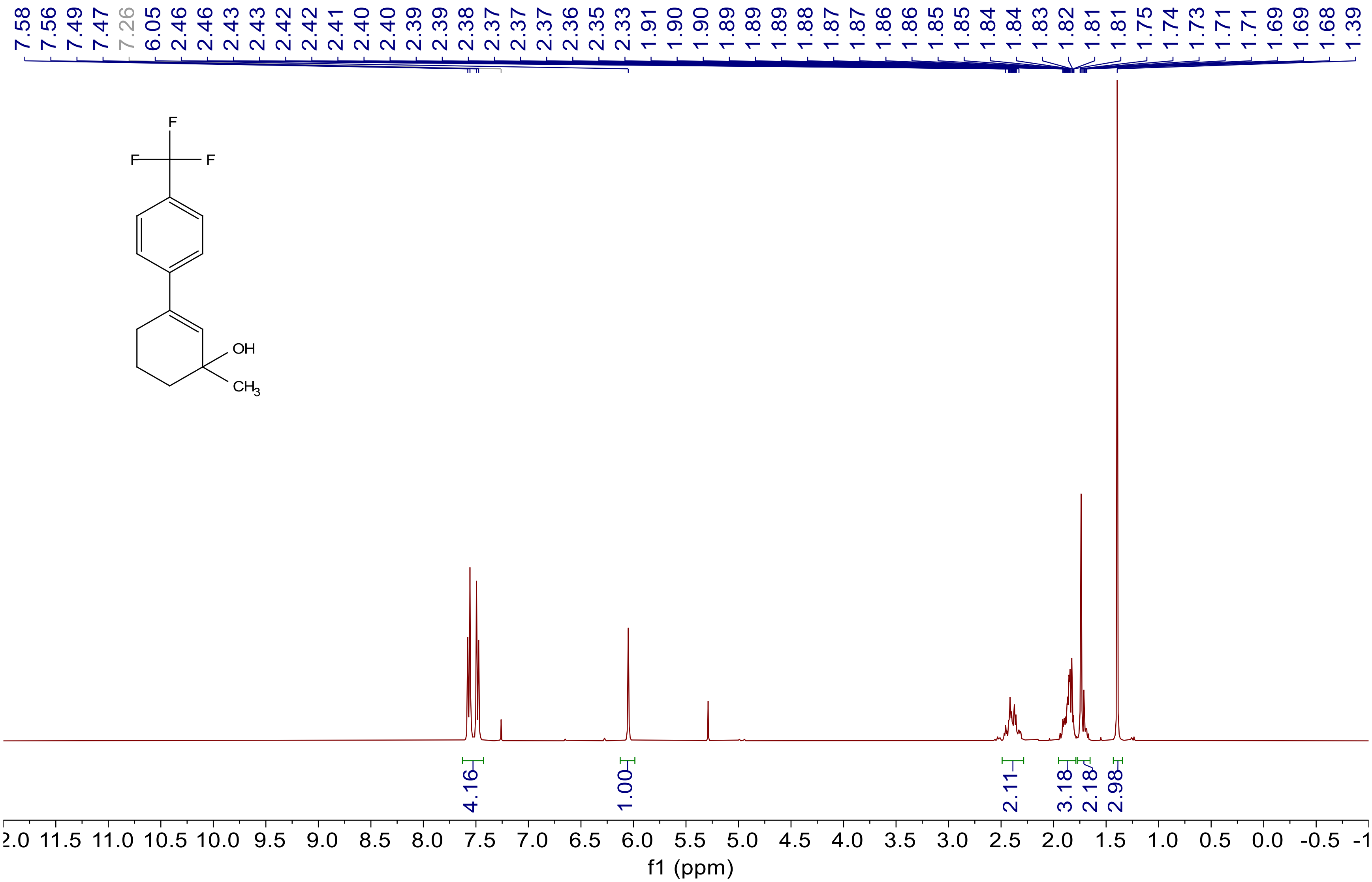

11b 3-methyl-4'-(trifluoromethyl)-3,4,5,6-tetrahydro-[1,1'-biphenyl]-3-ol
19F NMR at 376.48 MHz in CDCl3

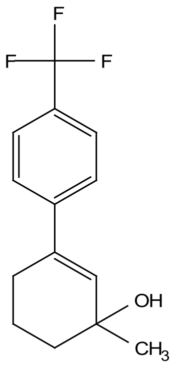

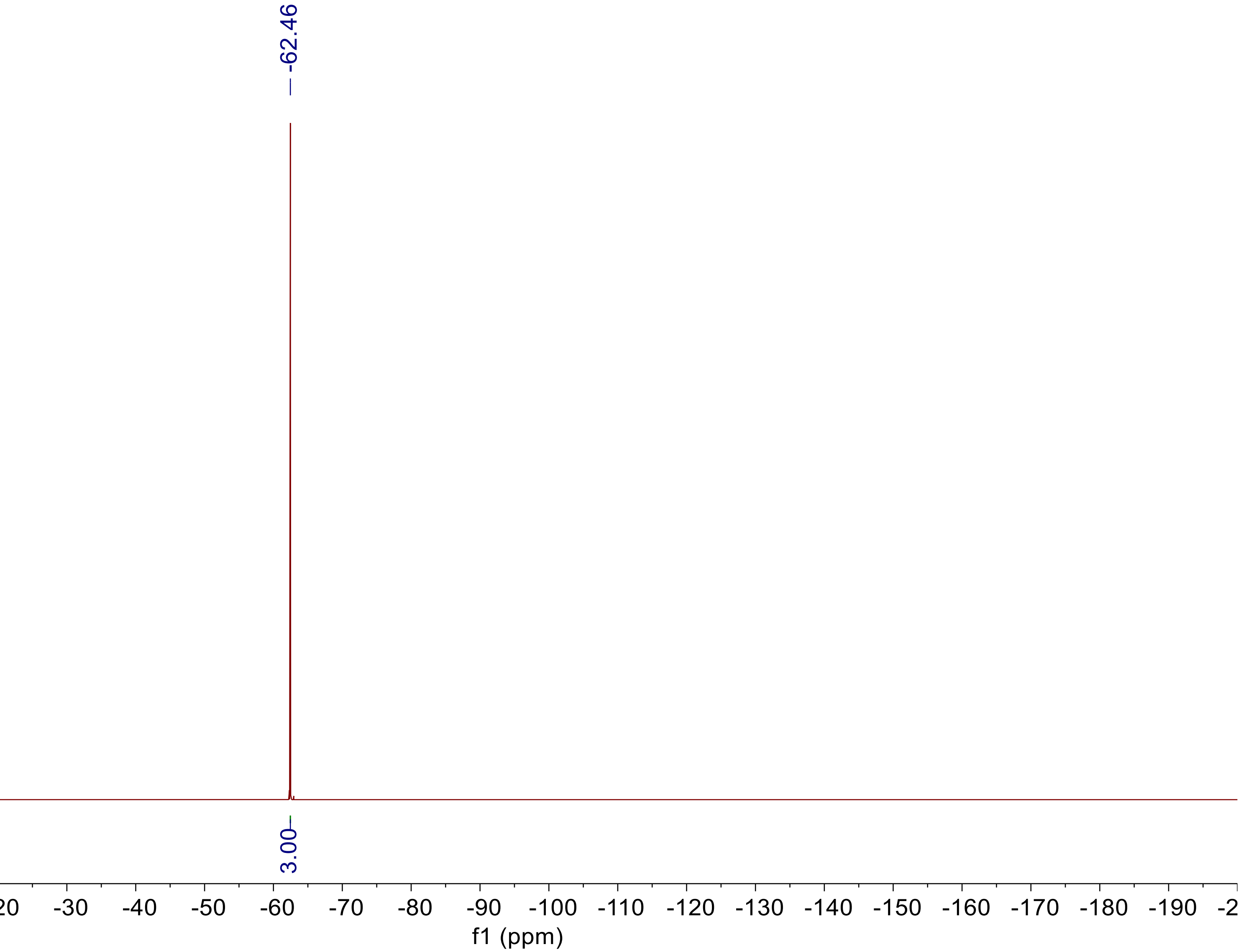

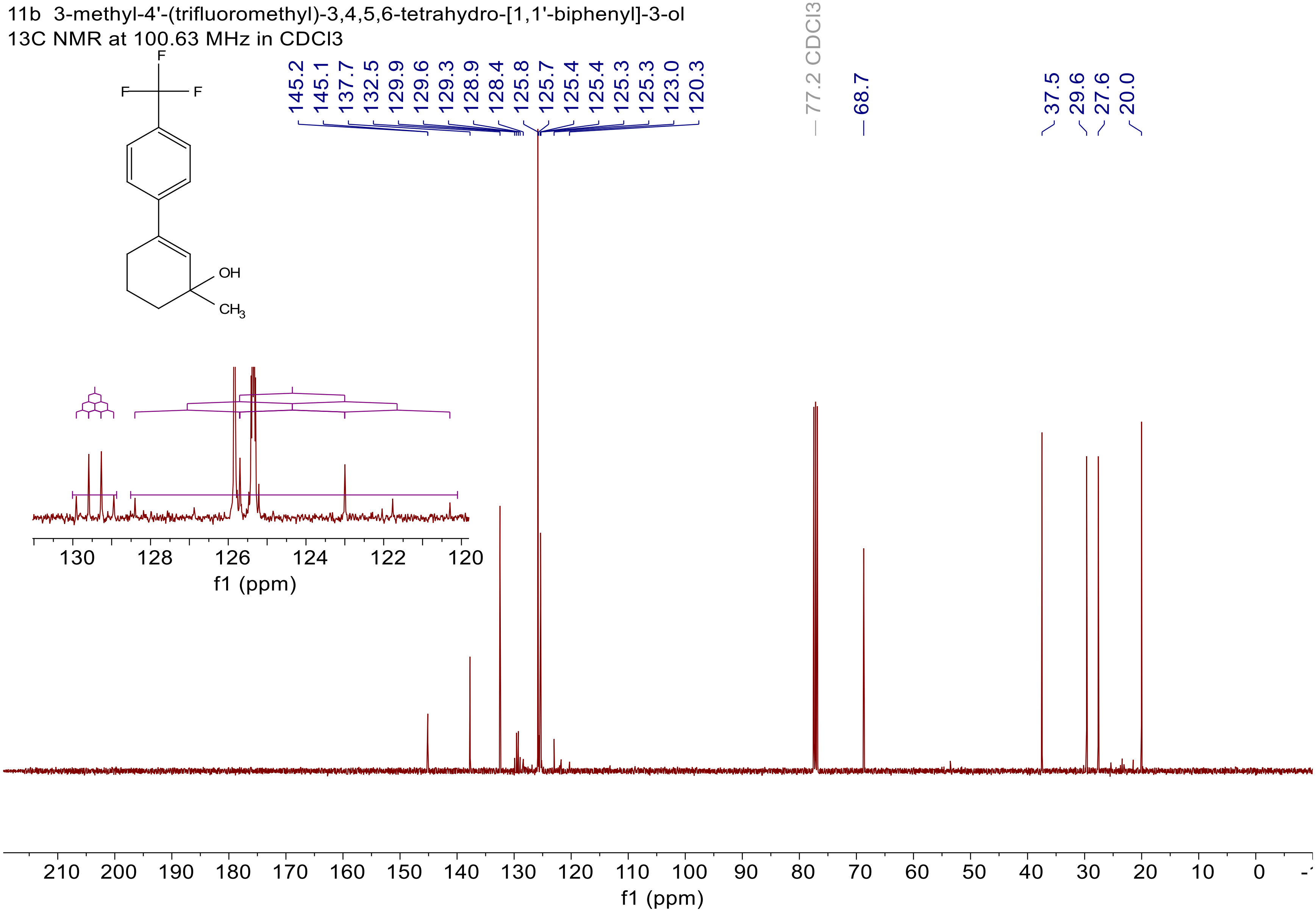
11b 3-methyl-4'-(trifluoromethyl)-3,4,5,6-tetrahydro-[1,1'-biphenyl]-3-ol
13C NMR at 100.63 MHz in CDCl3
F
F
F
OH
CH_3
145.2
145.1
137.7
132.5
129.9
129.6
129.3
128.9
128.4
125.8
125.7
125.4
125.4
125.3
125.3
123.0
120.3
77.2 CDCl3
68.7
37.5
29.6
27.6
20.0
130
128
126
124
122
120
f1 (ppm)
210
200
190
180
170
160
150
140
130
120
110
100
90
80
70
60
50
40
30
20
10
0
f1 (ppm)

11b 3-methyl-4'-(trifluoromethyl)-3,4,5,6-tetrahydro-[1,1'-biphenyl]-3-ol

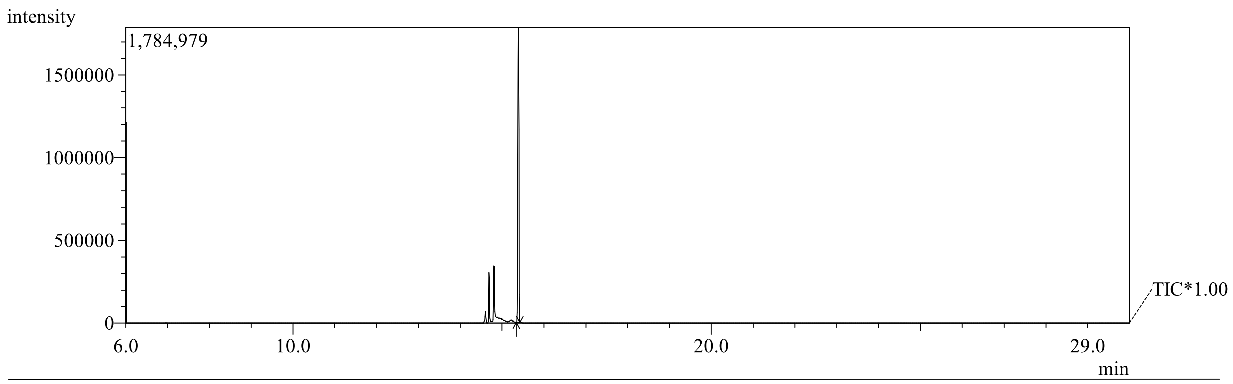

Spectrum

MassPeaks:122
RawMode:Averaged 15.4-15.4(1127-1129) BasePeak:241(383898)
BG Mode:Calc. from Peak

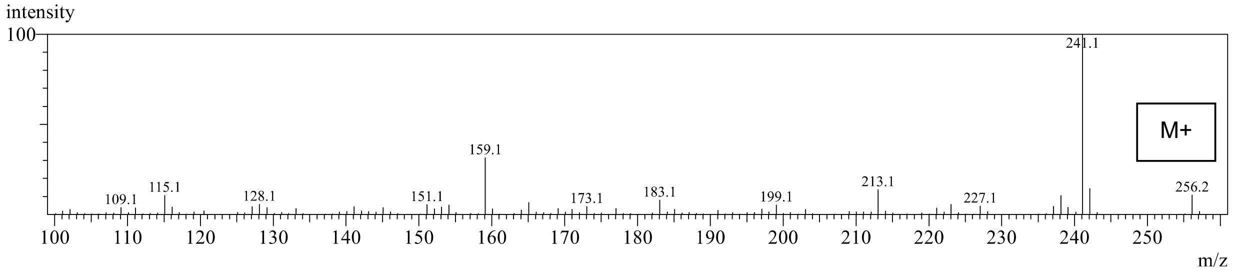

12 6-(4-(trifluoromethyl)phenyl)hept-6-en-2-one
1H NMR at 400.15 MHz in CDCl3

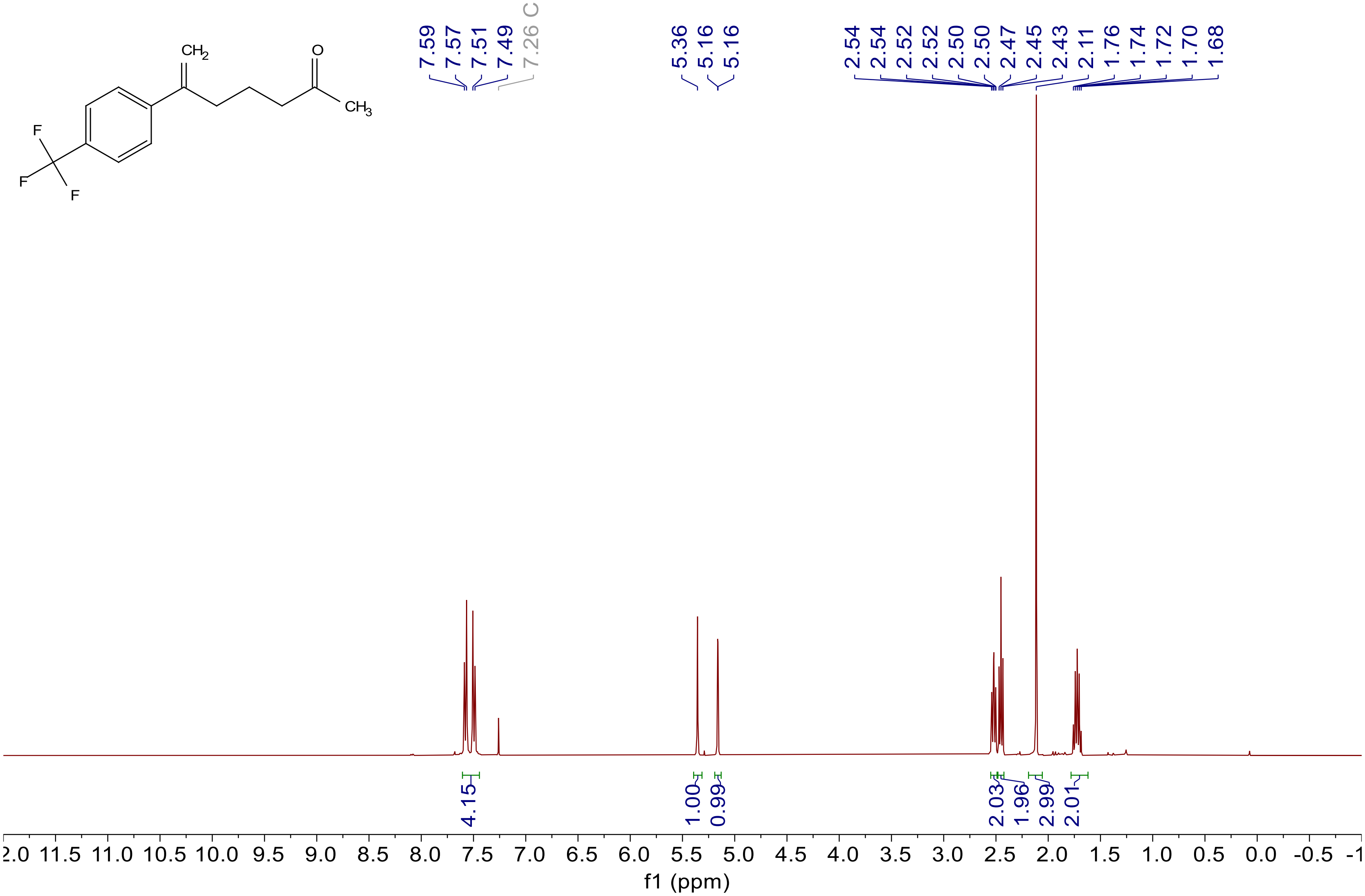

12 6-(4-(trifluoromethyl)phenyl)hept-6-en-2-one
19F NMR at 376.48 MHz in CDCl3

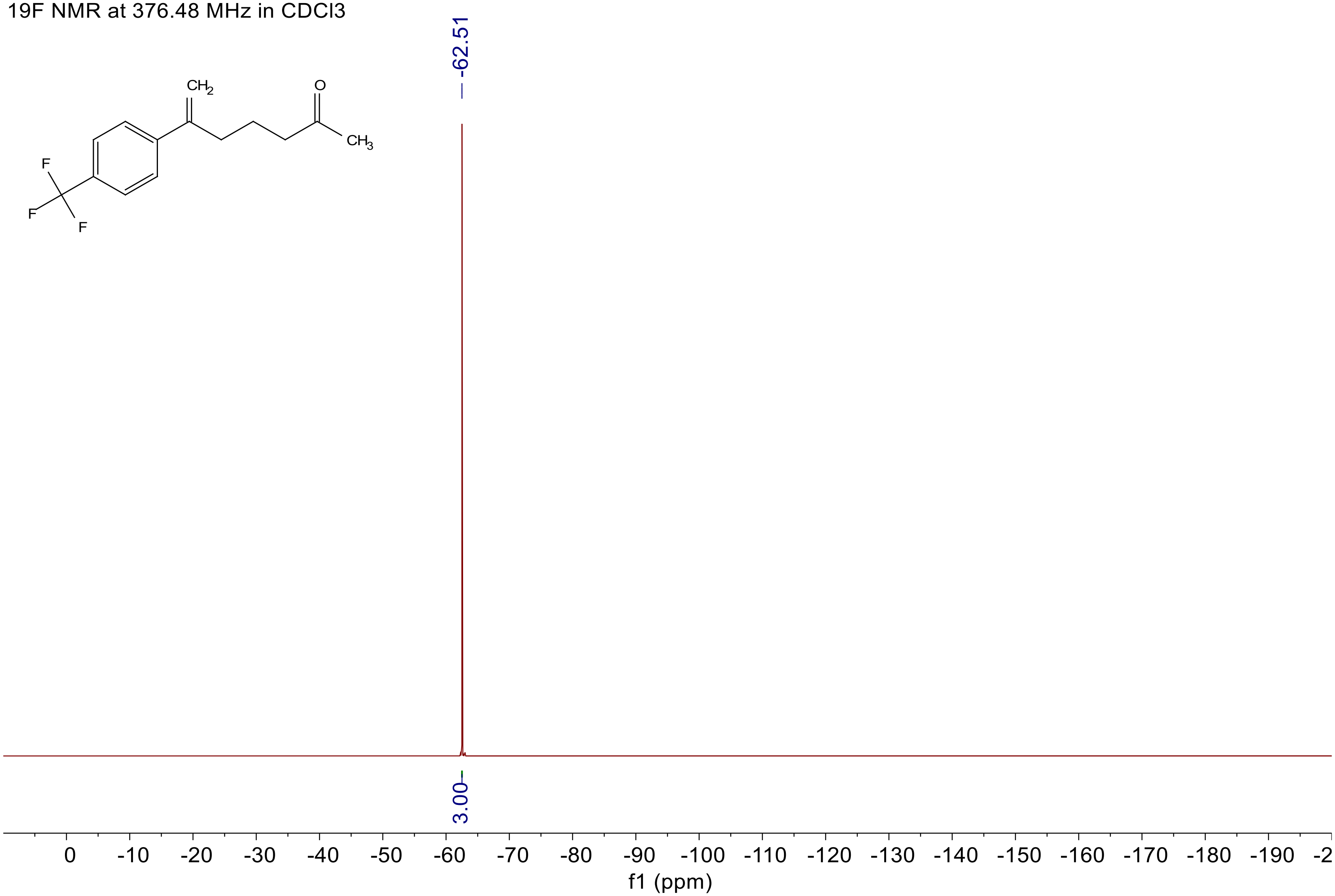

12 6-(4-(trifluoromethyl)phenyl)hept-6-en-2-one
13C NMR at 100.63 MHz in CDCl3

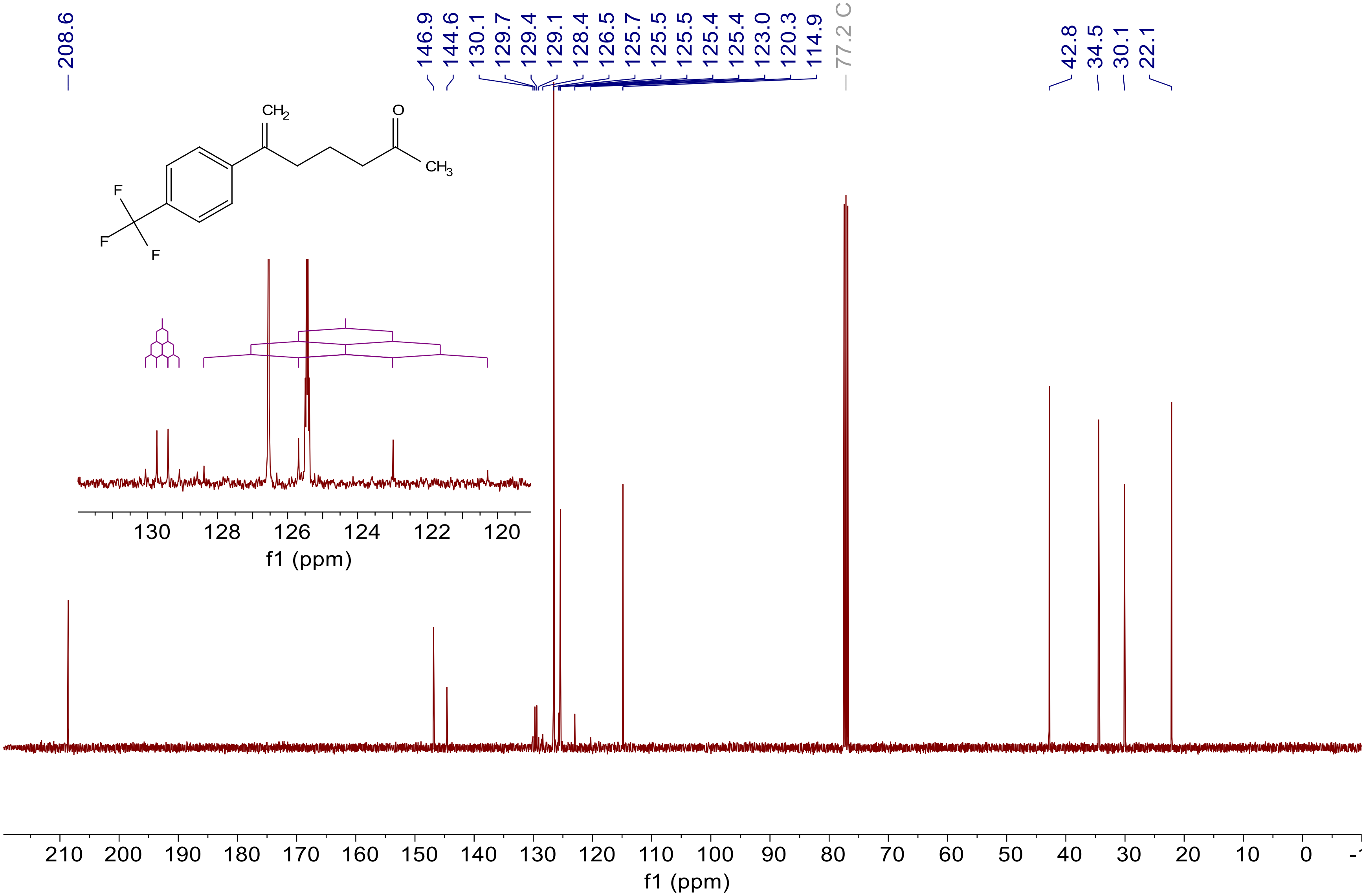

12 6-(4-(trifluoromethyl)phenyl)hept-6-en-2-one

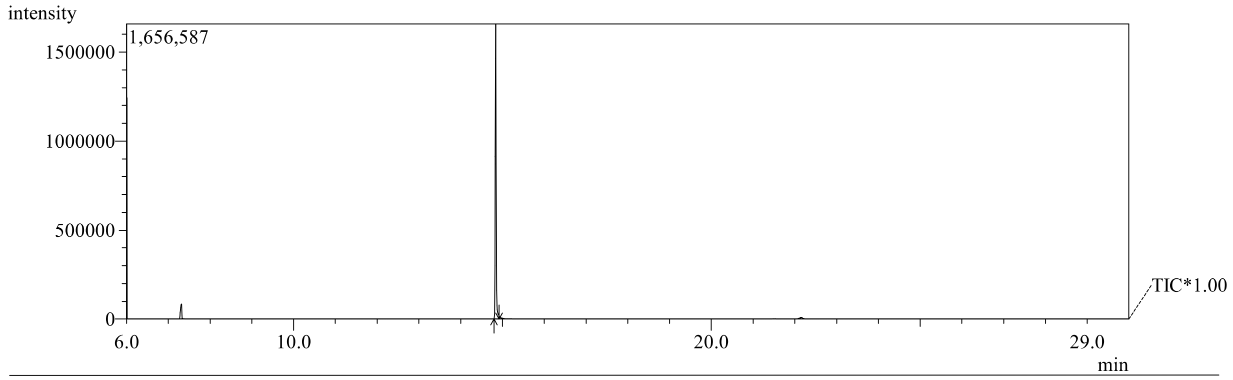

Spectrum

MassPeaks:98
RawMode:Averaged 14.8-14.9(1061-1063) BasePeak:129(300573)
BG Mode:Calc. from Peak

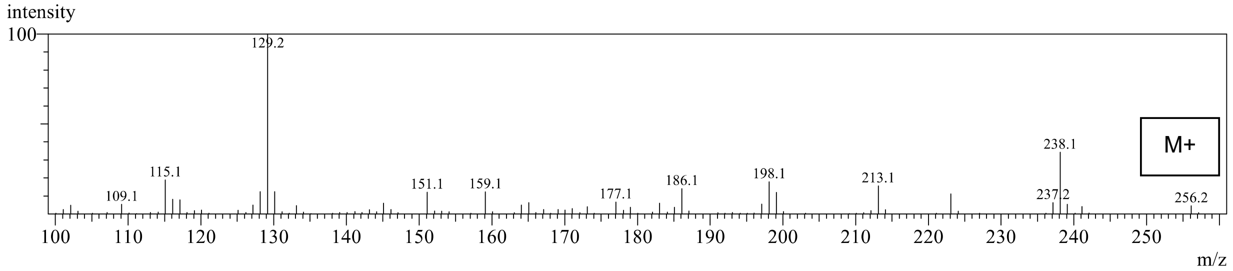

13 (3R,10aR)-1,2,3,9,10,10a-hexahydrophenanthren-3-ol
1H NMR at 400.15 MHz in CDCl3

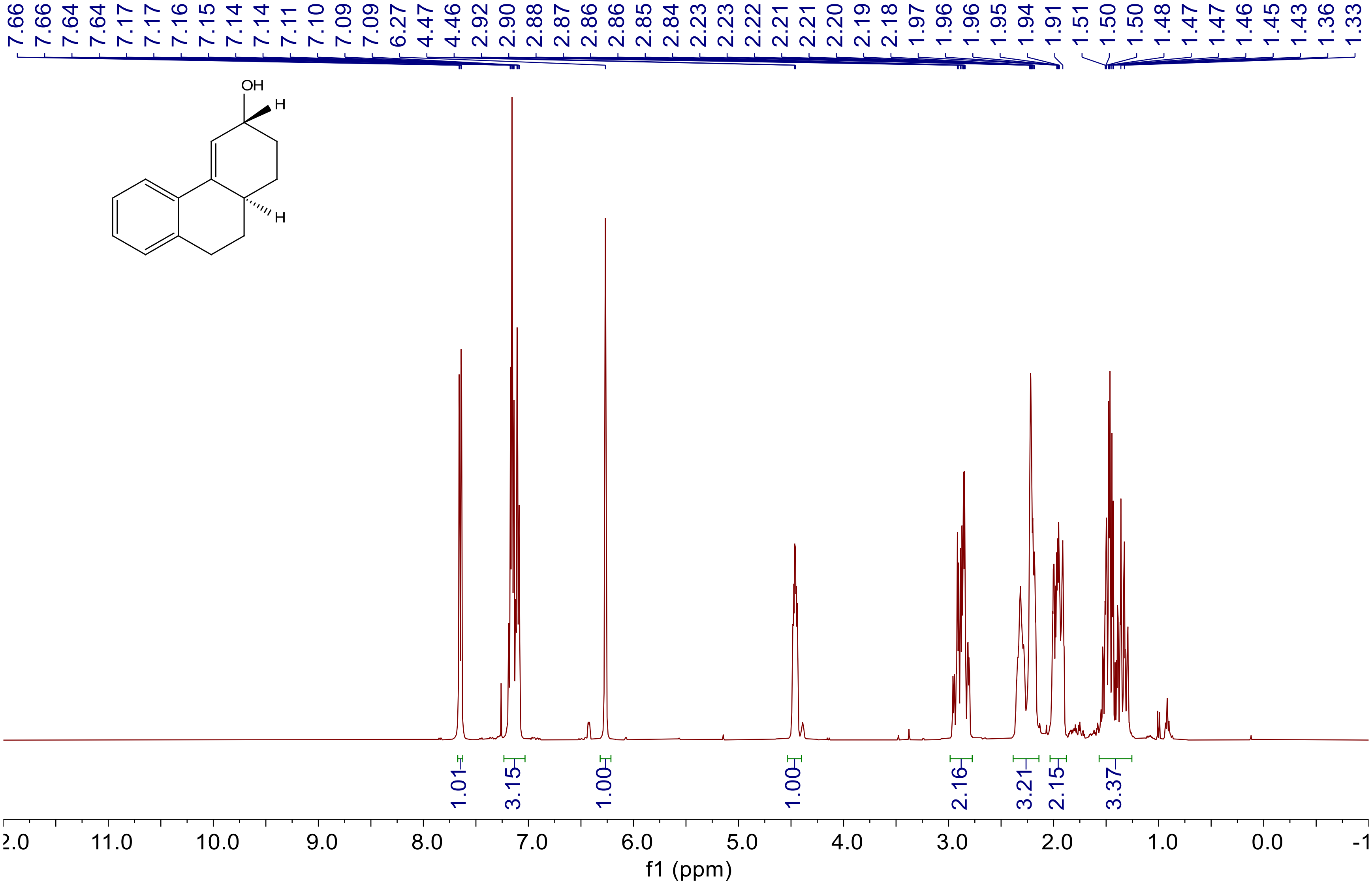

13 (3R,10aR)-1,2,3,9,10,10a-hexahydrophenanthren-3-ol
13C NMR at 100.63 MHz in CDCl3

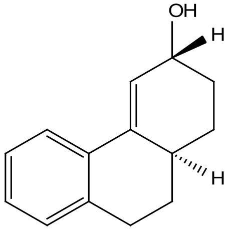

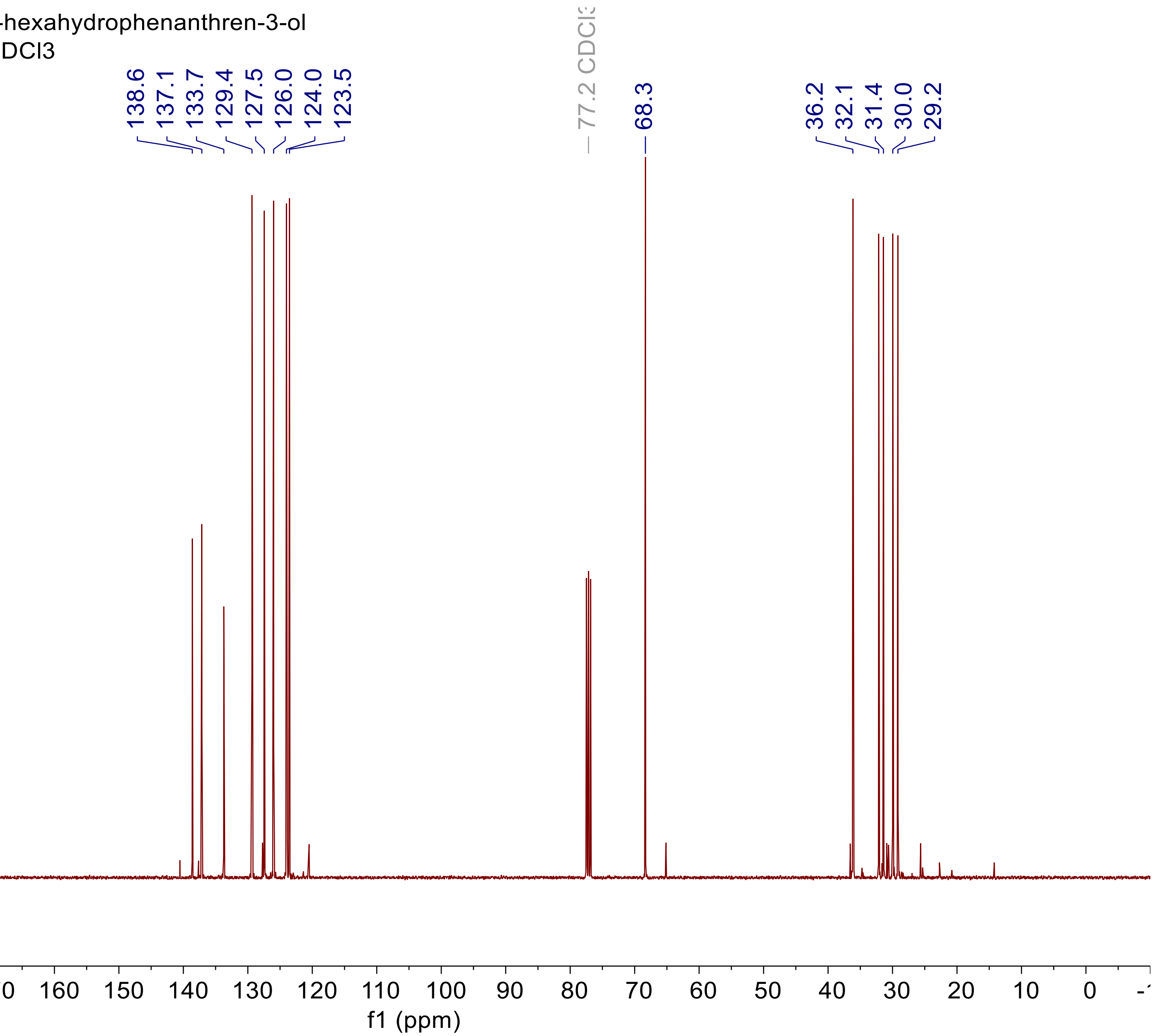

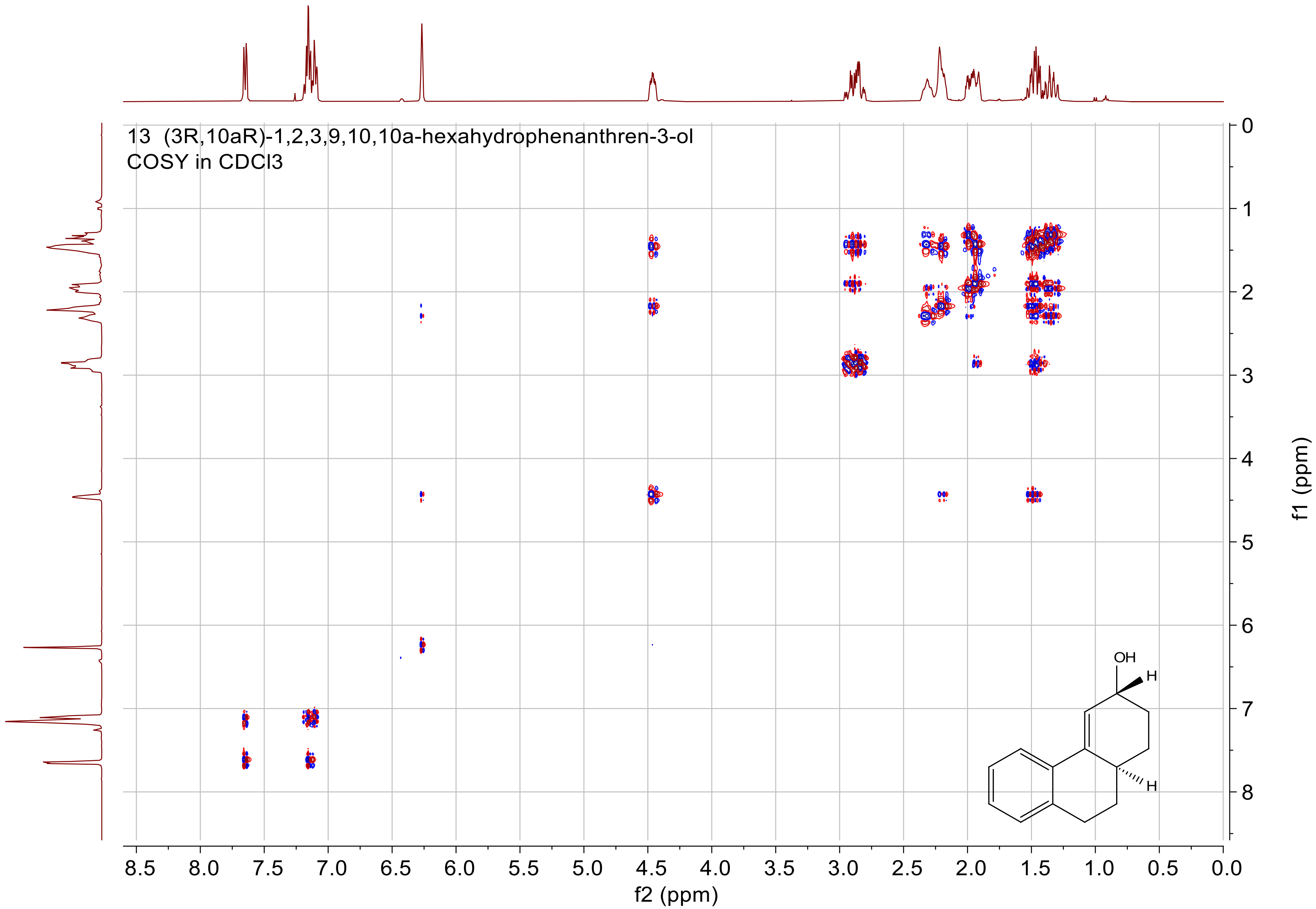
13 (3R,10aR)-1,2,3,9,10,10a-hexahydrophenanthren-3-ol
COSY in CDCl3
f1 (ppm)
f2 (ppm)
OH
H
H
8.5
8.0
7.5
7.0
6.5
6.0
5.5
5.0
4.5
4.0
3.5
3.0
2.5
2.0
1.5
1.0
0.5
0.0
0
1
2
3
4
5
6
7
8

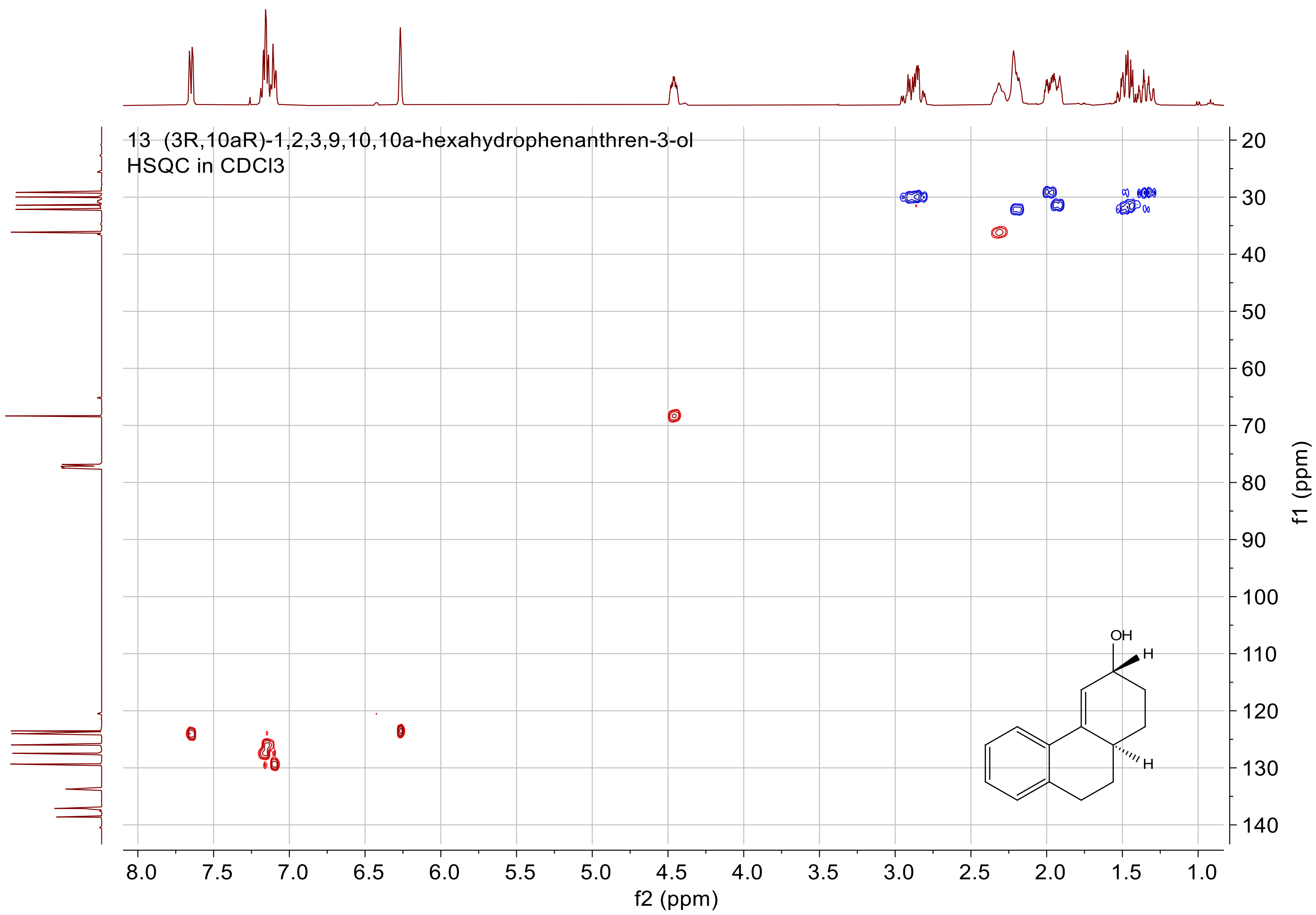
13 (3R,10aR)-1,2,3,9,10,10a-hexahydrophenanthren-3-ol
HSQC in CDCl3
OH
H
H
f1 (ppm)
f2 (ppm)
8.0
7.5
7.0
6.5
6.0
5.5
5.0
4.5
4.0
3.5
3.0
2.5
2.0
1.5
1.0
20
30
40
50
60
70
80
90
100
110
120
130
140

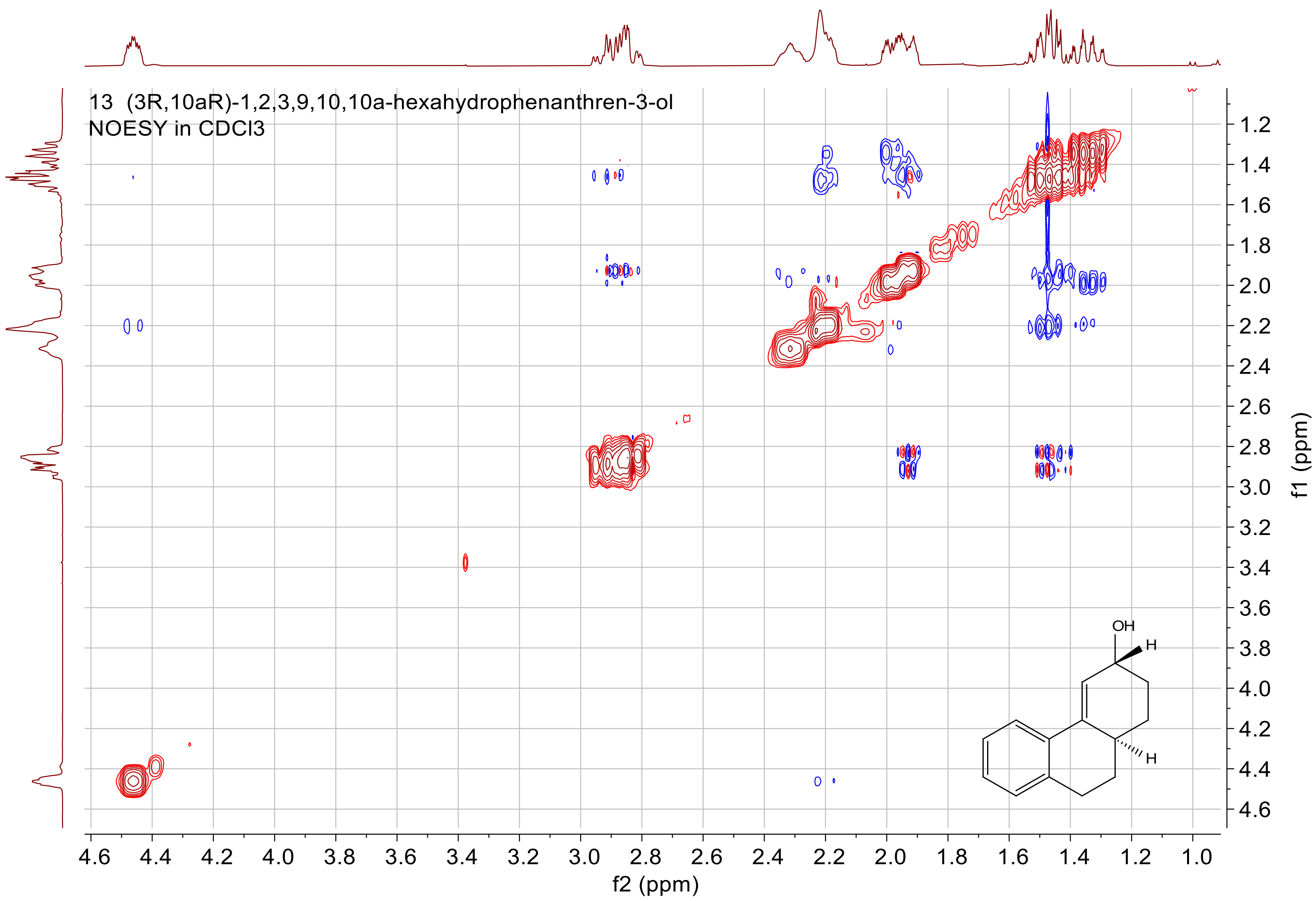

13 (3R,10aR)-1,2,3,9,10,10a-hexahydrophenanthren-3-ol
NOESY in CDCl3
OH
H
H
f2 (ppm)
f1 (ppm)

14 3-(1-methylene-1,2,3,4-tetrahydronaphthalen-2-yl)propanal
1H NMR at 400.15 MHz in CDCl3

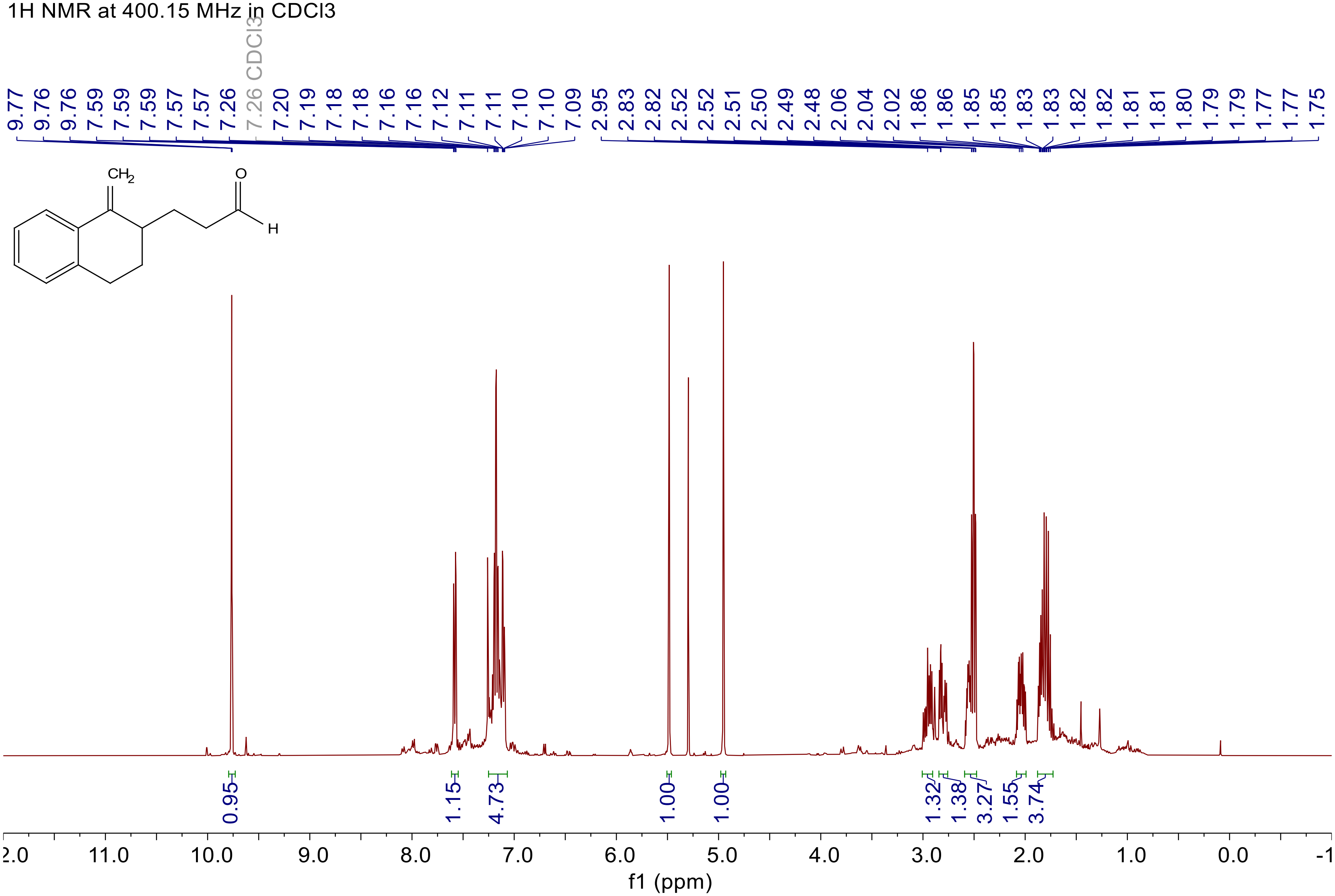

14 3-(1-methylene-1,2,3,4-tetrahydronaphthalen-2-yl)propanal
13C NMR at 100.63 MHz in CDCl3

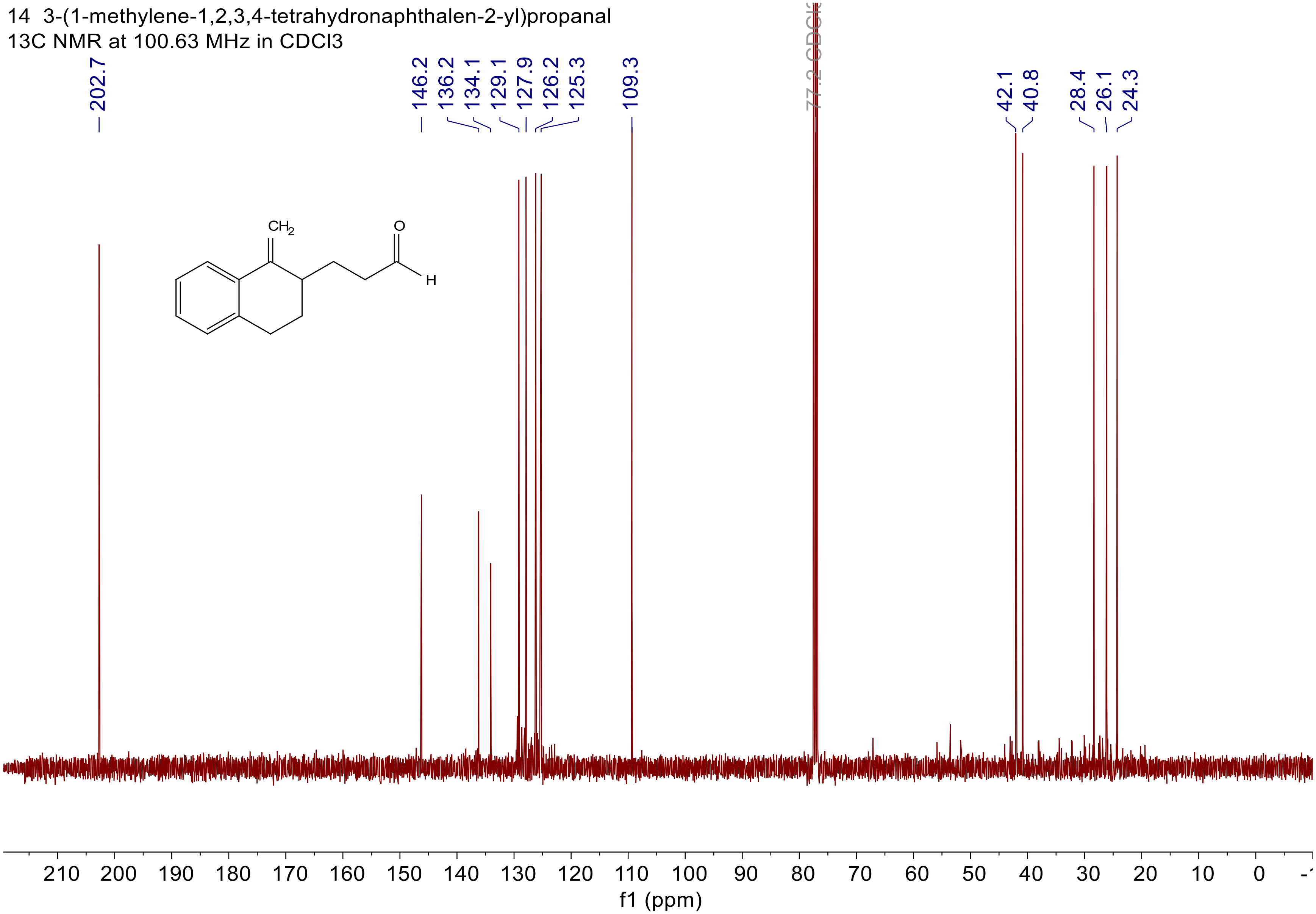

14 3-(1-methylene-1,2,3,4-tetrahydronaphthalen-2-yl)propanal

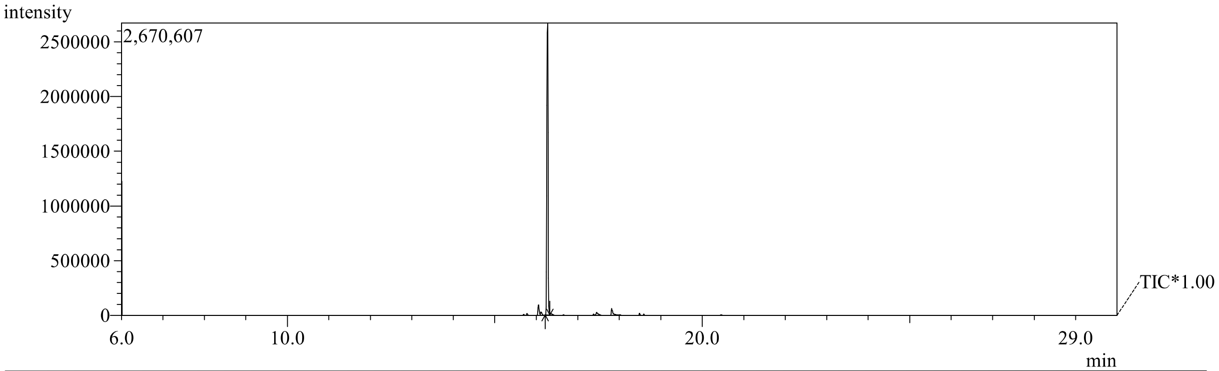

Spectrum

MassPeaks:57
RawMode:Averaged 16.3-16.3(1233-1235) BasePeak:129(458221)
BG Mode:Calc. from Peak

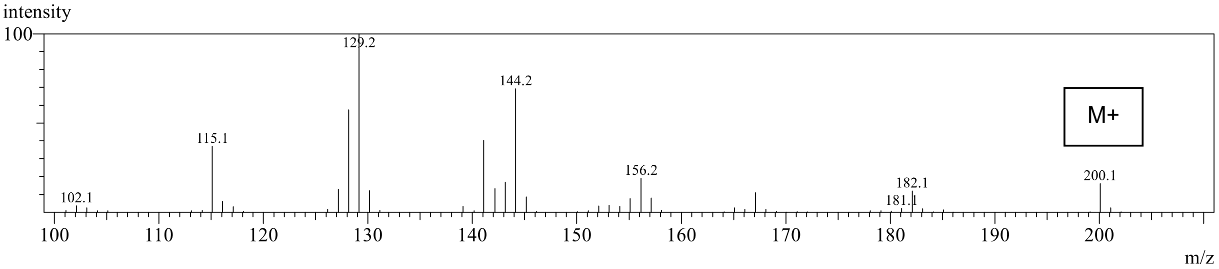

VIII. References

(1) Singh, A.; Teegardin, K.; Kelly, M.; Prasad, K. S.; Krishnan, S.; Weaver, J. D. Facile synthesis and complete characterization of homoleptic and heteroleptic cyclometalated Iridium(III) complexes for photocatalysts. *J. Organomet. Chem.* 2015, **776**, 51-59.

(2) Lujan-Montelongo, J. A.; Fleming, F. F. Preparation of 3-Oxocyclohex-1-ene-1-carbonitrile. *Org. Synth.* 2013, **90**, 229-239.

(3) Shibuya, M.; Tomizawa, M.; Iwabuchi, Y. TEMPO/$NaIO_4$—SiO_2: A Catalytic Oxidative Rearrangement of Tertiary Allylic Alcohols to β-Substituted α,β-Unsaturated Ketones. *Org. Lett.* 2008, **10 (21)**, 4715-4718.

(4) Wagh, S. J.; Chowdhury, R.; Ghosh, S. K. Pyrrolidine Catalyzed Direct Synthesis of 3,5-Diarylcyclohexenones from Acetone and Chalcones. *Curr. Organocatal.* 2014, **1 (2)**, 71-78.

(5) Latorre, A.; Urbano, A.; Carreño, M. C. Dynamic kinetic resolution in the asymmetric synthesis of atropisomeric biaryl[4] and [5]helicene quinones. *Chem. Commun.* 2009, **43**, 6652-6654.

(6) Zhong, Y.; Shing, T. K. M. Efficient and Facile Glycol Cleavage Oxidation Using Improved Silica Gel-Supported Sodium Metaperiodate. *J. Org. Chem.* 1997, **62 (8)**, 2622-2624. https://doi.org/10.1021/jo9621581.

(7) Frisch, M. J.; Trucks, G. W.; Schlegel, H. B.; Scuseria, G. E.; Robb, M. A.; Cheeseman, J. R.; Scalmani, G.; Barone, V.; Mennucci, B.; Petersson, G. A.; Nakatsuji, H.; Caricato, M.; Li, X.; Hratchian, H. P.; Izmaylov, A. F.; Bloino, J.; Zheng, G.; Sonnenberg, J. L.; Hada, M.; Ehara, M.; Toyota, K.; Fukuda, R.; Hasegawa, J.; Ishida, M.; Nakajima, T.; Honda, Y.; Kitao, O.; Nakai, H.; Vreven, T.; Montgomery Jr., J. A.; Peralta, J. E.; Ogliaro, F.; Bearpark, M.; Heyd, J. J.; Brothers, E.; Kudin, K. N.; Staroverov, V. N.; Kobayashi, R.; Normand, J.; Raghavachari, K.; Rendell, A.; Burant, J. C.; Iyengar, S. S.; Tomasi, J.; Cossi, M.; Rega, N.; Millam, J. M.; Klene, M.; Knox, J. E.; Cross, J. B.; Bakken, V.; Adamo, C.; Jaramillo, J.; Gomperts, R.; Stratmann, R. E.; Yazyev, O.; Austin, A. J.; Cammi, R.; Pomelli, C.; Ochterski, J. W.; Martin, R. L.; Morokuma, K.; Zakrzewski, V. G.; Voth, G. A.; Salvador, P.; Dannenberg, J. J.; Dapprich, S.; Daniels, A. D.; Farkas, Ö.; Foresman, J. B.; Ortiz, J. V; Cioslowski, J.; Fox, D. J. Gaussian 09, Revision C.01, Gaussian, Inc. Wallingford CT. Gaussian, Inc. Wallingford CT. 2009.

(8) Cramer, C. Essentials of Computational Chemistry: Theories and Models, 2nd ed.; John Wiley & Sons, 2004.

(9) Lee, C.; Yang, W.; Parr, R. G. Development of the Colle-Salvetti correlation-energy formula into a functional of the electron density. *Phys. Rev. B. Condens. Matter.* 1988, **37 (2)**, 785-789. https://doi.org/10.1103/physrevb.37.785.

(10) Becke, A. D. Density-functional thermochemistry. III. The role of exact exchange. *J. Chem. Phys.* 1993, **98**, 5648-5652. https://doi.org/10.1063/1.464913.

(11) Day, J. I.; Singh, K.; Trinh, W.; Weaver, J. D. Visible Light Mediated Generation of Trans-Arylcyclohexenes and Their Utilization in the Synthesis of Cyclic Bridged Ethers. J. Am. Chem. Soc. 2018, **140 (31)**, 9934–9941. https://doi.org/10.1021/jacs.8b04642.
